# Identification of candidate lethal haplotypes and genomic association with post-natal mortality and reproductive traits in Nellore cattle

**DOI:** 10.1038/s41598-023-37586-z

**Published:** 2023-06-27

**Authors:** Patrícia Iana Schmidt, Lucio Flavio Macedo Mota, Larissa Fernanda Simielli Fonseca, Danielly Beraldo dos Santos Silva, Gabriela Bonfá Frezarim, Leonardo Machestropa Arikawa, Daniel Jordan de Abreu Santos, Ana Fabrícia Braga Magalhães, John Bruce Cole, Roberto Carvalheiro, Henrique Nunes de Oliveira, Daniel Jacob Null, Paul VanRaden, Li Ma, Lucia Galvão de Albuquerque

**Affiliations:** 1 Animal Science Department, School of Agricultural and Veterinary Sciences, São Paulo State University (Unesp), Via de Acesso Paulo Donato Castellane S/N, Departamento de Zootecnia, Jaboticabal, SP 14884-900 Brazil; 2 Henry A. Wallace Beltsville Agricultural Research Center, Animal Genomics and Improvement Laboratory, Agricultural Research Service, USDA, Beltsville, MD 20705-2350 USA; 3 Department of Animal and Avian Sciences, University of Maryland, College Park, 20742 USA; 4 National Council for Scientific and Technological Development (CNPq), Brasília, Brazil

**Keywords:** Genetics, Animal breeding, Functional genomics, Genetic association study, Genetic markers, Genomics, Haplotypes, Population genetics

## Abstract

The wide use of genomic information has enabled the identification of lethal recessive alleles that are the major genetic causes of reduced conception rates, longer calving intervals, or lower survival for live-born animals. This study was carried out to screen the Nellore cattle genome for lethal recessive haplotypes based on deviation from the expected population homozygosity, and to test SNP markers surrounding the lethal haplotypes region for association with heifer rebreeding (HR), post-natal mortality (PNM) and stayability (STAY). This approach requires genotypes only from apparently normal individuals and not from affected embryos. A total of 62,022 animals were genotyped and imputed to a high-density panel (777,962 SNP markers). Expected numbers of homozygous individuals were calculated, and the probabilities of observing 0 homozygotes was obtained. Deregressed genomic breeding values [(G)EBVs] were used in a GWAS to identify candidate genes and biological mechanisms affecting HR, STAY and PNM. In the functional analyses, genes within 100 kb down and upstream of each significant SNP marker, were researched. Thirty haplotypes had high expected frequency, while no homozygotes were observed. Most of the alleles present in these haplotypes had a negative mean effect for PNM, HR and STAY. The GWAS revealed significant SNP markers involved in different physiological mechanisms, leading to harmful effect on the three traits. The functional analysis revealed 26 genes enriched for 19 GO terms. Most of the GO terms found for biological processes, molecular functions and pathways were related to tissue development and the immune system. More phenotypes underlying these putative regions in this population could be the subject of future investigation. Tests to find putative lethal haplotype carriers could help breeders to eliminate them from the population or manage matings in order to avoid homozygous.

## Introduction

Nellore is the most important beef breed raised in Brazil. The use of artificial insemination has allowed great diffusion of superior genetic material across different production systems, but it has also increased the excessive use of specific paternal lineages^1^. The widespread use of a few Nellore bulls has fixed long different homozygous segments in the genomes of more than 70% of the animals in a population^2^. These homozygous segments indicate the occurrence of inbreeding that leads to an increased accumulation of deleterious alleles, increasing the frequency and expression of harmful recessive genes^3–5^. In specialized beef cattle (Angus, Charolais, Hereford and Limousin) and dual-purpose (Simmental), where artificial insemination is extensively implemented, autosomal recessive lethal loci at moderate frequencies that significantly impact economically traits, have been recently found^6^.

The traditional methodology for identifying genetic factors related to lethal recessive variants causing defects or death, is to trace the common ancestors of the affected animals using pedigree information. As this method requires information for the target phenotype, it is not able to detect the deleterious genetic variants that cause embryonic losses, which is generally not observed^7^. With the availability of a large number of SNPs, combining genotypes with individuals pedigreed information enables to unraveling the genetic background of rare disorders in the cattle population^8^. Using genomic information, recessive deleterious variants have been identified using a statistical depletion test or by the absence of determined haplotypes in a homozygosity state^9^. This method represents a powerful tool to identify deleterious haplotypes that are common in the population without using any phenotypes, whereby its power for detecting is directly dependent on the number of genotyped animals^9^.

Growing interest has been directed toward identifying marker variants linked to lethal genomic elements by genotyping a large number of animals using SNP chips and applying a haplotype-based approach. This approach is useful due to its efficiency in capturing deleterious variation linked to some of the SNP markers, while most markers are expected to be in low linkage disequilibrium with the causal variant. Several authors have implemented and adapted this approach to identify and track lethal recessive haplotypes in different populations such as dairy breeds^8,10–12^, beef cattle^6,13^, horse^14^, pigs^15^ and dairy sheep^16^.

Identifying and monitoring lethal recessive haplotypes in a population can help breeders to anticipate problems caused by disadvantageous variants. Through the implementation of selection and mating strategies and, potentially, gene editing, problems which compromise post-natal survival and even reproduction into adulthood, could be avoid^17^. In this framework, reproduction inefficiency has an important effect on reductions of profitability in cattle^18^. Over the last few decades, increased attention has been directed toward reproduction efficiency and sexual precocity traits in Nellore cattle breeding goals^19^. In addition, advances in high-density genotyping have enabled the implementation of genomic selection jointly for production and reproduction traits in Nellore cattle^19,20^, but screening for recessive lethal alleles has not yet been applied in Nellore cattle.

Recessive lethal alleles show an impact on reproduction efficiency by embryonic death when an embryo is homozygous for it or result in reduced viability of embryos. Moreover, markers associated with lethal genetic element can also be related to reduced conception rates and longer calving intervals in adult cows, besides abortions and stillbirths^6^. If the recessive lethal allele in a heterozygote stage is associated with a positive effect on phenotypic expression of economic traits, it would lead to the selection of animals carrying the recessive lethal allele for the next generation, causing its propagation across the population^21^. Hence, this study was carried out to map lethal recessive haplotypes based on expected population frequencies of homozygous haplotypes; and detect the significant SNP markers surrounding the lethal haplotypes region for heifer rebreeding (HR), post-natal mortality (PNM) and stayability (STAY) in Nellore cattle using the genome-wide association study approach (GWAS). In addition, we in silico identified candidate genes and biological mechanisms that could influence these traits.

## Material and methods

The Nellore data base belonged to three commercial breeding programs (DeltaGen, Paint—CRV Lagoa and Cia de Melhoramento), which are part of Alliance Nellore database (www.gensys.com.br). The animals, born between 1984 and 2019, were from 276 commercial herds widely distributed in the Midwest, Southeast, and Northeast of Brazil with high connectedness by the common sires intensively used through artificial insemination (AI), with more than 50% of the calves born from AI.

### Pedigree and genotypic data information

Pedigree information recovered from historical records comprised 602,248 animals and pedigree evaluation using the INBUPGF90 program^22^, identified that 2.36% of the animals were inbred with an average inbreeding coefficient of 2.72 ± 3.96%. Those values may be underestimated since these records were incomplete due to the use of multiple-sire mattings; 40.4% and 18.7% of pedigrees included unknown sires and dams, respectively. Unknown dams were part of the base population. Genotype inconsistences between parents and progeny were adjusted using conflict.f90 software, which corrects for mendelian errors and fills missing SNP using parental genotypes^23^. A total of 62,022 Nellore animals, 24,042 females and 37,980 males, were genotyped with different Bead chip assay (Table 1). The animals genotyped with the lower density panel were imputed directly to the Illumina BovineHD panel using FImpute v2.2^24^ with expected accuracy higher than 0.97^25^.

**Table 1:** Number of animals and SNP markers for each marker panel used for genotyping animals used in the study.

Chip	Manufacturer	Number of males	Number of females	SNP number
Zoetis ZL4 (Kalamazoo, MI, USA)	Zoetis	721	307	17,794
GeneSeek® Genomic Profiler 20 K—Indicine (Lincoln, NE, USA)	Neogen	145	–	19,720
GGP Bovine LDv3 (Lincoln, NE, USA)	Neogen	49	–	26,151
Zchip (Araçatuba, SP, BR)	Deoxi	6,617	4,210	27,553
Zoetis Custom SNP ZL5 (Kalamazoo, MI, USA)	Zoetis	11,703	2,878	29,842
GGP Bovine LDv3 (Lincoln, NE, USA)	Neogen	1	54	30,108
GGP Indicus 35 K (Lincoln, NE, USA)	Neogen	10,526	9,334	35,339
Illumina BovineSNP50 (San Diego, CA, USA)	Illumina	870	429	54,609
GGP Indicus 50 K (Lincoln, NE, USA)	Neogen	1,569	2,198	54,791
GeneSeek® Genomic Profiler 75 K—Indicine (Lincoln, NE, USA)	Neogen	3,556	1,372	74,153
Illumina BovineHD BeadChip (San Diego, CA, USA)	Illumina	2,223	3,260	777,962

Genotype quality control was performed using the SNPStats package version 1.46.0^26^ of R software version 4.2.1. Markers were removed if they were non-autosomal or presenting a GenCall score lower than 0.90. SNP at the same genomic position or monomorphic were removed considering the ARS-UCD1.2 *Bos taurus* genome assembly^27^. After editing 62,022 genotyped animals, 612,154 autosomal SNP markers remained in genomic data set to carry out the further analyses.

### Phenotypic data information

The animals were raised in grazing systems, with differences in nutritional levels. In some herds, animals received protein and mineral supplementation, especially during the dry season, while in others, only urea supplementation was offered. In general, heifers were exposed to two breeding seasons: a sexual precocity test in an anticipated breeding season, occurring usually in the first trimester (January to March) for 60 days, with heifers being exposed to the first mating at around 16 months of age (irrespective of body weight and body condition score). Heifers that don't conceive in the anticipated breeding season have a second chance in a regular breeding season (November to January), approximately 8 months later, at the age of ~ 24 months. This sexual precocity test, began in the 1990s^27^, is carried out to identify precocious heifers in an out-of-season breeding and generate data to be used in genetic evaluations. On the other hand, some breeders started to expose females to reproduction around 14 months, at a breeding season for 90 days, aiming to intensify selection process for sexual precocity. Reproduction is carried out through artificial insemination or natural mating. When a fixed time AI protocol was used, the entire contemporary group received the same protocol. The diagnosis of pregnancy in heifers is performed by rectal palpation approximately 60 days after the end of the breeding season; females that did not conceive either in the first or second breeding season were discarded.

The reproduction efficiency related trait evaluated was heifer rebreeding (HR), which was determined attributing a value of 1 (success) or 0 (failure) to females that presented or not a second calf, respectively. Stayability (STAY) was determined by attributing a value of 1 (success) to cows with at least three calving's up 76 months and, otherwise, a value of 0 (failure). Post-natal mortality (PNM) was determined considering a value of 1 (mortality) for parents of dead calves that have not reached weaning and 0 (survived) for animals that were evaluated at weaning.

Contemporary groups (CG) for HR and STAY were defined considering information regarding the weaning of the first calf, considering the year and birth season, sex, herd, management group (at birth and weaning) and weaning date. For PNM the CG were formed by year and season at birth, farm, management group at birth and for animals evaluated at weaning were include also the variables sex, management group at weaning and weaning date. The season of birth was divided into two from August to January and from February to July. The CG with less than five animals or in which all scores for HR, STAY and PNM were the same, i.e., groups without variability, were eliminated. The descriptive statistics of the phenotypic data are shown in Table 2.

**Table 2:** Description of the data set for heifer rebreeding (HR), post-natal mortality (PNM) and stayability (STAY) of Nellore animals.

Description	Traits		
	HR	PNM	STAY
Nº of animals in the pedigree	1,348,163	566,364	1,348,163
Nº of animals with record	178,152	389,566	137,620
Nº of contemporary group	8,831	13,626	6,815
Mean cow age (months)	33.82 ± 3.84	60.14 ± 25.42	–
Frequency* (%)	59.34	2.12	47.38

*Success for HR is defined by to females that presented a second calf; for STAY to cows with at least three calving's up 76 months; for PNM represents the percentage of dead calves.

### Identification of candidate lethal haplotypes

The haplotypes were constructed using the sliding windows method implemented in findhap.f90 software v3^23^. By default, the program first examined haplotypes of 2,000 markers on the same chromosome, then 632 markers, and finally identified haplotypes with up to 200 markers, used for further analyses. The haplotypes with the highest frequencies (> 2%), which were never observed in a homozygous state, were prioritized for examination due to their potential significance and to enhance the likelihood of detection^23^.

Following VanRaden et al.^23^, two methods were used to calculate the expected number of homozygous individuals for each haplotype: (1) Simple method—it was assumed that all members of the population were on random mating over time and the formula used to calculate the expected number of homozygotes was to divide the number of genotyped individuals by 4 and then multiply it by the square of the carrier frequency of haplotypes; and (2) Mating method—the actual mating pattern (observed natural mating and AI) generating the genotyped individuals in the population. In this case, the number of carrier mating sire × carrier maternal grandsire pairs divided by 4 was used to obtain the expected number of homozygous. Allele frequencies for maternal granddams and maternal grandsire were assumed to be equal.

The probabilities of observing 0 homozygotes when *n* is expected were obtained by 2 analogous formulas that were used to obtain above expectations, following VanRaden et al.^9^ and Jenko et al.^6^. The first test, for the simple method the probability is: $Phh = {(1 - {C}^{2}/4)}^{N}$, where the probability of no homozygous animals $(Phh)$ depends on the carrier frequency of the heterozygous animals $(C)$ and the number of genotyped animals $(N)$. For the mating method that used the actual mating pattern, probability follows a Bernoulli process and is equal to 0.75 raised to the power of the observed number of carrier service sire × carrier maternal grandsire pairs.

Due to thousands haplotypes that could have 0 homozygotes by chance, to define a putative recessive lethal haplotype region, we set the following conditions, following Wu et al.^8^: (1) the haplotype carrier frequency had to be higher than 2%, (2) the number of expected homozygous individuals for the haplotype had to be higher than 1 (3) the probabilities of observing 0 homozygotes had to be < 0.6. All the haplotypes satisfying these conditions were selected.

### Genome-wide association analyses

GWAS were performed to identify significant SNP's within or close to the regions of the candidate's lethal haplotype, and were performed in two steps: in the first step the single-trait threshold animal model was used to estimate the breeding values (EBVs) for HR, PNM and STAY using gibbsf90 + program of blupf90 family^22^ considering the following general model:

$${\mathbf{l}} = {\mathbf{X\beta }} + {\mathbf{Za}} + {\mathbf{e}}$$

where ${\mathbf{l}}$ is the vector associated with underlying liabilities for HR, PNM and STAY; ${{\varvec{\upbeta}}}$ is the vector of fixed effect of CG; ${\mathbf{a}}$ is the additive effect of the animal and ${\mathbf{e}}$ is the residual effect. The ${\mathbf{X}}$ and ${\mathbf{Z}}$ are the incidence matrices related to fixed effects of CG and random effect of animals. In the model an underlying distribution was considered as follows: ${\text{f}}({\text{l}}|{\text{l}}_{{\text{i}}} ) = { }\mathop \prod \limits_{{{\text{i}} = 1}}^{{{\text{n}}_{{\text{i}}} }} 1\left( {{\text{l}}_{{\text{i}}} < {\text{t}}_{{\text{i}}} } \right)1\left( {{\text{l}} = 0} \right) + 1\left( {{\text{l}}_{{\text{i}}} > {\text{t}}_{{\text{i}}} } \right)1\left( {{\text{l}} = 1} \right)$, where l represents the binary trait (0 or 1) HR, PNM and STAY, ${\text{l}}_{{\text{i}}}$ represents the underlying liability for the binary observation i, ${\text{t}}_{{\text{i}}}$ is the threshold that defines the binary response for the ${\text{l}}$ scale and ${\text{n}}_{{\text{i}}}$ is the number of information for each trait.

The random effects of animal and residual were assumed to be normally distributed: ${\mathbf{a}}\sim (0,{\mathbf{A}}{\upsigma }_{{\text{a}}}^{2}$) and ${\mathbf{e}}\sim (0,{\mathbf{I}}{\upsigma }_{{\text{e}}}^{2}$), where ${\mathbf{A}}$ is the relationship matrix based on pedigree information, ${\mathbf{I}}$ is the identity matrix, ${\upsigma }_{{\text{a}}}^{2}$ is the additive genetic variance and ${\upsigma }_{{\text{e}}}^{2}$ is the residual variance, respectively. The heritabilities obtained for HR, STAY and PNM were 0.31 ± 0.011, 0.33 ± 0.015 and 0.41 ± 0.039, respectively.

Prior to performing the GWAS analysis, EBVs obtained in the first-step were deregressed following Garrick et al.^28^, and only the animals presenting deregressed EBVs (dEBV) with a minimum accuracy of 0.40 (based on prediction error variance), were used in the GWAS analysis. The GWAS was performed using the GCTA program^29^ for HR (n. 43,250 animals), STAY (n. 42,787 animals) and PNM (n. 25,330 animals), following the model:

$${\mathbf{y}}* = {{\varvec{\upmu}}} + {\mathbf{bx}} + {\mathbf{e}}$$

where ${\mathbf{y}}*$ is the n × 1 vector of dEBVs for the traits: HR, PNM and STAY; ${{\varvec{\upmu}}}$ is the overall mean, ${\mathbf{b}}$ is the additive effect (fixed effect) of the candidate SNP to be tested for association, ${\mathbf{x}}$ is the SNP genotype indicator variable coded as 0, 1 or 2 (for the number of copies of the allele B in genotypes AA, AB, and BB), and **e** is the vector of residual effects. For statistical tests, the SNP effects were standardized as follows: ${\text{t}}_{{\text{n}}} = \frac{{{\hat{\text{g}}}_{{\text{n}}} }}{{{\text{SE}}\left( {{\hat{\text{g}}}_{{\text{n}}} } \right)}}$, where, ${\text{t}}_{{\text{n}}}$ is the t-values for the SNP marker effect; ${\hat{\text{g}}}_{{\text{n}}}$ is the SNP effects for each trait and ${\text{SE}}\left( {{\hat{\text{g}}}_{{\text{n}}} } \right)$ is the standard error for SNP effect (${\hat{\text{g}}}_{{\text{n}}}$). The *p*-values for the SNP effects were computed as ${\text{p}} - {\text{value}} = 2\left( {1 - \phi \left( {\left| {{\text{t}}_{{\text{n}}} } \right|} \right)} \right)$, where $\phi \left( {\left| {{\text{t}}_{{\text{n}}} } \right|} \right)$ is the cumulative function of the t-distribution. The SNP markers estimated for the traits were deemed significant when − log10(*p*-value) > 6 (Bonferroni correction). To identify genes, a region of 100 kb down and upstream of each significant SNPs was considered within or close to the region of candidate lethal haplotypes (Supplementary material—Table S1, S2 and S3). Also, common SNP with significant effect for PNM, HR and STAY (Supplementary material—Table S4) and alleles of SNPs within the putative lethal haplotypes for each trait (Supplementary material—Table S5), were analyzed.

### Functional gene networks

The SNP markers deemed significant were used to search the genes harboring within 100 kb down and upstream of each significant SNP marker using the BioMart tool, from the ENSEMBL software (https://www.ensembl.org/info/docs/tools/vep/index.html), considering the ARS-UCD1.2 *Bos taurus* genome assembly^30^ (Supplementary material—Table S6, S7 and S8 for HR, PNM and STAY, respectively). The ClueGO version 2.5.9^31^ plug-in of Cytoscape v3.9.0 software^32^ was used to visualize the non-redundant biological terms for clusters of genes in a functionally grouped network. Two approaches were used to functional network analysis: 1) the gene set for all traits; 2) the gene set found commonly for the three traits. Gene ontologies (GO) biological process terms, immune system, molecular function and KEGG (Kyoto Encyclopedia of Genes and Genomes) were used to find the gene-networks annotations (Supplementary material—Table S9, S10, S11 and S12, for enrichment functional analysis for genes found related to biological process, immune system, molecular function and KEGG, respectively) (Supplementary material—Table S13, S14 and S15, for GO terms related to biological process, immune system and molecular function, respectively).

### Ethics approval and consent to participate

The animal procedures in this study were approved by the Institutional Animal Care and Use Committee of the São Paulo State University (UNESP), School of Agricultural and Veterinary Science Ethical Committee (protocol number 18.340/16). Furthermore, all the data sampling was performed following the CEUA/ FCAV-UNESP guidelines and regulations according to Regulations for the Administration of Affairs Concerning Experimental Animals (Ministry of Science and Technology, Brazil). In addition, we confirmed the statement that the study was conducted following the ARRIVE guidelines.

## Results and discussion

### Candidate lethal haplotypes and genome-wide association analyses

Analyzing phased haplotypes data for 62,022 genotyped animals and 612,154 SNP markers using sliding windows approach, the maximum number of SNPs per segment was 200 with minimum segment length of 194 to match chromosome length resulting in a total of 3,073 segments. On average, 900 haplotypes were found within each segment. Out of these, 30 haplotypes had no homozygous genotypes (Table 3). The presence of these regions in Nellore cattle, which are inherited and prevalent across the population, suggests that they may contain an autosomal recessive lethal allele. The defined 194–200 SNP segments for searching lethal haplotypes average 0.78 Mb in length. This physical length was smaller than reported by Hoff et al. (2017) 13, that defined an average of 1.14 (Mb) using 20-marker fixed sliding windows.

**Table 3:** Haplotype regions and carrier frequency for putatively lethal alleles in Nellore cattle population.

Potentially lethal haplotype^1^	BTA^2^	Haplotype						Expected homozygotes numbers and probabilities of observing zero homozygous			
		Distance between haplotypes (Mb)	Start position^3^	End position^3^	Length (Mb)	Number of carriers*	Haplotype %*	Simple^4^	Probabilities (*Phh*)^5^	Mating^6^	Probabilities^7^
25.1	1	–	21,773,327	22,571,004	0.79768	5,929	4.78	141	4,07E−16	69	2,39E−09
108.7	1	69.63095	92,201,954	93,179,359	0.97741	4,750	3.83	91	1,32E−10	123	4,29E−16
231.4	2	–	30,989,280	31,660,659	0.67138	4,998	4.03	101	1,15E−11	73	7,58E−10
346.2	2	93.94106	125,601,735	126,602,788	1.00105	11,771	9.49	560	1,93E−61	368	1,05E−46
396.34	3	–	27,103,538	27,639,143	0.53561	4,291	3.46	74	8,65E−09	7	1,33E−01
461.4	3	56.52740	84,166,546	84,882,788	0.71624	5,941	4.79	142	3,51E−16	96	1,01E−12
479.2	3	12.97973	97,862,526	98,537,136	0.67461	2,783	3.05	57	5,43E−07	60	3,19E−08
676.135	5	–	19,466,593	20,216,556	0.74996	4,130	3.33	69	3,40E−08	2	5,63E−01
769.3	5	79.20781	99,424,373	100,174,866	0.75049	4,143	3.34	69	3,07E−08	26	5,64E−04
966.2	7	–	19,468,345	20,417,099	0.94875	6,053	4.88	148	9,09E−17	58	5,67E−08
988.14	7	16.13788	36,554,986	37,251,910	0.69692	4,329	3.49	76	6,26E−09	26	5,64E−04
1045.24	7	43.68097	80,932,886	81,622,237	0.68935	4,564	3.68	84	7,57E−10	3	4,22E−01
1077.1	7	25.22425	106,846,493	107,525,464	0.67897	6,958	5.61	196	6,29E−22	170	5,76E−22
1184.1	8	–	78,277,928	79,262,115	0.98419	3,832	3.09	59	3,71E−07	37	2,38E−05
1192.1	8	5.68177	84,943,889	85,809,360	0.86547	12,441	10.03	625	1,48E−68	588	3,44E−74
1277.303	9	–	42,710,447	43,514,053	0.80361	4,056	3.27	66	6,29E−08	5	2,37E−01
1278.37	9	0.00169	43,515,752	44,346,392	0.83064	3,733	3.01	56	7,91E−07	4	3,16E−01
1283.33	9	3.79844	48,144,835	48,978,466	0.83363	3,981	3.21	63	1,15E−07	4	3,16E−01
1312.67	9	23.54260	72,521,066	73,292,804	0.77174	3,721	3	56	8,68E−07	4	3,16E−01
1457.2	10	–	85,722,335	86,530,381	0.80805	4,378	3.53	77	4,05E−09	55	1,34E−07
1655.2	12	–	31,305,864	32,200,052	0.89419	4,242	3.42	72	1,33E−08	79	1,35E−10
2213.8	17	–	65,952,250	66,468,921	0.51667	3,696	2.98	55	1,05E−06	9	7,51E−02
2237.1	18	–	9,785,118	10,380,109	0.59499	4,775	3.85	92	1,04E−10	4	3,16E−01
2270.1	18	25.88554	36,265,657	37,406,377	1.14072	3,795	3.06	58	4,94E−07	38	1,79E−05
2329.1	19	–	20,292,526	21,133,267	0.84074	5,358	4.32	116	2,69E−13	55	1,34E−07
2371.1	19	33.51829	54,651,560	55,237,521	0.58596	15,145	12.21	927	2,63E−101	1028	3,66E−129
2585.3	22	–	18,857,788	19,688,790	0.831	4,192	3.39	71	1,82E−08	78	1,80E−10
2620.22	22	27.00519	46,693,982	47,385,511	0.69153	4,180	3.37	70	2,25E−08	10	5,63E−02
2724.2	24	–	18,329,342	19,105,911	0.77657	4,651	3.75	87	3,38E−10	76	3,20E−10
3063.3	29	–	40,482,004	41,207,696	0.72569	6,189	4.99	154	1,69E−17	163	4,32E−21

*The number of carriers and frequency of haplotype among the heterozygous animals was calculated on the number of genotyped animals: 62,022.

^1^Haplotypes are identified by DNA segment number and haplotype within segment (e.g., 2371.1 indicates segment 2371 and haplotype 1).

^2^*Bos taurus* Autosome.

^3^ARS-UCD1.2 *Bos taurus* genome assembly (Rosen et al., 2020)^27^.

^4^Number of individuals genotyped (62,022) divided by 4 and multiplied by square of carrier frequency. The simple expected number of homozygotes does not require dividing by 4 if the haplotype frequency is used directly instead of the carrier frequency.

^5^ $Phh = {(1 - {C}^{2}/4)}^{N}$, where the probability of no homozygous animals $(Phh)$ depends on the carrier frequency of the heterozygous animals $(C)$ and the number of genotyped animals$(N)$.

^6^Number of carrier service sire × carrier maternal grandsire mattings divided by 4.

^7^Follows a Bernoulli process and is equal to 0.75 raised to the power of the observed number of carrier service sire × carrier maternal grandsire pairs.

Using two tests to search for haplotypes carrying possible recessive lethal alleles based on the number of expected recessive homozygous individuals in the entire population or from mattings between carriers, we found 30 potential lethal haplotypes that exhibited higher expected homozygotes, with a minimum frequency of 2% in the population (Table 3). When using the simple and mating methods, the probability of observing zero homozygous ranged from 2.63e-101 to 1.05e-06 and 3.66e-129 to 0.56, respectively (Table 3). It is desirable to have small probabilities of homozygous markers for lethal haplotypes in genotyped animals to ensure their fitness and prevent harmful effects on animal performance. VanRaden et al.^9^ screened genomic regions with fewer homozygous animals than expected in the American Holstein population and identified the potential haplotypes showing lethal variants. The detected thirty chromosomal regions identified to harbor lethal haplotypes were mapped on 15 chromosomes (1, 2, 3, 5, 7, 8, 9, 10, 12, 17, 18, 19, 22, 24, and 29) with a frequency for animals carrying recessive markers varied from 2.98% to 12.21% (Table 3). The frequency of carrier animals with the lethal haplotype is typically twice as high as the mean frequency of the lethal haplotype, as heterozygous animals only contribute 0.50 to the allele frequency.

In general, the observed average for the frequency of potentially lethal haplotypes of 4.41% shows a slight difference from those reported by Sahana et al.^33^ in a Nordic Holstein cattle population, 3.5% (ranging from 2.7% to 6.7%) on 17 haplotypes harboring possible recessive lethal alleles. On the other hand, we observed a possible lethal haplotypes carrier frequency 3.9 times lower than that found by Jenko et al.^6^ of 17.22% (1.2%–33.5%) in five beef cattle breeds. Those authors suggested that these high haplotype frequencies may come from their substitution effect with pleiotropy or linked selection on target traits goals of breeding programs.

The expected homozygous number obtained through the method considering random mating was overall greater (ranging from 55 to 927, average 150.4; under ‘Simple’ in Table 3) than that from the mating method (‘Mating’; ranging from 2 to 1,028, average 110.6). The greater values observed for detecting homozygous markers using random mating were expected due to the population showing multiple sire mattings in the breeding season. This reduces the accuracy of pedigree information to capture the progenitors carrying lethal haplotypes across generations in the evaluated population. In this context, using complete pedigree information and almost all ancestral genotypes, it is expected that the number of homozygous identified using both methods will tend to be similar^9^.

We found new candidate regions, different from those previously observed in the literature for beef cattle. Obviously, at least some of the differences could be due to sampling variation. Then again, one of the reasons for this difference might be due to the genetic distance between *Bos Indicus* and *Bos Taurus*, which despite having a common ancestor, present several morphological and physiological differences^34^. In accord, Lin et al.^35^ reported that the calculated distribution of minor allele frequencies and heterozygosity suggests that the genetic diversity of *Bos indicus* populations is lower than that of *Bos taurus* populations. The haplotypes found in this study may be specific to the species or even the Nellore breed, with emphasis on those with the highest frequencies: 2371.1 in BTA19, with more than 15,000 carriers, 1192.1 in BTA8 with more than 12,000 carriers and the 346.2 on the BTA2 found with over 11,000 carrier candidates. In addition, adjacent segments showing similar number of carriers also might be a good confirmation, indicating that several segments may all be tracking the same lethal variant, such as on BTA 3 (461.4 and 479.2), BTA 7 (966.2; 988.14; 1045.24 and 1077.1), BTA 8 (1184.1 and 1192.1) and BTA 9 (1277.303; 1278.37 and 1283.33) (Table 3).

Association analysis of lethal haplotypes for reproductive performance and post-natal mortality in Nellore cattle indicated that some haplotypes had significantly on the traits (Table 4). On BTAs 1, 2 (30.99–31.66 Mb), 3 (27.10–27.64 Mb and 84.17–84.88 Mb), 5(99.42–100.17 Mb), 7, 8 (84.94–85.81 Mb), 9 (48.14–48.98 Mb), 12, 18 show a significant harmful effect increasing PNM and reducing HR and STAY (Table 4). On the other hand, only four haplotypes had any significant effect on HR and STAY (Table 4). The presence of lethal alleles in a population across generations may occurs due to some marker alleles being linked with alleles that exhibits favorable effect on phenotypic expression. Fasquelle et al.^36^ and Jenko et al.^6^ observed that an increase in the frequency of lethal alleles in the population is due to heterozygous animals exhibit a favorable performance for the target trait, in which the presence of favorable allele copies to mask the harmful effect of the lethal allele.

**Table 4:** Association effect of the potential lethal haplotype for post-natal mortality (PNM), heifer rebreeding (HR) and stayability (STAY) in Nellore cattle.

Haplotype number	^1^BTA	^2^Start position (Mb)	^2^End position (Mb)	Nº of SNP markers	Post-natal mortality			Heifer Rebreeding			Stayability		
					^3^Effect	Standard error	t-test (*p*-value)	^3^Effect	Standard error	t-test (*p*-value)	^3^Effect	Standard error	t-test (*p*-value)
1	1	21.77	22.57	199	0.039	0.00863	0.046	− 0.092	0.00773	< .0001	− 0.084	0.00109	< .0001
7	1	92.20	93.18	199	0.045	0.00863	0.017	− 0.125	0.00775	< .0001	− 0.109	0.00109	< .0001
2	2	30.99	31.66	199	0.042	0.00865	0.03	− 0.129	0.00776	0.00763	− 0.05	0.00109	0.0001
4	2	125.60	126.60	199	0.015	0.00868	0.053	− 0.102	0.00781	0.005	− 0.091	0.0011	< .0001
2	3	27.10	27.64	200	0.081	0.01066	1.00E−04	− 0.06	0.00778	< .0001	− 0.08	0.00109	< .0001
34	3	84.17	84.88	199	0.075	0.00863	3.00E−04	− 0.108	0.00774	< .0001	− 0.04	0.00109	< .0001
4	3	97.86	98.54	197	0.034	0.00867	0.103	− 0.13	0.00781	< .0001	− 0.06	0.0011	< .0001
135	5	19.47	20.22	200	0.037	0.00868	0.062	− 0.101	0.0078	< .0001	− 0.0006	0.0011	0.6512
3	5	99.42	100.17	200	0.055	0.00872	0.01	− 0.098	0.00781	< .0001	− 0.099	0.0011	< .0001
1	7	19.47	20.42	200	0.05	0.00873	0.002	− 0.113	0.00784	< .0001	− 0.001	0.0011	0.4378
14	7	36.55	37.25	200	0.039	0.00877	0.049	0.088	0.00784	< .0001	0.018	0.0011	< .0001
2	7	80.93	81.62	199	0.069	0.00867	0.001	0.068	0.00776	< .0001	− 0.13	0.00109	< .0001
24	7	106.85	107.53	200	0.041	0.00874	5.0005	− 0.144	0.00785	< .0001	− 0.07	0.0011	< .0001
1	8	78.28	79.26	199	0.029	0.00865	0.15	− 0.027	0.00776	0.0004	− 0.052	0.00109	0.0017
1	8	84.94	85.81	200	0.082	0.00864	1.00E−04	− 0.357	0.00774	< .0001	− 0.07	0.00109	< .0001
303	9	42.71	43.51	199	0.001	0.00869	0.97	− 0.091	0.00776	< .0001	− 0.102	0.00109	< .0001
33	9	43.52	44.35	200	0.012	0.00874	0.587	− 0.036	0.00784	0.0024	− 0.063	0.0011	< .0001
37	9	48.14	48.98	199	0.086	0.00877	< .0001	− 0.111	0.00788	< .0001	− 0.15	0.00111	< .0001
67	9	72.52	73.29	200	0.06	0.00873	0.004	− 0.132	0.00782	< .0001	− 0.001	0.0011	0.7239
2	10	85.72	86.53	200	0.031	0.00866	0.144	− 0.003	0.00775	0.6454	0.002	0.00109	0.0365
2	12	31.31	32.20	196	− 0.053	0.00878	0.003	0.001	0.00789	0.432	− 0.108	0.00111	< .0001
8	17	65.95	66.47	200	0.004	0.00878	0.869	− 0.097	0.00786	< .0001	0.003	0.0011	0.1971
1	18	9.79	10.38	196	0.052	0.00877	0.014	− 0.044	0.0079	< .0001	− 0.08	0.00111	< .0001
1	18	36.27	37.41	196	0.115	0.0087	< .0001	− 0.246	0.00786	< .0001	− 0.06	0.0011	< .0001
1	19	20.29	21.13	198	0.03	0.00864	0.148	− 0.002	0.00774	0.9788	− 0.075	0.00109	< .0001
1	19	54.65	55.24	197	0.036	0.0087	0.069	− 0.052	0.00776	< .0001	− 0.01	0.00109	< .0001
22	22	18.86	19.69	197	− 0.021	0.00864	0.127	0.061	0.00775	< .0001	0.073	0.00109	< .0001
3	22	46.69	47.39	197	− 0.013	0.00868	0.529	− 0.023	0.00777	0.0008	− 0.062	0.00109	< .0001
2	24	18.33	19.11	198	0.007	0.00864	0.72	− 0.019	0.00774	0.0219	− 0.004	0.00109	0.0057
3	29	40.48	41.21	199	− 0.008	0.00869	0.678	− 0.086	0.00779	< .0001	0.081	0.00109	< .0001

^1^*Bos taurus* Autosome.

^2^ARS-UCD1.2 *Bos taurus* genome assembly (Rosen et al., 2020)^27^.

^3^Haplotype effect.

The lethal alleles carried by the influential sires in the population associated with a significant effect on female reproductive performance (lost pregnancies), can reach an important economic impact by reducing fertility. Testing animals for lethal haplotypes can help mate allocation to avoid animals that carry lethal alleles in the same locus and maximize the genetic gain rate towards a determined breeding objective^37^.

### Significant regions and genes surround lethal haplotypes

**Figure 1:**
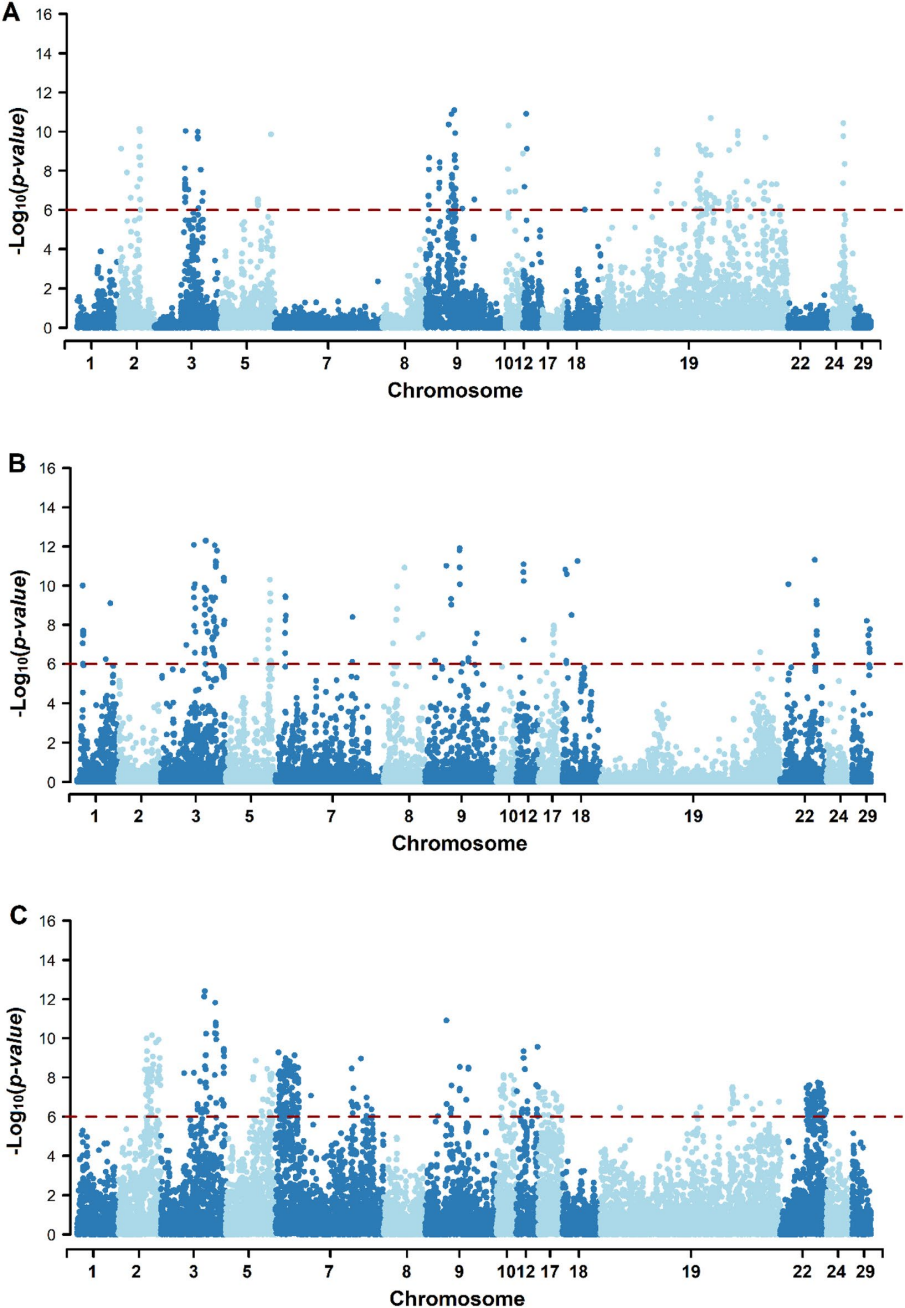
Manhattan plot of genome-wide association for SNPs in genomic regions surrounding the putative lethal haplotypes for post-natal mortality (PNM) (**A**), heifer rebreeding (HR) (**B**) and stayability (STAY) [C] in Nellore cattle. The horizontal red line represents the significance threshold − log10(*p*-value) > 6.0.

**Figure 2:**
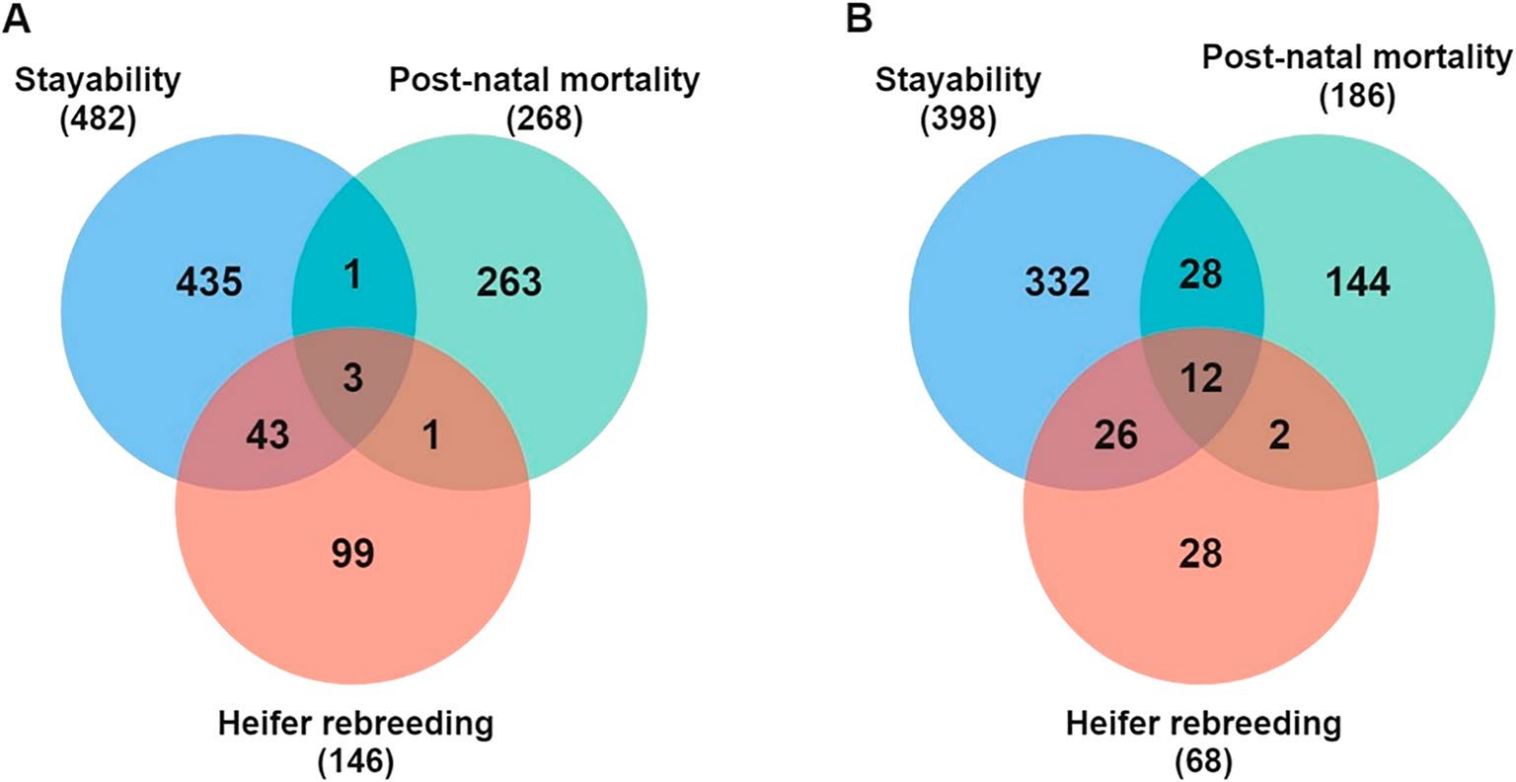
Venn diagram used to identify shared and specific SNPs (**A**) and genes (**B**) between the traits evaluated: heifer rebreeding (HR), post-natal mortality (PNM) and stayability (STAY) (Supplementary material Table S4).

The GWAS pointed out several significant SNP markers implying different physiology mechanisms that lead to harmful effects on reproductive performance (HR and STAY) and PNM in Nellore cattle (Fig. 1). A total of 146, 268, and 482 SNP markers were deemed significant at − log10(*p*-value) > 6 for HR, PNM, and STAY, respectively (Fig. 2A, Supplementary material—Table S1, S2 and S3). From those, 43 SNP markers were shared by HR and STAY, 1 marker was shared by PNM and STAY, and all traits shared 3 markers (Supplementary material—Table S4).

Moreover, we identified a total of 10 protein coding genes: *MTUS2, NFIA, A2M, A2ML1, KLRG1, M6PR, OVOS2, PHC1, BVES,* and *LIN28B*; and 2 RNAs: U6 and 5S_rRNA (Supplementary material—Table S6, S7 and S8), surrounding 200 kb of significant markers shared by HR, PNM and STAY, on BTAs 2, 3, 5, 9, 12 and 19 (Fig. 2B; Supplementary material—Table S4). On the other hand, the haplotypes identified in these BTAs showed a null mean effect for HR, while PNM exhibited (except on BTA 12) an unfavorable effect (Table 4).

### Functional gene network analysis

Genomic windows were used to map the candidate genes for each trait studied, of which 68 genes were associated with heifer rebreeding (HR), 186 with post-natal mortality (PNM), and 398 with stayability (STAY) (Supplementary material—Table S6, S7 and S8). Of the overall 572 unique genes found, 39 were non-coding RNAs, 1 was a pseudogene, and 532 were protein-coding. Non-coding RNAs play a key role in regulating gene expression^38^. Therefore, the non-coding RNAs mapped within the windows could be part of regulatory networks associated with the traits studied.

The annotated genes, protein-coding and non-coding genes with known function in ARS-UCD1.2 *Bos taurus* genome, were submitted to a non-redundant biological functional gene network analysis, a separate analysis for each trait. Most GO terms found for biological processes, molecular function, and pathways were related to tissue development, immune system, and disease-related metabolic pathways (Supplementary material—Table S9 to S15).

The GO Biological Process terms found for all genes were related to organ and tissue development as circulatory system development (GO:0072359), heart development (GO:0007507), and hemopoiesis (GO:0030097). According to Mathew and Bordoni^39^ the cardiovascular system is one of the first systems that develop in the embryo. Cardiac development occurs through a complex molecular interaction. Interference in this process, whether genetic or environmental, could lead to the formation of different heart diseases, including being lethal to the embryo. As explained above, genes related to these processes were found and associated with HR, PNM, and STAY, and could be a key to improving these traits.

The genes *LIN28B* (BTA 9) and *NFIA* (BTA 3) impacts reproductive functions by regulating adipogenesis and embryonic development^40,41^. *LIN28B* gene is associated with puberty and inhibits gonadal steroids production, while *NFIA* indirectly affects reproductive functions by regulating cellular differentiation and activating adipogenesis processes^42,43^. The markers surrounding these genes have a significant effect on reproductive functions (Supplementary material—Table S1, S2 and S3) and are linked to energy homeostasis and HPG axis functions^42,43^ and genes enhancing the metabolic signals for the start of the reproductive event in Nellore heifers^44^. In this context, the observed favorable additive effect for SNP markers within recessive lethal haplotypes region increases its frequency in the population (Supplementary material—Table S1, S2 and S3). This led to the selection of heterozygous carriers as parents of the next generation, allowing the recessive lethal allele propagates across the population, and significantly affect reproductive performance^21^. These markers contribute to heifers' rebreeding success after the first calving and their ability to stay longer in the herd with progenies (Supplementary material—Table S2 and S3).

The genes *A2M, A2ML1, KLRG1,* and *PHC1* on BTA5, shared by HR, PNM, and STAY, have a major effect on Bovine Paratuberculosis by the direct action of the genes *A2M* and *A2ML1*, while the *KLRG1* gene show an effect on inhibition of pro-inflammatory cytokines (IFN-γ and TNF-α) production^45^ (Supplementary material—Table S6, S7 and S8). Animals with increased *A2M* and *A2ML1* plasma levels and lower-expression of the *KLRG1* gene increase the progression of mycobacterial infection, inducing tuberculous lymphadenitis, pulmonary tuberculosis, and latent tuberculosis^45^. Bovine Paratuberculosis directly affects the production system by increasing on-farm post-natal mortality^46^. Elzo et al.^47^ observed that genes affecting the immunity system and incidence of paratuberculosis are associated with lower cow fertility, weight loss, and lower calf body weight at birth and at 205 days of age, which are in agreement with the unfavorable SNP effect observed for STAY (Supplementary material—Table S3).

A total of 26 genes surrounding biological processes related to energy metabolites and immune systems are shared by HR and STAY (Fig. 2B—Supplementary material-Table S6 and S8). On BTA3 (99.11 Mb–99.34 Mb), which showed an unfavorable effect for expression of HR and favorable for STAY (Table 4), mapping the genes *CYP4A11, CYP4A22, CYP4B1*, and *CYP4X1*, members of the cytochrome P450 superfamily of enzymes, are involved in the metabolism of fatty acids and synthesis of cholesterol and steroids^48^. The gene *SLC5A9* (BTA3 97.96 Mb) is related to activating the glucose-sodium cotransporters (SGLTs) that may significantly affect the energy homeostasis of the animal by a reduction in glucose supply^49^. Such findings for HR support the hypothesis that genomic regions with harmful effects surrounding genes related to body energy aspects delayed the return to reproductive activity due to lower body energy reserves and metabolic status. On the other hand, the favorable effect of these genomic regions is related to maintaining the energy body homeostasis, whereby provides a link between nutritional status and the gatekeeper signals to heifer attain reproductive function, which provides the longevity of the reproductive life of the female.

On BTA22 (46.96–47.09 Mb) including the gene set (*ACTR8*, *CACNA1D, CACNA2D3, CHDH, IL17RB,* and *SELENOK*), related to calcium transport and immune response (https://www.genecards.org/) (Supplementary material—Table S6 and S8). The immunity-related gene IL17RB plays a crucial role in inflammation, and the immune response and regions surrounding this gene were identified as affecting the slopes of the reaction norm to the fertility traits in Danish Holstein cattle^50^. In addition, the IL17RB encodes a cytokine that binds the *IL-25* (*IL17E*), mediating the immune response in dairy cattle in post-partum metabolic distress^51^.

Specific genomic regions affecting HR were identified on BTA 1, 3, 5, 7, 8, 9, 17, 18, 22 and 29, suggesting different physiology mechanisms leading to reproductive functions and adaptation to stress (Supplementary material—Table S2 and S6). The gene *NLGN1* encodes a member of a family of neuronal cell surface proteins with a role in synaptic signal transmission and functions affecting the GABAergic synapses^52^. Thus, the effect of the *NLGN1* gene on reproductive performance occurs by acting on GABAergic processes, which control reproduction by action on gonadotropin-releasing (GnRH) secretion^53^. Genes affecting physiological changes in lipid and glucose blood levels can affect reproductive functions because glucose levels represent a key biological link between metabolic factors and the endocrine axis to maintain the reproductive functions affecting the oocyte development and quality^54^.

## Conclusion

A total of thirty potentially lethal haplotypes were identified, and candidate regions can be studied in confirmatory analyzes (molecular and statistical). The GWAS pointed out significant SNP markers that implies different physiology mechanisms leading to harmful effects on heifer rebreeding (146 SNPs), post-natal mortality (268), and stayability (482) in Nellore cattle. The functional analysis showed 26 genes enriched for 19 GO terms (biological processes). We observed that most GO terms found for biological processes, molecular function, and pathways were related to tissue development and the immune system. Tests to find lethal haplotype carriers could help breeders to implement selection actions to eliminate these haplotypes from the population or manage mattings to avoid lethal allele dispersion. In addition, future studies with fine-mapping approaches, region sequencing, and expression analyses to find the causal mutation in candidate regions may also contribute to the decrease in the frequency of lethal alleles.

## Supplementary Information

Supplementary Information 1.

Supplementary Tables.

## Data Availability

The data that support the findings of this study have belonged to commercial breeding programs, and restrictions are applied to the availability of these data, which were used under license for the current study, and so are not publicly available. However, data are available by contacting the corresponding authors upon reasonable request and with permission of Alliance Nellore database (www.gensys.com.br).
