# Supplementary material for: Identification of candidate lethal haplotypes and genomic association with post-natal mortality and reproductive traits in Nellore cattle
Source: Sci Rep. 2023 Jun 27;13:10399. doi: 10.1038/s41598-023-37586-z (PMC10300016; doi:10.1038/s41598-023-37586-z)
Supplement: Supplementary file 1 — Supplementary Information 1. [file 41598_2023_37586_MOESM1_ESM.docx]

**Identification of candidate lethal haplotypes and genomic association with post-natal mortality and reproductive traits in Nellore cattle**

Patrícia Iana Schmidt^a,*^, Lucio Flavio Macedo Mota^a^, Larissa Fernanda Simielli Fonseca^a^, Danielly Beraldo dos Santos Silva^a^, Gabriela Bonfá Frezarim^a^, Leonardo Machestropa Arikawa^a^, Daniel Jordan de Abreu Santos^a^, Ana Fabrícia Braga Magalhães^a^, John Bruce Cole^b^, Roberto Carvalheiro^a^, Henrique Nunes de Oliveira^a^, Daniel Jacob Null^b^, Paul VanRaden^b^, Li Ma^c^, Lucia Galvão de Albuquerque^a,d,*^

^a^Animal Science Department, School of Agricultural and Veterinary Sciences, São Paulo State University (Unesp), Via de acesso Paulo Donato Castellane s/n. Departamento de Zootecnia, Jaboticabal, SP CEP 14884-900, Brazil

^b^Henry A. Wallace Beltsville Agricultural Research Center, Animal Genomics and Improvement Laboratory, Agricultural Research Service, USDA, Beltsville, MD 20705-2350, USA

^c^Department of Animal and Avian Sciences, University of Maryland, College Park 20742, USA

^d^National Council for Scientific and Technological Development (CNPq), Brasília, Brazil

^*^Corresponding authors: [pati.iana@hotmail.com](mailto:pati.iana@hotmail.com) (P.I. Schmidt), [galvao.albuquerque@unesp.br](mailto:galvao.albuquerque@unesp.br) (L.G. de Albuquerque)

## Functional gene networks

The annotated genes, protein-coding and non-coding genes with known function in ARS-UCD1.2 *Bos taurus* genome, were submitted to a non-redundant biological functional gene network analysis, a separate analysis for each trait. Most GO terms found for biological processes, molecular function, and pathways were related to tissue development, immune system, and disease-related metabolic pathways (Supplementary material - Table S9 to S15).

Several genes are associated with immune response activation and defense cells terms as immune system development (GO:0002520), leukocyte activation involved in immune response (GO:0002366), leukocyte differentiation (GO:0002521), regulation of immune system process (GO:0002682), lymphocyte activation involved in immune response (GO:0002285), leukocyte differentiation (GO:0002521), adaptive immune response (GO:0002250) and regulation of immune effector process (GO:0002697). In addition, terms related to cell differentiation as regulation of T cell activation (GO:0050863) and T cell differentiation (GO:0030217), regulation of lymphocyte activation (GO: 0051249), T cell activation (GO: 0042110) were also found. Genes related to immunity and defense systems could be crucial for HR, PNM, and STAY traits once they can determine the individual's survival.

KEGG Pathways associated with the disease were also found as Malaria (GO:0005144), MicroRNAs in cancer (GO:0005206), Salmonella infection (GO:0005132), Tuberculosis (GO:0005152), Pathways in cancer (GO:05200), Thyroid cancer (GO:05216), Melanoma (GO:005218), Gastric cancer (GO:05226), Bladder cancer (GO:05219) and Type II diabetes mellitus (GO:0004930). In humans, there is an association between metabolic disease as diabetes, coronary heart disease, and cardiovascular disease, which increases the risk of mortality.

The common genes for the traits are mainly related to the immune system process (GO:0002376), production of molecular mediator of immune response (GO:0002440), leukocyte mediated immunity (GO: 0002443), natural killer cell activation (GO:0030101), regulation of biological process (GO:0050789) and regulation of metabolic process (GO:0019222). As exposed before, HR, PNM, and STAY are closely connected to GO terms and pathways related to organ development, diseases, and evolution. According to Wilkinson et al. ^1^, in humans, the multiple mortality rates for two, three, four, or more failed organ systems were 26%, 62%, and 88%, respectively. Marín-Garzón et al. ^2^, studying maternal effects of preweaning calf mortality in Nellore cattle, also found organ development, embryonic development, immune system, and disease related-terms and pathways.

The innate immune system responds quickly to various pathological challenges and may play an important role in pregnancy, being found in the placenta. This system also may help detect and control early signs of cancer and is known for killing virally infected cells ^3^. Therefore, cancer, infection, and viruses related terms, besides innate and adaptive terms and pathways, were much enriched in this study (Supplementary material - Table S10 an S14).

The adaptive immune system, represented by T cells and lymphocytes, is also active in response to specific recognition of cancer cells ^4^ and is associated with a wide variety of immune responses, such as viral infections, blood cancers, autoimmune diseases, stem cell transplantation, and pregnancy ^5^. Another important pathway enriched with genes in the three traits is the PPAR signaling pathway (GO:0003320). The PPAR metabolism played an essential role in cellular lipid circulation and oxidation and was related to productive traits in bovines as lipid content in *Longissimus thoracis* muscle tissue ^6,7^. Inflammation and lipid signaling are intertwined modulators of homeostasis and immunity. In addition, emerging studies indicate that many other lipid species regulate inflammatory responses positively and negatively ^8^.

On BTA22 (46.96 – 47.09 Mb) the genes *CACNA1D* and *CACNA2D3* are related to the voltage-dependent calcium (Ca­_v_) channel complex, mediating the influx of calcium ions, which is involved in calcium-dependent, processes such as muscle contraction, hormone, and neurotransmitter release and gene expression ^9,10^ (Supplementary material - Table S6, S7 and S8). Furthermore, these genes directly affect insulin secretory granules, contributing to maintaining *B*-cell mass and function ^9^. In addition, the gene *SELENOK* has been reported to regulate the redox equilibrium and modulate calcium flux, aiming to reduce the intracellular ROS (reactive oxygen species) levels and oxidative stress-induced ^11^. In this context, gene *SELENOK* affecting the ROS activity can indirectly affect reproduction due to ROS imbalance affecting multiple physiological processes from oocyte maturation to fertilization, embryo development, and pregnancy ^12^.

Considering a 100kb up and downstream from SNP markers, a total of 28 genes were shared among STAY and PNM only (Figure 2 B and Supplementary material - Table S4). From those, six genes showed a relation with HR and PNM. The gene *ABCD4* is a member of the superfamily of ATP-binding cassette (*ABC*) transporters with a key function of modulating the transfer of substrates from the cytosol toward the extracellular space ^13^. In addition, the *ABC* gene transporters family are also important directly and indirectly to key reproductive processes by transporting steroid hormones, cholesterol, and fat-soluble vitamins ^14^. In humans, Bloise et al. ^15^ observed that the *ABC* gene family plays an essential physiological and protective role during processes related to gametogenesis, embryogenesis, and embryo/fetal development. In addition, it is relevant in steroidogenesis modulation and acts as a gatekeeper against the harmful effects of immunological response.

The *CDH13* gene on BTA18 was common to HR and PNM traits (Supplementary material - Table S6 and S7) and encodes a member of the cadherin superfamily acting as a negative regulator of axon growth during neural differentiation, as well as protecting the cells from apoptosis due to oxidative. In addition, *CDH13* interacts with adiponectin in the smooth muscle and endothelial cells, which is involved in regulating glucose levels and fatty acid breakdown ^16^. Adiponectin is related to decreased insulin resistance and body weight due to increased lipid oxidation in muscle and liver, which increases the levels of reactive oxygen and nitrogen species (ROS and RNS) in the blood ^17^.

The *AFMID* gene is involved in the kynurenine pathway of tryptophan metabolism, which controls important physiological processes throughout the body, producing several biologically active metabolites, such as the redox cofactors oxidized NAD^+^(P^+^) and their reduced form *NADPH* and niacin level ^18^. Petrović et al. ^19^ observed that niacin with the NAD:NADP ratio represents an essential function on body homeostasis in dairy cattle in early lactation due to positive association with glucose, insulin, glucose to insulin ratio, triglycerides, and cholesterol.

The candidate genes (*ARRDC5, DPP9, FEM1A, MYDGF, PLIN3, TICAM1, TNFAIP8L1* and *UHRF1*) were found in regions of candidate haplotypes present on BTA7 (Supplementary material - Table S6, S7 and S8). The gene *TNFAIP8L2* is a member of tumor necrosis factor-alpha (TNFα), which exerts a critical biological action in immunity that maintains immune homeostasis, and depletion increases expression of genes associated with anti-proliferation, apoptosis, and lipid metabolism ^20^. The gene *FEM1A* plays an anti-inflammatory role by liganded EP4 subtype prostaglandin E2 receptor ^21^. Reduction in *FEM1A* function is associated with reductions in prostaglandin E2-mediated suppression of TNFα ^21^, and TNFα appears to be related to the adipocyte metabolism at numerous sites, including transcriptional regulation, glucose, and fatty acid metabolism, and hormone receptor signaling ^22^.

The gene *ALDH6A1* is involved in various biological processes in response to abiotic and biotic stress generated by perturbed endobiotic and/or xenobiotic metabolism, decreasing the harmful effect of oxidative stress ^23^. Oxidative stress is well-established as an underlying cause of infectious, metabolic diseases and reproductive disorders ^24^. The gene *BATF* is a bZIP transcription factor that plays an important role in regulating differentiation ^25^. In addition, it is involved in the immune regulation of dendritic cells (DC), T cells, and B cells with, directly and indirectly, action in regulating their adaptive immune responses against environmental and self-antigens ^26^. The gene *SYNGR2* regulates the membrane traffic, exocytosis, and storage and transport of *GLUT4* at the plasma membrane ^27^. This represents an important aspect of whole-body energy homeostasis due to the translocation of *GLUT4* to the plasma membrane stimulating insulin production. Finally, the *COQ6* gene is a key component of the mitochondrial electron transport chain and the major potent lipophilic antioxidant involved in protection from cellular damage by reactive oxygen species ^28^.

We found some genes and GO terms common for the studied traits (Supplementary material - Table S4). For PNM and STAY, six common genes were found: *LRTM1, TK1, JDP2, BATF, ABR*, and *GPR12*. The *LRTM1* is an integral membrane component that positively regulates synapse assembly. In humans, according to Samata et al. ^29^, the gene may be used as a tool for efficient and safe cell therapy for Parkinson's disease patients. In mice, according to Li et al. (2020) ^30^, *LRTM1*, an inhibitory regulator of the *FGFR* signaling pathway, negatively modulates the activation of *ERK* and promotes the differentiation of myoblast cells. In buffalos, this gene was associated with milk production traits ^31^.

The *TK1* gene, which encodes thymine kinase, a cytosolic and mitochondrial protein, was associated with a tumor in calf form bovine leucosis ^32^. Alpha-2-macroglobulin (A2M), common to HR, STAY, and PNM, is an ancient component of the innate immune system that has been highly conserved across species ^33^. This gene binds proteases, acting as a defense barrier against pathogens in plasma and tissues. Furthermore, there is evidence that A2M may be a biomarker for the early detection of ovarian cancer ^34^, since its plasma levels increase in the late stages of this type of cancer ^35^. In bovines, this gene was associated with mastitis-infected mammary ^36^.

Among the genes common for PNM and STAY, *SLC7A1* belongs to the arginine uptake and flow system across plasma membranes. Arginine is an amino acid precursor of nitric oxide, a free radical that plays a vital role in regulating placental angiogenesis and uteroplacental-fetal flow of nutrients and oxygen ^37^. Furthermore, this amino acid is related to embryonic/conceptus survival and growth ^38^. In sheep, the translational knockdown of *SLC7A1* resulted in morphologically and functionally delayed development of conceptuses due to arginine deficiency ^37^.

The *PLIN3* gene, common to HR and STAY, is an important regulator of triglyceride storage in the form of cytosolic lipid droplets, which are the main energy resource during oocyte development and in the preimplantation mammalian embryos ^39^. Also, this gene plays a crucial role in the immune system, as it promotes prostaglandin E2 synthesis, a key mediator of the inflammatory process ^40^.

*TICAM1* is a gene responsible for the production of Interferon-beta (IFN-β), a cytokine that has diverse effects on innate immune cells as well as non-immune cells ^41,42^. It was reported that IFN-β plays a role in anti-inflammatory and pro-inflammatory responses and regulates the activation and development of innate and adaptive immune effector cells ^42^. However, evidence suggests that its effect on reproductive events is unfavorable. For example, in a study conducted on humans, it was observed that high levels of IFN-β during pregnancy might be associated with an increased risk of fetal loss and low birth weight ^43^.

The gene *PLAAT5* is involved in peroxisome regulation and plasmalogen metabolism, which is associated with controlling lipogenesis and adipogenesis ^44,45^. The gene *SLC2A12* belongs to a family responsible for glucose transport by encoding the glucose transporters family (GLUTS) ^46^. The gene *TCF21* promotes the transcription of lipoprotein lipase (LPL) by directly binding to the E-box motif in the LPL promoter ^47^.

The gene *FGD3* is involved in the GTPase activity and activation of *JUN Kinase*, which controls cell apoptosis. In this context, the genes *JUN kinase* with *ERKs* and *MAPKs* are involved in redirecting energy harvesting for glycolysis by affecting the activity of key metabolic regulators ^48^.

References

1. Wilkinson, J. D. *et al.* Mortality associated with multiple organ system failure and sepsis in pediatric intensive care unit. *J Pediatr* **111**, 324–328 (1987).

2. Marín-Garzón, N. A. *et al.* Genome-wide scan reveals genomic regions and candidate genes underlying direct and maternal effects of preweaning calf mortality in Nellore cattle. *Genomics* **113**, 1386–1395 (2021).

3. Abel, A. M., Yang, C., Thakar, M. S. & Malarkannan, S. Natural Killer Cells: Development, Maturation, and Clinical Utilization. *Front Immunol* **9**, (2018).

4. Ribas, A. Adaptive immune resistance: How cancer protects from immune attack. *Cancer Discov* **5**, 915 (2015).

5. Sojka, D. K. *et al.* Tissue-resident natural killer (NK) cells are cell lineages distinct from thymic and conventional splenic NK cells. *Elife* **3**, (2014).

6. Berton, M. P. *et al.* Gene expression profile of intramuscular muscle in Nellore cattle with extreme values of fatty acid. *BMC Genomics* **17**, 1–16 (2016).

7. dos Santos Silva, D. B. *et al.* Prediction of hub genes associated with intramuscular fat content in Nelore cattle. *BMC Genomics* **20**, 1–12 (2019).

8. Glass, C. K. & Olefsky, J. M. Inflammation and lipid signaling in the etiology of insulin resistance. *Cell Metab* **15**, 635–645 (2012).

9. Catterall, W. A. Structure and Regulation of Voltage-Gated Ca 2+ Channels. *Annu Rev Cell Dev Biol* **16**, 521–555 (2000).

10. Yang, S.-N. & Berggren, P.-O. The Role of Voltage-Gated Calcium Channels in Pancreatic β-Cell Physiology and Pathophysiology. *Endocr Rev* **27**, 621–676 (2006).

11. Jia, S. Z. *et al.* Selenoprotein K deficiency-induced apoptosis: A role for calpain and the ERS pathway. *Redox Biol* **47**, (2021).

12. Agarwal, A., Gupta, S. & Sharma, R. K. Role of oxidative stress in female reproduction. *Reproductive Biology and Endocrinology* **3**, 28 (2005).

13. Tarling, E. J. & Edwards, P. A. ATP binding cassette transporter G1 (ABCG1) is an intracellular sterol transporter. *Proceedings of the National Academy of Sciences* **108**, 19719–19724 (2011).

14. Ikonen, E. Cellular cholesterol trafficking and compartmentalization. *Nat Rev Mol Cell Biol* **9**, 125–138 (2008).

15. Bloise, E. *et al.* ATP-binding cassette transporters in reproduction: a new frontier. *Hum Reprod Update* **22**, dmv049 (2015).

16. Jee, S. H. *et al.* Adiponectin concentrations: A genome-wide association study. *Am J Hum Genet* **87**, 545–552 (2010).

17. Maslov, L. N. *et al.* Is oxidative stress of adipocytes a cause or a consequence of the metabolic syndrome? *J Clin Transl Endocrinol* **15**, 1–5 (2019).

18. Badawy, A. A. B. Kynurenine Pathway of Tryptophan Metabolism: Regulatory and Functional Aspects. *International Journal of Tryptophan Research* **10**, 1–20 (2017).

19. Petrović, K. *et al.* Niacin Status Indicators and Their Relationship with Metabolic Parameters in Dairy Cows during Early Lactation. *Animals* **12**, 1–14 (2022).

20. Sethi, J. K. & Hotamisligil, G. S. The role of TNFα in adipocyte metabolism. *Semin Cell Dev Biol* **10**, 19–29 (1999).

21. Takayama, K., Sukhova, G. K., Chin, M. T. & Libby, P. A novel prostaglandin E receptor 4-associated protein participates in antiinflammatory signaling. *Circ Res* **98**, 499–504 (2006).

22. Higuchi, S. *et al.* EP 4 receptor-associated protein regulates gluconeogenesis in the liver and is associated with hyperglycemia in diabetic mice. *American Journal of Physiology-Endocrinology and Metabolism* **316**, E410–E417 (2019).

23. Singh, S. *et al.* Aldehyde dehydrogenases in cellular responses to oxidative/electrophilicstress. *Free Radic Biol Med* **56**, 89–101 (2013).

24. Folnožić, I. *et al.* Influence of Body Condition on Serum Metabolic Indicators of Lipid Mobilization and Oxidative Stress in Dairy Cows During the Transition Period. *Reproduction in Domestic Animals* **50**, 910–917 (2015).

25. Martin, L. J. & Nguyen, H. T. Basic Leucine Zipper Transcription Factors as Important Regulators of Leydig Cells’ Functions. *Int J Mol Sci* **23**, 12887 (2022).

26. Betz, B. C. *et al.* Batf coordinates multiple aspects of B and T cell function required for normal antibody responses. *Journal of Experimental Medicine* **207**, 933–942 (2010).

27. Kioumourtzoglou, D., Pryor, P. R., Gould, G. W. & Bryant, N. J. Alternative routes to the cell surface underpin insulin-regulated membrane trafficking of GLUT4. *J Cell Sci* **128**, 2423–2429 (2015).

28. González-García, P. *et al.* Animal Models of Coenzyme Q Deficiency: Mechanistic and Translational Learnings. (2021) doi:10.3390/antiox10110000.

29. Samata, B. *et al.* Purification of functional human ES and iPSC-derived midbrain dopaminergic progenitors using LRTM1. *Nature Communications 2016 7:1* **7**, 1–11 (2016).

30. Li, H. *et al.* LRTM1 promotes the differentiation of myoblast cells by negatively regulating the FGFR1 signaling pathway. *Exp Cell Res* **396**, 112237 (2020).

31. Deng, T. *et al.* Integrative analysis of transcriptome and GWAS data to identify the hub genes associated with milk yield trait in buffalo. *Front Genet* **10**, 36 (2019).

32. Tawfeeq, M. M. *et al.* Calf Form Bovine Leukosis with Lameness in a Holstein Heifer. *J. Vet. Med. Sci* **74**, 1225–1228 (2012).

33. Rehman, A. A., Ahsan, H. & Khan, F. H. Alpha-2-macroglobulin: A physiological guardian. *J Cell Physiol* **228**, 1665–1675 (2013).

34. Lim, W. & Song, G. JOURNAL OF CANCER PREVENTION Discovery of Prognostic Factors for Diagnosis and Treatment of Epithelial-Derived Ovarian Cancer from Laying Hens REVIEW. **18**, (2013).

35. Hawkridge, A. M. *et al.* Measuring the intra-individual variability of the plasma proteome in the chicken model of spontaneous ovarian adenocarcinoma. *Anal Bioanal Chem* **398**, 737–749 (2010).

36. Wang, X. *et al.* The exon 29 c.3535A>T in the alpha-2-macroglobulin gene causing aberrant splice variants is associated with mastitis in dairy cattle. *Immunogenetics* **64**, 807–816 (2012).

37. Wang, X. *et al.* Functional role of arginine during the peri-implantation period of pregnancy. I. Consequences of loss of function of arginine transporter SLC7A1 mRNA in ovine conceptus trophectoderm. *FASEB Journal* **28**, 2852–2863 (2014).

38. Wu, Y., Fan, H., Wang, Y., Zhang, L. & Gao, X. Genome-Wide Association Studies Using Haplotypes and Individual SNPs in Simmental Cattle. *PLoS One* **9**, (2014).

39. Ferguson, E. M. & Leese, H. J. A potential role for triglyceride as an energy source during bovine oocyte maturation and early embryo development. *Mol Reprod Dev* **73**, 1195–1201 (2006).

40. Itabe, H., Yamaguchi, T., Nimura, S. & Sasabe, N. Perilipins: a diversity of intracellular lipid droplet proteins. *Lipids in Health and Disease* vol. 16 Preprint at https://doi.org/10.1186/s12944-017-0473-y (2017).

41. Seya, T., Oshiumi, H., Sasai, M., Akazawa, T. & Matsumoto, M. TICAM-1 and TICAM-2: Toll-like receptor adapters that participate in induction of type 1 interferons. *International Journal of Biochemistry and Cell Biology* vol. 37 524–529 Preprint at https://doi.org/10.1016/j.biocel.2004.07.018 (2005).

42. McNab, F., Mayer-Barber, K., Sher, A., Wack, A. & O’Garra, A. Type I interferons in infectious disease. *Nature Reviews Immunology* vol. 15 87–103 Preprint at https://doi.org/10.1038/nri3787 (2015).

43. Boskovic, R., Wide, R., Wolpin, J., Bauer, D. & Koren, G. *The reproductive effects of beta interferon therapy in pregnancy A longitudinal cohort*. (2005).

44. Uyama, T., Tsuboi, K. & Ueda, N. An involvement of phospholipase A/acyltransferase family proteins in peroxisome regulation and plasmalogen metabolism. *FEBS Lett* **591**, 2745–2760 (2017).

45. Liu, J., Lu, W., Shi, B., Klein, S. & Su, X. Peroxisomal regulation of redox homeostasis and adipocyte metabolism. *Redox Biol* **24**, 101167 (2019).

46. Rogers, S. *et al.* Identification of a novel glucose transporter-like protein—GLUT-12. *American Journal of Physiology-Endocrinology and Metabolism* **282**, E733–E738 (2002).

47. Zhang, X. *et al.* A novel regulator of preadipocyte differentiation, transcription factor TCF21, functions partially through promoting LPL expression. *Front Physiol* **10**, (2019).

48. Weston, C. R. & Davis, R. J. The JNK signal transduction pathway. *Curr Opin Genet Dev* **12**, 14–21 (2002).
