# Supplementary material for: Identification of candidate lethal haplotypes and genomic association with post-natal mortality and reproductive traits in Nellore cattle
Source: Sci Rep. 2023 Jun 27;13:10399. doi: 10.1038/s41598-023-37586-z (PMC10300016; doi:10.1038/s41598-023-37586-z)
Supplement: Supplementary file 2 — Supplementary Tables. [file 41598_2023_37586_MOESM2_ESM.docx]

# Identification of candidate lethal haplotypes and genomic association with post-natal mortality and reproductive traits in Nellore cattle

Patrícia Iana Schmidt^a,*^, Lucio Flavio Macedo Mota^a^, Larissa Fernanda Simielli Fonseca^a^, Danielly Beraldo dos Santos Silva^a^, Gabriela Bonfá Frezarim^a^, Leonardo Machestropa Arikawa^a^, Daniel Jordan de Abreu Santos^a^, Ana Fabrícia Braga Magalhães^a^, John Bruce Cole^b^, Roberto Carvalheiro^a^, Henrique Nunes de Oliveira^a^, Daniel Jacob Null^b^, Paul VanRaden^b^, Li Ma^c^, Lucia Galvão de Albuquerque^a,d,*^

^a^Animal Science Department, School of Agricultural and Veterinary Sciences, São Paulo State University (Unesp), Via de acesso Paulo Donato Castellane s/n. Departamento de Zootecnia, Jaboticabal, SP CEP 14884-900, Brazil

^b^Henry A. Wallace Beltsville Agricultural Research Center, Animal Genomics and Improvement Laboratory, Agricultural Research Service, USDA, Beltsville, MD 20705-2350, USA

^c^Department of Animal and Avian Sciences, University of Maryland, College Park 20742, USA

^d^National Council for Scientific and Technological Development (CNPq), Brasília, Brazil

^*^Corresponding authors: [pati.iana@hotmail.com](mailto:pati.iana@hotmail.com) (P.I. Schmidt), [galvao.albuquerque@unesp.br](mailto:galvao.albuquerque@unesp.br) (L.G. de Albuquerque)

Table S1 – Chromosome (BTA), SNP and position in bp (a region of 100 kb down and upstream of each significant SNP) alleles of SNP and p-values for the SNP effects for post-natal mortality (PNM) of Nellore animals.

| BTA | SNP | ^1^Position | ^2^Start position | ^2^End position | ^3^A1 | ^3^A2 | BETA | SE | logp | p_value |
| --- | --- | --- | --- | --- | --- | --- | --- | --- | --- | --- |
| 1 | BovineHD0100025911 | 90720556 | 90620556 | 90820556 | A | B | -0.05964 | 0.00224 | 6.12385 | 7.52E-07 |
| 1 | BovineHD0100025921 | 90753390 | 90653390 | 90853390 | A | B | 0.06117 | 0.00228 | 6.21980 | 6.03E-07 |
| 2 | BovineHD0200008098 | 27715902 | 27615902 | 27815902 | A | B | 0.12336 | 0.00406 | 6.90569 | 1.24E-07 |
| 2 | BovineHD0200008259 | 28207961 | 28107961 | 28307961 | A | B | 0.10199 | 0.00357 | 6.20771 | 6.20E-07 |
| 2 | BovineHD0200008364 | 28439128 | 28339128 | 28539128 | B | A | 0.08901 | 0.00307 | 6.36287 | 4.34E-07 |
| 2 | BTB-01141260 | 28442555 | 28342555 | 28542555 | A | B | 0.08914 | 0.00307 | 6.37733 | 4.19E-07 |
| 2 | BovineHD0200008367 | 28451487 | 28351487 | 28551487 | B | A | 0.07264 | 0.00259 | 6.00479 | 9.89E-07 |
| 2 | BovineHD0200008371 | 28470505 | 28370505 | 28570505 | A | B | 0.07233 | 0.00258 | 6.00644 | 9.85E-07 |
| 2 | BovineHD0200008372 | 28471747 | 28371747 | 28571747 | A | B | 0.07233 | 0.00258 | 6.00644 | 9.85E-07 |
| 2 | BovineHD0200008373 | 28478055 | 28378055 | 28578055 | A | B | 0.07275 | 0.00258 | 6.05609 | 8.79E-07 |
| 2 | BovineHD0200008376 | 28484785 | 28384785 | 28584785 | B | A | 0.07322 | 0.00258 | 6.14795 | 7.11E-07 |
| 2 | BovineHD0200008552 | 29114918 | 29014918 | 29214918 | B | A | -0.08147 | 0.00276 | 6.54897 | 2.83E-07 |
| 2 | BovineHD0200008553 | 29115724 | 29015724 | 29215724 | A | B | -0.08110 | 0.00277 | 6.48296 | 3.29E-07 |
| 2 | BovineHD0200008554 | 29119788 | 29019788 | 29219788 | A | B | -0.08446 | 0.00274 | 7.08495 | 8.22E-08 |
| 2 | BTB-01987495 | 29121314 | 29021314 | 29221314 | B | A | -0.08446 | 0.00274 | 7.08495 | 8.22E-08 |
| 2 | BovineHD0200008900 | 30227987 | 30127987 | 30327987 | A | B | 0.07961 | 0.00241 | 8.00677 | 9.85E-09 |
| 2 | BovineHD0200009100 | 30768645 | 30668645 | 30868645 | B | A | 0.11667 | 0.00369 | 7.40620 | 3.92E-08 |
| 2 | BovineHD0200009125 | 30826160 | 30726160 | 30926160 | A | B | 0.11010 | 0.00393 | 6.01543 | 9.65E-07 |
| 2 | BovineHD0200009365 | 31507610 | 31407610 | 31607610 | A | B | -0.06943 | 0.00232 | 6.71703 | 1.92E-07 |
| 2 | BovineHD0200009473 | 31849497 | 31749497 | 31949497 | B | A | 0.07408 | 0.00264 | 6.02858 | 9.36E-07 |
| 2 | BovineHD0200009558 | 32230040 | 32130040 | 32330040 | B | A | 0.06851 | 0.00223 | 7.02015 | 9.55E-08 |
| 2 | BovineHD0200009618 | 32508293 | 32408293 | 32608293 | B | A | 0.07752 | 0.00238 | 7.79453 | 1.60E-08 |
| 2 | BovineHD0200009621 | 32511999 | 32411999 | 32611999 | A | B | 0.07752 | 0.00238 | 7.79453 | 1.60E-08 |
| 2 | BovineHD0200009622 | 32513160 | 32413160 | 32613160 | B | A | 0.07864 | 0.00230 | 8.47417 | 3.36E-09 |
| 2 | BovineHD0200009624 | 32541962 | 32441962 | 32641962 | A | B | -0.06784 | 0.00240 | 6.11084 | 7.75E-07 |
| 2 | BovineHD0200009628 | 32564150 | 32464150 | 32664150 | B | A | 0.07830 | 0.00236 | 8.06199 | 8.67E-09 |
| 2 | BovineHD0200009632 | 32586997 | 32486997 | 32686997 | A | B | 0.07608 | 0.00224 | 8.41946 | 3.81E-09 |
| 2 | BovineHD0200009637 | 32618197 | 32518197 | 32718197 | A | B | 0.10779 | 0.00331 | 7.79729 | 1.59E-08 |
| 2 | BovineHD0200009639 | 32629733 | 32529733 | 32729733 | B | A | 0.09694 | 0.00326 | 6.66627 | 2.16E-07 |
| 2 | BovineHD0200009640 | 32633136 | 32533136 | 32733136 | B | A | 0.10188 | 0.00327 | 7.23112 | 5.87E-08 |
| 2 | BovineHD0200009643 | 32642246 | 32542246 | 32742246 | A | B | 0.09581 | 0.00331 | 6.36128 | 4.35E-07 |
| 2 | BovineHD0200009648 | 32659369 | 32559369 | 32759369 | A | B | 0.10552 | 0.00329 | 7.59637 | 2.53E-08 |
| 3 | BovineHD0300023763 | 82491911 | 82391911 | 82591911 | A | B | 0.08070 | 0.00286 | 6.07559 | 8.40E-07 |
| 3 | BovineHD0300024086 | 83774261 | 83674261 | 83874261 | B | A | -0.07412 | 0.00232 | 7.51895 | 3.03E-08 |
| 3 | BovineHD0300024088 | 83777034 | 83677034 | 83877034 | B | A | -0.07310 | 0.00234 | 7.22879 | 5.90E-08 |
| 3 | BovineHD0300024090 | 83782532 | 83682532 | 83882532 | A | B | -0.07064 | 0.00230 | 7.04803 | 8.95E-08 |
| 3 | BovineHD0300024091 | 83784158 | 83684158 | 83884158 | A | B | -0.07188 | 0.00234 | 7.01676 | 9.62E-08 |
| 3 | BovineHD0300024092 | 83784970 | 83684970 | 83884970 | B | A | -0.07022 | 0.00230 | 6.95353 | 1.11E-07 |
| 3 | BovineHD0300024093 | 83786013 | 83686013 | 83886013 | A | B | -0.07034 | 0.00230 | 6.99246 | 1.02E-07 |
| 3 | BovineHD0300024094 | 83787374 | 83687374 | 83887374 | A | B | -0.07257 | 0.00235 | 7.11662 | 7.65E-08 |
| 3 | BovineHD0300024095 | 83788223 | 83688223 | 83888223 | B | A | -0.06936 | 0.00234 | 6.59248 | 2.56E-07 |
| 3 | BovineHD0300024096 | 83789003 | 83689003 | 83889003 | A | B | -0.07028 | 0.00235 | 6.72791 | 1.87E-07 |
| 3 | BovineHD0300024097 | 83790150 | 83690150 | 83890150 | B | A | -0.07028 | 0.00235 | 6.72791 | 1.87E-07 |
| 3 | BovineHD0300024098 | 83790992 | 83690992 | 83890992 | A | B | -0.07028 | 0.00235 | 6.72791 | 1.87E-07 |
| 3 | BovineHD0300024099 | 83791830 | 83691830 | 83891830 | A | B | 0.06694 | 0.00238 | 6.04278 | 9.06E-07 |
| 3 | BovineHD0300024106 | 83802212 | 83702212 | 83902212 | A | B | -0.08017 | 0.00235 | 8.42587 | 3.75E-09 |
| 3 | BovineHD0300024192 | 84138434 | 84038434 | 84238434 | B | A | -0.06832 | 0.00224 | 6.94321 | 1.14E-07 |
| 3 | BovineHD0300024214 | 84205383 | 84105383 | 84305383 | B | A | 0.06534 | 0.00233 | 6.03499 | 9.23E-07 |
| 3 | BovineHD0300024335 | 84651785 | 84551785 | 84751785 | A | B | -0.07117 | 0.00248 | 6.26857 | 5.39E-07 |
| 3 | ARS-BFGL-NGS-103524 | 84706206 | 84606206 | 84806206 | B | A | -0.07609 | 0.00270 | 6.08120 | 8.29E-07 |
| 3 | BovineHD0300024367 | 84709527 | 84609527 | 84809527 | A | B | -0.07587 | 0.00270 | 6.05025 | 8.91E-07 |
| 3 | BovineHD0300024368 | 84710542 | 84610542 | 84810542 | A | B | -0.07601 | 0.00270 | 6.06979 | 8.52E-07 |
| 3 | BovineHD0300024480 | 85132855 | 85032855 | 85232855 | A | B | 0.09392 | 0.00276 | 8.41102 | 3.88E-09 |
| 3 | BovineHD0300024481 | 85134816 | 85034816 | 85234816 | B | A | 0.09318 | 0.00276 | 8.28167 | 5.23E-09 |
| 3 | BovineHD0300024485 | 85142247 | 85042247 | 85242247 | A | B | 0.09298 | 0.00276 | 8.25018 | 5.62E-09 |
| 3 | BovineHD0300024491 | 85147583 | 85047583 | 85247583 | A | B | 0.09308 | 0.00276 | 8.26623 | 5.42E-09 |
| 3 | BTB-01389905 | 85192900 | 85092900 | 85292900 | A | B | 0.08359 | 0.00292 | 6.21416 | 6.11E-07 |
| 3 | BovineHD0300024505 | 85248054 | 85148054 | 85348054 | B | A | 0.08250 | 0.00283 | 6.41286 | 3.86E-07 |
| 3 | BovineHD0300024506 | 85249969 | 85149969 | 85349969 | A | B | 0.08250 | 0.00283 | 6.41286 | 3.86E-07 |
| 3 | BovineHD0300024590 | 85563203 | 85463203 | 85663203 | B | A | 0.06758 | 0.00241 | 6.01294 | 9.71E-07 |
| 3 | BovineHD0300024592 | 85565938 | 85465938 | 85665938 | A | B | -0.07106 | 0.00223 | 7.47753 | 3.33E-08 |
| 3 | Hapmap43465-BTA-111846 | 85755209 | 85655209 | 85855209 | A | B | 0.07479 | 0.00252 | 6.61992 | 2.40E-07 |
| 3 | BovineHD0300024640 | 85756884 | 85656884 | 85856884 | B | A | 0.06961 | 0.00247 | 6.06413 | 8.63E-07 |
| 3 | BovineHD0300024667 | 85809695 | 85709695 | 85909695 | B | A | 0.07577 | 0.00269 | 6.05924 | 8.72E-07 |
| 3 | BovineHD0300024669 | 85819068 | 85719068 | 85919068 | A | B | 0.09329 | 0.00308 | 6.86108 | 1.38E-07 |
| 3 | BovineHD0300025043 | 86841639 | 86741639 | 86941639 | A | B | 0.06278 | 0.00224 | 6.02327 | 9.48E-07 |
| 5 | BovineHD0500015915 | 55555845 | 55455845 | 55655845 | B | A | -0.10225 | 0.00225 | 6.30842 | 4.92E-07 |
| 5 | BovineHD0500028552 | 99424373 | 99324373 | 99524373 | B | A | 0.07171 | 0.00242 | 6.61964 | 2.40E-07 |
| 5 | BovineHD0500028553 | 99424952 | 99324952 | 99524952 | B | A | 0.07171 | 0.00242 | 6.61964 | 2.40E-07 |
| 5 | BovineHD0500028559 | 99438837 | 99338837 | 99538837 | B | A | 0.07317 | 0.00249 | 6.50525 | 3.12E-07 |
| 5 | BovineHD0500028560 | 99439661 | 99339661 | 99539661 | B | A | 0.07412 | 0.00249 | 6.67690 | 2.10E-07 |
| 5 | BovineHD0500028563 | 99446410 | 99346410 | 99546410 | B | A | 0.07326 | 0.00249 | 6.51965 | 3.02E-07 |
| 5 | BovineHD0500028564 | 99448139 | 99348139 | 99548139 | B | A | 0.07314 | 0.00249 | 6.49996 | 3.16E-07 |
| 5 | BovineHD0500028975 | 100803335 | 100703335 | 100903335 | B | A | 0.06538 | 0.00230 | 6.14911 | 7.09E-07 |
| 5 | BovineHD0500029073 | 101056230 | 100956230 | 101156230 | B | A | 0.13393 | 0.00396 | 8.34654 | 4.50E-09 |
| 9 | BovineHD0900011501 | 40802292 | 40702292 | 40902292 | B | A | 0.07092 | 0.00235 | 6.80130 | 1.58E-07 |
| 9 | ARS-BFGL-NGS-37643 | 41871249 | 41771249 | 41971249 | A | B | 0.10703 | 0.00356 | 6.77477 | 1.68E-07 |
| 9 | BovineHD0900011755 | 41875611 | 41775611 | 41975611 | B | A | 0.10696 | 0.00356 | 6.76108 | 1.73E-07 |
| 9 | BovineHD0900011756 | 41878308 | 41778308 | 41978308 | B | A | 0.10847 | 0.00384 | 6.07707 | 8.37E-07 |
| 9 | BovineHD0900011761 | 41895521 | 41795521 | 41995521 | B | A | 0.10468 | 0.00356 | 6.51135 | 3.08E-07 |
| 9 | BovineHD0900011764 | 41905812 | 41805812 | 42005812 | B | A | 0.13365 | 0.00420 | 7.48235 | 3.29E-08 |
| 9 | BovineHD0900011769 | 41924411 | 41824411 | 42024411 | A | B | 0.12338 | 0.00379 | 7.78400 | 1.64E-08 |
| 9 | BovineHD0900012075 | 43065391 | 42965391 | 43165391 | B | A | -0.04446 | 0.00237 | 6.97514 | 1.06E-07 |
| 9 | BovineHD0900012078 | 43067691 | 42967691 | 43167691 | A | B | -0.04497 | 0.00236 | 7.13607 | 7.31E-08 |
| 9 | BovineHD0900012087 | 43090013 | 42990013 | 43190013 | B | A | -0.04497 | 0.00236 | 7.13607 | 7.31E-08 |
| 9 | BovineHD0900012088 | 43091303 | 42991303 | 43191303 | B | A | -0.04589 | 0.00233 | 7.51709 | 3.04E-08 |
| 9 | BovineHD0900012089 | 43092753 | 42992753 | 43192753 | B | A | -0.04654 | 0.00234 | 7.67463 | 2.12E-08 |
| 9 | BovineHD0900012319 | 43827153 | 43727153 | 43927153 | A | B | -0.09080 | 0.00501 | 6.59257 | 2.56E-07 |
| 9 | BovineHD0900012329 | 43850334 | 43750334 | 43950334 | A | B | 0.07287 | 0.00257 | 6.13561 | 7.32E-07 |
| 9 | BovineHD0900012333 | 43862448 | 43762448 | 43962448 | B | A | 0.06624 | 0.00310 | 8.57489 | 2.66E-09 |
| 9 | BovineHD0900031445 | 43888647 | 43788647 | 43988647 | A | B | 0.07655 | 0.00265 | 6.32495 | 4.73E-07 |
| 9 | BovineHD0900012416 | 44172955 | 44072955 | 44272955 | A | B | -0.10310 | 0.00334 | 7.08729 | 8.18E-08 |
| 9 | BovineHD0900012417 | 44179205 | 44079205 | 44279205 | B | A | -0.04845 | 0.00223 | 8.80214 | 1.58E-09 |
| 9 | BovineHD0900012418 | 44185717 | 44085717 | 44285717 | A | B | -0.08230 | 0.00275 | 6.73496 | 1.84E-07 |
| 9 | BovineHD0900012419 | 44188847 | 44088847 | 44288847 | A | B | -0.10261 | 0.00334 | 7.04497 | 9.02E-08 |
| 9 | BovineHD0900012420 | 44190008 | 44090008 | 44290008 | B | A | -0.10240 | 0.00334 | 7.02459 | 9.45E-08 |
| 9 | BovineHD0900012423 | 44200920 | 44100920 | 44300920 | A | B | -0.10461 | 0.00340 | 7.06158 | 8.68E-08 |
| 9 | BovineHD0900012430 | 44237536 | 44137536 | 44337536 | A | B | -0.06394 | 0.00330 | 7.33972 | 4.57E-08 |
| 9 | BovineHD0900012431 | 44240652 | 44140652 | 44340652 | A | B | -0.05273 | 0.00302 | 6.21256 | 6.13E-07 |
| 9 | BovineHD0900012436 | 44266326 | 44166326 | 44366326 | A | B | -0.04424 | 0.00230 | 7.25934 | 5.50E-08 |
| 9 | BTA-83636-no-rs | 44411983 | 44311983 | 44511983 | A | B | 0.13936 | 0.00457 | 6.94848 | 1.13E-07 |
| 9 | BovineHD0900012505 | 44471447 | 44371447 | 44571447 | B | A | 0.11164 | 0.00370 | 6.81487 | 1.53E-07 |
| 9 | BovineHD0900012544 | 44592047 | 44492047 | 44692047 | B | A | -0.04364 | 0.00236 | 6.80515 | 1.57E-07 |
| 9 | BovineHD0900012559 | 44620153 | 44520153 | 44720153 | B | A | 0.12765 | 0.00428 | 6.69907 | 2.00E-07 |
| 9 | BovineHD0900012560 | 44623806 | 44523806 | 44723806 | A | B | 0.12765 | 0.00428 | 6.69907 | 2.00E-07 |
| 9 | BovineHD0900012564 | 44642478 | 44542478 | 44742478 | A | B | 0.12876 | 0.00427 | 6.80250 | 1.58E-07 |
| 9 | BovineHD0900012566 | 44648448 | 44548448 | 44748448 | B | A | 0.12217 | 0.00418 | 6.46029 | 3.47E-07 |
| 9 | BovineHD0900012568 | 44650515 | 44550515 | 44750515 | B | A | 0.12737 | 0.00454 | 6.02746 | 9.39E-07 |
| 9 | BovineHD0900012569 | 44653503 | 44553503 | 44753503 | B | A | 0.12744 | 0.00453 | 6.03577 | 9.21E-07 |
| 9 | BovineHD0900012570 | 44655917 | 44555917 | 44755917 | A | B | 0.12744 | 0.00453 | 6.03577 | 9.21E-07 |
| 9 | BovineHD0900012571 | 44657638 | 44557638 | 44757638 | A | B | 0.12744 | 0.00453 | 6.03577 | 9.21E-07 |
| 9 | BovineHD0900012574 | 44667939 | 44567939 | 44767939 | A | B | 0.12678 | 0.00453 | 6.00115 | 9.97E-07 |
| 9 | BovineHD0900012577 | 44681489 | 44581489 | 44781489 | A | B | 0.12697 | 0.00453 | 6.00677 | 9.85E-07 |
| 9 | BovineHD0900031450 | 44697983 | 44597983 | 44797983 | A | B | 0.05773 | 0.00265 | 8.89109 | 1.29E-09 |
| 9 | BovineHD0900012583 | 44708506 | 44608506 | 44808506 | B | A | 0.12691 | 0.00453 | 6.01174 | 9.73E-07 |
| 9 | BovineHD0900012584 | 44710611 | 44610611 | 44810611 | A | B | 0.12691 | 0.00453 | 6.01174 | 9.73E-07 |
| 9 | BovineHD0900012587 | 44726004 | 44626004 | 44826004 | A | B | 0.05785 | 0.00286 | 7.84813 | 1.42E-08 |
| 9 | BovineHD0900012590 | 44749461 | 44649461 | 44849461 | B | A | 0.05240 | 0.00262 | 7.72587 | 1.88E-08 |
| 9 | BovineHD0900012591 | 44755844 | 44655844 | 44855844 | A | B | 0.11069 | 0.00385 | 6.26893 | 5.38E-07 |
| 9 | BovineHD0900012593 | 44765124 | 44665124 | 44865124 | A | B | 0.11103 | 0.00385 | 6.30102 | 5.00E-07 |
| 9 | BovineHD0900012601 | 44785829 | 44685829 | 44885829 | A | B | 0.11247 | 0.00383 | 6.50560 | 3.12E-07 |
| 9 | BovineHD0900012606 | 44829036 | 44729036 | 44929036 | B | A | 0.11241 | 0.00386 | 6.42362 | 3.77E-07 |
| 9 | BovineHD0900012607 | 44836227 | 44736227 | 44936227 | A | B | 0.05320 | 0.00253 | 8.37332 | 4.23E-09 |
| 9 | BovineHD0900012599 | 44842415 | 44742415 | 44942415 | A | B | 0.12764 | 0.00453 | 6.07014 | 8.51E-07 |
| 9 | BovineHD0900012616 | 44881464 | 44781464 | 44981464 | B | A | 0.08472 | 0.00285 | 6.63559 | 2.31E-07 |
| 9 | BovineHD0900012619 | 44900439 | 44800439 | 45000439 | A | B | -0.05375 | 0.00286 | 6.97204 | 1.07E-07 |
| 9 | BovineHD0900012637 | 44965261 | 44865261 | 45065261 | A | B | -0.04281 | 0.00239 | 6.49485 | 3.20E-07 |
| 9 | BovineHD0900012643 | 44989866 | 44889866 | 45089866 | B | A | -0.04114 | 0.00239 | 6.10242 | 7.90E-07 |
| 9 | BovineHD0900012644 | 44994161 | 44894161 | 45094161 | A | B | -0.04114 | 0.00239 | 6.10242 | 7.90E-07 |
| 9 | BovineHD0900012650 | 45010018 | 44910018 | 45110018 | B | A | -0.06280 | 0.00319 | 7.52933 | 2.96E-08 |
| 9 | BovineHD0900013323 | 47760100 | 47660100 | 47860100 | B | A | -0.06855 | 0.00236 | 6.40027 | 3.98E-07 |
| 9 | BovineHD0900013709 | 49122134 | 49022134 | 49222134 | A | B | 0.11758 | 0.00395 | 6.66857 | 2.14E-07 |
| 10 | BovineHD1000023576 | 82406000 | 82306000 | 82506000 | B | A | 0.10741 | 0.00376 | 6.21597 | 6.08E-07 |
| 10 | BovineHD1000023609 | 82485409 | 82385409 | 82585409 | A | B | 0.09621 | 0.00333 | 6.32775 | 4.70E-07 |
| 10 | BovineHD1000023611 | 82492493 | 82392493 | 82592493 | A | B | 0.13678 | 0.00449 | 6.93286 | 1.17E-07 |
| 10 | BovineHD1000024063 | 83997833 | 83897833 | 84097833 | B | A | 0.07139 | 0.00234 | 6.95208 | 1.12E-07 |
| 10 | BovineHD1000024065 | 83999197 | 83899197 | 84099197 | A | B | 0.07419 | 0.00232 | 7.52922 | 2.96E-08 |
| 10 | BovineHD1000024066 | 84002112 | 83902112 | 84102112 | B | A | -0.07926 | 0.00226 | 8.88429 | 1.31E-09 |
| 10 | BovineHD1000024072 | 84013909 | 83913909 | 84113909 | A | B | 0.07408 | 0.00244 | 6.88023 | 1.32E-07 |
| 10 | BovineHD1000030799 | 84043808 | 83943808 | 84143808 | A | B | 0.08282 | 0.00272 | 6.91919 | 1.20E-07 |
| 10 | BovineHD1000024085 | 84066002 | 83966002 | 84166002 | A | B | -0.07711 | 0.00224 | 8.58291 | 2.61E-09 |
| 10 | BovineHD1000024093 | 84075982 | 83975982 | 84175982 | B | A | 0.07409 | 0.00228 | 7.76837 | 1.70E-08 |
| 10 | BovineHD1000024170 | 84257922 | 84157922 | 84357922 | B | A | -0.07353 | 0.00224 | 7.87094 | 1.35E-08 |
| 10 | BovineHD1000024171 | 84265590 | 84165590 | 84365590 | B | A | -0.07534 | 0.00227 | 8.03085 | 9.31E-09 |
| 10 | BovineHD1000031563 | 84280408 | 84180408 | 84380408 | B | A | -0.09692 | 0.00328 | 6.57342 | 2.67E-07 |
| 10 | BovineHD1000024177 | 84285612 | 84185612 | 84385612 | A | B | -0.08970 | 0.00294 | 6.93469 | 1.16E-07 |
| 10 | BovineHD1000024180 | 84297520 | 84197520 | 84397520 | A | B | -0.07737 | 0.00268 | 6.30278 | 4.98E-07 |
| 10 | BovineHD1000024465 | 85402576 | 85302576 | 85502576 | B | A | 0.11125 | 0.00349 | 7.49452 | 3.20E-08 |
| 10 | BovineHD1000024483 | 85513009 | 85413009 | 85613009 | A | B | -0.08644 | 0.00304 | 6.15065 | 7.07E-07 |
| 10 | BovineHD1000024484 | 85515577 | 85415577 | 85615577 | B | A | -0.08644 | 0.00304 | 6.15065 | 7.07E-07 |
| 10 | BovineHD1000024485 | 85522467 | 85422467 | 85622467 | B | A | -0.08742 | 0.00304 | 6.27015 | 5.37E-07 |
| 10 | BovineHD1000024488 | 85537455 | 85437455 | 85637455 | A | B | -0.10576 | 0.00308 | 8.54843 | 2.83E-09 |
| 10 | BovineHD1000024489 | 85541155 | 85441155 | 85641155 | B | A | -0.08644 | 0.00304 | 6.15065 | 7.07E-07 |
| 10 | BovineHD1000024490 | 85543925 | 85443925 | 85643925 | B | A | -0.08627 | 0.00304 | 6.12961 | 7.42E-07 |
| 10 | BovineHD1000024507 | 85604897 | 85504897 | 85704897 | B | A | -0.09525 | 0.00314 | 6.87890 | 1.32E-07 |
| 10 | BovineHD1000030812 | 86660675 | 86560675 | 86760675 | A | B | -0.08774 | 0.00289 | 6.89394 | 1.28E-07 |
| 10 | BovineHD1000025199 | 87525074 | 87425074 | 87625074 | B | A | 0.13454 | 0.00410 | 7.88577 | 1.30E-08 |
| 12 | BovineHD1200008852 | 30012519 | 29912519 | 30112519 | A | B | -0.09242 | 0.00292 | 7.40362 | 3.95E-08 |
| 12 | BovineHD1200008980 | 30478972 | 30378972 | 30578972 | B | A | 0.08401 | 0.00274 | 7.02144 | 9.52E-08 |
| 12 | BovineHD1200009089 | 30912280 | 30812280 | 31012280 | B | A | 0.07877 | 0.00226 | 8.81482 | 1.53E-09 |
| 12 | BovineHD1200027770 | 30974743 | 30874743 | 31074743 | A | B | -0.07521 | 0.00268 | 6.03596 | 9.21E-07 |
| 12 | BovineHD1200009121 | 31020417 | 30920417 | 31120417 | A | B | -0.07463 | 0.00226 | 8.00957 | 9.78E-09 |
| 18 | BovineHD1800003544 | 9926826 | 9826826 | 10026826 | A | B | 0.80130 | 0.00549 | 7.77 | 1.69778E-08 |
| 18 | BovineHD1800010927 | 36102127 | 36002127 | 36202127 | A | B | 0.07062 | 0.00244 | 6.36845 | 4.28E-07 |
| 19 | BovineHD1900002318 | 8258059 | 8158059 | 8358059 | B | A | 0.06366 | 0.00224 | 6.14396 | 7.18E-07 |
| 19 | BovineHD1900002458 | 8572531 | 8472531 | 8672531 | A | B | 0.09815 | 0.00323 | 6.89873 | 1.26E-07 |
| 19 | BovineHD4100013893 | 8607433 | 8507433 | 8707433 | A | B | 0.13824 | 0.00422 | 7.87331 | 1.34E-08 |
| 19 | BovineHD1900002479 | 8629692 | 8529692 | 8729692 | A | B | 0.13543 | 0.00410 | 7.98111 | 1.04E-08 |
| 19 | BovineHD1900002543 | 8817259 | 8717259 | 8917259 | B | A | 0.15321 | 0.00496 | 7.09831 | 7.97E-08 |
| 19 | BovineHD1900003138 | 11549243 | 11449243 | 11649243 | B | A | -0.08937 | 0.00303 | 6.54697 | 2.84E-07 |
| 19 | BovineHD1900003579 | 13221808 | 13121808 | 13321808 | B | A | 0.06712 | 0.00228 | 6.53248 | 2.93E-07 |
| 19 | BovineHD1900004003 | 14639993 | 14539993 | 14739993 | B | A | 0.06460 | 0.00223 | 6.37569 | 4.21E-07 |
| 19 | BovineHD1900004059 | 14890522 | 14790522 | 14990522 | A | B | 0.08759 | 0.00282 | 7.19054 | 6.45E-08 |
| 19 | BovineHD1900004072 | 14907049 | 14807049 | 15007049 | B | A | 0.07826 | 0.00257 | 6.92091 | 1.20E-07 |
| 19 | BovineHD1900004104 | 14959229 | 14859229 | 15059229 | B | A | 0.09402 | 0.00282 | 8.09567 | 8.02E-09 |
| 19 | BovineHD1900004116 | 14976930 | 14876930 | 15076930 | A | B | 0.06679 | 0.00235 | 6.15360 | 7.02E-07 |
| 19 | BovineHD1900004118 | 14979635 | 14879635 | 15079635 | B | A | 0.11390 | 0.00401 | 6.15561 | 6.99E-07 |
| 19 | BovineHD1900004137 | 15014172 | 14914172 | 15114172 | A | B | 0.08883 | 0.00291 | 6.97205 | 1.07E-07 |
| 19 | BovineHD1900004149 | 15046211 | 14946211 | 15146211 | A | B | 0.09174 | 0.00292 | 7.33343 | 4.64E-08 |
| 19 | BovineHD1900004151 | 15051285 | 14951285 | 15151285 | B | A | -0.06633 | 0.00228 | 6.39379 | 4.04E-07 |
| 19 | BovineHD1900004152 | 15053973 | 14953973 | 15153973 | A | B | 0.09197 | 0.00292 | 7.36409 | 4.32E-08 |
| 19 | BovineHD1900004153 | 15055167 | 14955167 | 15155167 | A | B | 0.07541 | 0.00253 | 6.67615 | 2.11E-07 |
| 19 | BovineHD1900004155 | 15060629 | 14960629 | 15160629 | B | A | -0.06899 | 0.00229 | 6.81341 | 1.54E-07 |
| 19 | BovineHD1900004161 | 15071219 | 14971219 | 15171219 | A | B | -0.06826 | 0.00229 | 6.68607 | 2.06E-07 |
| 19 | BovineHD1900004163 | 15073800 | 14973800 | 15173800 | A | B | -0.06825 | 0.00229 | 6.68372 | 2.07E-07 |
| 19 | BovineHD1900004166 | 15081891 | 14981891 | 15181891 | A | B | 0.09648 | 0.00292 | 7.97617 | 1.06E-08 |
| 19 | BovineHD1900004189 | 15126694 | 15026694 | 15226694 | A | B | 0.09416 | 0.00286 | 7.93996 | 1.15E-08 |
| 19 | BovineHD1900004191 | 15146986 | 15046986 | 15246986 | A | B | 0.07520 | 0.00263 | 6.19780 | 6.34E-07 |
| 19 | Hapmap38895-BTA-46550 | 15158520 | 15058520 | 15258520 | B | A | 0.07543 | 0.00263 | 6.23247 | 5.86E-07 |
| 19 | BovineHD1900004237 | 15339526 | 15239526 | 15439526 | A | B | 0.08988 | 0.00312 | 6.29880 | 5.03E-07 |
| 19 | UA-IFASA-6239 | 15569005 | 15469005 | 15669005 | A | B | 0.07304 | 0.00242 | 6.80914 | 1.55E-07 |
| 19 | BovineHD1900004327 | 15579056 | 15479056 | 15679056 | B | A | -0.06579 | 0.00223 | 6.56440 | 2.73E-07 |
| 19 | BovineHD1900004331 | 15586107 | 15486107 | 15686107 | B | A | -0.07459 | 0.00226 | 7.99818 | 1.00E-08 |
| 19 | BovineHD1900004332 | 15587128 | 15487128 | 15687128 | B | A | -0.07366 | 0.00225 | 7.85340 | 1.40E-08 |
| 19 | BovineHD1900004335 | 15598591 | 15498591 | 15698591 | B | A | 0.10660 | 0.00357 | 6.68553 | 2.06E-07 |
| 19 | BovineHD1900004336 | 15601722 | 15501722 | 15701722 | B | A | -0.07467 | 0.00247 | 6.83368 | 1.47E-07 |
| 19 | BovineHD1900004337 | 15602696 | 15502696 | 15702696 | B | A | -0.07482 | 0.00247 | 6.85098 | 1.41E-07 |
| 19 | BovineHD1900004352 | 15624039 | 15524039 | 15724039 | A | B | 0.11593 | 0.00405 | 6.22451 | 5.96E-07 |
| 19 | BovineHD1900004361 | 15639141 | 15539141 | 15739141 | A | B | 0.06662 | 0.00226 | 6.53717 | 2.90E-07 |
| 19 | BovineHD1900004362 | 15640028 | 15540028 | 15740028 | B | A | 0.06575 | 0.00227 | 6.37060 | 4.26E-07 |
| 19 | BovineHD1900004480 | 15941554 | 15841554 | 16041554 | B | A | -0.06740 | 0.00224 | 6.78899 | 1.63E-07 |
| 19 | BovineHD1900004484 | 15953240 | 15853240 | 16053240 | B | A | 0.07391 | 0.00226 | 7.85326 | 1.40E-08 |
| 19 | BovineHD1900004497 | 15997041 | 15897041 | 16097041 | A | B | 0.07790 | 0.00224 | 8.71674 | 1.92E-09 |
| 19 | BovineHD1900004534 | 16105231 | 16005231 | 16205231 | A | B | 0.11067 | 0.00380 | 6.40698 | 3.92E-07 |
| 19 | BovineHD1900004536 | 16114522 | 16014522 | 16214522 | A | B | 0.11089 | 0.00380 | 6.42738 | 3.74E-07 |
| 19 | BovineHD1900004539 | 16118926 | 16018926 | 16218926 | B | A | 0.11089 | 0.00380 | 6.42738 | 3.74E-07 |
| 19 | BovineHD1900004540 | 16120516 | 16020516 | 16220516 | A | B | 0.11089 | 0.00380 | 6.42738 | 3.74E-07 |
| 19 | BovineHD1900004541 | 16121717 | 16021717 | 16221717 | B | A | 0.11046 | 0.00380 | 6.40429 | 3.94E-07 |
| 19 | Hapmap53903-rs29027471 | 16127706 | 16027706 | 16227706 | B | A | 0.11089 | 0.00380 | 6.42738 | 3.74E-07 |
| 19 | BovineHD1900004587 | 16230579 | 16130579 | 16330579 | A | B | 0.11511 | 0.00398 | 6.34526 | 4.52E-07 |
| 19 | BovineHD1900004588 | 16231340 | 16131340 | 16331340 | B | A | 0.11315 | 0.00393 | 6.28263 | 5.22E-07 |
| 19 | BovineHD1900004591 | 16234128 | 16134128 | 16334128 | A | B | 0.11449 | 0.00398 | 6.28849 | 5.15E-07 |
| 19 | BovineHD1900004597 | 16251659 | 16151659 | 16351659 | B | A | 0.11000 | 0.00391 | 6.05439 | 8.82E-07 |
| 19 | BovineHD1900004600 | 16254778 | 16154778 | 16354778 | B | A | 0.11012 | 0.00392 | 6.02046 | 9.54E-07 |
| 19 | BovineHD1900004610 | 16282057 | 16182057 | 16382057 | B | A | 0.11889 | 0.00398 | 6.71024 | 1.95E-07 |
| 19 | BovineHD1900004630 | 16348801 | 16248801 | 16448801 | A | B | -0.07863 | 0.00266 | 6.57151 | 2.68E-07 |
| 19 | ARS-BFGL-NGS-17597 | 16698614 | 16598614 | 16798614 | B | A | -0.07116 | 0.00240 | 6.61087 | 2.45E-07 |
| 19 | BovineHD1900004756 | 16699408 | 16599408 | 16799408 | A | B | -0.07081 | 0.00240 | 6.55863 | 2.76E-07 |
| 19 | BovineHD1900005059 | 17458651 | 17358651 | 17558651 | B | A | -0.06964 | 0.00241 | 6.34376 | 4.53E-07 |
| 19 | BovineHD1900005083 | 17492552 | 17392552 | 17592552 | A | B | -0.07211 | 0.00244 | 6.56005 | 2.75E-07 |
| 19 | BovineHD1900005085 | 17494292 | 17394292 | 17594292 | B | A | -0.07195 | 0.00244 | 6.53632 | 2.91E-07 |
| 19 | BovineHD1900005098 | 17523263 | 17423263 | 17623263 | A | B | 0.07737 | 0.00235 | 7.97015 | 1.07E-08 |
| 19 | BovineHD1900005111 | 17540346 | 17440346 | 17640346 | B | A | 0.06736 | 0.00231 | 6.45009 | 3.55E-07 |
| 19 | BovineHD1900005115 | 17552464 | 17452464 | 17652464 | B | A | -0.07726 | 0.00258 | 6.72958 | 1.86E-07 |
| 19 | BovineHD1900005307 | 18166490 | 18066490 | 18266490 | B | A | 0.10878 | 0.00359 | 6.86144 | 1.38E-07 |
| 19 | BovineHD1900005376 | 18468875 | 18368875 | 18568875 | B | A | 0.09377 | 0.00315 | 6.64757 | 2.25E-07 |
| 19 | BovineHD1900005377 | 18469645 | 18369645 | 18569645 | A | B | 0.09377 | 0.00315 | 6.64757 | 2.25E-07 |
| 19 | BovineHD1900005378 | 18470382 | 18370382 | 18570382 | B | A | 0.09377 | 0.00315 | 6.64757 | 2.25E-07 |
| 19 | BovineHD1900005379 | 18471534 | 18371534 | 18571534 | A | B | 0.09377 | 0.00315 | 6.64757 | 2.25E-07 |
| 19 | BovineHD1900005380 | 18476066 | 18376066 | 18576066 | A | B | 0.09263 | 0.00316 | 6.48120 | 3.30E-07 |
| 19 | BovineHD1900005453 | 18729900 | 18629900 | 18829900 | B | A | 0.10844 | 0.00321 | 8.32128 | 4.77E-09 |
| 19 | BovineHD1900005455 | 18749019 | 18649019 | 18849019 | A | B | 0.10815 | 0.00318 | 8.42054 | 3.80E-09 |
| 19 | BovineHD1900005459 | 18785251 | 18685251 | 18885251 | B | A | 0.10657 | 0.00319 | 8.12760 | 7.45E-09 |
| 19 | BovineHD1900005460 | 18788958 | 18688958 | 18888958 | B | A | 0.10648 | 0.00319 | 8.12971 | 7.42E-09 |
| 19 | BovineHD1900005798 | 19674513 | 19574513 | 19774513 | A | B | 0.07130 | 0.00230 | 7.16842 | 6.79E-08 |
| 19 | BovineHD1900005895 | 20007548 | 19907548 | 20107548 | A | B | -0.07660 | 0.00257 | 6.65784 | 2.20E-07 |
| 19 | BovineHD1900006082 | 20549539 | 20449539 | 20649539 | A | B | -0.07351 | 0.00259 | 6.14685 | 7.13E-07 |
| 19 | BovineHD1900006239 | 21208547 | 21108547 | 21308547 | B | A | 0.10627 | 0.00361 | 6.52914 | 2.96E-07 |
| 19 | BovineHD1900006445 | 21997999 | 21897999 | 22097999 | B | A | 0.12331 | 0.00401 | 7.04668 | 8.98E-08 |
| 19 | BovineHD1900015079 | 53208094 | 53108094 | 53308094 | A | B | -0.08108 | 0.00287 | 6.10084 | 7.93E-07 |
| 19 | BovineHD1900015088 | 53229099 | 53129099 | 53329099 | A | B | -0.07365 | 0.00224 | 7.90120 | 1.26E-08 |
| 19 | ARS-BFGL-NGS-113058 | 53314493 | 53214493 | 53414493 | B | A | -0.06753 | 0.00225 | 6.77485 | 1.68E-07 |
| 19 | BovineHD1900015112 | 53339100 | 53239100 | 53439100 | B | A | -0.07001 | 0.00225 | 7.20352 | 6.26E-08 |
| 19 | BovineHD1900015116 | 53358257 | 53258257 | 53458257 | A | B | -0.10079 | 0.00321 | 7.31776 | 4.81E-08 |
| 19 | BovineHD1900015117 | 53360620 | 53260620 | 53460620 | A | B | -0.10079 | 0.00321 | 7.31776 | 4.81E-08 |
| 19 | BovineHD1900015132 | 53446892 | 53346892 | 53546892 | B | A | -0.08349 | 0.00286 | 6.44081 | 3.62E-07 |
| 19 | ARS-BFGL-NGS-89323 | 53715038 | 53615038 | 53815038 | A | B | 0.09364 | 0.00326 | 6.25205 | 5.60E-07 |
| 19 | BovineHD1900015225 | 53724564 | 53624564 | 53824564 | A | B | -0.06817 | 0.00229 | 6.68830 | 2.05E-07 |
| 19 | BovineHD1900015232 | 53745414 | 53645414 | 53845414 | A | B | 0.06248 | 0.00222 | 6.03060 | 9.32E-07 |
| 19 | BovineHD1900015233 | 53748765 | 53648765 | 53848765 | A | B | 0.06248 | 0.00222 | 6.03060 | 9.32E-07 |
| 19 | BovineHD1900015249 | 53777486 | 53677486 | 53877486 | B | A | 0.07824 | 0.00253 | 7.13003 | 7.41E-08 |
| 19 | BovineHD1900015250 | 53778116 | 53678116 | 53878116 | A | B | 0.07688 | 0.00254 | 6.87371 | 1.34E-07 |
| 19 | BovineHD1900015275 | 53895316 | 53795316 | 53995316 | B | A | -0.08042 | 0.00239 | 8.27375 | 5.32E-09 |
| 19 | Hapmap49620-BTA-45922 | 54137367 | 54037367 | 54237367 | A | B | 0.06797 | 0.00229 | 6.64328 | 2.27E-07 |
| 19 | BovineHD1900015493 | 54468738 | 54368738 | 54568738 | B | A | -0.10020 | 0.00325 | 7.08437 | 8.23E-08 |
| 19 | BovineHD1900015524 | 54526769 | 54426769 | 54626769 | A | B | 0.07909 | 0.00276 | 6.25097 | 5.61E-07 |
| 19 | BovineHD1900015539 | 54584349 | 54484349 | 54684349 | A | B | 0.06932 | 0.00241 | 6.29061 | 5.12E-07 |
| 19 | BovineHD1900015683 | 54917584 | 54817584 | 55017584 | B | A | -0.06990 | 0.00227 | 7.09069 | 8.12E-08 |
| 19 | BovineHD1900015795 | 55229429 | 55129429 | 55329429 | A | B | 0.07581 | 0.00265 | 6.20942 | 6.17E-07 |
| 19 | BovineHD1900015827 | 55380352 | 55280352 | 55480352 | B | A | -0.07242 | 0.00248 | 6.45858 | 3.48E-07 |
| 19 | ARS-BFGL-NGS-88285 | 55474861 | 55374861 | 55574861 | B | A | 0.11576 | 0.00413 | 6.00411 | 9.91E-07 |
| 24 | BovineHD2400005064 | 18969475 | 18869475 | 19069475 | A | B | 0.14041 | 0.00454 | 7.11909 | 7.60E-08 |
| 24 | BovineHD2400005070 | 18983546 | 18883546 | 19083546 | B | A | 0.15973 | 0.00464 | 8.60259 | 2.50E-09 |
| 24 | BovineHD2400005071 | 18984285 | 18884285 | 19084285 | B | A | 0.15973 | 0.00464 | 8.60259 | 2.50E-09 |
| 24 | BovineHD2400005072 | 18985979 | 18885979 | 19085979 | A | B | 0.15615 | 0.00463 | 8.30164 | 4.99E-09 |
| 24 | BovineHD2400005106 | 19052164 | 18952164 | 19152164 | B | A | 0.07308 | 0.00227 | 7.63263 | 2.33E-08 |
| 24 | BovineHD2400005109 | 19056185 | 18956185 | 19156185 | A | B | 0.07380 | 0.00258 | 6.20717 | 6.21E-07 |
| 24 | BovineHD2400005132 | 19116307 | 19016307 | 19216307 | A | B | -0.06329 | 0.00225 | 6.06454 | 8.62E-07 |

^1^Positions of SNPs based on ARS-UCD1.2 Bos taurus genome assembly

^2^Start and end position for region of 100 kb down and upstream of each significant SNP

^3^Alleles of significant SNPs

Table S2 – Chromosome (BTA), SNP and position in bp (a region of 100 kb down and upstream of each significant SNP) alleles of SNP and p-values for the SNP effects for heifer rebreeding (HR) of Nellore females.

| BTA | SNP | ^1^Position | ^2^Start position | ^2^End position | ^3^A1 | ^3^A2 | BETA | SE | ${-log}_{10}(p-value)$ | p_value |
| --- | --- | --- | --- | --- | --- | --- | --- | --- | --- | --- |
| 1 | BovineHD0100006145 | 21253257 | 21153257 | 21353257 | B | A | 0.1552 | 0.0024 | 6.1573 | 6.96E-07 |
| 1 | UA-IFASA-1500 | 21277546 | 21177546 | 21377546 | A | B | 0.1697 | 0.0025 | 7.4498 | 3.55E-08 |
| 1 | BovineHD4100000122 | 21315127 | 21215127 | 21415127 | B | A | 0.1277 | 0.0023 | 6.3600 | 4.36E-07 |
| 1 | BovineHD0100006187 | 21323815 | 21223815 | 21423815 | B | A | 0.1242 | 0.0022 | 6.4184 | 3.82E-07 |
| 1 | BovineHD0100006188 | 21324921 | 21224921 | 21424921 | A | B | 0.1245 | 0.0022 | 6.4585 | 3.48E-07 |
| 1 | BovineHD0100026566 | 93151332 | 93051332 | 93251332 | A | B | 0.1672 | 0.0022 | 7.0745 | 8.42E-08 |
| 1 | BovineHD0100026567 | 93152835 | 93052835 | 93252835 | B | A | 0.1672 | 0.0022 | 7.0745 | 8.42E-08 |
| 3 | BovineHD0300024063 | 83680525 | 83580525 | 83780525 | A | B | -0.0828 | 0.0012 | 6.1167 | 7.64E-07 |
| 3 | BovineHD0300024337 | 84657325 | 84557325 | 84757325 | A | B | 0.1846 | 0.0027 | 7.4048 | 3.94E-08 |
| 3 | BovineHD0300024339 | 84659661 | 84559661 | 84759661 | A | B | 0.1849 | 0.0028 | 7.2058 | 6.23E-08 |
| 3 | BovineHD0300024340 | 84662228 | 84562228 | 84762228 | A | B | 0.1827 | 0.0026 | 8.6796 | 2.09E-09 |
| 3 | BovineHD0300024350 | 84681765 | 84581765 | 84781765 | A | B | 0.1678 | 0.0030 | 6.5719 | 2.68E-07 |
| 3 | BovineHD0300024375 | 84732770 | 84632770 | 84832770 | B | A | 0.1458 | 0.0019 | 10.8129 | 1.54E-11 |
| 3 | BovineHD0300024391 | 84792282 | 84692282 | 84892282 | A | B | -0.0831 | 0.0012 | 6.9673 | 1.08E-07 |
| 3 | BovineHD4100002278 | 84806170 | 84706170 | 84906170 | B | A | -0.1121 | 0.0015 | 9.3425 | 4.54E-10 |
| 3 | BovineHD0300027981 | 97002410 | 96902410 | 97102410 | B | A | -0.1307 | 0.0022 | 7.4085 | 3.90E-08 |
| 3 | BovineHD0300027982 | 97006745 | 96906745 | 97106745 | A | B | -0.1338 | 0.0024 | 6.0274 | 9.39E-07 |
| 3 | BovineHD0300027986 | 97017532 | 96917532 | 97117532 | B | A | 0.1479 | 0.0020 | 9.8987 | 1.26E-10 |
| 3 | BovineHD0300027989 | 97021182 | 96921182 | 97121182 | A | B | 0.1491 | 0.0020 | 10.1976 | 6.34E-11 |
| 3 | BTB-00147905 | 97023337 | 96923337 | 97123337 | A | B | 0.1491 | 0.0020 | 10.1976 | 6.34E-11 |
| 3 | BTB-01919893 | 97070925 | 96970925 | 97170925 | B | A | 0.1716 | 0.0021 | 7.1705 | 6.75E-08 |
| 3 | BovineHD0300028017 | 97127451 | 97027451 | 97227451 | A | B | 0.2417 | 0.0026 | 12.6196 | 2.40E-13 |
| 3 | BovineHD0300028020 | 97132012 | 97032012 | 97232012 | B | A | 0.2417 | 0.0026 | 12.6196 | 2.40E-13 |
| 3 | BovineHD0300028022 | 97134694 | 97034694 | 97234694 | B | A | 0.2417 | 0.0026 | 12.6196 | 2.40E-13 |
| 3 | BovineHD0300028025 | 97144061 | 97044061 | 97244061 | A | B | 0.1727 | 0.0021 | 7.3596 | 4.37E-08 |
| 3 | BovineHD0300028033 | 97182222 | 97082222 | 97282222 | A | B | -0.3190 | 0.0029 | 9.6100 | 2.45E-10 |
| 3 | BovineHD0300028098 | 97379888 | 97279888 | 97479888 | B | A | -0.1520 | 0.0027 | 6.4436 | 3.60E-07 |
| 3 | BovineHD0300028105 | 97388363 | 97288363 | 97488363 | A | B | -0.1518 | 0.0027 | 6.4298 | 3.72E-07 |
| 3 | BovineHD0300028109 | 97392796 | 97292796 | 97492796 | A | B | -0.1518 | 0.0027 | 6.4298 | 3.72E-07 |
| 3 | BovineHD0300028185 | 97568452 | 97468452 | 97668452 | A | B | 0.1031 | 0.0018 | 6.9361 | 1.16E-07 |
| 3 | BovineHD0300028225 | 97695578 | 97595578 | 97795578 | B | A | -0.0875 | 0.0012 | 7.2109 | 6.15E-08 |
| 3 | BovineHD0300028260 | 97778126 | 97678126 | 97878126 | B | A | -0.0907 | 0.0016 | 6.0450 | 9.02E-07 |
| 3 | BovineHD0300028261 | 97780594 | 97680594 | 97880594 | B | A | -0.0907 | 0.0016 | 6.0450 | 9.02E-07 |
| 3 | BovineHD0300028262 | 97781265 | 97681265 | 97881265 | B | A | -0.0907 | 0.0016 | 6.0450 | 9.02E-07 |
| 3 | BovineHD0300028264 | 97784393 | 97684393 | 97884393 | A | B | -0.0920 | 0.0017 | 6.2539 | 5.57E-07 |
| 3 | BovineHD0300028268 | 97802755 | 97702755 | 97902755 | A | B | -0.0922 | 0.0017 | 6.2873 | 5.16E-07 |
| 3 | BTB-00152030 | 97818135 | 97718135 | 97918135 | B | A | -0.0917 | 0.0016 | 6.2980 | 5.03E-07 |
| 3 | BovineHD0300028273 | 97827408 | 97727408 | 97927408 | A | B | -0.0918 | 0.0016 | 6.3132 | 4.86E-07 |
| 3 | BovineHD0300028292 | 97856362 | 97756362 | 97956362 | B | A | -0.0887 | 0.0013 | 7.1276 | 7.45E-08 |
| 3 | Hapmap50068-BTA-69023 | 97857026 | 97757026 | 97957026 | B | A | -0.0887 | 0.0013 | 7.1276 | 7.45E-08 |
| 3 | BovineHD0300028293 | 97860225 | 97760225 | 97960225 | A | B | -0.0803 | 0.0015 | 6.0043 | 9.90E-07 |
| 3 | BovineHD0300028304 | 97879072 | 97779072 | 97979072 | A | B | -0.0920 | 0.0016 | 6.2989 | 5.03E-07 |
| 3 | BovineHD0300028315 | 97916922 | 97816922 | 98016922 | A | B | -0.0931 | 0.0016 | 6.6962 | 2.01E-07 |
| 3 | BovineHD0300028316 | 97917876 | 97817876 | 98017876 | A | B | -0.0930 | 0.0016 | 6.6727 | 2.12E-07 |
| 3 | BovineHD0300028317 | 97918694 | 97818694 | 98018694 | B | A | -0.0935 | 0.0016 | 6.7750 | 1.68E-07 |
| 3 | BovineHD0300028318 | 97919315 | 97819315 | 98019315 | A | B | -0.0932 | 0.0016 | 6.7162 | 1.92E-07 |
| 3 | BovineHD0300028320 | 97923325 | 97823325 | 98023325 | A | B | -0.0932 | 0.0016 | 6.7162 | 1.92E-07 |
| 3 | BovineHD0300028324 | 97929484 | 97829484 | 98029484 | B | A | -0.0933 | 0.0016 | 6.7236 | 1.89E-07 |
| 3 | BovineHD0300028325 | 97930159 | 97830159 | 98030159 | A | B | -0.0933 | 0.0016 | 6.7236 | 1.89E-07 |
| 3 | BovineHD0300028326 | 97933311 | 97833311 | 98033311 | A | B | -0.0933 | 0.0016 | 6.7192 | 1.91E-07 |
| 3 | BovineHD0300028349 | 98012275 | 97912275 | 98112275 | B | A | -0.6168 | 0.0040 | 8.6698 | 2.14E-09 |
| 3 | BovineHD0300028358 | 98057144 | 97957144 | 98157144 | B | A | 0.2405 | 0.0028 | 9.3664 | 4.30E-10 |
| 3 | BovineHD0300028369 | 98100979 | 98000979 | 98200979 | B | A | -0.7865 | 0.0045 | 7.2017 | 6.29E-08 |
| 3 | BovineHD0300028373 | 98119475 | 98019475 | 98219475 | B | A | -0.0994 | 0.0014 | 6.4687 | 3.40E-07 |
| 3 | BovineHD0300028381 | 98146410 | 98046410 | 98246410 | A | B | -0.7467 | 0.0044 | 11.4589 | 3.48E-12 |
| 3 | BovineHD0300028385 | 98166506 | 98066506 | 98266506 | A | B | -0.7454 | 0.0045 | 11.3477 | 4.49E-12 |
| 3 | BovineHD0300028390 | 98187368 | 98087368 | 98287368 | A | B | -0.7442 | 0.0045 | 11.3022 | 4.99E-12 |
| 3 | BovineHD0300028395 | 98200605 | 98100605 | 98300605 | A | B | -0.7454 | 0.0045 | 11.3477 | 4.49E-12 |
| 3 | BovineHD0300028396 | 98205419 | 98105419 | 98305419 | A | B | 0.1558 | 0.0023 | 7.8619 | 1.37E-08 |
| 3 | BovineHD0300028405 | 98230941 | 98130941 | 98330941 | B | A | 0.2081 | 0.0029 | 9.2914 | 5.11E-10 |
| 3 | BovineHD0300028406 | 98232293 | 98132293 | 98332293 | A | B | 0.1460 | 0.0023 | 6.5415 | 2.87E-07 |
| 3 | BovineHD0300028407 | 98234237 | 98134237 | 98334237 | B | A | 0.1561 | 0.0023 | 7.9202 | 1.20E-08 |
| 3 | BovineHD0300028408 | 98235934 | 98135934 | 98335934 | A | B | 0.2081 | 0.0029 | 9.2914 | 5.11E-10 |
| 3 | BovineHD0300028409 | 98238757 | 98138757 | 98338757 | A | B | 0.2081 | 0.0029 | 9.2914 | 5.11E-10 |
| 3 | BovineHD0300028412 | 98244243 | 98144243 | 98344243 | A | B | 0.2081 | 0.0029 | 9.2914 | 5.11E-10 |
| 3 | BovineHD0300028413 | 98245263 | 98145263 | 98345263 | A | B | 0.2081 | 0.0029 | 9.2914 | 5.11E-10 |
| 3 | BovineHD0300028432 | 98303693 | 98203693 | 98403693 | A | B | 0.1941 | 0.0028 | 8.5639 | 2.73E-09 |
| 3 | BovineHD0300028667 | 99178761 | 99078761 | 99278761 | A | B | 0.1284 | 0.0019 | 7.6107 | 2.45E-08 |
| 3 | BovineHD0300028680 | 99260362 | 99160362 | 99360362 | B | A | 0.1272 | 0.0019 | 7.5501 | 2.82E-08 |
| 3 | BovineHD0300028682 | 99270414 | 99170414 | 99370414 | A | B | 0.1220 | 0.0019 | 6.6091 | 2.46E-07 |
| 3 | ARS-BFGL-NGS-111078 | 99342353 | 99242353 | 99442353 | A | B | 0.1416 | 0.0022 | 6.6921 | 2.03E-07 |
| 5 | BovineHD0500028937 | 100693400 | 100593400 | 100793400 | A | B | -0.1384 | 0.0022 | 6.2493 | 5.63E-07 |
| 5 | BovineHD0500028949 | 100726195 | 100626195 | 100826195 | B | A | -0.0736 | 0.0013 | 6.4833 | 3.29E-07 |
| 5 | BovineHD0500028950 | 100727070 | 100627070 | 100827070 | B | A | -0.0736 | 0.0013 | 6.4833 | 3.29E-07 |
| 5 | BovineHD0500028951 | 100728733 | 100628733 | 100828733 | A | B | -0.0721 | 0.0013 | 6.0391 | 9.14E-07 |
| 5 | BovineHD0500035515 | 100887486 | 100787486 | 100987486 | B | A | -0.0823 | 0.0012 | 7.5706 | 2.69E-08 |
| 5 | BovineHD0500029017 | 100912450 | 100812450 | 101012450 | A | B | -0.0834 | 0.0012 | 6.7034 | 1.98E-07 |
| 5 | BovineHD0500029022 | 100930308 | 100830308 | 101030308 | A | B | -0.1444 | 0.0025 | 7.1155 | 7.67E-08 |
| 5 | BovineHD0500029026 | 100946084 | 100846084 | 101046084 | B | A | -0.0770 | 0.0013 | 7.2902 | 5.13E-08 |
| 7 | BovineHD0700005726 | 19325172 | 19225172 | 19425172 | B | A | -0.0933 | 0.0012 | 6.4037 | 3.95E-07 |
| 7 | ARS-BFGL-NGS-104684 | 19339413 | 19239413 | 19439413 | A | B | 0.1318 | 0.0023 | 6.6963 | 2.01E-07 |
| 7 | BovineHD0700005733 | 19342782 | 19242782 | 19442782 | A | B | 0.1691 | 0.0025 | 7.2302 | 5.89E-08 |
| 7 | BovineHD0700005738 | 19373525 | 19273525 | 19473525 | A | B | 0.1690 | 0.0025 | 7.2164 | 6.08E-08 |
| 7 | BovineHD0700005739 | 19382328 | 19282328 | 19482328 | B | A | 0.1690 | 0.0025 | 7.2164 | 6.08E-08 |
| 7 | BovineHD0700005740 | 19382970 | 19282970 | 19482970 | B | A | 0.1690 | 0.0025 | 7.2071 | 6.21E-08 |
| 7 | BovineHD0700005741 | 19388961 | 19288961 | 19488961 | B | A | 0.1690 | 0.0025 | 7.2164 | 6.08E-08 |
| 7 | BovineHD0700005749 | 19407665 | 19307665 | 19507665 | A | B | 0.1639 | 0.0025 | 6.8066 | 1.56E-07 |
| 7 | Hapmap56111-rs29019494 | 106096645 | 105996645 | 106196645 | A | B | 0.1821 | 0.0032 | 6.7719 | 1.69E-07 |
| 8 | BovineHD0800023917 | 78628433 | 78528433 | 78728433 | B | A | -0.0908 | 0.0014 | 6.1560 | 6.98E-07 |
| 8 | BovineHD0800024040 | 79157821 | 79057821 | 79257821 | A | B | -0.1094 | 0.0017 | 6.7139 | 1.93E-07 |
| 8 | BovineHD0800024041 | 79167259 | 79067259 | 79267259 | A | B | -0.1093 | 0.0017 | 6.7033 | 1.98E-07 |
| 8 | BovineHD0800024042 | 79178729 | 79078729 | 79278729 | A | B | -0.1094 | 0.0017 | 6.7168 | 1.92E-07 |
| 8 | BovineHD0800024069 | 79336769 | 79236769 | 79436769 | B | A | -0.0707 | 0.0012 | 7.4346 | 3.68E-08 |
| 8 | BovineHD0800024079 | 79406602 | 79306602 | 79506602 | A | B | -0.0838 | 0.0012 | 6.9493 | 1.12E-07 |
| 8 | BovineHD0800025432 | 84331485 | 84231485 | 84431485 | A | B | -0.4906 | 0.0063 | 7.8103 | 1.55E-08 |
| 8 | ARS-BFGL-NGS-119337 | 86042480 | 85942480 | 86142480 | B | A | -0.1581 | 0.0028 | 6.2938 | 5.08E-07 |
| 8 | BovineHD0800026107 | 86554470 | 86454470 | 86654470 | B | A | -0.1532 | 0.0024 | 6.3767 | 4.20E-07 |
| 8 | BovineHD0800026108 | 86556263 | 86456263 | 86656263 | B | A | -0.1532 | 0.0024 | 6.3767 | 4.20E-07 |
| 9 | BovineHD0900012464 | 44368160 | 44268160 | 44468160 | A | B | 0.0918 | 0.0013 | 7.8445 | 1.43E-08 |
| 9 | BovineHD0900012637 | 44965261 | 44865261 | 45065261 | A | B | -0.0915 | 0.0013 | 7.1729 | 6.72E-08 |
| 9 | BovineHD0900012643 | 44989866 | 44889866 | 45089866 | B | A | -0.0910 | 0.0013 | 7.0410 | 9.10E-08 |
| 9 | BovineHD0900012644 | 44994161 | 44894161 | 45094161 | A | B | -0.0910 | 0.0013 | 7.0410 | 9.10E-08 |
| 9 | BovineHD0900013423 | 48122653 | 48022653 | 48222653 | A | B | 0.2327 | 0.0030 | 8.1418 | 7.21E-09 |
| 9 | BovineHD0900013424 | 48125976 | 48025976 | 48225976 | B | A | 0.2335 | 0.0030 | 8.2229 | 5.98E-09 |
| 9 | BovineHD0900013427 | 48130311 | 48030311 | 48230311 | B | A | 0.2335 | 0.0030 | 8.2229 | 5.98E-09 |
| 9 | BovineHD4100007421 | 48133749 | 48033749 | 48233749 | A | B | 0.2335 | 0.0030 | 8.2229 | 5.98E-09 |
| 9 | BovineHD0900013434 | 48144835 | 48044835 | 48244835 | B | A | 0.2330 | 0.0030 | 8.1829 | 6.56E-09 |
| 9 | BovineHD0900013435 | 48146895 | 48046895 | 48246895 | B | A | 0.2499 | 0.0028 | 7.4746 | 3.35E-08 |
| 9 | BovineHD0900020189 | 71626328 | 71526328 | 71726328 | A | B | -0.0670 | 0.0012 | 6.1587 | 6.94E-07 |
| 9 | BovineHD0900020288 | 72118168 | 72018168 | 72218168 | A | B | -0.2531 | 0.0039 | 6.3947 | 4.03E-07 |
| 12 | BovineHD1200009199 | 31234131 | 31134131 | 31334131 | A | B | 0.1625 | 0.0024 | 7.7229 | 1.89E-08 |
| 12 | BovineHD1200009200 | 31235054 | 31135054 | 31335054 | B | A | 0.1625 | 0.0024 | 7.7229 | 1.89E-08 |
| 12 | BovineHD4100009495 | 31246005 | 31146005 | 31346005 | A | B | 0.1646 | 0.0024 | 7.8782 | 1.32E-08 |
| 12 | ARS-BFGL-NGS-77086 | 31247454 | 31147454 | 31347454 | A | B | 0.1215 | 0.0022 | 6.2451 | 5.69E-07 |
| 12 | BovineHD1200009212 | 31257193 | 31157193 | 31357193 | A | B | 0.1619 | 0.0024 | 7.5433 | 2.86E-08 |
| 17 | BovineHD1700019994 | 66377715 | 66277715 | 66477715 | A | B | -0.0666 | 0.0012 | 6.3735 | 4.23E-07 |
| 17 | BovineHD1700020022 | 66429338 | 66329338 | 66529338 | A | B | -0.0930 | 0.0013 | 6.5785 | 2.64E-07 |
| 17 | BovineHD1700020030 | 66439682 | 66339682 | 66539682 | A | B | -0.1133 | 0.0016 | 6.4843 | 3.28E-07 |
| 17 | BovineHD1700020033 | 66442725 | 66342725 | 66542725 | A | B | -0.1133 | 0.0016 | 6.4843 | 3.28E-07 |
| 17 | BovineHD1700020034 | 66443423 | 66343423 | 66543423 | A | B | -0.1133 | 0.0016 | 6.4843 | 3.28E-07 |
| 17 | BovineHD1700020035 | 66444556 | 66344556 | 66544556 | B | A | -0.1133 | 0.0016 | 6.4843 | 3.28E-07 |
| 17 | ARS-BFGL-NGS-3756 | 66445449 | 66345449 | 66545449 | A | B | -0.1133 | 0.0016 | 6.4843 | 3.28E-07 |
| 17 | BovineHD1700020036 | 66445904 | 66345904 | 66545904 | A | B | -0.1133 | 0.0016 | 6.4843 | 3.28E-07 |
| 17 | BovineHD1700020037 | 66447784 | 66347784 | 66547784 | A | B | -0.1134 | 0.0016 | 6.4993 | 3.17E-07 |
| 17 | BovineHD1700020041 | 66456535 | 66356535 | 66556535 | A | B | -0.1099 | 0.0016 | 6.1669 | 6.81E-07 |
| 18 | BovineHD1800003041 | 8790922 | 8690922 | 8890922 | A | B | -0.1724 | 0.0024 | 9.2139 | 6.11E-10 |
| 18 | BovineHD1800003325 | 9506480 | 9406480 | 9606480 | A | B | -0.2717 | 0.0049 | 6.3187 | 4.80E-07 |
| 18 | BovineHD1800003544 | 9926826 | 9826826 | 10026826 | A | B | -0.3754 | 0.0055 | 7.7701 | 1.70E-08 |
| 18 | BovineHD1800003590 | 10042619 | 9942619 | 10142619 | B | A | -0.4907 | 0.0060 | 7.6831 | 2.07E-08 |
| 18 | BovineHD1800003776 | 10615357 | 10515357 | 10715357 | A | B | -0.3477 | 0.0047 | 9.8889 | 1.29E-10 |
| 18 | BovineHD1800004006 | 11349111 | 11249111 | 11449111 | A | B | -0.5144 | 0.0066 | 12.0477 | 8.96E-13 |
| 22 | BovineHD2200005265 | 18271433 | 18171433 | 18371433 | B | A | -0.5857 | 0.0067 | 7.4815 | 3.30E-08 |
| 22 | BovineHD2200013622 | 46852806 | 46752806 | 46952806 | A | B | -0.3841 | 0.0055 | 6.1125 | 7.72E-07 |
| 22 | BovineHD2200013643 | 46888884 | 46788884 | 46988884 | A | B | -0.6556 | 0.0072 | 8.3812 | 4.16E-09 |
| 22 | BovineHD2200013666 | 46959142 | 46859142 | 47059142 | A | B | -0.3929 | 0.0057 | 6.0123 | 9.72E-07 |
| 22 | BovineHD2200013706 | 47065048 | 46965048 | 47165048 | B | A | -0.4131 | 0.0057 | 7.1382 | 7.27E-08 |
| 22 | BovineHD2200013712 | 47084663 | 46984663 | 47184663 | A | B | -0.3794 | 0.0054 | 6.3635 | 4.33E-07 |
| 22 | BovineHD2200013714 | 47086766 | 46986766 | 47186766 | B | A | -0.7030 | 0.0073 | 10.2126 | 6.13E-11 |
| 22 | BovineHD2200013716 | 47092221 | 46992221 | 47192221 | A | B | -0.3820 | 0.0054 | 6.4514 | 3.54E-07 |
| 29 | BovineHD2900012696 | 41382863 | 41282863 | 41482863 | A | B | -0.3438 | 0.0051 | 6.6882 | 2.05E-07 |
| 29 | BovineHD2900012031 | 41692680 | 41592680 | 41792680 | A | B | -0.3065 | 0.0046 | 6.3554 | 4.41E-07 |
| 29 | BovineHD3300000875 | 41693503 | 41593503 | 41793503 | A | B | -0.2931 | 0.0045 | 6.1531 | 7.03E-07 |
| 29 | BovineHD2900012041 | 41695636 | 41595636 | 41795636 | B | A | -0.3065 | 0.0046 | 6.3554 | 4.41E-07 |
| 29 | BovineHD2900012042 | 41696227 | 41596227 | 41796227 | A | B | -0.3065 | 0.0046 | 6.3554 | 4.41E-07 |
| 29 | BovineHD2900012746 | 41763109 | 41663109 | 41863109 | A | B | -0.3133 | 0.0048 | 6.0260 | 9.42E-07 |
| 29 | BovineHD2900012750 | 41787029 | 41687029 | 41887029 | B | A | -0.3254 | 0.0049 | 6.4945 | 3.20E-07 |

^1^Positions of SNPs based on ARS-UCD1.2 Bos taurus genome assembly

^2^Start and end position for region of 100 kb down and upstream of each significant SNP

^3^Alleles of significant SNPs

Table S3 – Chromosome (BTA), SNP and position in bp (a region of 100 kb down and upstream of each significant SNP) alleles of SNP and p-values for the SNP effects for stayability (STAY) of Nellore females.

| BTA | SNP | ^1^Position | ^2^Start position | ^2^End position | ^3^A1 | ^3^A2 | BETA | SE | ${-log}_{10}(p-value)$ | p-value |
| --- | --- | --- | --- | --- | --- | --- | --- | --- | --- | --- |
| 2 | BovineHD0200035262 | 120894962 | 120794962 | 120994962 | A | B | 0.2686 | 0.0045 | 8.1837 | 6.55E-09 |
| 2 | BovineHD0200035276 | 120917758 | 120817758 | 121017758 | B | A | 0.2234 | 0.0042 | 6.6046 | 2.49E-07 |
| 2 | BovineHD0200035317 | 121014227 | 120914227 | 121114227 | B | A | 0.1713 | 0.0033 | 6.2232 | 5.98E-07 |
| 2 | Hapmap29048-BTA-157271 | 121015841 | 120915841 | 121115841 | A | B | 0.1695 | 0.0033 | 6.1097 | 7.77E-07 |
| 2 | BovineHD0200036225 | 124122307 | 124022307 | 124222307 | A | B | -0.1056 | 0.0014 | 6.3200 | 4.79E-07 |
| 2 | BovineHD0200036537 | 125373682 | 125273682 | 125473682 | A | B | 0.0782 | 0.0021 | 8.0731 | 8.45E-09 |
| 2 | BovineHD0200036595 | 125479428 | 125379428 | 125579428 | A | B | -0.2120 | 0.0045 | 6.8334 | 1.47E-07 |
| 2 | BovineHD4100001645 | 125561143 | 125461143 | 125661143 | A | B | -0.2202 | 0.0045 | 7.3972 | 4.01E-08 |
| 2 | BovineHD0200036640 | 125564049 | 125464049 | 125664049 | A | B | -0.2206 | 0.0045 | 7.4133 | 3.86E-08 |
| 2 | BovineHD0200036644 | 125574703 | 125474703 | 125674703 | A | B | -0.0859 | 0.0015 | 9.5907 | 2.57E-10 |
| 2 | BovineHD0200036648 | 125596823 | 125496823 | 125696823 | A | B | -0.2101 | 0.0040 | 8.5458 | 2.85E-09 |
| 2 | BovineHD0200036656 | 125635937 | 125535937 | 125735937 | B | A | -0.2531 | 0.0047 | 8.9600 | 1.10E-09 |
| 2 | BovineHD0200036670 | 125678511 | 125578511 | 125778511 | B | A | -0.4115 | 0.0092 | 6.2546 | 5.56E-07 |
| 2 | BovineHD0200036676 | 125697430 | 125597430 | 125797430 | A | B | -0.2817 | 0.0057 | 7.5324 | 2.93E-08 |
| 2 | BovineHD0200036679 | 125707702 | 125607702 | 125807702 | A | B | -0.2833 | 0.0057 | 7.6430 | 2.28E-08 |
| 2 | BovineHD0200036686 | 125737605 | 125637605 | 125837605 | B | A | -0.2833 | 0.0057 | 7.6430 | 2.28E-08 |
| 2 | BovineHD0200036687 | 125742333 | 125642333 | 125842333 | A | B | -0.2804 | 0.0060 | 6.7711 | 1.69E-07 |
| 2 | ARS-BFGL-NGS-15428 | 125756528 | 125656528 | 125856528 | A | B | -0.2804 | 0.0060 | 6.7711 | 1.69E-07 |
| 2 | BovineHD0200036692 | 125764283 | 125664283 | 125864283 | A | B | -0.2833 | 0.0057 | 7.6430 | 2.28E-08 |
| 2 | BovineHD0200036698 | 125791205 | 125691205 | 125891205 | A | B | -0.3103 | 0.0062 | 7.7427 | 1.81E-08 |
| 2 | BovineHD0200036700 | 125804296 | 125704296 | 125904296 | A | B | -0.3068 | 0.0060 | 8.0527 | 8.86E-09 |
| 2 | BovineHD0200036707 | 125832164 | 125732164 | 125932164 | A | B | -0.3055 | 0.0060 | 7.9379 | 1.15E-08 |
| 2 | BTB-00115953 | 125835970 | 125735970 | 125935970 | A | B | -0.3055 | 0.0060 | 7.9379 | 1.15E-08 |
| 2 | BovineHD0200036711 | 125849656 | 125749656 | 125949656 | B | A | -0.4877 | 0.0103 | 6.8489 | 1.42E-07 |
| 2 | BovineHD0200036713 | 125854307 | 125754307 | 125954307 | A | B | -0.3052 | 0.0060 | 7.9191 | 1.20E-08 |
| 2 | BovineHD0200036714 | 125857473 | 125757473 | 125957473 | A | B | -0.3052 | 0.0060 | 7.9191 | 1.20E-08 |
| 2 | BovineHD0200036715 | 125862235 | 125762235 | 125962235 | B | A | -0.3052 | 0.0060 | 7.9191 | 1.20E-08 |
| 2 | BovineHD0200036722 | 125891278 | 125791278 | 125991278 | A | B | -0.3079 | 0.0060 | 8.0766 | 8.38E-09 |
| 2 | BovineHD0200040286 | 125906852 | 125806852 | 126006852 | B | A | -0.3016 | 0.0060 | 7.7249 | 1.88E-08 |
| 2 | BovineHD0200036727 | 125918679 | 125818679 | 126018679 | A | B | -0.4891 | 0.0103 | 6.9387 | 1.15E-07 |
| 2 | BovineHD0200036728 | 125923129 | 125823129 | 126023129 | A | B | -0.3049 | 0.0060 | 7.9490 | 1.12E-08 |
| 2 | BovineHD0200036730 | 125931255 | 125831255 | 126031255 | A | B | -0.3044 | 0.0060 | 7.9199 | 1.20E-08 |
| 2 | BovineHD0200036731 | 125936747 | 125836747 | 126036747 | A | B | -0.3011 | 0.0060 | 7.6961 | 2.01E-08 |
| 2 | BovineHD0200036735 | 125955166 | 125855166 | 126055166 | B | A | -0.4470 | 0.0093 | 7.0637 | 8.63E-08 |
| 2 | BovineHD0200036737 | 125963366 | 125863366 | 126063366 | B | A | -0.4777 | 0.0099 | 7.1570 | 6.97E-08 |
| 2 | BovineHD0200036763 | 126059896 | 125959896 | 126159896 | B | A | -0.5154 | 0.0108 | 7.0374 | 9.18E-08 |
| 2 | BovineHD0200036811 | 126199837 | 126099837 | 126299837 | A | B | -0.4964 | 0.0108 | 6.5165 | 3.04E-07 |
| 2 | BovineHD0200036814 | 126209428 | 126109428 | 126309428 | A | B | -0.4974 | 0.0108 | 6.5233 | 3.00E-07 |
| 2 | BovineHD0200036815 | 126216772 | 126116772 | 126316772 | B | A | -0.4974 | 0.0108 | 6.5233 | 3.00E-07 |
| 2 | BovineHD0200036816 | 126221467 | 126121467 | 126321467 | A | B | -0.5010 | 0.0112 | 6.1454 | 7.15E-07 |
| 2 | BovineHD0200036818 | 126230900 | 126130900 | 126330900 | A | B | -0.5010 | 0.0112 | 6.1454 | 7.15E-07 |
| 2 | BovineHD0200036828 | 126252239 | 126152239 | 126352239 | B | A | -0.5009 | 0.0113 | 6.1253 | 7.49E-07 |
| 2 | BovineHD0200036833 | 126272662 | 126172662 | 126372662 | A | B | -0.3275 | 0.0064 | 7.9102 | 1.23E-08 |
| 2 | BovineHD0200036835 | 126280441 | 126180441 | 126380441 | B | A | -0.5346 | 0.0111 | 7.0960 | 8.02E-08 |
| 2 | BovineHD0200036838 | 126294898 | 126194898 | 126394898 | A | B | -0.5440 | 0.0110 | 7.4851 | 3.27E-08 |
| 2 | BovineHD0200036846 | 126341842 | 126241842 | 126441842 | A | B | -0.0804 | 0.0018 | 6.1383 | 7.27E-07 |
| 2 | BovineHD0200036848 | 126351252 | 126251252 | 126451252 | A | B | -0.3006 | 0.0059 | 7.8518 | 1.41E-08 |
| 2 | BovineHD0200036849 | 126357202 | 126257202 | 126457202 | A | B | -0.5305 | 0.0111 | 6.9939 | 1.01E-07 |
| 2 | BovineHD0200036857 | 126391334 | 126291334 | 126491334 | B | A | -0.5745 | 0.0114 | 7.7632 | 1.73E-08 |
| 2 | BovineHD0200036864 | 126411289 | 126311289 | 126511289 | A | B | -0.5800 | 0.0113 | 8.0023 | 9.95E-09 |
| 2 | BovineHD0200036868 | 126427346 | 126327346 | 126527346 | B | A | -0.5800 | 0.0113 | 8.0023 | 9.95E-09 |
| 2 | BovineHD0200036869 | 126432262 | 126332262 | 126532262 | A | B | -0.5745 | 0.0114 | 7.7632 | 1.73E-08 |
| 2 | BovineHD0200036870 | 126433864 | 126333864 | 126533864 | A | B | -0.5608 | 0.0109 | 8.1145 | 7.68E-09 |
| 2 | BovineHD0200036873 | 126437224 | 126337224 | 126537224 | B | A | -0.5800 | 0.0113 | 8.0023 | 9.95E-09 |
| 2 | BovineHD0200036877 | 126447591 | 126347591 | 126547591 | B | A | -0.2870 | 0.0051 | 9.7492 | 1.78E-10 |
| 2 | BovineHD0200036888 | 126481229 | 126381229 | 126581229 | B | A | -0.6018 | 0.0117 | 8.0442 | 9.03E-09 |
| 2 | BovineHD0200036910 | 126596365 | 126496365 | 126696365 | A | B | -0.3316 | 0.0062 | 8.7033 | 1.98E-09 |
| 2 | BovineHD0200036926 | 126654159 | 126554159 | 126754159 | B | A | -0.5544 | 0.0106 | 8.3123 | 4.87E-09 |
| 2 | BovineHD0200037025 | 126972577 | 126872577 | 127072577 | B | A | -0.0801 | 0.0014 | 9.3835 | 4.14E-10 |
| 2 | BovineHD0200037051 | 127037548 | 126937548 | 127137548 | A | B | -0.3059 | 0.0061 | 7.6055 | 2.48E-08 |
| 2 | BovineHD0200037094 | 127145687 | 127045687 | 127245687 | B | A | -0.2688 | 0.0055 | 7.4398 | 3.63E-08 |
| 2 | BovineHD0200037110 | 127208929 | 127108929 | 127308929 | B | A | -0.0806 | 0.0016 | 7.9541 | 1.11E-08 |
| 2 | BovineHD0200037114 | 127227843 | 127127843 | 127327843 | A | B | -0.0731 | 0.0017 | 6.0554 | 8.80E-07 |
| 2 | BovineHD4100001660 | 127292762 | 127192762 | 127392762 | B | A | -0.0796 | 0.0014 | 9.5244 | 2.99E-10 |
| 2 | BovineHD0200037137 | 127293824 | 127193824 | 127393824 | B | A | -0.0796 | 0.0014 | 9.5360 | 2.91E-10 |
| 2 | BovineHD0200037159 | 127323570 | 127223570 | 127423570 | A | B | -0.2314 | 0.0046 | 7.7661 | 1.71E-08 |
| 2 | BovineHD0200037163 | 127333970 | 127233970 | 127433970 | B | A | -0.2369 | 0.0046 | 8.0257 | 9.42E-09 |
| 2 | BovineHD0200037164 | 127338512 | 127238512 | 127438512 | B | A | -0.2380 | 0.0046 | 8.0396 | 9.13E-09 |
| 2 | BovineHD0200037165 | 127343239 | 127243239 | 127443239 | B | A | -0.2399 | 0.0046 | 8.2228 | 5.99E-09 |
| 2 | BovineHD0200037183 | 127403337 | 127303337 | 127503337 | A | B | -0.0774 | 0.0015 | 8.6323 | 2.33E-09 |
| 2 | BovineHD0200037201 | 127490407 | 127390407 | 127590407 | A | B | -0.2622 | 0.0053 | 7.6184 | 2.41E-08 |
| 2 | BovineHD0200037203 | 127496188 | 127396188 | 127596188 | B | A | -0.2510 | 0.0051 | 7.3007 | 5.00E-08 |
| 2 | ARS-BFGL-NGS-118648 | 129013033 | 128913033 | 129113033 | A | B | -0.1071 | 0.0014 | 6.7485 | 1.78E-07 |
| 2 | BovineHD0200037695 | 129095315 | 128995315 | 129195315 | A | B | -0.1018 | 0.0014 | 6.1208 | 7.57E-07 |
| 2 | BovineHD0200037768 | 129353800 | 129253800 | 129453800 | A | B | -0.1043 | 0.0015 | 6.0331 | 9.27E-07 |
| 2 | BovineHD0200039544 | 134609828 | 134509828 | 134709828 | A | B | -0.1460 | 0.0020 | 6.3443 | 4.53E-07 |
| 2 | BovineHD0200039714 | 135069280 | 134969280 | 135169280 | B | A | -0.1214 | 0.0017 | 6.3509 | 4.46E-07 |
| 2 | BovineHD0200039719 | 135081375 | 134981375 | 135181375 | A | B | -0.1148 | 0.0016 | 6.3421 | 4.55E-07 |
| 3 | BovineHD0300036269 | 83332655 | 83232655 | 83432655 | A | B | 0.0923 | 0.0016 | 7.8904 | 1.29E-08 |
| 3 | BovineHD0300024340 | 84662228 | 84562228 | 84762228 | A | B | 0.1847 | 0.0032 | 7.9177 | 1.21E-08 |
| 3 | BovineHD0300024459 | 85051292 | 84951292 | 85151292 | A | B | -0.1063 | 0.0015 | 6.1059 | 7.84E-07 |
| 3 | BovineHD0300024460 | 85051929 | 84951929 | 85151929 | B | A | -0.1127 | 0.0015 | 6.3798 | 4.17E-07 |
| 3 | BovineHD0300024461 | 85053031 | 84953031 | 85153031 | B | A | -0.1122 | 0.0015 | 6.3283 | 4.70E-07 |
| 3 | BovineHD0300024462 | 85056741 | 84956741 | 85156741 | B | A | -0.1122 | 0.0015 | 6.3280 | 4.70E-07 |
| 3 | BovineHD0300024464 | 85058550 | 84958550 | 85158550 | A | B | -0.1123 | 0.0015 | 6.3262 | 4.72E-07 |
| 3 | BovineHD0300024468 | 85064822 | 84964822 | 85164822 | B | A | -0.1123 | 0.0015 | 6.3262 | 4.72E-07 |
| 3 | BovineHD0300024483 | 85138684 | 85038684 | 85238684 | B | A | -0.1050 | 0.0014 | 6.2923 | 5.10E-07 |
| 3 | BovineHD0300024567 | 85477905 | 85377905 | 85577905 | B | A | -0.1236 | 0.0016 | 7.4849 | 3.27E-08 |
| 3 | BovineHD0300024600 | 85589630 | 85489630 | 85689630 | A | B | -0.1208 | 0.0017 | 6.0256 | 9.43E-07 |
| 3 | BovineHD0300024640 | 85756884 | 85656884 | 85856884 | B | A | -0.1113 | 0.0016 | 6.1161 | 7.65E-07 |
| 3 | BovineHD0300025690 | 88950093 | 88850093 | 89050093 | A | B | -0.4678 | 0.0039 | 6.6571 | 2.20E-07 |
| 3 | BovineHD0300027343 | 94415641 | 94315641 | 94515641 | B | A | -0.7960 | 0.0070 | 6.0045 | 9.90E-07 |
| 3 | BovineHD0300027344 | 94416489 | 94316489 | 94516489 | A | B | -0.9051 | 0.0075 | 6.8242 | 1.50E-07 |
| 3 | BovineHD0300027345 | 94418008 | 94318008 | 94518008 | B | A | -0.9757 | 0.0077 | 7.4207 | 3.80E-08 |
| 3 | BovineHD0300027353 | 94470905 | 94370905 | 94570905 | A | B | -0.9164 | 0.0075 | 6.9092 | 1.23E-07 |
| 3 | BovineHD0300027370 | 94570511 | 94470511 | 94670511 | A | B | -0.9392 | 0.0075 | 7.4175 | 3.82E-08 |
| 3 | BovineHD0300027373 | 94584777 | 94484777 | 94684777 | B | A | -0.8917 | 0.0073 | 6.9031 | 1.25E-07 |
| 3 | BovineHD0300027375 | 94591946 | 94491946 | 94691946 | A | B | -0.9462 | 0.0075 | 7.4685 | 3.40E-08 |
| 3 | BovineHD0300027513 | 95194898 | 95094898 | 95294898 | A | B | -0.9673 | 0.0078 | 7.2730 | 5.33E-08 |
| 3 | BovineHD0300027522 | 95227477 | 95127477 | 95327477 | B | A | -0.9724 | 0.0078 | 7.3271 | 4.71E-08 |
| 3 | BovineHD0300027527 | 95255144 | 95155144 | 95355144 | B | A | -0.9872 | 0.0078 | 7.4755 | 3.35E-08 |
| 3 | BTA-95308-no-rs | 95291661 | 95191661 | 95391661 | B | A | 0.8049 | 0.0082 | 8.8465 | 1.42E-09 |
| 3 | BovineHD0300027531 | 95306394 | 95206394 | 95406394 | A | B | 0.7195 | 0.0079 | 7.6323 | 2.33E-08 |
| 3 | Hapmap58527-rs29018282 | 96163545 | 96063545 | 96263545 | A | B | 0.7206 | 0.0080 | 7.3880 | 4.09E-08 |
| 3 | Hapmap52129-rs29016142 | 96294680 | 96194680 | 96394680 | B | A | 0.7104 | 0.0079 | 7.3720 | 4.25E-08 |
| 3 | ARS-BFGL-NGS-67035 | 96947843 | 96847843 | 97047843 | A | B | -0.6307 | 0.0063 | 11.6453 | 2.26E-12 |
| 3 | BovineHD0300027986 | 97017532 | 96917532 | 97117532 | B | A | 0.1768 | 0.0025 | 11.9127 | 1.22E-12 |
| 3 | BovineHD0300027989 | 97021182 | 96921182 | 97121182 | A | B | 0.1791 | 0.0025 | 6.1196 | 7.59E-07 |
| 3 | BTB-00147905 | 97023337 | 96923337 | 97123337 | A | B | 0.1791 | 0.0025 | 6.1196 | 7.59E-07 |
| 3 | BovineHD0300027997 | 97051671 | 96951671 | 97151671 | A | B | -0.4422 | 0.0053 | 8.2182 | 6.05E-09 |
| 3 | BTB-01919893 | 97070925 | 96970925 | 97170925 | B | A | 0.2108 | 0.0026 | 7.3705 | 4.26E-08 |
| 3 | BovineHD0300028013 | 97106709 | 97006709 | 97206709 | A | B | 0.1315 | 0.0022 | 8.0791 | 8.33E-09 |
| 3 | BovineHD0300028017 | 97127451 | 97027451 | 97227451 | A | B | 0.2755 | 0.0032 | 8.7689 | 1.70E-09 |
| 3 | BovineHD0300028020 | 97132012 | 97032012 | 97232012 | B | A | 0.2755 | 0.0032 | 8.7689 | 1.70E-09 |
| 3 | BovineHD0300028022 | 97134694 | 97034694 | 97234694 | B | A | -0.3857 | 0.0032 | 8.7689 | 1.70E-09 |
| 3 | BovineHD0300028025 | 97144061 | 97044061 | 97244061 | A | B | 0.2114 | 0.0026 | 7.4323 | 3.70E-08 |
| 3 | BovineHD4100002363 | 97155868 | 97055868 | 97255868 | A | B | -0.4763 | 0.0061 | 7.1928 | 6.42E-08 |
| 3 | BovineHD0300028033 | 97182222 | 97082222 | 97282222 | A | B | 0.2347 | 0.0036 | 9.8180 | 1.52E-10 |
| 3 | BovineHD0300028048 | 97217726 | 97117726 | 97317726 | A | B | -0.3864 | 0.0053 | 6.3553 | 4.41E-07 |
| 3 | BovineHD0300028070 | 97288380 | 97188380 | 97388380 | B | A | 0.1998 | 0.0039 | 6.0016 | 9.96E-07 |
| 3 | BovineHD0300028185 | 97568452 | 97468452 | 97668452 | A | B | 0.1173 | 0.0022 | 6.6943 | 2.02E-07 |
| 3 | BovineHD0300028349 | 98012275 | 97912275 | 98112275 | B | A | -0.5712 | 0.0050 | 7.7977 | 1.59E-08 |
| 3 | BovineHD0300028358 | 98057144 | 97957144 | 98157144 | B | A | 0.2227 | 0.0034 | 9.8429 | 1.44E-10 |
| 3 | BovineHD0300028369 | 98100979 | 98000979 | 98200979 | B | A | -0.7801 | 0.0056 | 11.3455 | 4.51E-12 |
| 3 | BovineHD0300028381 | 98146410 | 98046410 | 98246410 | A | B | -0.7347 | 0.0055 | 10.3699 | 4.27E-11 |
| 3 | BovineHD0300028385 | 98166506 | 98066506 | 98266506 | A | B | -0.7296 | 0.0055 | 10.2348 | 5.82E-11 |
| 3 | BovineHD0300028390 | 98187368 | 98087368 | 98287368 | A | B | -0.7286 | 0.0055 | 10.2152 | 6.09E-11 |
| 3 | BovineHD0300028395 | 98200605 | 98100605 | 98300605 | A | B | -0.7296 | 0.0055 | 10.2348 | 5.82E-11 |
| 3 | BovineHD0300028396 | 98205419 | 98105419 | 98305419 | A | B | 0.1814 | 0.0028 | 9.8231 | 1.50E-10 |
| 3 | BovineHD0300028405 | 98230941 | 98130941 | 98330941 | B | A | 0.1856 | 0.0035 | 6.4266 | 3.74E-07 |
| 3 | BovineHD0300028406 | 98232293 | 98132293 | 98332293 | A | B | 0.1776 | 0.0028 | 9.5463 | 2.84E-10 |
| 3 | BovineHD0300028407 | 98234237 | 98134237 | 98334237 | B | A | 0.1813 | 0.0028 | 9.8097 | 1.55E-10 |
| 3 | BovineHD0300028408 | 98235934 | 98135934 | 98335934 | A | B | 0.1856 | 0.0035 | 6.4266 | 3.74E-07 |
| 3 | BovineHD0300028409 | 98238757 | 98138757 | 98338757 | A | B | 0.1856 | 0.0035 | 6.4266 | 3.74E-07 |
| 3 | BovineHD0300028412 | 98244243 | 98144243 | 98344243 | A | B | 0.1856 | 0.0035 | 6.4266 | 3.74E-07 |
| 3 | BovineHD0300028413 | 98245263 | 98145263 | 98345263 | A | B | 0.1856 | 0.0035 | 6.4266 | 3.74E-07 |
| 3 | BovineHD0300028598 | 98886449 | 98786449 | 98986449 | B | A | 0.1120 | 0.0021 | 6.5747 | 2.66E-07 |
| 3 | BovineHD0300028654 | 99112755 | 99012755 | 99212755 | B | A | 0.1244 | 0.0024 | 6.2826 | 5.22E-07 |
| 3 | BovineHD0300028655 | 99119483 | 99019483 | 99219483 | A | B | 0.1247 | 0.0024 | 6.3105 | 4.89E-07 |
| 3 | BovineHD0300028656 | 99121156 | 99021156 | 99221156 | A | B | 0.1252 | 0.0024 | 6.3551 | 4.41E-07 |
| 3 | BovineHD0300028657 | 99124406 | 99024406 | 99224406 | A | B | 0.1247 | 0.0024 | 6.3105 | 4.89E-07 |
| 3 | BovineHD0300028658 | 99129833 | 99029833 | 99229833 | A | B | 0.1247 | 0.0024 | 6.3105 | 4.89E-07 |
| 3 | BovineHD0300028659 | 99137027 | 99037027 | 99237027 | A | B | 0.1252 | 0.0024 | 6.3551 | 4.41E-07 |
| 3 | BovineHD0300028661 | 99148368 | 99048368 | 99248368 | B | A | 0.1252 | 0.0024 | 6.3551 | 4.41E-07 |
| 3 | BovineHD0300028662 | 99152632 | 99052632 | 99252632 | A | B | 0.1341 | 0.0023 | 8.1900 | 6.46E-09 |
| 3 | BovineHD0300028663 | 99159384 | 99059384 | 99259384 | A | B | 0.1254 | 0.0024 | 6.3775 | 4.19E-07 |
| 3 | BovineHD0300028665 | 99164968 | 99064968 | 99264968 | A | B | 0.1255 | 0.0024 | 6.3842 | 4.13E-07 |
| 3 | BovineHD0300028667 | 99178761 | 99078761 | 99278761 | A | B | 0.1440 | 0.0023 | 8.9597 | 1.10E-09 |
| 3 | BovineHD0300036334 | 99203708 | 99103708 | 99303708 | B | A | 0.1317 | 0.0023 | 7.8907 | 1.29E-08 |
| 3 | BovineHD0300028680 | 99260362 | 99160362 | 99360362 | B | A | 0.1439 | 0.0023 | 9.0680 | 8.55E-10 |
| 3 | BovineHD0300028682 | 99270414 | 99170414 | 99370414 | A | B | 0.1412 | 0.0023 | 8.7228 | 1.89E-09 |
| 3 | BovineHD0300032427 | 111934355 | 111834355 | 112034355 | B | A | -0.7833 | 0.0067 | 6.4922 | 3.22E-07 |
| 5 | BovineHD0500028221 | 98479661 | 98379661 | 98579661 | A | B | -0.0748 | 0.0014 | 7.5690 | 2.70E-08 |
| 5 | BovineHD0500028243 | 98540791 | 98440791 | 98640791 | A | B | -0.0988 | 0.0019 | 7.7036 | 1.98E-08 |
| 5 | BovineHD0500028347 | 98822711 | 98722711 | 98922711 | A | B | -0.0835 | 0.0015 | 8.5038 | 3.13E-09 |
| 5 | BovineHD0500028663 | 99690951 | 99590951 | 99790951 | B | A | -0.0854 | 0.0018 | 6.0287 | 9.36E-07 |
| 5 | BovineHD0500028720 | 99868728 | 99768728 | 99968728 | A | B | -0.0752 | 0.0015 | 6.6030 | 2.49E-07 |
| 5 | BovineHD0500028904 | 100508949 | 100408949 | 100608949 | B | A | -0.0786 | 0.0014 | 8.0991 | 7.96E-09 |
| 5 | BovineHD0500036210 | 100623510 | 100523510 | 100723510 | A | B | 0.1214 | 0.0035 | 6.5760 | 2.65E-07 |
| 5 | BovineHD0500028927 | 100658909 | 100558909 | 100758909 | A | B | -0.0704 | 0.0014 | 6.5154 | 3.05E-07 |
| 5 | BovineHD0500029017 | 100912450 | 100812450 | 101012450 | A | B | -0.0726 | 0.0014 | 7.0230 | 9.48E-08 |
| 5 | BovineHD0500029022 | 100930308 | 100830308 | 101030308 | A | B | -0.1551 | 0.0031 | 7.0841 | 8.24E-08 |
| 5 | BovineHD0500029028 | 100952842 | 100852842 | 101052842 | B | A | -0.1742 | 0.0033 | 7.8206 | 1.51E-08 |
| 5 | BovineHD0500029030 | 100959360 | 100859360 | 101059360 | B | A | -0.1742 | 0.0033 | 7.8206 | 1.51E-08 |
| 5 | BovineHD0500029040 | 100982874 | 100882874 | 101082874 | A | B | -0.1827 | 0.0034 | 7.6896 | 2.04E-08 |
| 5 | BovineHD0500029042 | 100986678 | 100886678 | 101086678 | A | B | -0.1822 | 0.0034 | 7.6576 | 2.20E-08 |
| 5 | BovineHD0500029044 | 100988857 | 100888857 | 101088857 | A | B | -0.1811 | 0.0034 | 7.5746 | 2.66E-08 |
| 5 | BovineHD0500029045 | 100989766 | 100889766 | 101089766 | A | B | -0.1829 | 0.0034 | 7.7141 | 1.93E-08 |
| 5 | BovineHD0500029046 | 100990309 | 100890309 | 101090309 | A | B | -0.1822 | 0.0034 | 7.6576 | 2.20E-08 |
| 5 | BovineHD0500029047 | 100990795 | 100890795 | 101090795 | B | A | -0.1822 | 0.0034 | 7.6576 | 2.20E-08 |
| 5 | BovineHD0500029049 | 100992602 | 100892602 | 101092602 | B | A | -0.1809 | 0.0034 | 7.5641 | 2.73E-08 |
| 5 | BovineHD0500029050 | 100993190 | 100893190 | 101093190 | A | B | -0.1820 | 0.0034 | 7.6372 | 2.31E-08 |
| 5 | BovineHD0500029051 | 100993672 | 100893672 | 101093672 | B | A | -0.1822 | 0.0034 | 7.6576 | 2.20E-08 |
| 5 | Hapmap25949-BTA-74741 | 100994547 | 100894547 | 101094547 | A | B | -0.1822 | 0.0034 | 7.6576 | 2.20E-08 |
| 5 | BovineHD0500029052 | 100995536 | 100895536 | 101095536 | A | B | -0.1822 | 0.0034 | 7.6576 | 2.20E-08 |
| 5 | BovineHD0500029054 | 100999642 | 100899642 | 101099642 | A | B | -0.1822 | 0.0034 | 7.6576 | 2.20E-08 |
| 5 | BovineHD0500029056 | 101004164 | 100904164 | 101104164 | A | B | -0.1826 | 0.0034 | 7.6949 | 2.02E-08 |
| 5 | BovineHD0500029090 | 101077927 | 100977927 | 101177927 | B | A | -0.1697 | 0.0032 | 7.8670 | 1.36E-08 |
| 5 | BovineHD4100004004 | 101081882 | 100981882 | 101181882 | A | B | -0.0851 | 0.0018 | 6.3962 | 4.02E-07 |
| 5 | BovineHD0500029131 | 101221922 | 101121922 | 101321922 | A | B | 0.0866 | 0.0015 | 6.8399 | 1.45E-07 |
| 7 | BovineHD0700004938 | 16370132 | 16270132 | 16470132 | A | B | -0.0976 | 0.0016 | 8.6210 | 2.39E-09 |
| 7 | BovineHD0700004941 | 16383383 | 16283383 | 16483383 | B | A | -0.0787 | 0.0016 | 6.2280 | 5.91E-07 |
| 7 | BovineHD0700005249 | 17553883 | 17453883 | 17653883 | A | B | -0.0723 | 0.0014 | 6.4308 | 3.71E-07 |
| 7 | BovineHD0700005423 | 18288687 | 18188687 | 18388687 | A | B | 0.0937 | 0.0027 | 6.0511 | 8.89E-07 |
| 7 | Hapmap43686-BTA-78412 | 18490225 | 18390225 | 18590225 | B | A | -0.0862 | 0.0023 | 8.8990 | 1.26E-09 |
| 7 | BovineHD0700005486 | 18494068 | 18394068 | 18594068 | A | B | 0.1316 | 0.0053 | 7.8267 | 1.49E-08 |
| 7 | BovineHD0700005508 | 18558647 | 18458647 | 18658647 | A | B | 0.1156 | 0.0034 | 6.4102 | 3.89E-07 |
| 7 | BovineHD0700005515 | 18569768 | 18469768 | 18669768 | A | B | -0.0433 | 0.0014 | 6.1749 | 6.69E-07 |
| 7 | BTB-00295891 | 18570908 | 18470908 | 18670908 | B | A | -0.0494 | 0.0014 | 7.8428 | 1.44E-08 |
| 7 | BovineHD0700005524 | 18578613 | 18478613 | 18678613 | B | A | 0.1297 | 0.0055 | 7.0855 | 8.21E-08 |
| 7 | BovineHD0700005526 | 18584525 | 18484525 | 18684525 | B | A | 0.1189 | 0.0052 | 6.7739 | 1.68E-07 |
| 7 | BovineHD0700005530 | 18596221 | 18496221 | 18696221 | B | A | 0.1181 | 0.0052 | 6.7116 | 1.94E-07 |
| 7 | BovineHD0700005531 | 18598851 | 18498851 | 18698851 | A | B | 0.1180 | 0.0052 | 6.6853 | 2.06E-07 |
| 7 | BovineHD0700005532 | 18599529 | 18499529 | 18699529 | A | B | 0.1253 | 0.0053 | 7.2131 | 6.12E-08 |
| 7 | UA-IFASA-8172 | 18603438 | 18503438 | 18703438 | B | A | 0.1177 | 0.0052 | 6.6604 | 2.19E-07 |
| 7 | BovineHD0700005535 | 18607538 | 18507538 | 18707538 | A | B | 0.1258 | 0.0053 | 7.2670 | 5.41E-08 |
| 7 | BovineHD0700005537 | 18619681 | 18519681 | 18719681 | A | B | -0.0529 | 0.0017 | 6.1153 | 7.67E-07 |
| 7 | BovineHD0700005538 | 18624062 | 18524062 | 18724062 | B | A | -0.0529 | 0.0017 | 6.1153 | 7.67E-07 |
| 7 | BovineHD0700005539 | 18626255 | 18526255 | 18726255 | A | B | 0.1253 | 0.0053 | 7.2131 | 6.12E-08 |
| 7 | BovineHD0700005542 | 18648300 | 18548300 | 18748300 | A | B | -0.0531 | 0.0017 | 6.1538 | 7.02E-07 |
| 7 | BovineHD0700005543 | 18652751 | 18552751 | 18752751 | B | A | -0.0531 | 0.0017 | 6.1538 | 7.02E-07 |
| 7 | BovineHD0700005545 | 18659720 | 18559720 | 18759720 | A | B | -0.0507 | 0.0014 | 7.9729 | 1.06E-08 |
| 7 | BovineHD0700005549 | 18676149 | 18576149 | 18776149 | B | A | -0.0801 | 0.0023 | 7.9342 | 1.16E-08 |
| 7 | BovineHD0700005559 | 18723175 | 18623175 | 18823175 | A | B | -0.0590 | 0.0017 | 7.7296 | 1.86E-08 |
| 7 | BovineHD0700033620 | 18740072 | 18640072 | 18840072 | B | A | -0.0585 | 0.0017 | 7.6039 | 2.49E-08 |
| 7 | BovineHD0700005565 | 18771796 | 18671796 | 18871796 | A | B | -0.0504 | 0.0016 | 6.7530 | 1.77E-07 |
| 7 | BovineHD0700005631 | 19038570 | 18938570 | 19138570 | B | A | -0.0506 | 0.0016 | 6.9342 | 1.16E-07 |
| 7 | BovineHD0700005632 | 19043069 | 18943069 | 19143069 | B | A | -0.0566 | 0.0017 | 7.6154 | 2.42E-08 |
| 7 | BovineHD0700005643 | 19096070 | 18996070 | 19196070 | B | A | -0.0457 | 0.0015 | 6.3016 | 4.99E-07 |
| 7 | BovineHD0700005648 | 19107522 | 19007522 | 19207522 | A | B | -0.0606 | 0.0019 | 6.9362 | 1.16E-07 |
| 7 | BovineHD0700005651 | 19112335 | 19012335 | 19212335 | A | B | -0.0540 | 0.0018 | 6.0745 | 8.42E-07 |
| 7 | BovineHD0700005653 | 19114252 | 19014252 | 19214252 | A | B | -0.0544 | 0.0018 | 6.1434 | 7.19E-07 |
| 7 | BovineHD0700005654 | 19115126 | 19015126 | 19215126 | B | A | -0.0553 | 0.0018 | 6.3409 | 4.56E-07 |
| 7 | BovineHD0700005676 | 19162485 | 19062485 | 19262485 | B | A | -0.0478 | 0.0014 | 7.2584 | 5.52E-08 |
| 7 | BovineHD0700005679 | 19171356 | 19071356 | 19271356 | B | A | -0.0476 | 0.0014 | 7.1939 | 6.40E-08 |
| 7 | BovineHD0700005680 | 19175464 | 19075464 | 19275464 | B | A | -0.0444 | 0.0014 | 6.2621 | 5.47E-07 |
| 7 | BovineHD0700005682 | 19180822 | 19080822 | 19280822 | B | A | 0.1366 | 0.0054 | 7.9746 | 1.06E-08 |
| 7 | BovineHD0700005683 | 19185466 | 19085466 | 19285466 | A | B | 0.1366 | 0.0054 | 7.9746 | 1.06E-08 |
| 7 | BovineHD0700005692 | 19227351 | 19127351 | 19327351 | B | A | 0.1333 | 0.0054 | 7.7140 | 1.93E-08 |
| 7 | BovineHD0700005697 | 19236502 | 19136502 | 19336502 | A | B | -0.0512 | 0.0014 | 8.2853 | 5.18E-09 |
| 7 | BovineHD0700005720 | 19310698 | 19210698 | 19410698 | A | B | -0.0996 | 0.0029 | 7.4582 | 3.48E-08 |
| 7 | BovineHD0700005721 | 19314666 | 19214666 | 19414666 | B | A | -0.0975 | 0.0027 | 8.4156 | 3.84E-09 |
| 7 | BovineHD0700005735 | 19349358 | 19249358 | 19449358 | B | A | -0.0525 | 0.0014 | 8.6203 | 2.40E-09 |
| 7 | BovineHD0700005744 | 19396676 | 19296676 | 19496676 | A | B | -0.0479 | 0.0014 | 7.2889 | 5.14E-08 |
| 7 | BovineHD0700005749 | 19407665 | 19307665 | 19507665 | A | B | 0.0766 | 0.0031 | 7.8818 | 1.31E-08 |
| 7 | BovineHD0700005765 | 19501368 | 19401368 | 19601368 | B | A | -0.0558 | 0.0016 | 7.4626 | 3.45E-08 |
| 7 | BovineHD0700005771 | 19539618 | 19439618 | 19639618 | A | B | 0.1397 | 0.0055 | 8.2241 | 5.97E-09 |
| 7 | BovineHD0700005772 | 19546266 | 19446266 | 19646266 | A | B | 0.0744 | 0.0034 | 6.0518 | 8.88E-07 |
| 7 | BovineHD0700005774 | 19560991 | 19460991 | 19660991 | A | B | 0.0750 | 0.0034 | 6.2153 | 6.09E-07 |
| 7 | BovineHD0700005775 | 19564946 | 19464946 | 19664946 | B | A | 0.0807 | 0.0035 | 7.0011 | 9.97E-08 |
| 7 | BovineHD0700005776 | 19570534 | 19470534 | 19670534 | A | B | 0.0766 | 0.0034 | 6.4341 | 3.68E-07 |
| 7 | BovineHD0700005777 | 19576220 | 19476220 | 19676220 | A | B | 0.0881 | 0.0035 | 8.0089 | 9.80E-09 |
| 7 | BovineHD0700005778 | 19582665 | 19482665 | 19682665 | B | A | 0.0881 | 0.0035 | 8.0089 | 9.80E-09 |
| 7 | BovineHD0700005782 | 19601934 | 19501934 | 19701934 | B | A | -0.0525 | 0.0014 | 8.5399 | 2.88E-09 |
| 7 | BovineHD0700005783 | 19612322 | 19512322 | 19712322 | A | B | 0.0948 | 0.0040 | 7.3386 | 4.59E-08 |
| 7 | BovineHD0700005824 | 19805543 | 19705543 | 19905543 | A | B | 0.0803 | 0.0032 | 7.8343 | 1.46E-08 |
| 7 | BovineHD0700005860 | 19948369 | 19848369 | 20048369 | B | A | 0.0772 | 0.0031 | 7.7743 | 1.68E-08 |
| 7 | BovineHD0700032986 | 19974241 | 19874241 | 20074241 | A | B | 0.0956 | 0.0037 | 8.3120 | 4.88E-09 |
| 7 | BovineHD0700005866 | 19977349 | 19877349 | 20077349 | A | B | 0.0953 | 0.0037 | 8.2570 | 5.53E-09 |
| 7 | BovineHD0700005867 | 19982084 | 19882084 | 20082084 | A | B | 0.0956 | 0.0037 | 8.3001 | 5.01E-09 |
| 7 | BovineHD0700005868 | 19989123 | 19889123 | 20089123 | B | A | 0.0953 | 0.0037 | 8.2570 | 5.53E-09 |
| 7 | BovineHD0700005873 | 20018689 | 19918689 | 20118689 | B | A | 0.0869 | 0.0037 | 7.0536 | 8.84E-08 |
| 7 | BovineHD0700005875 | 20031719 | 19931719 | 20131719 | B | A | 0.0941 | 0.0037 | 8.0599 | 8.71E-09 |
| 7 | BovineHD0700005876 | 20036738 | 19936738 | 20136738 | B | A | 0.0935 | 0.0037 | 7.9976 | 1.01E-08 |
| 7 | BovineHD0700005879 | 20050474 | 19950474 | 20150474 | A | B | 0.0967 | 0.0037 | 8.4689 | 3.40E-09 |
| 7 | BovineHD0700005880 | 20053188 | 19953188 | 20153188 | A | B | 0.0930 | 0.0037 | 7.9480 | 1.13E-08 |
| 7 | BovineHD0700005881 | 20057534 | 19957534 | 20157534 | B | A | 0.0931 | 0.0037 | 7.9688 | 1.07E-08 |
| 7 | BovineHD0700005883 | 20064774 | 19964774 | 20164774 | A | B | 0.0967 | 0.0037 | 8.4689 | 3.40E-09 |
| 7 | BovineHD0700005906 | 20153088 | 20053088 | 20253088 | B | A | 0.1094 | 0.0051 | 6.0225 | 9.49E-07 |
| 7 | BovineHD0700005968 | 20374841 | 20274841 | 20474841 | A | B | -0.0745 | 0.0023 | 6.8173 | 1.52E-07 |
| 7 | BovineHD0700005967 | 20375720 | 20275720 | 20475720 | B | A | -0.0745 | 0.0023 | 6.8173 | 1.52E-07 |
| 7 | BovineHD0700005972 | 20401149 | 20301149 | 20501149 | A | B | 0.1134 | 0.0051 | 6.3273 | 4.71E-07 |
| 7 | BovineHD0700005973 | 20401996 | 20301996 | 20501996 | B | A | -0.0802 | 0.0024 | 7.1800 | 6.61E-08 |
| 7 | BovineHD0700005974 | 20408948 | 20308948 | 20508948 | B | A | -0.0578 | 0.0016 | 7.9698 | 1.07E-08 |
| 7 | BovineHD0700005975 | 20417099 | 20317099 | 20517099 | B | A | 0.1153 | 0.0051 | 6.5055 | 3.12E-07 |
| 7 | BovineHD0700005979 | 20431993 | 20331993 | 20531993 | A | B | 0.1291 | 0.0052 | 7.7231 | 1.89E-08 |
| 7 | BovineHD0700005980 | 20432703 | 20332703 | 20532703 | B | A | 0.1106 | 0.0051 | 6.1057 | 7.84E-07 |
| 7 | BovineHD0700005982 | 20440154 | 20340154 | 20540154 | A | B | 0.1233 | 0.0054 | 6.6204 | 2.40E-07 |
| 7 | BovineHD0700005984 | 20451707 | 20351707 | 20551707 | A | B | 0.1129 | 0.0051 | 6.4213 | 3.79E-07 |
| 7 | BovineHD0700005989 | 20472083 | 20372083 | 20572083 | B | A | 0.1132 | 0.0051 | 6.4423 | 3.61E-07 |
| 7 | BovineHD0700005990 | 20474073 | 20374073 | 20574073 | A | B | 0.1421 | 0.0056 | 8.1809 | 6.59E-09 |
| 7 | BovineHD0700005991 | 20475220 | 20375220 | 20575220 | A | B | 0.1421 | 0.0056 | 8.1809 | 6.59E-09 |
| 7 | BovineHD0700005995 | 20484512 | 20384512 | 20584512 | B | A | -0.0495 | 0.0016 | 6.5322 | 2.94E-07 |
| 7 | BovineHD0700006000 | 20506133 | 20406133 | 20606133 | A | B | 0.1195 | 0.0055 | 6.1896 | 6.46E-07 |
| 7 | BovineHD0700006001 | 20511430 | 20411430 | 20611430 | A | B | 0.0919 | 0.0042 | 6.0961 | 8.02E-07 |
| 7 | BovineHD0700006002 | 20517963 | 20417963 | 20617963 | A | B | 0.1201 | 0.0055 | 6.2532 | 5.58E-07 |
| 7 | BovineHD0700006003 | 20520942 | 20420942 | 20620942 | A | B | 0.1200 | 0.0055 | 6.2156 | 6.09E-07 |
| 7 | BovineHD0700006006 | 20535133 | 20435133 | 20635133 | A | B | 0.0926 | 0.0042 | 6.1817 | 6.58E-07 |
| 7 | BovineHD0700006042 | 20677668 | 20577668 | 20777668 | A | B | 0.1238 | 0.0054 | 6.6474 | 2.25E-07 |
| 7 | BovineHD0700006061 | 20747092 | 20647092 | 20847092 | B | A | -0.2390 | 0.0068 | 8.0778 | 8.36E-09 |
| 7 | BovineHD0700006062 | 20749727 | 20649727 | 20849727 | A | B | -0.2426 | 0.0068 | 8.2252 | 5.95E-09 |
| 7 | BovineHD0700006069 | 20770746 | 20670746 | 20870746 | A | B | -0.2379 | 0.0064 | 8.7733 | 1.69E-09 |
| 7 | BovineHD0700006072 | 20775225 | 20675225 | 20875225 | A | B | -0.2007 | 0.0059 | 7.4972 | 3.18E-08 |
| 7 | BovineHD0700006080 | 20799991 | 20699991 | 20899991 | B | A | -0.1681 | 0.0055 | 6.2342 | 5.83E-07 |
| 7 | BovineHD0700006093 | 20887371 | 20787371 | 20987371 | A | B | 0.1185 | 0.0052 | 6.7272 | 1.87E-07 |
| 7 | BovineHD0700006099 | 20905514 | 20805514 | 21005514 | B | A | 0.1189 | 0.0052 | 6.7651 | 1.72E-07 |
| 7 | BovineHD0700006100 | 20907156 | 20807156 | 21007156 | A | B | 0.1189 | 0.0052 | 6.7651 | 1.72E-07 |
| 7 | BovineHD0700006101 | 20909147 | 20809147 | 21009147 | A | B | 0.1189 | 0.0052 | 6.7651 | 1.72E-07 |
| 7 | BovineHD0700006102 | 20909704 | 20809704 | 21009704 | A | B | 0.1189 | 0.0052 | 6.7651 | 1.72E-07 |
| 7 | BovineHD0700006103 | 20912796 | 20812796 | 21012796 | B | A | 0.1189 | 0.0052 | 6.7651 | 1.72E-07 |
| 7 | BovineHD0700006109 | 20939693 | 20839693 | 21039693 | A | B | 0.1151 | 0.0053 | 6.0313 | 9.30E-07 |
| 7 | BovineHD0700006118 | 20961001 | 20861001 | 21061001 | B | A | -0.0506 | 0.0015 | 7.7933 | 1.61E-08 |
| 7 | BovineHD0700006139 | 21027256 | 20927256 | 21127256 | A | B | 0.0948 | 0.0042 | 6.6995 | 2.00E-07 |
| 7 | BovineHD0700006157 | 21094221 | 20994221 | 21194221 | A | B | 0.0987 | 0.0043 | 6.8135 | 1.54E-07 |
| 7 | BovineHD0700006199 | 21192993 | 21092993 | 21292993 | A | B | 0.1168 | 0.0050 | 6.8762 | 1.33E-07 |
| 7 | BovineHD0700006206 | 21222341 | 21122341 | 21322341 | A | B | 0.1318 | 0.0054 | 7.5318 | 2.94E-08 |
| 7 | BovineHD0700006209 | 21242403 | 21142403 | 21342403 | A | B | 0.1331 | 0.0054 | 7.6295 | 2.35E-08 |
| 7 | BovineHD0700006210 | 21242948 | 21142948 | 21342948 | A | B | -0.0599 | 0.0017 | 8.1606 | 6.91E-09 |
| 7 | BovineHD0700006216 | 21264231 | 21164231 | 21364231 | B | A | 0.1258 | 0.0052 | 7.4224 | 3.78E-08 |
| 7 | BovineHD0700006241 | 21381658 | 21281658 | 21481658 | A | B | 0.1161 | 0.0052 | 6.5268 | 2.97E-07 |
| 7 | BovineHD0700006249 | 21416511 | 21316511 | 21516511 | B | A | 0.1167 | 0.0053 | 6.2185 | 6.05E-07 |
| 7 | BovineHD0700011012 | 36825617 | 36725617 | 36925617 | A | B | -0.1096 | 0.0015 | 6.7858 | 1.64E-07 |
| 7 | BovineHD0700027731 | 92848570 | 92748570 | 92948570 | A | B | -0.2247 | 0.0029 | 6.3464 | 4.50E-07 |
| 7 | BovineHD0700028144 | 94273399 | 94173399 | 94373399 | B | A | 0.0841 | 0.0015 | 6.2193 | 6.04E-07 |
| 7 | BovineHD0700028159 | 94320864 | 94220864 | 94420864 | A | B | 0.1214 | 0.0021 | 6.9350 | 1.16E-07 |
| 7 | BovineHD0700028194 | 94386639 | 94286639 | 94486639 | A | B | 0.1214 | 0.0021 | 7.0019 | 9.96E-08 |
| 7 | BovineHD0700028260 | 94516701 | 94416701 | 94616701 | A | B | 0.0910 | 0.0015 | 7.3753 | 4.21E-08 |
| 7 | BovineHD0700028316 | 94638772 | 94538772 | 94738772 | B | A | 0.0849 | 0.0016 | 6.1186 | 7.61E-07 |
| 7 | BovineHD0700028493 | 95206532 | 95106532 | 95306532 | A | B | -0.1891 | 0.0025 | 6.0184 | 9.58E-07 |
| 7 | BovineHD0700028495 | 95208850 | 95108850 | 95308850 | A | B | -0.1894 | 0.0025 | 6.0361 | 9.20E-07 |
| 7 | BovineHD0700028496 | 95209522 | 95109522 | 95309522 | B | A | -0.1894 | 0.0025 | 6.0361 | 9.20E-07 |
| 7 | BovineHD0700028813 | 96205366 | 96105366 | 96305366 | B | A | 0.0782 | 0.0014 | 6.1842 | 6.54E-07 |
| 7 | BovineHD0700028928 | 96467565 | 96367565 | 96567565 | A | B | -0.1192 | 0.0016 | 6.1246 | 7.51E-07 |
| 7 | BovineHD0700031628 | 105956774 | 105856774 | 106056774 | A | B | 0.1306 | 0.0025 | 6.4760 | 3.34E-07 |
| 7 | ARS-BFGL-NGS-70170 | 105990353 | 105890353 | 106090353 | A | B | 0.1606 | 0.0027 | 8.1201 | 7.58E-09 |
| 7 | BovineHD0700031668 | 106086580 | 105986580 | 106186580 | B | A | -0.0862 | 0.0018 | 6.3116 | 4.88E-07 |
| 7 | BovineHD0700031692 | 106204994 | 106104994 | 106304994 | B | A | 0.1453 | 0.0043 | 6.2687 | 5.39E-07 |
| 7 | BovineHD0700031701 | 106249472 | 106149472 | 106349472 | A | B | 0.1480 | 0.0041 | 7.1469 | 7.13E-08 |
| 7 | BovineHD0700031817 | 106602215 | 106502215 | 106702215 | B | A | -0.1037 | 0.0022 | 6.3623 | 4.34E-07 |
| 7 | BovineHD1000003179 | 106690658 | 106590658 | 106790658 | A | B | -0.0802 | 0.0017 | 6.2089 | 6.18E-07 |
| 7 | BovineHD4100006419 | 106975839 | 106875839 | 107075839 | A | B | -0.1886 | 0.0034 | 8.6080 | 2.47E-09 |
| 7 | BovineHD0700032160 | 107633419 | 107533419 | 107733419 | B | A | -0.1724 | 0.0035 | 6.6937 | 2.02E-07 |
| 7 | BovineHD0700032171 | 107654624 | 107554624 | 107754624 | B | A | -0.1475 | 0.0031 | 6.3027 | 4.98E-07 |
| 7 | BovineHD0700032328 | 108248782 | 108148782 | 108348782 | B | A | -0.0968 | 0.0021 | 6.1245 | 7.51E-07 |
| 9 | BovineHD0900012464 | 44368160 | 44268160 | 44468160 | A | B | 0.1106 | 0.0016 | 10.4694 | 3.39E-11 |
| 9 | BovineHD0900012468 | 44376181 | 44276181 | 44476181 | A | B | 0.1377 | 0.0026 | 6.3769 | 4.20E-07 |
| 9 | BovineHD0900012473 | 44385925 | 44285925 | 44485925 | B | A | 0.1377 | 0.0026 | 6.3769 | 4.20E-07 |
| 9 | BovineHD0900012477 | 44390242 | 44290242 | 44490242 | A | B | 0.1377 | 0.0026 | 6.3769 | 4.20E-07 |
| 9 | BovineHD0900012637 | 44965261 | 44865261 | 45065261 | A | B | 0.0790 | 0.0015 | 6.1661 | 6.82E-07 |
| 9 | BovineHD0900012643 | 44989866 | 44889866 | 45089866 | B | A | 0.0787 | 0.0015 | 6.1181 | 7.62E-07 |
| 9 | BovineHD0900012644 | 44994161 | 44894161 | 45094161 | A | B | 0.0787 | 0.0015 | 6.1181 | 7.62E-07 |
| 9 | BovineHD0900012649 | 45008663 | 44908663 | 45108663 | B | A | -0.7854 | 0.0100 | 7.2879 | 5.15E-08 |
| 9 | BovineHD0900013423 | 48122653 | 48022653 | 48222653 | A | B | 0.2026 | 0.0037 | 7.0441 | 9.03E-08 |
| 9 | BovineHD0900013424 | 48125976 | 48025976 | 48225976 | B | A | 0.2034 | 0.0037 | 7.0856 | 8.21E-08 |
| 9 | BovineHD0900013427 | 48130311 | 48030311 | 48230311 | B | A | 0.2034 | 0.0037 | 7.0856 | 8.21E-08 |
| 9 | BovineHD4100007421 | 48133749 | 48033749 | 48233749 | A | B | 0.2034 | 0.0037 | 7.0856 | 8.21E-08 |
| 9 | BovineHD0900013434 | 48144835 | 48044835 | 48244835 | B | A | 0.2040 | 0.0037 | 7.1349 | 7.33E-08 |
| 9 | BovineHD0900013435 | 48146895 | 48046895 | 48246895 | B | A | 0.2031 | 0.0034 | 8.1938 | 6.40E-09 |
| 9 | BovineHD0900013692 | 49051468 | 48951468 | 49151468 | A | B | -0.6926 | 0.0093 | 6.5870 | 2.59E-07 |
| 9 | BTB-02006579 | 49257679 | 49157679 | 49357679 | A | B | 0.1962 | 0.0033 | 8.0980 | 7.98E-09 |
| 9 | BovineHD0900013771 | 49271986 | 49171986 | 49371986 | B | A | 0.1966 | 0.0033 | 8.1579 | 6.95E-09 |
| 9 | BovineHD0900015340 | 55086475 | 54986475 | 55186475 | B | A | -1.5224 | 0.0125 | 6.9708 | 1.07E-07 |
| 9 | BovineHD0900015356 | 55155573 | 55055573 | 55255573 | B | A | 1.0848 | 0.0125 | 6.9631 | 1.09E-07 |
| 10 | BovineHD1000019267 | 66812736 | 66712736 | 66912736 | B | A | -0.4675 | 0.0039 | 6.8115 | 1.54E-07 |
| 10 | BovineHD1000024447 | 85339737 | 85239737 | 85439737 | B | A | -0.0850 | 0.0017 | 7.0855 | 8.21E-08 |
| 10 | BovineHD1000031569 | 85548689 | 85448689 | 85648689 | B | A | -0.0741 | 0.0015 | 6.7798 | 1.66E-07 |
| 10 | BovineHD1000024492 | 85559015 | 85459015 | 85659015 | A | B | -0.0741 | 0.0015 | 6.7816 | 1.65E-07 |
| 10 | BovineHD1000024496 | 85572410 | 85472410 | 85672410 | A | B | -0.0706 | 0.0015 | 6.2048 | 6.24E-07 |
| 10 | ARS-BFGL-NGS-16573 | 85643599 | 85543599 | 85743599 | A | B | -0.0717 | 0.0014 | 6.9666 | 1.08E-07 |
| 10 | BovineHD1000024552 | 85735767 | 85635767 | 85835767 | A | B | -0.0722 | 0.0015 | 6.8048 | 1.57E-07 |
| 10 | BovineHD1000024565 | 85768012 | 85668012 | 85868012 | A | B | 0.0790 | 0.0021 | 7.7603 | 1.74E-08 |
| 10 | BovineHD1000024567 | 85775860 | 85675860 | 85875860 | B | A | -0.1028 | 0.0020 | 7.2916 | 5.11E-08 |
| 10 | BovineHD1000024568 | 85777174 | 85677174 | 85877174 | B | A | 0.0790 | 0.0021 | 7.7606 | 1.74E-08 |
| 10 | BovineHD1000024570 | 85787290 | 85687290 | 85887290 | A | B | 0.0789 | 0.0021 | 7.7650 | 1.72E-08 |
| 10 | BovineHD4100008306 | 85792402 | 85692402 | 85892402 | A | B | 0.0791 | 0.0021 | 7.7908 | 1.62E-08 |
| 10 | BovineHD1000024571 | 85796107 | 85696107 | 85896107 | A | B | 0.0746 | 0.0021 | 7.0971 | 8.00E-08 |
| 10 | BovineHD1000024572 | 85799991 | 85699991 | 85899991 | A | B | 0.0784 | 0.0021 | 7.6526 | 2.23E-08 |
| 10 | BovineHD1000024625 | 86023643 | 85923643 | 86123643 | B | A | -0.0920 | 0.0019 | 6.6632 | 2.17E-07 |
| 10 | BovineHD1000024985 | 86797673 | 86697673 | 86897673 | A | B | -0.1158 | 0.0022 | 7.7767 | 1.67E-08 |
| 10 | BovineHD1000024989 | 86863386 | 86763386 | 86963386 | B | A | -0.0681 | 0.0014 | 6.4126 | 3.87E-07 |
| 10 | BovineHD1000025086 | 87205082 | 87105082 | 87305082 | A | B | -0.0682 | 0.0014 | 6.3227 | 4.76E-07 |
| 10 | BovineHD1000025088 | 87218629 | 87118629 | 87318629 | A | B | -0.0682 | 0.0014 | 6.3354 | 4.62E-07 |
| 10 | BovineHD1000030815 | 87253561 | 87153561 | 87353561 | A | B | -0.0723 | 0.0014 | 7.0835 | 8.25E-08 |
| 10 | BovineHD1000025103 | 87301213 | 87201213 | 87401213 | A | B | -0.0707 | 0.0014 | 6.8711 | 1.35E-07 |
| 10 | BovineHD1000025109 | 87317156 | 87217156 | 87417156 | A | B | -0.1033 | 0.0020 | 7.5673 | 2.71E-08 |
| 12 | BovineHD4100009489 | 30390289 | 30290289 | 30490289 | A | B | 0.1819 | 0.0033 | 7.0031 | 9.93E-08 |
| 12 | BovineHD4100009490 | 30407613 | 30307613 | 30507613 | A | B | 0.1812 | 0.0033 | 7.0029 | 9.93E-08 |
| 12 | BovineHD1200009148 | 31097191 | 30997191 | 31197191 | A | B | 0.1708 | 0.0034 | 6.0386 | 9.15E-07 |
| 12 | BovineHD1200009165 | 31126054 | 31026054 | 31226054 | A | B | 0.1720 | 0.0034 | 6.1260 | 7.48E-07 |
| 12 | BovineHD1200009166 | 31130156 | 31030156 | 31230156 | A | B | 0.1718 | 0.0034 | 6.1118 | 7.73E-07 |
| 12 | BovineHD1200009167 | 31131877 | 31031877 | 31231877 | B | A | 0.1720 | 0.0034 | 6.1260 | 7.48E-07 |
| 12 | BovineHD1200009199 | 31234131 | 31134131 | 31334131 | A | B | 0.1783 | 0.0029 | 8.6334 | 2.33E-09 |
| 12 | BovineHD1200009200 | 31235054 | 31135054 | 31335054 | B | A | 0.1783 | 0.0029 | 8.6334 | 2.33E-09 |
| 12 | BovineHD4100009495 | 31246005 | 31146005 | 31346005 | A | B | 0.1830 | 0.0029 | 8.9655 | 1.08E-09 |
| 12 | BovineHD1200009212 | 31257193 | 31157193 | 31357193 | A | B | 0.1790 | 0.0029 | 8.6322 | 2.33E-09 |
| 12 | BovineHD1200009253 | 31390466 | 31290466 | 31490466 | B | A | -0.1184 | 0.0014 | 8.0896 | 8.14E-09 |
| 12 | BovineHD1200009257 | 31412549 | 31312549 | 31512549 | A | B | -0.1184 | 0.0014 | 8.0812 | 8.30E-09 |
| 12 | BovineHD1200009359 | 31797445 | 31697445 | 31897445 | A | B | 0.1669 | 0.0032 | 6.5171 | 3.04E-07 |
| 12 | BovineHD1200009362 | 31804769 | 31704769 | 31904769 | A | B | 0.1538 | 0.0030 | 6.1106 | 7.75E-07 |
| 12 | BovineHD1200009363 | 31805379 | 31705379 | 31905379 | A | B | 0.1538 | 0.0030 | 6.1106 | 7.75E-07 |
| 12 | BovineHD1200009734 | 32942749 | 32842749 | 33042749 | A | B | 0.0918 | 0.0016 | 7.3103 | 4.89E-08 |
| 12 | BovineHD1200009750 | 32990315 | 32890315 | 33090315 | B | A | 0.0970 | 0.0019 | 6.1271 | 7.46E-07 |
| 12 | ARS-BFGL-NGS-23611 | 33103396 | 33003396 | 33203396 | B | A | 0.1268 | 0.0016 | 9.1662 | 6.82E-10 |
| 12 | BovineHD1200027797 | 33181041 | 33081041 | 33281041 | B | A | 0.1188 | 0.0021 | 7.2234 | 5.98E-08 |
| 17 | BovineHD1700009632 | 34385180 | 34285180 | 34485180 | A | B | 0.1608 | 0.0020 | 6.1923 | 6.42E-07 |
| 17 | BovineHD1700019474 | 65004563 | 64904563 | 65104563 | A | B | -0.1672 | 0.0030 | 6.9083 | 1.24E-07 |
| 17 | BovineHD1700019486 | 65038744 | 64938744 | 65138744 | B | A | -0.1017 | 0.0018 | 6.6928 | 2.03E-07 |
| 17 | BovineHD1700019591 | 65327288 | 65227288 | 65427288 | A | B | -0.2468 | 0.0044 | 6.9455 | 1.13E-07 |
| 17 | BovineHD1700019611 | 65383384 | 65283384 | 65483384 | A | B | -0.2444 | 0.0045 | 6.5873 | 2.59E-07 |
| 17 | ARS-BFGL-NGS-27727 | 65655991 | 65555991 | 65755991 | A | B | -0.1317 | 0.0023 | 6.9666 | 1.08E-07 |
| 17 | BovineHD1700019710 | 65658888 | 65558888 | 65758888 | B | A | -0.1272 | 0.0024 | 6.3141 | 4.85E-07 |
| 17 | BovineHD1700019712 | 65661972 | 65561972 | 65761972 | A | B | -0.0914 | 0.0017 | 6.6421 | 2.28E-07 |
| 17 | BovineHD1700019859 | 66034733 | 65934733 | 66134733 | A | B | -0.0871 | 0.0016 | 6.3036 | 4.97E-07 |
| 17 | BovineHD1700020023 | 66430883 | 66330883 | 66530883 | B | A | -0.0801 | 0.0014 | 6.9066 | 1.24E-07 |
| 17 | BovineHD1700020024 | 66431606 | 66331606 | 66531606 | A | B | -0.0801 | 0.0014 | 6.8953 | 1.27E-07 |
| 17 | BovineHD1700020025 | 66432299 | 66332299 | 66532299 | A | B | -0.0801 | 0.0014 | 6.8953 | 1.27E-07 |
| 17 | BovineHD1700020104 | 66600283 | 66500283 | 66700283 | A | B | 0.1303 | 0.0034 | 6.1879 | 6.49E-07 |
| 17 | BovineHD1700020118 | 66649673 | 66549673 | 66749673 | A | B | -0.1212 | 0.0022 | 6.5772 | 2.65E-07 |
| 17 | BovineHD1700020121 | 66660651 | 66560651 | 66760651 | A | B | -0.1219 | 0.0022 | 6.7692 | 1.70E-07 |
| 17 | BovineHD1700020192 | 66885887 | 66785887 | 66985887 | A | B | 0.1008 | 0.0027 | 6.0114 | 9.74E-07 |
| 17 | ARS-BFGL-NGS-20761 | 67203528 | 67103528 | 67303528 | A | B | 0.0830 | 0.0014 | 6.1634 | 6.86E-07 |
| 17 | BovineHD1700020275 | 67237701 | 67137701 | 67337701 | B | A | -0.0934 | 0.0018 | 6.1251 | 7.50E-07 |
| 17 | BovineHD1700020289 | 67300948 | 67200948 | 67400948 | A | B | -0.0774 | 0.0014 | 6.2555 | 5.55E-07 |
| 19 | BovineHD4100013852 | 5337524 | 5237524 | 5437524 | B | A | 0.0774 | 0.0014 | 6.2045 | 6.24E-07 |
| 19 | BovineHD1900004418 | 15726309 | 15626309 | 15826309 | A | B | -0.7117 | 0.0093 | 6.2260 | 5.94E-07 |
| 19 | BovineHD1900005528 | 19081321 | 18981321 | 19181321 | B | A | -0.1085 | 0.0014 | 6.1384 | 7.27E-07 |
| 19 | BovineHD1900005585 | 19304006 | 19204006 | 19404006 | A | B | 0.1027 | 0.0028 | 7.0881 | 8.16E-08 |
| 19 | BovineHD1900005593 | 19342101 | 19242101 | 19442101 | A | B | 0.1030 | 0.0028 | 7.1329 | 7.36E-08 |
| 19 | BovineHD1900005596 | 19347875 | 19247875 | 19447875 | B | A | 0.1029 | 0.0028 | 7.1191 | 7.60E-08 |
| 19 | ARS-BFGL-NGS-107725 | 19348535 | 19248535 | 19448535 | B | A | 0.1030 | 0.0028 | 7.1329 | 7.36E-08 |
| 19 | BovineHD1900005601 | 19353652 | 19253652 | 19453652 | B | A | 0.1035 | 0.0028 | 7.2023 | 6.28E-08 |
| 19 | BovineHD1900005602 | 19354643 | 19254643 | 19454643 | A | B | 0.1031 | 0.0028 | 7.1537 | 7.02E-08 |
| 19 | BovineHD1900005604 | 19357143 | 19257143 | 19457143 | A | B | 0.1031 | 0.0028 | 7.1537 | 7.02E-08 |
| 19 | BovineHD1900005605 | 19358121 | 19258121 | 19458121 | A | B | 0.1031 | 0.0028 | 7.1537 | 7.02E-08 |
| 19 | BovineHD1900005606 | 19359040 | 19259040 | 19459040 | A | B | 0.1031 | 0.0028 | 7.1537 | 7.02E-08 |
| 19 | BovineHD1900005607 | 19362639 | 19262639 | 19462639 | A | B | 0.1031 | 0.0028 | 7.1537 | 7.02E-08 |
| 19 | BovineHD1900005610 | 19366645 | 19266645 | 19466645 | A | B | 0.1031 | 0.0028 | 7.1537 | 7.02E-08 |
| 19 | BovineHD1900005612 | 19370468 | 19270468 | 19470468 | A | B | 0.1008 | 0.0028 | 6.8860 | 1.30E-07 |
| 19 | BovineHD1900005613 | 19371451 | 19271451 | 19471451 | A | B | 0.0962 | 0.0028 | 6.5384 | 2.89E-07 |
| 19 | BovineHD1900005618 | 19380476 | 19280476 | 19480476 | A | B | 0.0949 | 0.0028 | 6.4050 | 3.94E-07 |
| 19 | BovineHD1900005619 | 19385801 | 19285801 | 19485801 | A | B | 0.0952 | 0.0028 | 6.4405 | 3.63E-07 |
| 19 | BovineHD1900005631 | 19409334 | 19309334 | 19509334 | A | B | 0.0977 | 0.0028 | 6.7698 | 1.70E-07 |
| 19 | BovineHD1900005633 | 19410892 | 19310892 | 19510892 | B | A | 0.0977 | 0.0028 | 6.7698 | 1.70E-07 |
| 19 | BovineHD1900005636 | 19414668 | 19314668 | 19514668 | A | B | 0.0977 | 0.0028 | 6.7698 | 1.70E-07 |
| 19 | ARS-BFGL-NGS-16975 | 19904364 | 19804364 | 20004364 | B | A | -0.1461 | 0.0030 | 6.4407 | 3.62E-07 |
| 19 | BovineHD1900006175 | 20981242 | 20881242 | 21081242 | A | B | -0.1494 | 0.0030 | 6.7487 | 1.78E-07 |
| 19 | BovineHD1900006415 | 21901749 | 21801749 | 22001749 | B | A | -0.5781 | 0.0081 | 6.1274 | 7.46E-07 |
| 19 | BovineHD1900015350 | 54071105 | 53971105 | 54171105 | B | A | 0.1222 | 0.0023 | 6.4138 | 3.86E-07 |
| 19 | BovineHD1900016036 | 56071372 | 55971372 | 56171372 | A | B | 0.1748 | 0.0033 | 6.4932 | 3.21E-07 |
| 22 | BovineHD2200013311 | 45695459 | 45595459 | 45795459 | B | A | -0.1427 | 0.0047 | 7.1712 | 6.74E-08 |
| 22 | BovineHD2200013312 | 45697467 | 45597467 | 45797467 | A | B | -0.1330 | 0.0046 | 6.5892 | 2.57E-07 |
| 22 | BovineHD2200013322 | 45719793 | 45619793 | 45819793 | B | A | -0.0483 | 0.0017 | 6.1206 | 7.57E-07 |
| 22 | BovineHD2200013358 | 45876122 | 45776122 | 45976122 | B | A | -0.0435 | 0.0015 | 6.3891 | 4.08E-07 |
| 22 | BovineHD2200013360 | 45886149 | 45786149 | 45986149 | B | A | -0.0450 | 0.0015 | 6.9141 | 1.22E-07 |
| 22 | BovineHD2200013370 | 45976030 | 45876030 | 46076030 | A | B | -0.0401 | 0.0014 | 6.0923 | 8.09E-07 |
| 22 | ARS-BFGL-NGS-817 | 45978694 | 45878694 | 46078694 | B | A | -0.1754 | 0.0038 | 6.8812 | 1.31E-07 |
| 22 | BovineHD2200013375 | 45988174 | 45888174 | 46088174 | A | B | -0.0542 | 0.0014 | 7.1676 | 6.80E-08 |
| 22 | BovineHD2200013376 | 45992536 | 45892536 | 46092536 | A | B | -0.0516 | 0.0014 | 6.5164 | 3.04E-07 |
| 22 | BovineHD2200013378 | 46000744 | 45900744 | 46100744 | A | B | -0.0546 | 0.0014 | 7.2721 | 5.34E-08 |
| 22 | BovineHD2200013379 | 46004799 | 45904799 | 46104799 | A | B | -0.0547 | 0.0014 | 7.2858 | 5.18E-08 |
| 22 | BovineHD2200013388 | 46050377 | 45950377 | 46150377 | A | B | -0.2261 | 0.0041 | 6.4808 | 3.31E-07 |
| 22 | BovineHD2200013399 | 46095367 | 45995367 | 46195367 | B | A | -0.1931 | 0.0054 | 6.4556 | 3.50E-07 |
| 22 | BovineHD2200013411 | 46139321 | 46039321 | 46239321 | B | A | -0.0646 | 0.0015 | 6.1145 | 7.68E-07 |
| 22 | BovineHD2200013426 | 46164744 | 46064744 | 46264744 | A | B | -0.0410 | 0.0015 | 6.0623 | 8.66E-07 |
| 22 | BovineHD2200013437 | 46188862 | 46088862 | 46288862 | A | B | -0.1670 | 0.0044 | 7.3005 | 5.01E-08 |
| 22 | BovineHD2200013476 | 46340416 | 46240416 | 46440416 | A | B | -0.0899 | 0.0026 | 6.2529 | 5.59E-07 |
| 22 | BovineHD2200013477 | 46343124 | 46243124 | 46443124 | A | B | -0.1027 | 0.0029 | 6.4973 | 3.18E-07 |
| 22 | BovineHD2200013495 | 46401242 | 46301242 | 46501242 | A | B | -0.2559 | 0.0036 | 6.7526 | 1.77E-07 |
| 22 | BovineHD2200013503 | 46440893 | 46340893 | 46540893 | B | A | -0.0417 | 0.0014 | 6.5332 | 2.93E-07 |
| 22 | BovineHD2200013541 | 46559904 | 46459904 | 46659904 | A | B | -0.0457 | 0.0016 | 6.5993 | 2.52E-07 |
| 22 | BovineHD2200013542 | 46573613 | 46473613 | 46673613 | A | B | -0.0426 | 0.0014 | 6.8629 | 1.37E-07 |
| 22 | BovineHD2200013546 | 46597639 | 46497639 | 46697639 | A | B | -0.0911 | 0.0025 | 6.5627 | 2.74E-07 |
| 22 | BTA-54671-no-rs | 46609167 | 46509167 | 46709167 | A | B | -0.0934 | 0.0026 | 6.3124 | 4.87E-07 |
| 22 | BovineHD2200013554 | 46647388 | 46547388 | 46747388 | A | B | -0.0964 | 0.0027 | 6.5994 | 2.52E-07 |
| 22 | BTA-07404-no-rs | 46664801 | 46564801 | 46764801 | A | B | 0.1118 | 0.0037 | 6.0182 | 9.59E-07 |
| 22 | BovineHD2200013562 | 46674650 | 46574650 | 46774650 | A | B | -0.1245 | 0.0028 | 6.3846 | 4.12E-07 |
| 22 | BovineHD2200013564 | 46682852 | 46582852 | 46782852 | B | A | -0.1244 | 0.0028 | 6.3810 | 4.16E-07 |
| 22 | BovineHD2200018100 | 46720856 | 46620856 | 46820856 | B | A | -0.1196 | 0.0027 | 6.4358 | 3.67E-07 |
| 22 | BovineHD2200013583 | 46725351 | 46625351 | 46825351 | B | A | -0.1245 | 0.0028 | 6.3454 | 4.51E-07 |
| 22 | BovineHD2200013586 | 46732689 | 46632689 | 46832689 | A | B | -0.1229 | 0.0028 | 6.4061 | 3.93E-07 |
| 22 | BovineHD2200013618 | 46836989 | 46736989 | 46936989 | A | B | -0.1016 | 0.0029 | 6.0484 | 8.94E-07 |
| 22 | BovineHD2200013620 | 46842317 | 46742317 | 46942317 | B | A | -0.0497 | 0.0018 | 6.2107 | 6.16E-07 |
| 22 | BovineHD2200017926 | 46857136 | 46757136 | 46957136 | B | A | -0.0504 | 0.0018 | 6.2473 | 5.66E-07 |
| 22 | BovineHD2200013629 | 46877692 | 46777692 | 46977692 | A | B | -0.0733 | 0.0016 | 6.9918 | 1.02E-07 |
| 22 | BovineHD2200013633 | 46881264 | 46781264 | 46981264 | B | A | -0.0731 | 0.0016 | 6.9609 | 1.09E-07 |
| 22 | BovineHD2200013635 | 46883183 | 46783183 | 46983183 | A | B | -0.1022 | 0.0014 | 6.9678 | 1.08E-07 |
| 22 | ARS-BFGL-NGS-46087 | 46889869 | 46789869 | 46989869 | B | A | -0.0730 | 0.0016 | 6.9407 | 1.15E-07 |
| 22 | BovineHD2200013651 | 46899356 | 46799356 | 46999356 | A | B | 0.1206 | 0.0038 | 6.5336 | 2.93E-07 |
| 22 | BovineHD2200013652 | 46900191 | 46800191 | 47000191 | B | A | -0.0524 | 0.0018 | 6.4762 | 3.34E-07 |
| 22 | BovineHD2200013653 | 46908778 | 46808778 | 47008778 | A | B | -0.0768 | 0.0017 | 6.7847 | 1.64E-07 |
| 22 | BovineHD2200013667 | 46960031 | 46860031 | 47060031 | A | B | -0.0950 | 0.0016 | 6.1506 | 7.07E-07 |
| 22 | BovineHD2200013701 | 47059104 | 46959104 | 47159104 | B | A | -0.1564 | 0.0053 | 6.7050 | 1.97E-07 |
| 22 | BovineHD2200018101 | 47245083 | 47145083 | 47345083 | A | B | -0.0617 | 0.0020 | 7.4304 | 3.71E-08 |
| 22 | BovineHD2200013783 | 47361554 | 47261554 | 47461554 | A | B | -0.1170 | 0.0026 | 6.8193 | 1.52E-07 |
| 22 | BovineHD2200013792 | 47375457 | 47275457 | 47475457 | B | A | -0.1202 | 0.0025 | 7.2200 | 6.03E-08 |
| 22 | BovineHD2200013800 | 47396587 | 47296587 | 47496587 | B | A | -0.1197 | 0.0026 | 7.0441 | 9.03E-08 |
| 22 | BovineHD2200013801 | 47397348 | 47297348 | 47497348 | A | B | -0.0663 | 0.0015 | 6.2469 | 5.66E-07 |
| 22 | BovineHD2200013802 | 47397768 | 47297768 | 47497768 | B | A | -0.0666 | 0.0015 | 6.2924 | 5.10E-07 |
| 22 | BovineHD2200013803 | 47399544 | 47299544 | 47499544 | A | B | -0.0665 | 0.0015 | 6.2869 | 5.16E-07 |
| 22 | BovineHD2200013812 | 47431174 | 47331174 | 47531174 | B | A | -0.0527 | 0.0014 | 6.8565 | 1.39E-07 |
| 22 | BovineHD2200018102 | 47526849 | 47426849 | 47626849 | A | B | -0.1111 | 0.0016 | 6.6765 | 2.11E-07 |
| 22 | BovineHD2200013850 | 47599909 | 47499909 | 47699909 | A | B | -0.0473 | 0.0015 | 7.3773 | 4.19E-08 |
| 22 | BovineHD2200013864 | 47639594 | 47539594 | 47739594 | B | A | -0.0887 | 0.0016 | 6.2704 | 5.36E-07 |
| 22 | BovineHD2200013872 | 47660525 | 47560525 | 47760525 | A | B | -0.0908 | 0.0016 | 6.5418 | 2.87E-07 |
| 22 | ARS-BFGL-NGS-22249 | 47726147 | 47626147 | 47826147 | A | B | -0.0624 | 0.0021 | 7.0741 | 8.43E-08 |
| 22 | BovineHD4100015763 | 47738702 | 47638702 | 47838702 | B | A | -0.0758 | 0.0020 | 6.8935 | 1.28E-07 |
| 22 | BovineHD2200013900 | 47762387 | 47662387 | 47862387 | A | B | -0.2094 | 0.0033 | 6.7397 | 1.82E-07 |
| 22 | BovineHD2200013901 | 47764899 | 47664899 | 47864899 | B | A | -0.1312 | 0.0020 | 6.9706 | 1.07E-07 |
| 22 | BovineHD2200013920 | 47826334 | 47726334 | 47926334 | B | A | -0.0723 | 0.0016 | 6.9659 | 1.08E-07 |
| 22 | BovineHD2200013938 | 47914220 | 47814220 | 48014220 | B | A | -0.0826 | 0.0023 | 6.4362 | 3.66E-07 |
| 22 | BovineHD2200013977 | 48051489 | 47951489 | 48151489 | B | A | -0.0397 | 0.0014 | 6.1085 | 7.79E-07 |
| 22 | BovineHD2200014031 | 48343257 | 48243257 | 48443257 | A | B | -0.0488 | 0.0018 | 6.0668 | 8.57E-07 |

^1^Positions of SNPs based on ARS-UCD1.2 Bos taurus genome assembly

^2^Start and end position for region of 100 kb down and upstream of each significant SNP

^3^Alleles of significant SNPs

Table S4 - Common SNP with significant effect for post-natal mortality (PNM), heifer rebreeding (HR), and stayability (STAY) in Nellore cattle

| ^1^BTA | SNP | ^2^Position | Post-natal mortality | | | |  | Heifer rebreeding | | | |  | Stayability | | | |
| --- | --- | --- | --- | --- | --- | --- | --- | --- | --- | --- | --- | --- | --- | --- | --- | --- |
|  |  |  | BETA | SE | ${-log}_{10}(p-value)$ | p-value |  | BETA | SE | ${-log}_{10}(p-value)$ | p-value |  | BETA | SE | ${-log}_{10}(p-value)$ | p-value |
| 18 | BovineHD1800003544 | 9926826 | 0.80 | 0.01 | 7.77 | 1.70E-08 |  | -0.375 | 0.005 | 7.77 | 1.70E-08 |  | - | - | - | - |
| 3 | BovineHD0300024640 | 85756884 | 0.07 | 0.00 | 6.06 | 8.63E-07 |  | - | - | - | - |  | -0.111 | 0.002 | 6.12 | 7.65E-07 |
| 12 | BovineHD1200009199 | 31234131 | - | - | - | - |  | 0.162 | 0.002 | 7.72 | 1.89E-08 |  | 0.178 | 0.003 | 8.63 | 2.33E-09 |
| 12 | BovineHD1200009200 | 31235054 | - | - | - | - |  | 0.162 | 0.002 | 7.72 | 1.89E-08 |  | 0.178 | 0.003 | 8.63 | 2.33E-09 |
| 12 | BovineHD1200009212 | 31257193 | - | - | - | - |  | 0.162 | 0.002 | 7.54 | 2.86E-08 |  | 0.179 | 0.003 | 8.63 | 2.33E-09 |
| 12 | BovineHD4100009495 | 31246005 | - | - | - | - |  | 0.165 | 0.002 | 7.88 | 1.32E-08 |  | 0.183 | 0.003 | 8.97 | 1.08E-09 |
| 3 | BovineHD0300024340 | 84662228 | - | - | - | - |  | 0.183 | 0.003 | 8.68 | 2.09E-09 |  | 0.185 | 0.003 | 7.92 | 1.21E-08 |
| 3 | BovineHD0300027986 | 97017532 | - | - | - | - |  | 0.148 | 0.002 | 9.90 | 1.26E-10 |  | 0.177 | 0.002 | 11.91 | 1.22E-12 |
| 3 | BovineHD0300027989 | 97021182 | - | - | - | - |  | 0.149 | 0.002 | 10.20 | 6.34E-11 |  | 0.179 | 0.002 | 6.12 | 7.59E-07 |
| 3 | BovineHD0300028017 | 97127451 | - | - | - | - |  | 0.242 | 0.003 | 12.62 | 2.40E-13 |  | 0.276 | 0.003 | 8.77 | 1.70E-09 |
| 3 | BovineHD0300028020 | 97132012 | - | - | - | - |  | 0.242 | 0.003 | 12.62 | 2.40E-13 |  | 0.276 | 0.003 | 8.77 | 1.70E-09 |
| 3 | BovineHD0300028022 | 97134694 | - | - | - | - |  | 0.242 | 0.003 | 12.62 | 2.40E-13 |  | -0.386 | 0.003 | 8.77 | 1.70E-09 |
| 3 | BovineHD0300028025 | 97144061 | - | - | - | - |  | 0.173 | 0.002 | 7.36 | 4.37E-08 |  | 0.211 | 0.003 | 7.43 | 3.70E-08 |
| 3 | BovineHD0300028033 | 97182222 | - | - | - | - |  | -0.319 | 0.003 | 9.61 | 2.45E-10 |  | 0.235 | 0.004 | 9.82 | 1.52E-10 |
| 3 | BovineHD0300028185 | 97568452 | - | - | - | - |  | 0.103 | 0.002 | 6.94 | 1.16E-07 |  | 0.117 | 0.002 | 6.69 | 2.02E-07 |
| 3 | BovineHD0300028349 | 98012275 | - | - | - | - |  | -0.617 | 0.004 | 8.67 | 2.14E-09 |  | -0.571 | 0.005 | 7.80 | 1.59E-08 |
| 3 | BovineHD0300028358 | 98057144 | - | - | - | - |  | 0.240 | 0.003 | 9.37 | 4.30E-10 |  | 0.223 | 0.003 | 9.84 | 1.44E-10 |
| 3 | BovineHD0300028369 | 98100979 | - | - | - | - |  | -0.786 | 0.005 | 7.20 | 6.29E-08 |  | -0.780 | 0.006 | 11.35 | 4.51E-12 |
| 3 | BovineHD0300028381 | 98146410 | - | - | - | - |  | -0.747 | 0.004 | 11.46 | 3.48E-12 |  | -0.735 | 0.006 | 10.37 | 4.27E-11 |
| 3 | BovineHD0300028385 | 98166506 | - | - | - | - |  | -0.745 | 0.004 | 11.35 | 4.49E-12 |  | -0.730 | 0.006 | 10.23 | 5.82E-11 |
| 3 | BovineHD0300028390 | 98187368 | - | - | - | - |  | -0.744 | 0.004 | 11.30 | 4.99E-12 |  | -0.729 | 0.006 | 10.22 | 6.09E-11 |
| 3 | BovineHD0300028395 | 98200605 | - | - | - | - |  | -0.745 | 0.004 | 11.35 | 4.49E-12 |  | -0.730 | 0.006 | 10.23 | 5.82E-11 |
| 3 | BovineHD0300028396 | 98205419 | - | - | - | - |  | 0.156 | 0.002 | 7.86 | 1.37E-08 |  | 0.181 | 0.003 | 9.82 | 1.50E-10 |
| 3 | BovineHD0300028405 | 98230941 | - | - | - | - |  | 0.208 | 0.003 | 9.29 | 5.11E-10 |  | 0.186 | 0.004 | 6.43 | 3.74E-07 |
| 3 | BovineHD0300028406 | 98232293 | - | - | - | - |  | 0.146 | 0.002 | 6.54 | 2.87E-07 |  | 0.178 | 0.003 | 9.55 | 2.84E-10 |
| 3 | BovineHD0300028407 | 98234237 | - | - | - | - |  | 0.156 | 0.002 | 7.92 | 1.20E-08 |  | 0.181 | 0.003 | 9.81 | 1.55E-10 |
| 3 | BovineHD0300028408 | 98235934 | - | - | - | - |  | 0.208 | 0.003 | 9.29 | 5.11E-10 |  | 0.186 | 0.004 | 6.43 | 3.74E-07 |
| 3 | BovineHD0300028409 | 98238757 | - | - | - | - |  | 0.208 | 0.003 | 9.29 | 5.11E-10 |  | 0.186 | 0.004 | 6.43 | 3.74E-07 |
| 3 | BovineHD0300028412 | 98244243 | - | - | - | - |  | 0.208 | 0.003 | 9.29 | 5.11E-10 |  | 0.186 | 0.004 | 6.43 | 3.74E-07 |
| 3 | BovineHD0300028413 | 98245263 | - | - | - | - |  | 0.208 | 0.003 | 9.29 | 5.11E-10 |  | 0.186 | 0.004 | 6.43 | 3.74E-07 |
| 3 | BovineHD0300028667 | 99178761 | - | - | - | - |  | 0.128 | 0.002 | 7.61 | 2.45E-08 |  | 0.144 | 0.002 | 8.96 | 1.10E-09 |
| 3 | BovineHD0300028680 | 99260362 | - | - | - | - |  | 0.127 | 0.002 | 7.55 | 2.82E-08 |  | 0.144 | 0.002 | 9.07 | 8.55E-10 |
| 3 | BovineHD0300028682 | 99270414 | - | - | - | - |  | 0.122 | 0.002 | 6.61 | 2.46E-07 |  | 0.141 | 0.002 | 8.72 | 1.89E-09 |
| 3 | BTB-00147905 | 97023337 | - | - | - | - |  | 0.149 | 0.002 | 10.20 | 6.34E-11 |  | 0.179 | 0.002 | 6.12 | 7.59E-07 |
| 3 | BTB-01919893 | 97070925 | - | - | - | - |  | 0.172 | 0.002 | 7.17 | 6.75E-08 |  | 0.211 | 0.003 | 7.37 | 4.26E-08 |
| 5 | BovineHD0500029017 | 1.01E+08 | - | - | - | - |  | -0.083 | 0.001 | 6.70 | 1.98E-07 |  | -0.073 | 0.001 | 7.02 | 9.48E-08 |
| 5 | BovineHD0500029022 | 1.01E+08 | - | - | - | - |  | -0.144 | 0.002 | 7.12 | 7.67E-08 |  | -0.155 | 0.003 | 7.08 | 8.24E-08 |
| 7 | BovineHD0700005749 | 19407665 | - | - | - | - |  | 0.164 | 0.002 | 6.81 | 1.56E-07 |  | 0.077 | 0.003 | 7.88 | 1.31E-08 |
| 9 | BovineHD0900012464 | 44368160 | - | - | - | - |  | 0.092 | 0.001 | 7.84 | 1.43E-08 |  | 0.111 | 0.002 | 10.47 | 3.39E-11 |
| 9 | BovineHD0900012637 | 44965261 | - | - | - | - |  | -0.092 | 0.001 | 7.17 | 6.72E-08 |  | 0.079 | 0.002 | 6.17 | 6.82E-07 |
| 9 | BovineHD0900012643 | 44989866 | - | - | - | - |  | -0.091 | 0.001 | 7.04 | 9.10E-08 |  | 0.079 | 0.002 | 6.12 | 7.62E-07 |
| 9 | BovineHD0900012644 | 44994161 | - | - | - | - |  | -0.091 | 0.001 | 7.04 | 9.10E-08 |  | 0.079 | 0.002 | 6.12 | 7.62E-07 |
| 9 | BovineHD0900013423 | 48122653 | - | - | - | - |  | 0.233 | 0.003 | 8.14 | 7.21E-09 |  | 0.203 | 0.004 | 7.04 | 9.03E-08 |
| 9 | BovineHD0900013424 | 48125976 | - | - | - | - |  | 0.234 | 0.003 | 8.22 | 5.98E-09 |  | 0.203 | 0.004 | 7.09 | 8.21E-08 |
| 9 | BovineHD0900013427 | 48130311 | - | - | - | - |  | 0.234 | 0.003 | 8.22 | 5.98E-09 |  | 0.203 | 0.004 | 7.09 | 8.21E-08 |
| 9 | BovineHD0900013434 | 48144835 | - | - | - | - |  | 0.233 | 0.003 | 8.18 | 6.56E-09 |  | 0.204 | 0.004 | 7.13 | 7.33E-08 |
| 9 | BovineHD0900013435 | 48146895 | - | - | - | - |  | 0.250 | 0.003 | 7.47 | 3.35E-08 |  | 0.203 | 0.003 | 8.19 | 6.40E-09 |
| 9 | BovineHD4100007421 | 48133749 | - | - | - | - |  | 0.234 | 0.003 | 8.22 | 5.98E-09 |  | 0.203 | 0.004 | 7.09 | 8.21E-08 |

^1^BTA: chromosome

^2^Positions of SNPs based on ARS-UCD1.2 Bos taurus genome assembly

Table S5 - List of lethal haplotype candidates, their location, and alleles of SNPs within the putative lethal haplotypes for post-natal mortality (PNM), heifer rebreeding (HR) and stayability (STAY) in Nellore cattle

| Haplotype number | ^1^Segment | ^2^BTA | ^3^Start position | ^3^End position | Haplotypes |
| --- | --- | --- | --- | --- | --- |
| 1 | 25 | 1 | 21773327 | 22571004 | AA AA AA AA BB AA BB BB AA BB BB BB AA BB BB AA BB BB BB BB BB AA BB AA BB BB BB BB BB BB AA AA AA AA BB AA BB AA BB AA AA BB AA BB AA BB AA BB BB AA BB AA AA AA BB BB AA AA BB AA BB BB BB BB BB BB BB AA BB BB BB BB AA AA BB AA AA BB BB BB BB AA AA BB BB BB BB BB BB BB BB AA BB BB AA AA AA BB AA BB AA AA BB BB BB AA AA BB BB BB AA AA AA AA BB AA AA BB BB AA BB AA BB BB AA BB BB BB BB BB AA AA AA BB BB BB BB AA BB AA AA AA BB AA AA BB BB AA BB AA AA AA BB AA BB BB BB AA BB BB BB AA BB AA BB AA BB BB BB AA AA AA BB BB AA AA BB AA AA BB BB AA AA BB BB AA BB BB BB BB BB BB BB AA BB BB BB AA BB |
| 7 | 108 | 1 | 92201954 | 93179359 | AA BB BB BB BB AA BB BB AA AA BB BB BB BB BB BB AA AA AA BB AA BB BB BB AA AA BB BB BB AA BB AA BB AA AA BB AA BB AA AA BB BB AA AA AA BB BB AA BB AA BB BB BB AA BB BB AA BB AA BB BB BB AA AA AA AA BB AA BB BB BB BB AA BB BB AA BB BB AA AA AA AA AA AA AA AA BB AA AA BB BB AA AA AA BB AA BB BB AA AA BB AA AA AA BB BB BB AA AA AA BB BB BB BB BB BB AA BB AA AA AA AA AA BB BB AA BB AA BB BB BB AA AA BB AA BB BB AA BB BB BB BB BB BB AA AA AA BB AA BB BB BB BB BB BB BB AA BB BB AA AA BB AA AA BB AA AA AA BB AA AA BB BB BB BB BB BB BB AA BB AA BB BB BB BB AA AA AA BB BB BB AA AA AA AA BB AA BB BB |
| 2 | 346 | 2 | 30989280 | 31660659 | AA AA BB AA BB AA BB AA BB BB BB AA BB BB BB BB BB BB BB BB AA BB AA BB AA BB BB BB BB BB AA BB BB BB BB AA BB AA BB BB BB AA AA BB BB BB AA AA BB BB AA BB BB BB AA BB AA AA AA AA BB BB AA AA AA BB AA AA BB AA AA BB BB AA BB BB AA AA BB BB AA BB BB BB BB AA AA BB BB BB BB BB AA AA BB AA AA BB BB AA AA BB BB AA AA BB BB AA BB BB BB BB BB AA BB AA AA BB BB AA BB BB BB BB BB BB AA BB BB BB AA BB AA BB BB AA AA AA AA AA BB BB BB BB BB BB BB BB BB AA BB AA BB AA BB BB BB AA BB BB BB AA AA BB AA AA BB BB AA AA AA AA BB AA BB BB AA AA BB AA AA BB AA AA BB BB BB BB BB AA BB AA AA BB BB BB BB BB AA |
| 4 | 231 | 2 | 125601735 | 126602788 | AA BB AA BB AA AA AA AA BB BB BB BB BB BB AA BB AA AA BB AA AA AA AA BB AA AA BB AA BB AA BB BB AA BB AA AA AA BB AA BB AA BB AA BB AA BB BB AA AA AA AA AA AA BB AA AA AA AA AA BB AA AA AA BB AA BB BB BB AA AA BB AA AA BB BB AA AA BB AA AA BB BB AA AA BB BB BB BB BB AA BB BB BB BB AA BB BB AA AA AA AA AA AA AA AA BB AA BB AA BB AA AA BB BB AA BB BB AA AA AA AA AA BB BB AA AA AA AA BB AA AA AA AA BB BB BB AA BB BB AA AA BB AA BB AA BB BB BB BB BB BB AA AA BB AA AA BB BB AA BB BB AA AA BB AA BB AA AA AA BB AA BB BB BB AA BB AA AA AA BB BB AA AA AA BB BB BB BB BB BB AA BB AA AA BB AA BB BB BB |
| 2 | 479 | 3 | 27103538 | 27639143 | AA BB BB BB BB BB AA AA AA BB BB AA BB AA AA BB BB BB BB BB BB BB AA BB BB BB AA AA BB BB AA AA AA AA BB AA BB AA AA AA BB AA AA BB BB BB BB BB AA AA AA AA BB AA BB AA BB AA BB BB BB BB AA AA AA AA AA AA BB BB AA AA AA BB AA AA BB BB AA AA AA BB BB BB BB BB BB BB BB AA BB BB BB BB BB BB AA AA AA AA AA BB AA BB BB BB BB BB BB BB AA BB BB BB BB AA AA BB BB BB AA AA AA BB AA BB AA AA BB AA BB BB AA AA AA AA AA AA BB AA BB AA AA AA BB AA AA AA AA BB BB AA BB BB AA BB BB AA BB AA BB BB BB AA BB BB BB AA BB BB AA BB BB BB BB AA AA BB BB BB AA BB AA BB AA BB AA AA AA BB AA BB AA AA BB BB BB BB BB AA |
| 34 | 396 | 3 | 84166546 | 84882788 | BB BB BB AA BB BB BB AA BB BB BB BB BB AA BB AA BB BB BB BB AA BB AA BB BB BB BB BB AA BB AA BB BB AA BB AA BB BB BB BB AA AA BB AA AA BB BB AA AA BB BB BB BB BB BB BB BB BB BB AA AA BB BB BB BB BB AA BB AA BB BB BB AA BB AA AA BB BB BB AA BB BB BB BB BB BB AA BB BB BB AA AA AA BB AA AA BB BB BB BB BB AA BB BB AA AA AA BB BB AA BB AA BB AA AA AA AA AA BB AA AA BB BB BB BB AA BB AA AA AA AA BB AA AA AA AA AA AA BB AA BB AA BB BB AA BB AA BB BB BB BB BB BB BB AA AA AA AA AA BB AA AA BB BB AA AA AA BB AA BB BB BB BB BB AA BB AA AA AA BB BB AA BB AA BB BB AA BB AA AA AA AA BB AA BB BB AA BB AA |
| 4 | 461 | 3 | 97862526 | 98537136 | AA BB BB BB BB BB BB AA BB AA AA BB BB AA AA BB BB AA AA BB BB AA AA BB BB BB AA BB AA BB AA BB AA AA BB BB AA BB BB AA AA BB BB BB BB AA AA AA AA AA BB AA BB AA AA BB BB AA BB BB AA BB BB AA BB BB AA BB BB AA BB AA AA BB BB BB AA AA BB AA AA AA AA AA AA BB BB AA AA BB AA BB AA BB AA AA BB AA BB BB BB BB AA AA 111BB BB AA BB BB AA AA BB AA BB AA BB BB AA AA BB AA BB BB AA BB BB BB AA AA AA BB AA AA BB BB AA BB AA AA BB BB BB AA BB AA AA BB BB AA AA BB BB BB BB BB BB BB AA BB BB BB AA AA BB BB AA AA AA AA AA BB AA AA BB BB AA AA BB AA BB BB AA AA AA BB BB BB AA BB AA BB BB AA BB BB AA AA |
| 135 | 676 | 5 | 19466593 | 20216556 | AA AA AA BB BB AA AA AA BB BB BB AA AA AA BB AA AA BB AA BB AA AA BB AA AA BB BB AA BB BB BB AA BB AA BB AA BB BB AA AA AA BB AA BB BB AA AA AA AA AA BB AA AA AA BB AA BB BB BB AA AA BB AA BB BB BB AA BB AA AA BB BB AA BB BB BB AA BB AA AA AA BB BB BB BB AA AA AA AA BB AA BB AA BB BB BB BB BB BB BB AA AA AA BB BB AA BB BB BB AA AA AA BB BB BB BB AA AA BB BB AA BB AA AA AA BB AA BB AA BB AA AA AA BB BB AA AA AA BB AA BB BB AA AA BB BB AA AA BB BB AA AA BB BB BB BB AA BB BB BB BB BB BB AA BB BB BB AA AA AA BB AA AA BB AA AA AA AA BB AA BB AA AA BB AA BB BB BB AA AA AA AA AA BB AA BB BB AA BB BB |
| 3 | 769 | 5 | 99424373 | 100174866 | BB BB AA BB BB BB BB AA BB BB BB BB BB BB AA AA BB BB BB BB AA BB AA BB AA AA BB BB BB BB BB AA BB AA AA BB BB AA AA AA AA AA AA BB AA BB BB AA BB BB AA AA AA AA BB BB BB BB BB AA AA BB BB BB BB BB AA AA AA AA AA BB BB BB AA BB AA AA BB BB AA AA AA AA AA AA BB AA BB AA AA AA BB AA BB BB BB BB BB BB BB BB BB BB BB BB AA AA AA BB BB BB BB BB BB BB AA BB BB BB AA BB BB AA AA BB BB BB AA AA AA AA AA AA AA BB AA AA BB AA BB BB BB AA BB BB BB AA BB AA AA AA BB BB AA AA BB BB BB BB AA AA BB BB BB BB BB BB AA AA BB BB AA BB BB BB BB AA BB BB BB BB AA BB BB AA AA BB AA AA BB AA AA AA AA AA BB BB BB BB |
| 1 | 1077 | 7 | 19468345 | 20417099 | AA BB BB BB BB AA AA BB BB AA AA AA AA BB AA AA BB AA BB AA BB BB BB BB AA AA BB AA AA BB AA BB BB AA BB AA AA BB AA BB AA AA BB BB BB AA BB AA BB BB AA BB BB AA AA BB BB BB AA BB AA BB BB AA BB BB AA AA AA BB BB AA AA BB AA AA BB AA BB AA BB BB BB AA BB AA BB BB AA AA BB AA AA AA BB BB BB BB AA BB BB BB BB BB BB BB BB BB AA AA AA BB BB BB BB BB AA AA AA AA BB BB BB BB AA AA BB AA BB BB BB AA AA BB BB BB BB BB BB BB BB BB BB AA AA AA BB BB BB AA BB AA BB AA BB AA AA AA AA AA BB BB AA AA BB BB AA AA BB AA AA AA AA AA BB AA AA BB BB AA AA AA BB BB AA BB BB BB BB BB AA AA BB AA BB BB BB AA BB AA |
| 14 | 988 | 7 | 36554986 | 37251910 | BB AA AA BB AA BB BB BB BB BB BB BB AA BB AA AA BB BB AA AA AA BB AA BB BB BB BB BB AA BB BB AA BB AA AA BB AA BB AA BB AA AA AA BB BB AA BB AA BB AA AA BB BB BB BB AA BB BB AA AA BB BB AA BB AA AA BB BB AA BB AA BB AA AA BB AA BB BB BB BB AA AA BB BB BB AA BB AA BB AA BB AA AA AA BB BB AA BB BB AA BB AA AA BB BB BB BB BB AA AA AA BB AA AA AA AA AA AA BB BB BB BB AA AA AA AA BB AA BB BB AA AA AA AA AA BB BB BB BB AA AA BB BB AA BB BB AA AA BB AA BB AA AA AA AA AA AA BB AA BB BB BB BB BB AA BB BB AA BB BB BB AA BB BB BB BB BB BB AA AA BB BB BB AA AA AA BB AA BB BB AA AA AA AA BB BB AA BB BB AA |
| 2 | 966 | 7 | 80932886 | 81622237 | BB AA BB BB AA AA AA AA AA AA BB BB BB BB AA BB BB AA AA BB AA BB AA BB AA BB BB BB BB BB BB BB BB BB BB BB BB BB BB BB BB BB BB BB BB BB BB AA AA BB BB BB BB BB BB BB AA AA AA AA BB AA BB BB BB AA BB BB BB AA BB BB BB BB BB BB BB BB BB AA BB AA BB BB BB BB BB BB BB BB BB AA AA AA BB AA BB AA BB BB BB AA AA BB BB AA AA AA BB AA BB BB AA BB AA BB AA BB AA BB AA BB AA BB AA BB BB AA BB AA BB BB BB BB AA BB BB AA AA BB BB AA BB AA BB BB AA BB AA BB AA BB BB AA BB BB BB AA AA AA BB AA AA AA BB BB BB BB BB BB AA AA BB AA AA AA AA AA AA AA AA BB AA BB AA AA AA AA BB BB AA AA BB BB AA AA AA AA BB |
| 24 | 1045 | 7 | 106846493 | 107525464 | AA AA BB AA AA BB BB AA AA AA BB AA AA AA BB BB AA AA AA BB BB AA AA AA AA AA BB AA AA BB BB AA AA AA BB BB BB BB BB AA AA AA AA AA BB AA BB BB AA BB AA AA AA AA AA BB AA BB BB BB BB BB AA AA BB BB AA AA AA BB BB BB AA BB AA BB BB BB BB AA BB BB BB BB BB AA BB AA AA BB BB BB AA AA AA BB AA BB AA BB BB AA AA AA BB AA BB BB AA AA AA AA AA AA AA AA BB AA AA BB BB AA AA BB BB BB BB BB AA AA AA AA BB BB AA AA AA BB AA BB BB AA BB BB AA AA BB BB AA AA AA AA AA AA BB AA AA AA BB AA AA AA AA BB BB AA AA BB AA AA BB AA AA AA BB AA AA BB AA BB AA BB BB AA AA AA AA BB BB BB AA BB AA BB BB BB AA BB BB AA |
| 1 | 1184 | 8 | 78277928 | 79262115 | BB BB AA BB BB AA BB BB BB BB AA AA BB BB AA BB BB BB BB AA AA AA AA AA AA BB BB AA BB BB BB AA BB BB AA AA BB AA AA AA BB BB AA BB BB BB AA AA BB AA BB BB BB BB BB BB BB AA BB BB BB AA BB AA BB BB BB BB BB BB BB BB BB AA BB AA AA AA BB AA BB BB AA AA AA BB AA BB AA BB AA AA AA AA BB BB AA BB AA BB AA AA AA BB AA AA AA BB BB AA BB BB BB BB BB BB AA AA AA BB AA BB BB BB AA BB BB BB AA AA BB BB AA AA BB BB AA BB BB AA BB AA BB BB BB BB BB AA AA AA BB BB AA BB AA AA AA BB AA AA AA BB BB BB BB AA AA AA AA AA AA AA AA AA BB AA BB BB BB AA AA AA AA BB AA BB BB AA AA BB BB BB BB BB AA AA AA AA AA |
| 1 | 1192 | 8 | 84943889 | 85809360 | AA BB BB BB AA AA BB BB BB BB AA BB AA AA AA AA AA BB AA AA AA BB BB BB BB BB BB AA BB BB AA BB BB BB BB AA BB BB AA BB AA BB BB BB AA BB AA AA AA AA BB AA AA BB BB BB BB BB BB BB AA BB BB AA AA BB BB BB BB AA BB BB AA AA AA BB AA AA BB AA BB BB BB AA BB AA BB AA AA BB BB BB AA BB BB AA BB AA AA BB BB BB BB BB BB BB AA AA BB BB BB AA AA BB BB BB BB BB BB AA AA BB AA BB BB BB BB BB BB AA BB AA BB BB AA AA BB BB BB BB BB AA AA AA BB BB BB BB BB BB BB BB AA BB AA BB BB BB AA AA BB AA AA AA AA AA BB AA BB BB BB BB BB BB BB AA AA AA BB BB BB AA BB BB BB BB BB BB BB BB BB AA BB BB BB BB BB BB AA BB |
| 303 | 1277 | 9 | 42710447 | 43514053 | AA AA AA BB AA AA BB BB BB AA BB BB BB AA AA AA BB BB BB BB BB AA BB BB BB AA BB AA BB AA AA BB AA BB AA AA AA AA BB AA BB AA BB BB AA BB AA AA BB BB BB BB AA BB BB AA BB AA AA BB AA BB AA BB BB AA AA BB AA AA AA AA AA BB AA AA BB BB AA AA AA AA AA BB BB AA BB BB BB AA AA AA AA BB BB BB BB BB BB BB AA BB BB BB AA BB BB BB BB BB BB BB BB AA AA BB AA BB BB BB AA BB AA BB AA BB BB AA BB AA BB BB BB BB AA BB AA BB BB AA BB AA AA BB BB BB AA BB BB AA AA BB AA BB BB BB AA BB BB AA AA AA AA AA BB BB AA BB AA BB AA BB AA AA BB AA BB BB BB BB BB BB AA BB AA BB BB BB AA BB BB AA AA AA AA BB AA AA AA |
| 33 | 1283 | 9 | 43515752 | 44346392 | AA BB BB BB BB AA BB BB BB BB BB BB BB BB AA AA BB AA AA BB AA AA AA BB AA AA AA AA AA AA BB BB AA AA AA AA BB AA BB BB BB BB BB BB BB BB AA BB AA BB BB AA BB AA AA AA BB AA BB AA AA BB BB AA AA BB BB AA AA BB BB AA BB BB AA BB AA AA AA BB AA AA BB AA AA AA BB BB AA AA AA AA AA BB AA BB BB AA BB AA BB AA BB BB BB BB BB AA AA AA AA BB BB BB AA AA AA AA BB AA AA AA BB AA BB BB BB BB BB AA BB BB BB AA BB BB BB BB BB BB BB BB AA BB BB BB BB BB BB BB AA AA BB BB BB BB AA AA AA BB BB BB AA BB BB BB BB AA BB AA AA AA BB AA AA BB BB BB BB BB BB BB AA AA AA BB BB AA BB BB AA BB BB AA BB AA BB BB AA BB |
| 37 | 1278 | 9 | 48144835 | 48978466 | BB AA BB BB AA BB BB BB BB AA AA BB BB BB AA AA BB BB AA BB BB AA BB AA BB BB BB BB AA BB BB BB AA BB BB BB AA AA AA BB BB AA AA AA AA AA AA AA AA AA AA BB AA BB BB BB BB BB BB AA BB AA AA BB AA BB BB AA AA AA BB BB AA AA AA BB BB AA AA BB AA BB BB AA BB BB AA AA AA AA BB AA BB AA AA AA AA AA AA BB BB BB BB AA BB AA AA AA AA AA AA BB BB AA AA BB BB BB AA AA BB AA BB AA BB AA AA BB BB BB BB AA AA BB BB BB AA AA BB AA BB AA AA AA BB AA BB BB BB AA BB AA AA BB AA AA BB BB AA AA BB AA AA BB BB AA AA BB AA AA AA BB BB BB AA BB AA BB AA AA AA AA BB BB AA AA AA BB AA BB BB BB AA AA BB BB AA BB BB |
| 67 | 1312 | 9 | 72521066 | 73292804 | AA AA AA AA BB BB BB AA BB AA BB BB AA BB BB AA BB BB BB AA AA AA BB AA AA BB BB BB BB BB BB BB AA BB AA AA BB AA BB BB AA BB AA AA BB BB BB BB AA AA AA BB AA BB BB BB AA BB BB AA BB AA BB BB BB BB AA AA AA AA BB BB BB AA AA AA AA AA BB AA BB AA BB AA BB BB BB AA BB AA BB AA AA AA AA BB AA BB BB BB AA AA BB AA AA BB AA AA BB BB AA BB BB BB AA BB BB AA BB AA AA BB BB AA BB BB AA AA AA AA BB BB BB AA AA AA BB BB AA BB AA BB AA BB BB AA AA BB BB BB BB AA AA AA AA BB AA BB AA BB AA AA BB AA AA AA BB BB BB BB AA BB AA AA BB BB BB BB BB AA AA AA BB AA BB BB BB AA AA AA AA BB BB BB AA BB BB BB BB BB |
| 2 | 1457 | 10 | 85722335 | 86530381 | BB BB BB AA BB BB AA BB BB BB BB AA BB AA BB AA BB BB AA AA BB BB BB BB BB BB AA BB BB AA AA BB BB AA BB BB BB AA BB BB BB BB BB AA BB AA BB AA BB BB AA AA BB AA BB BB BB BB BB BB BB BB BB BB BB BB BB AA AA AA BB AA AA AA BB BB BB BB AA AA AA AA AA BB AA AA BB AA BB BB AA BB AA AA BB AA AA BB BB AA BB BB BB AA AA BB BB BB BB BB BB BB BB BB BB AA BB AA AA BB BB BB AA AA BB AA AA BB AA BB AA BB AA BB AA AA BB AA BB BB BB BB AA AA BB AA BB BB AA BB AA AA AA BB AA AA AA BB BB BB BB AA BB BB BB BB BB BB BB BB BB BB BB BB BB BB BB AA BB BB BB BB BB AA AA AA BB BB AA BB AA AA AA BB AA BB BB BB BB BB |
| 2 | 1655 | 12 | 31305864 | 32200052 | AA BB BB AA BB BB BB BB AA BB BB AA BB BB BB BB AA BB BB BB AA AA BB AA AA AA BB BB BB AA AA AA AA AA BB BB AA AA AA BB AA AA AA BB BB BB AA AA BB BB BB BB AA BB AA BB AA AA BB BB BB AA BB AA BB AA AA BB BB AA BB BB BB BB AA BB BB BB AA BB BB AA AA AA AA AA AA BB BB BB AA BB AA AA AA BB AA BB AA AA AA AA BB AA AA AA AA BB BB BB BB BB BB BB AA AA BB AA AA BB BB BB AA AA AA AA AA AA BB AA AA AA BB AA AA BB BB AA BB BB BB BB BB AA BB AA BB AA BB BB BB BB BB BB BB BB AA BB BB BB AA BB AA AA AA BB AA AA BB AA BB AA AA BB AA AA BB AA BB AA BB BB AA BB AA BB BB AA BB BB BB BB BB BB BB BB |
| 8 | 2213 | 17 | 65952250 | 66468921 | BB BB BB AA BB AA AA AA AA AA BB AA BB AA BB BB BB BB BB AA BB BB BB AA BB BB BB BB AA BB BB AA AA BB AA AA BB BB BB BB BB BB AA AA BB AA AA BB BB BB BB BB BB AA AA AA BB BB BB AA BB AA AA BB BB BB BB BB BB AA BB AA AA BB BB AA BB BB AA BB AA AA AA AA BB BB BB BB BB BB BB BB AA BB BB BB BB BB BB BB BB BB AA BB AA AA BB BB BB BB AA BB BB AA BB AA AA AA BB AA AA AA BB BB AA BB BB BB BB BB BB BB BB BB BB BB AA BB BB BB BB BB AA AA AA BB AA BB BB AA AA BB BB AA AA AA BB BB BB BB BB AA BB BB BB BB BB BB BB AA BB BB BB BB BB AA BB BB BB BB BB AA BB AA BB BB AA BB BB BB AA AA AA BB BB BB BB BB AA BB |
| 1 | 2237 | 18 | 9785118 | 10380109 | BB BB BB BB AA AA BB BB BB BB BB AA BB AA AA AA AA BB BB BB AA BB BB AA BB AA BB BB BB BB BB AA BB BB BB AA BB BB AA BB BB BB AA BB BB BB BB BB BB BB BB BB BB BB BB BB AA BB BB BB BB AA BB BB AA AA BB BB AA AA BB BB BB BB AA BB BB BB AA BB AA AA BB BB BB BB BB AA AA BB AA AA BB BB BB AA BB AA BB BB BB AA BB AA AA BB AA AA BB AA BB AA BB BB AA BB AA AA AA BB AA AA BB BB BB BB BB BB AA BB BB BB AA AA BB BB AA BB BB AA BB BB AA AA BB AA BB BB AA AA BB BB AA BB AA AA BB BB AA BB AA BB BB AA BB BB BB AA AA BB BB BB BB BB BB BB AA BB BB BB AA BB AA AA BB AA BB BB AA BB AA BB AA BB BB BB |
| 1 | 2270 | 18 | 36265657 | 37406377 | BB AA BB BB BB BB AA BB BB BB AA AA BB BB BB BB BB BB AA AA BB BB AA BB AA BB BB BB BB BB BB AA BB AA AA AA AA BB AA AA BB BB AA AA BB BB AA BB BB BB BB AA BB BB BB AA BB BB AA BB BB BB AA BB AA AA AA BB BB BB AA AA BB AA AA AA AA BB BB BB BB AA BB AA BB AA AA AA BB AA BB BB AA AA AA BB AA BB BB AA BB AA BB AA AA BB AA BB AA BB AA BB AA BB BB BB AA BB AA AA BB BB AA BB AA BB BB AA BB AA AA BB BB BB BB AA BB BB BB BB BB AA BB AA BB AA AA BB BB AA BB BB BB BB AA BB AA AA AA BB BB AA BB BB AA AA AA AA AA BB AA AA AA AA AA BB BB AA BB BB BB AA AA AA BB BB AA AA AA AA BB BB BB BB BB AA |
| 1 | 2329 | 19 | 20292526 | 21133267 | BB BB AA BB BB BB BB BB BB BB BB AA BB BB BB AA BB BB BB AA BB BB BB AA BB AA AA AA AA AA BB AA AA AA BB BB AA BB AA BB AA BB BB BB BB BB AA AA AA BB AA AA BB BB AA AA BB BB BB AA AA BB BB AA BB BB BB BB BB BB BB BB BB AA BB BB BB BB BB BB BB BB AA BB BB BB AA BB BB BB BB AA BB AA BB BB AA BB BB BB AA AA AA BB BB BB AA AA AA BB BB AA AA BB BB AA AA BB BB BB AA AA AA BB AA AA AA BB BB AA AA BB BB AA BB AA AA AA AA AA BB BB BB BB BB BB BB BB BB AA BB AA AA AA BB BB BB BB AA AA BB BB BB BB AA BB AA BB AA BB BB AA AA AA BB BB AA AA BB BB BB BB BB BB BB BB BB AA BB BB BB BB AA BB AA BB AA AA |
| 1 | 2371 | 19 | 54651560 | 55237521 | AA AA BB AA BB AA AA BB AA BB AA AA AA BB AA AA BB BB BB BB AA AA BB BB AA AA BB BB AA AA AA AA BB BB AA AA BB BB BB AA AA BB AA BB BB AA BB BB BB AA BB BB BB AA BB BB AA AA BB AA AA AA AA AA BB AA BB BB AA BB AA AA BB AA BB BB AA BB BB BB BB AA BB AA BB AA AA BB BB BB AA AA BB AA AA BB BB AA BB BB AA AA AA AA AA AA BB AA BB AA AA BB BB BB BB BB BB BB BB AA AA AA AA BB AA AA AA AA BB AA BB BB AA BB AA BB BB AA AA AA BB BB BB BB BB AA AA BB AA BB BB AA AA AA BB BB BB BB AA BB BB BB AA AA BB AA BB BB BB BB BB BB AA BB BB AA BB BB BB AA BB BB AA BB AA BB BB AA BB BB AA BB BB BB BB AA BB |
| 22 | 2620 | 22 | 18857788 | 19688790 | AA BB AA BB BB BB BB AA BB AA BB AA AA AA BB AA BB AA BB AA BB BB BB BB AA BB BB BB BB AA AA BB BB BB BB BB AA AA BB BB BB BB BB AA AA BB AA BB AA BB BB BB AA AA AA AA BB BB AA BB AA BB AA AA AA BB AA BB BB BB BB AA BB BB BB BB AA BB AA AA AA BB AA BB BB AA BB BB AA AA BB AA BB AA BB AA BB BB BB AA BB BB BB BB BB BB AA AA BB BB AA BB BB AA AA BB BB AA AA BB BB BB AA BB AA BB AA AA AA BB BB BB BB AA AA AA AA BB BB BB BB BB AA AA AA BB BB AA BB BB BB BB BB BB BB AA BB BB BB AA AA AA AA BB BB BB BB BB BB BB BB BB AA BB BB BB BB AA BB AA AA BB BB BB BB BB BB AA BB BB AA AA AA BB AA BB BB |
| 3 | 2585 | 22 | 46693982 | 47385511 | AA AA BB BB AA BB AA BB AA AA AA BB BB BB AA AA BB AA AA AA BB AA BB AA AA AA BB BB BB AA AA BB AA BB BB AA AA AA AA BB AA AA AA BB AA AA BB BB AA AA BB AA BB AA BB BB BB BB AA BB AA BB AA AA BB BB BB AA BB BB BB AA AA BB AA BB AA BB BB AA BB BB AA BB AA AA BB AA AA AA BB BB BB AA BB BB AA AA AA AA BB AA BB BB AA BB BB AA BB AA BB AA AA BB BB BB BB BB AA BB AA BB BB BB AA AA BB AA AA AA AA AA AA BB BB AA BB AA BB AA AA AA BB AA BB BB AA AA BB BB AA BB BB BB BB BB AA BB BB BB BB AA BB BB BB BB BB AA BB BB AA BB AA BB AA BB BB AA AA BB BB BB BB BB BB AA BB AA BB AA BB BB BB BB BB BB AA |
| 2 | 2724 | 24 | 18329342 | 19105911 | BB BB AA BB AA BB BB AA BB AA BB AA BB AA AA BB AA AA BB AA AA AA BB AA AA AA AA BB BB BB BB BB AA AA BB BB BB BB AA AA AA BB BB BB AA BB BB AA BB BB BB BB AA AA BB AA AA AA BB AA BB AA BB AA AA BB AA BB BB BB BB BB BB AA BB AA BB AA BB AA BB BB AA BB BB AA AA BB AA BB BB BB AA BB BB BB BB BB BB AA BB AA BB AA BB BB BB AA BB BB AA BB AA AA BB BB BB AA BB AA BB BB BB BB AA AA BB AA BB AA BB AA AA BB BB AA BB AA BB BB AA AA BB BB BB AA AA BB BB AA AA BB AA AA BB AA BB AA AA BB BB AA AA AA AA AA AA AA AA AA AA BB AA AA BB AA AA AA BB BB BB BB BB BB AA AA AA AA BB AA AA AA BB AA AA AA AA AA |
| 3 | 3063 | 29 | 40482004 | 41207696 | AA BB BB BB AA AA AA BB BB AA BB AA AA BB BB AA BB BB BB BB BB AA AA BB AA BB AA AA BB BB AA BB AA AA AA BB BB AA BB BB BB AA BB BB BB AA BB BB BB AA BB BB BB BB AA BB BB BB BB BB BB BB BB AA BB BB AA BB AA AA AA AA BB BB BB AA BB BB BB AA BB BB AA AA BB BB BB AA AA AA BB AA BB AA BB BB AA BB AA AA BB AA AA AA BB BB BB AA AA AA BB BB AA BB BB AA AA BB AA AA BB BB AA BB BB BB AA AA AA AA BB AA BB BB AA BB AA AA BB BB AA BB AA BB AA AA BB BB AA BB AA AA BB BB AA BB AA AA AA BB BB BB BB BB BB AA BB AA AA BB BB BB BB AA BB BB BB AA AA BB BB AA AA AA AA BB AA AA BB BB BB BB BB AA BB BB BB BB BB |

^1^Segment: a group of consecutive SNPs

^2^Chromosome

^3^Positions based on ARS-UCD1.2 Bos taurus genome assembly

Table S6 – Chromosome (BTA) and identification of genes (start and end position, name, type, ID and description) for heifer rebreeding (HR) of Nellore females

| BTA | ^1^Gene start | ^1^Gene end | Gene name | Gene type | ^2^Gene stable ID version | ^3^Gene description |
| --- | --- | --- | --- | --- | --- | --- |
| 1 | 21,167,188 | 21,328,190 | USP25 | protein_coding | ENSBTAG00000019314.5 | ubiquitin specific peptidase 25 [Source:VGNC Symbol;Acc:VGNC:36718] |
| 1 | 93,147,160 | 93,897,117 | NLGN1 | protein_coding | ENSBTAG00000031891.4 | neuroligin 1 [Source:VGNC Symbol;Acc:VGNC:58395] |
| 3 | 83,556,891 | 83,936,160 | PATJ | protein_coding | ENSBTAG00000021975.6 | PATJ crumbs cell polarity complex component [Source:VGNC Symbol;Acc:VGNC:32592] |
| 3 | 84,203,809 | 84,620,790 | NFIA | protein_coding | ENSBTAG00000000074.5 | nuclear factor I A [Source:VGNC Symbol;Acc:VGNC:32038] |
| 3 | 97,468,115 | 97,518,125 | BEND5 | protein_coding | ENSBTAG00000009209.5 | BEN domain containing 5 [Source:VGNC Symbol;Acc:VGNC:26468] |
| 3 | 97,776,435 | 97,942,867 | SPATA6 | protein_coding | ENSBTAG00000003128.6 | spermatosis associated 6 [Source:VGNC Symbol;Acc:VGNC:35189] |
| 3 | 97,963,204 | 98,001,735 | SLC5A9 | protein_coding | ENSBTAG00000014225.6 | solute carrier family 5 member 9 [Source:VGNC Symbol;Acc:VGNC:34912] |
| 3 | 98,066,623 | 98,090,010 | SKINT1 | protein_coding | ENSBTAG00000009192.6 | selection and upkeep of intraepithelial T cells 1 [Source:NCBI gene (formerly Entrezgene);Acc:528184] |
| 3 | 98,264,664 | 98,264,770 | U6 | snRNA | ENSBTAG00000050751.1 | U6 spliceosomal RNA [Source:RFAM;Acc:RF00026] |
| 3 | 98,278,655 | 98,494,949 | TRABD2B | protein_coding | ENSBTAG00000054324.1 | TraB domain containing 2B [Source:VGNC Symbol;Acc:VGNC:106996] |
| 3 | 99,069,008 | 99,097,597 | CYP4X1 | protein_coding | ENSBTAG00000015647.6 | cytochrome P450 family 4 subfamily X member 1 [Source:HGNC Symbol;Acc:HGNC:20244] |
| 3 | 99,145,033 | 99,145,170 | 5S_rRNA | rRNA | ENSBTAG00000028857.2 | 5S ribosomal RNA [Source:RFAM;Acc:RF00001] |
| 3 | 99,208,577 | 99,222,625 | CYP4A11 | protein_coding | ENSBTAG00000037890.3 | cytochrome P450, family 4, subfamily A, polypeptide 11 [Source:NCBI gene (formerly Entrezgene);Acc:511890] |
| 3 | 99,319,612 | 99,334,690 | CYP4A22 | protein_coding | ENSBTAG00000013481.6 | cytochrome P450, family 4, subfamily A, polypeptide 22 [Source:NCBI gene (formerly Entrezgene);Acc:516085] |
| 3 | 99,337,603 | 99,358,612 | CYP4B1 | protein_coding | ENSBTAG00000011976.5 | cytochrome P450, family 4, subfamily B, polypeptide 1 [Source:NCBI gene (formerly Entrezgene);Acc:540149] |
| 3 | 99,421,569 | 99,457,891 | EFCAB14 | protein_coding | ENSBTAG00000000097.4 | EF-hand calcium binding domain 14 [Source:VGNC Symbol;Acc:VGNC:28342] |
| 5 | 100,504,892 | 100,630,214 | KLRB1 | protein_coding | ENSBTAG00000012029.6 | killer cell lectin like receptor B1 [Source:VGNC Symbol;Acc:VGNC:30684] |
| 5 | 100,679,985 | 100,732,005 | OVOS2 | protein_coding | ENSBTAG00000005243.6 | ovostatin 2 [Source:NCBI gene (formerly Entrezgene);Acc:508026] |
| 5 | 100,873,612 | 100,891,039 | A2M | protein_coding | ENSBTAG00000018137.6 | alpha-2-macroglobulin [Source:VGNC Symbol;Acc:VGNC:55837] |
| 5 | 100,946,808 | 100,964,339 | KLRG1 | protein_coding | ENSBTAG00000013640.5 | killer cell lectin like receptor G1 [Source:VGNC Symbol;Acc:VGNC:30687] |
| 5 | 100,984,954 | 100,998,031 | M6PR | protein_coding | ENSBTAG00000018207.4 | mannose-6-phosphate receptor, cation dependent [Source:VGNC Symbol;Acc:VGNC:31125] |
| 5 | 100,997,211 | 101,017,135 | PHC1 | protein_coding | ENSBTAG00000018205.6 | polyhomeotic homolog 1 [Source:VGNC Symbol;Acc:VGNC:32810] |
| 5 | 101,041,433 | 101,090,609 | A2ML1 | protein_coding | ENSBTAG00000004097.6 | alpha-2-macroglobulin like 1 [Source:VGNC Symbol;Acc:VGNC:25435] |
| 7 | 19,223,877 | 19,257,053 | UHRF1 | protein_coding | ENSBTAG00000002224.6 | ubiquitin like with PHD and ring finger domains 1 [Source:VGNC Symbol;Acc:VGNC:36654] |
| 7 | 19,261,039 | 19,274,362 | ARRDC5 | protein_coding | ENSBTAG00000031673.2 | arrestin domain containing 5 [Source:VGNC Symbol;Acc:VGNC:26175] |
| 7 | 19,291,174 | 19,311,897 | PLIN3 | protein_coding | ENSBTAG00000011805.6 | perilipin 3 [Source:VGNC Symbol;Acc:VGNC:33031] |
| 7 | 19,335,555 | 19,337,855 | TICAM1 | pseudogene | ENSBTAG00000019966.6 | toll like receptor adaptor molecule 1 [Source:VGNC Symbol;Acc:VGNC:52887] |
| 7 | 19,353,553 | 19,357,132 | FEM1A | protein_coding | ENSBTAG00000003476.6 | fem-1 homolog A [Source:VGNC Symbol;Acc:VGNC:28944] |
| 7 | 19,372,417 | 19,372,498 | bta-mir-7-3 | miRNA | ENSBTAG00000029968.2 | bta-mir-7-3 [Source:miRBase;Acc:MI0005056] |
| 7 | 19,428,848 | 19,469,011 | DPP9 | protein_coding | ENSBTAG00000021134.6 | dipeptidyl peptidase 9 [Source:VGNC Symbol;Acc:VGNC:28190] |
| 7 | 19,470,114 | 19,482,055 | MYDGF | protein_coding | ENSBTAG00000018655.4 | myeloid derived growth factor [Source:VGNC Symbol;Acc:VGNC:31790] |
| 7 | 19,483,938 | 19,503,011 | TNFAIP8L1 | protein_coding | ENSBTAG00000037765.3 | TNF alpha induced protein 8 like 1 [Source:VGNC Symbol;Acc:VGNC:36158] |
| 8 | 79,167,865 | 79,255,810 | NAA35 | protein_coding | ENSBTAG00000001016.6 | N-alpha-acetyltransferase 35, NatC auxiliary subunit [Source:VGNC Symbol;Acc:VGNC:56977] |
| 8 | 79,263,894 | 79,310,527 | GOLM1 | protein_coding | ENSBTAG00000031569.4 | golgi membrane protein 1 [Source:VGNC Symbol;Acc:VGNC:29491] |
| 8 | 79,446,098 | 79,458,984 | ISCA1 | protein_coding | ENSBTAG00000053156.1 | iron-sulfur cluster assembly 1 [Source:NCBI gene (formerly Entrezgene);Acc:514652] |
| 8 | 79,466,141 | 79,523,806 | TUT7 | protein_coding | ENSBTAG00000001737.6 | terminal uridylyl transferase 7 [Source:VGNC Symbol;Acc:VGNC:37121] |
| 8 | 84,187,019 | 84,246,783 | IPPK | protein_coding | ENSBTAG00000011859.6 | inositol-pentakisphosphate 2-kinase [Source:VGNC Symbol;Acc:VGNC:56974] |
| 8 | 84,256,055 | 84,311,895 | BICD2 | protein_coding | ENSBTAG00000046549.2 | BICD cargo adaptor 2 [Source:VGNC Symbol;Acc:VGNC:26492] |
| 8 | 84,388,645 | 84,420,636 | FGD3 | protein_coding | ENSBTAG00000006939.6 | FYVE, RhoGEF and PH domain containing 3 [Source:VGNC Symbol;Acc:VGNC:28966] |
| 8 | 85,905,346 | 86,141,520 | ROR2 | protein_coding | ENSBTAG00000005092.7 | receptor tyrosine kinase like orphan receptor 2 [Source:VGNC Symbol;Acc:VGNC:34087] |
| 8 | 86,504,014 | 86,686,160 | AUH | protein_coding | ENSBTAG00000047649.2 | AU RNA binding methylglutaconyl-CoA hydratase [Source:NCBI gene (formerly Entrezgene);Acc:614716] |
| 9 | 44,880,467 | 44,930,046 | BVES | protein_coding | ENSBTAG00000018790.5 | blood vessel epicardial substance [Source:VGNC Symbol;Acc:VGNC:26610] |
| 9 | 44,948,437 | 45,069,126 | LIN28B | protein_coding | ENSBTAG00000043973.3 | lin-28 homolog B [Source:VGNC Symbol;Acc:VGNC:108131] |
| 9 | 47,889,245 | 48,618,507 | GRIK2 | protein_coding | ENSBTAG00000033153.4 | glutamate ionotropic receptor kainate type subunit 2 [Source:VGNC Symbol;Acc:VGNC:29643] |
| 9 | 71,362,334 | 71,725,682 | EYA4 | protein_coding | ENSBTAG00000021970.6 | EYA transcriptional coactivator and phosphatase 4 [Source:VGNC Symbol;Acc:VGNC:28673] |
| 9 | 72,066,568 | 72,069,851 | TCF21 | protein_coding | ENSBTAG00000021827.5 | transcription factor 21 [Source:VGNC Symbol;Acc:VGNC:35690] |
| 9 | 72,112,133 | 72,151,639 | TBPL1 | protein_coding | ENSBTAG00000035370.3 | TATA-box binding protein like 1 [Source:VGNC Symbol;Acc:VGNC:35660] |
| 9 | 72,154,434 | 72,223,405 | SLC2A12 | protein_coding | ENSBTAG00000019002.4 | solute carrier family 2 member 12 [Source:VGNC Symbol;Acc:VGNC:34798] |
| 12 | 31,001,535 | 31,388,275 | MTUS2 | protein_coding | ENSBTAG00000001094.6 | microtubule associated scaffold protein 2 [Source:VGNC Symbol;Acc:VGNC:31757] |
| 17 | 66,251,943 | 66,289,558 | CRYBB1 | protein_coding | ENSBTAG00000019280.6 | crystallin beta B1 [Source:VGNC Symbol;Acc:VGNC:27736] |
| 18 | 8,717,274 | 8,729,011 | MPHOSPH6 | protein_coding | ENSBTAG00000009746.5 | M-phase phosphoprotein 6 [Source:VGNC Symbol;Acc:VGNC:31574] |
| 18 | 9,350,020 | 10,154,230 | CDH13 | protein_coding | ENSBTAG00000034373.4 | cadherin 13 [Source:VGNC Symbol;Acc:VGNC:27093] |
| 18 | 10,520,491 | 10,581,675 | WFDC1 | protein_coding | ENSBTAG00000049788.1 | WAP four-disulfide core domain 1 [Source:VGNC Symbol;Acc:VGNC:107028] |
| 18 | 10,607,005 | 10,683,673 | ATP2C2 | protein_coding | ENSBTAG00000000945.7 | ATPase secretory pathway Ca2+ transporting 2 [Source:VGNC Symbol;Acc:VGNC:26297] |
| 18 | 10,690,109 | 10,751,195 | MEAK7 | protein_coding | ENSBTAG00000000946.6 | MTOR associated protein, eak-7 homolog [Source:VGNC Symbol;Acc:VGNC:35889] |
| 18 | 11,409,193 | 11,645,115 | GSE1 | protein_coding | ENSBTAG00000009918.6 | Gse1 coiled-coil protein [Source:VGNC Symbol;Acc:VGNC:55049] |
| 22 | 45,924,535 | 46,818,511 | CACNA2D3 | protein_coding | ENSBTAG00000013117.7 | calcium voltage-gated channel auxiliary subunit alpha2delta 3 [Source:VGNC Symbol;Acc:VGNC:26681] |
| 22 | 47,024,359 | 47,031,572 | SELENOK | protein_coding | ENSBTAG00000032374.5 | selenoprotein K [Source:NCBI gene (formerly Entrezgene);Acc:615114] |
| 22 | 47,032,971 | 47,056,658 | ACTR8 | protein_coding | ENSBTAG00000011180.4 | actin related protein 8 [Source:VGNC Symbol;Acc:VGNC:25589] |
| 22 | 47,051,292 | 47,066,805 | IL17RB | protein_coding | ENSBTAG00000011178.6 | interleukin 17 receptor B [Source:HGNC Symbol;Acc:HGNC:18015] |
| 22 | 47,133,914 | 47,158,681 | CHDH | protein_coding | ENSBTAG00000010027.5 | choline dehydrogenase [Source:VGNC Symbol;Acc:VGNC:27285] |
| 22 | 47,162,857 | 47,508,559 | CACNA1D | protein_coding | ENSBTAG00000010026.6 | calcium voltage-gated channel subunit alpha1 D [Source:VGNC Symbol;Acc:VGNC:26674] |
| 29 | 41,285,605 | 41,293,751 | SLC22A6 | protein_coding | ENSBTAG00000013642.4 | solute carrier family 22 member 6 [Source:NCBI gene (formerly Entrezgene);Acc:407180] |
| 29 | 41,302,545 | 41,323,004 | SLC22A8 | protein_coding | ENSBTAG00000013644.7 | solute carrier family 22 member 8 [Source:VGNC Symbol;Acc:VGNC:34732] |
| 29 | 41,581,967 | 41,608,502 | SLC22A10 | protein_coding | ENSBTAG00000014584.6 | solute carrier family 22, member 10 [Source:NCBI gene (formerly Entrezgene);Acc:509728] |
| 29 | 41,664,021 | 41,689,067 | SLC22A9 | protein_coding | ENSBTAG00000039463.3 | solute carrier family 22 (organic anion transporter), member 9 [Source:NCBI gene (formerly Entrezgene);Acc:508494] |
| 29 | 41,752,532 | 41,774,361 | PLAAT5 | protein_coding | ENSBTAG00000032730.4 | phospholipase A and acyltransferase 5 [Source:VGNC Symbol;Acc:VGNC:106869] |
| 29 | 41,783,243 | 41,792,648 | LGALS12 | protein_coding | ENSBTAG00000007108.5 | galectin 12 [Source:VGNC Symbol;Acc:VGNC:30851] |

^1^Positions based on ARS-UCD1.2 Bos taurus genome assembly

^2^Ensembl database Gene stable ID version accession

^3^Ensembl database Gene description

Table S7 – Chromosome (BTA) and identification of genes (start and end position, name, type, ID and description) for post-natal mortality (PNM) of Nellore animals

| BTA | ^1^Gene start | ^1^Gene end | Gene name | Gene type | ^2^Gene stable ID version | ^3^Gene description |
| --- | --- | --- | --- | --- | --- | --- |
| 2 | 27,359,574 | 27,712,447 | CERS6 | protein_coding | ENSBTAG00000044179.3 | ceramide synthase 6 [Source:VGNC Symbol;Acc:VGNC:27228] |
| 2 | 27,870,256 | 28,191,872 | STK39 | protein_coding | ENSBTAG00000017162.7 | serine/threonine kinase 39 [Source:VGNC Symbol;Acc:VGNC:56152] |
| 2 | 28,990,245 | 29,067,371 | XIRP2 | protein_coding | ENSBTAG00000017743.6 | xin actin binding repeat containing 2 [Source:VGNC Symbol;Acc:VGNC:36986] |
| 2 | 29,049,864 | 29,049,946 | bta-mir-2285w | miRNA | ENSBTAG00000051197.1 | bta-mir-2285w [Source:miRBase;Acc:MI0025189] |
| 2 | 30,176,165 | 30,176,271 | U6 | snRNA | ENSBTAG00000042597.2 | U6 spliceosomal RNA [Source:RFAM;Acc:RF00026] |
| 2 | 30,224,956 | 30,320,883 | SCN1A | protein_coding | ENSBTAG00000018520.6 | sodium voltage-gated channel alpha subunit 1 [Source:VGNC Symbol;Acc:VGNC:34345] |
| 2 | 30,617,671 | 30,798,791 | CSRNP3 | protein_coding | ENSBTAG00000039091.4 | cysteine and serine rich nuclear protein 3 [Source:VGNC Symbol;Acc:VGNC:55343] |
| 2 | 30,880,623 | 31,019,443 | SCN2A | protein_coding | ENSBTAG00000038180.4 | sodium voltage-gated channel alpha subunit 2 [Source:VGNC Symbol;Acc:VGNC:34347] |
| 2 | 31,429,997 | 31,587,069 | COBLL1 | protein_coding | ENSBTAG00000005501.6 | cordon-bleu WH2 repeat protein like 1 [Source:VGNC Symbol;Acc:VGNC:106698] |
| 2 | 31,430,122 | 31,430,181 | bta-mir-11978 | miRNA | ENSBTAG00000049050.1 | bta-mir-11978 [Source:miRBase;Acc:MI0038188] |
| 2 | 31,681,686 | 31,810,646 | GRB14 | protein_coding | ENSBTAG00000019291.5 | growth factor receptor bound protein 14 [Source:VGNC Symbol;Acc:VGNC:29630] |
| 2 | 32,625,453 | 32,751,507 | FIGN | protein_coding | ENSBTAG00000008704.6 | fidgetin, microtubule severing factor [Source:VGNC Symbol;Acc:VGNC:29010] |
| 3 | 83,556,891 | 83,936,160 | PATJ | protein_coding | ENSBTAG00000021975.6 | PATJ crumbs cell polarity complex component [Source:VGNC Symbol;Acc:VGNC:32592] |
| 3 | 84,203,809 | 84,620,790 | NFIA | protein_coding | ENSBTAG00000000074.5 | nuclear factor I A [Source:VGNC Symbol;Acc:VGNC:32038] |
| 3 | 85,704,934 | 85,786,712 | C3H1orf87 | protein_coding | ENSBTAG00000009481.6 | chromosome 3 C1orf87 homolog [Source:VGNC Symbol;Acc:VGNC:52717] |
| 5 | 55,516,050 | 55,539,039 | ATP23 | protein_coding | ENSBTAG00000017543.6 | ATP23 metallopeptidase and ATP synthase assembly factor homolog [Source:VGNC Symbol;Acc:VGNC:26289] |
| 5 | 55,634,355 | 55,656,768 | CTDSP2 | protein_coding | ENSBTAG00000016508.5 | CTD small phosphatase 2 [Source:VGNC Symbol;Acc:VGNC:27791] |
| 5 | 55,652,073 | 55,652,156 | bta-mir-26a-2 | miRNA | ENSBTAG00000029869.2 | bta-mir-26a-2 [Source:miRBase;Acc:MI0004731] |
| 5 | 99,425,439 | 99,435,639 | KLRJ1 | protein_coding | ENSBTAG00000022861.4 | killer cell lectin-like receptor family J member 1 [Source:NCBI gene (formerly Entrezgene);Acc:444861] |
| 5 | 99,539,090 | 99,544,252 | NKG2A | protein_coding | ENSBTAG00000052658.1 | NKG2-A/NKG2-B type II integral membrane protein-like [Source:NCBI gene (formerly Entrezgene);Acc:514897] |
| 5 | 100,679,985 | 100,732,005 | OVOS2 | protein_coding | ENSBTAG00000005243.6 | ovostatin 2 [Source:NCBI gene (formerly Entrezgene);Acc:508026] |
| 5 | 100,873,612 | 100,891,039 | A2M | protein_coding | ENSBTAG00000018137.6 | alpha-2-macroglobulin [Source:VGNC Symbol;Acc:VGNC:55837] |
| 5 | 100,946,808 | 100,964,339 | KLRG1 | protein_coding | ENSBTAG00000013640.5 | killer cell lectin like receptor G1 [Source:VGNC Symbol;Acc:VGNC:30687] |
| 5 | 100,984,954 | 100,998,031 | M6PR | protein_coding | ENSBTAG00000018207.4 | mannose-6-phosphate receptor, cation dependent [Source:VGNC Symbol;Acc:VGNC:31125] |
| 5 | 100,997,211 | 101,017,135 | PHC1 | protein_coding | ENSBTAG00000018205.6 | polyhomeotic homolog 1 [Source:VGNC Symbol;Acc:VGNC:32810] |
| 5 | 101,041,433 | 101,090,609 | A2ML1 | protein_coding | ENSBTAG00000004097.6 | alpha-2-macroglobulin like 1 [Source:VGNC Symbol;Acc:VGNC:25435] |
| 5 | 101,087,152 | 101,087,214 | bta-mir-2284r | miRNA | ENSBTAG00000044623.2 | bta-mir-2284r [Source:miRBase;Acc:MI0011488] |
| 5 | 101,125,927 | 101,163,364 | RIMKLB | protein_coding | ENSBTAG00000003291.6 | ribosomal modification protein rimK like family member B [Source:VGNC Symbol;Acc:VGNC:33971] |
| 9 | 40,698,480 | 40,710,698 | MICAL1 | protein_coding | ENSBTAG00000008147.6 | microtubule associated monooxygenase, calponin and LIM domain containing 1 [Source:VGNC Symbol;Acc:VGNC:31457] |
| 9 | 40,710,828 | 40,714,565 | SMPD2 | protein_coding | ENSBTAG00000008145.4 | sphingomyelin phosphodiesterase 2 [Source:VGNC Symbol;Acc:VGNC:35030] |
| 9 | 40,714,260 | 40,749,877 | PPIL6 | protein_coding | ENSBTAG00000008144.4 | peptidylprolyl isomerase like 6 [Source:VGNC Symbol;Acc:VGNC:33205] |
| 9 | 40,759,545 | 40,770,266 | CD164 | protein_coding | ENSBTAG00000017992.4 | CD164 molecule [Source:VGNC Symbol;Acc:VGNC:27004] |
| 9 | 41,655,110 | 41,851,120 | AFG1L | protein_coding | ENSBTAG00000014592.6 | AFG1 like ATPase [Source:VGNC Symbol;Acc:VGNC:25713] |
| 9 | 41,870,929 | 41,918,140 | SNX3 | protein_coding | ENSBTAG00000018801.4 | sorting nexin 3 [Source:VGNC Symbol;Acc:VGNC:35107] |
| 9 | 41,936,619 | 41,957,320 | NR2E1 | protein_coding | ENSBTAG00000013991.6 | nuclear receptor subfamily 2 group E member 1 [Source:VGNC Symbol;Acc:VGNC:32238] |
| 9 | 43,655,210 | 43,768,691 | ATG5 | protein_coding | ENSBTAG00000005400.5 | autophagy related 5 [Source:VGNC Symbol;Acc:VGNC:56361] |
| 9 | 43,843,907 | 43,866,544 | PRDM1 | protein_coding | ENSBTAG00000000816.6 | PR/SET domain 1 [Source:VGNC Symbol;Acc:VGNC:33288] |
| 9 | 44,576,094 | 44,708,594 | PREP | protein_coding | ENSBTAG00000016281.5 | prolyl endopeptidase [Source:VGNC Symbol;Acc:VGNC:33310] |
| 9 | 44,857,715 | 44,860,928 | POPDC3 | protein_coding | ENSBTAG00000046017.2 | popeye domain containing 3 [Source:VGNC Symbol;Acc:VGNC:33168] |
| 9 | 44,880,467 | 44,930,046 | BVES | protein_coding | ENSBTAG00000018790.5 | blood vessel epicardial substance [Source:VGNC Symbol;Acc:VGNC:26610] |
| 9 | 44,948,437 | 45069126 | LIN28B | protein_coding | ENSBTAG00000043973.3 | lin-28 homolog B [Source:VGNC Symbol;Acc:VGNC:108131] |
| 9 | 49,096,929 | 49,429,819 | ASCC3 | protein_coding | ENSBTAG00000020482.6 | activating signal cointegrator 1 complex subunit 3 [Source:VGNC Symbol;Acc:VGNC:26205] |
| 10 | 82,281,701 | 82,314,251 | TTC9 | protein_coding | ENSBTAG00000045604.2 | tetratricopeptide repeat domain 9 [Source:VGNC Symbol;Acc:VGNC:55151] |
| 10 | 82,375,960 | 82,454,849 | MAP3K9 | protein_coding | ENSBTAG00000005700.6 | mitogen-activated protein kinase kinase kinase 9 [Source:VGNC Symbol;Acc:VGNC:31203] |
| 10 | 82,525,805 | 82,704,991 | PCNX1 | protein_coding | ENSBTAG00000012565.6 | pecanex 1 [Source:VGNC Symbol;Acc:VGNC:32638] |
| 10 | 83,871,181 | 84,062,445 | RGS6 | protein_coding | ENSBTAG00000014189.6 | regulator of G protein signaling 6 [Source:VGNC Symbol;Acc:VGNC:54476] |
| 10 | 84,122,761 | 84,405,606 | DPF3 | protein_coding | ENSBTAG00000025458.4 | double PHD fingers 3 [Source:VGNC Symbol;Acc:VGNC:28177] |
| 10 | 85,277,621 | 85,320,048 | ZNF410 | protein_coding | ENSBTAG00000020327.5 | zinc finger protein 410 [Source:VGNC Symbol;Acc:VGNC:37271] |
| 10 | 85,321,516 | 85,333,750 | FAM161B | protein_coding | ENSBTAG00000020329.6 | FAM161 centrosomal protein B [Source:VGNC Symbol;Acc:VGNC:28743] |
| 10 | 85,333,909 | 85,348,765 | COQ6 | protein_coding | ENSBTAG00000020331.4 | coenzyme Q6, monooxygenase [Source:VGNC Symbol;Acc:VGNC:27614] |
| 10 | 85,351,607 | 85,377,557 | ENTPD5 | protein_coding | ENSBTAG00000020334.6 | ectonucleoside triphosphate diphosphohydrolase 5 (inactive) [Source:VGNC Symbol;Acc:VGNC:28511] |
| 10 | 85,384,726 | 85,426,894 | BBOF1 | protein_coding | ENSBTAG00000018467.5 | basal body orientation factor 1 [Source:VGNC Symbol;Acc:VGNC:26431] |
| 10 | 85,428,884 | 85,447,592 | ALDH6A1 | protein_coding | ENSBTAG00000018469.4 | aldehyde dehydrogenase 6 family member A1 [Source:VGNC Symbol;Acc:VGNC:25817] |
| 10 | 85,448,145 | 85,560,860 | LIN52 | protein_coding | ENSBTAG00000030557.3 | lin-52 DREAM MuvB core complex component [Source:VGNC Symbol;Acc:VGNC:30893] |
| 10 | 85,599,605 | 85,618,776 | VSX2 | protein_coding | ENSBTAG00000014632.5 | visual system homeobox 2 [Source:VGNC Symbol;Acc:VGNC:36846] |
| 10 | 85,631,547 | 85,647,111 | ABCD4 | protein_coding | ENSBTAG00000014633.6 | ATP binding cassette subfamily D member 4 [Source:VGNC Symbol;Acc:VGNC:55099] |
| 10 | 85,679,086 | 85,681,188 | VRTN | protein_coding | ENSBTAG00000005774.5 | vertebrae development associated [Source:VGNC Symbol;Acc:VGNC:36837] |
| 10 | 86,610,233 | 86,655,611 | JDP2 | protein_coding | ENSBTAG00000009451.5 | Jun dimerization protein 2 [Source:VGNC Symbol;Acc:VGNC:30374] |
| 10 | 86,624,969 | 86,625,047 | bta-mir-10162 | miRNA | ENSBTAG00000049624.1 | bta-mir-10162 [Source:miRBase;Acc:MI0032914] |
| 10 | 86,713,963 | 86736092 | BATF | protein_coding | ENSBTAG00000025405.4 | basic leucine zipper ATF-like transcription factor [Source:VGNC Symbol;Acc:VGNC:26425] |
| 10 | 87,459,365 | 87,517,588 | GPATCH2L | protein_coding | ENSBTAG00000003084.7 | G-patch domain containing 2 like [Source:VGNC Symbol;Acc:VGNC:29517] |
| 12 | 29,967,343 | 30,001,111 | TEX26 | protein_coding | ENSBTAG00000033386.4 | testis expressed 26 [Source:VGNC Symbol;Acc:VGNC:35764] |
| 12 | 29,997,123 | 30,014,621 | MEDAG | protein_coding | ENSBTAG00000008271.5 | mesenteric estrogen dependent adiposis [Source:VGNC Symbol;Acc:VGNC:31371] |
| 12 | 30,108,987 | 30,138,259 | ALOX5AP | protein_coding | ENSBTAG00000013201.4 | arachidonate 5-lipoxygenase activating protein [Source:VGNC Symbol;Acc:VGNC:25845] |
| 12 | 30,478,919 | 30,533,274 | KATNAL1 | protein_coding | ENSBTAG00000009340.6 | katanin catalytic subunit A1 like 1 [Source:VGNC Symbol;Acc:VGNC:30406] |
| 12 | 30,562,148 | 30,562,253 | SNORA70 | snoRNA | ENSBTAG00000045239.2 | Small nucleolar RNA SNORA70 [Source:RFAM;Acc:RF00156] |
| 12 | 30,806,229 | 30,854,060 | UBL3 | protein_coding | ENSBTAG00000012170.5 | ubiquitin like 3 [Source:VGNC Symbol;Acc:VGNC:36607] |
| 12 | 30,934,105 | 30,991,889 | SLC7A1 | protein_coding | ENSBTAG00000018577.4 | solute carrier family 7 member 1 [Source:VGNC Symbol;Acc:VGNC:34924] |
| 12 | 31,001,535 | 31,388,275 | MTUS2 | protein_coding | ENSBTAG00000001094.6 | microtubule associated scaffold protein 2 [Source:VGNC Symbol;Acc:VGNC:31757] |
| 18 | 93,50,020 | 10,154,230 | CDH13 | protein_coding | ENSBTAG00000034373.4 | cadherin 13 [Source:VGNC Symbol;Acc:VGNC:27093] |
| 18 | 35,970,301 | 36,017,135 | CDH3 | protein_coding | ENSBTAG00000033248.4 | cadherin 3 [Source:VGNC Symbol;Acc:VGNC:27103] |
| 18 | 36,046,665 | 36,119,368 | CDH1 | protein_coding | ENSBTAG00000015991.6 | cadherin 1 [Source:VGNC Symbol;Acc:VGNC:27090] |
| 18 | 36,121,354 | 36,305,835 | TANGO6 | protein_coding | ENSBTAG00000013941.6 | transport and golgi organization 6 homolog [Source:VGNC Symbol;Acc:VGNC:35593] |
| 19 | 8,092,267 | 8,492,726 | MSI2 | protein_coding | ENSBTAG00000004406.7 | musashi RNA binding protein 2 [Source:VGNC Symbol;Acc:VGNC:107272] |
| 19 | 8,484,500 | 8,484,590 | bta-mir-378d | miRNA | ENSBTAG00000047296.2 | bta-mir-378d [Source:miRBase;Acc:MI0031501] |
| 19 | 8,562,129 | 8,562,590 | CCDC182 | protein_coding | ENSBTAG00000047272.2 | coiled-coil domain containing 182 [Source:VGNC Symbol;Acc:VGNC:26877] |
| 19 | 8,655,164 | 8,661,689 | MRPS23 | protein_coding | ENSBTAG00000012366.4 | mitochondrial ribosomal protein S23 [Source:VGNC Symbol;Acc:VGNC:31663] |
| 19 | 8,670,138 | 8,760,139 | CUEDC1 | protein_coding | ENSBTAG00000007147.6 | CUE domain containing 1 [Source:VGNC Symbol;Acc:VGNC:27829] |
| 19 | 8,775,705 | 8,787,368 | VEZF1 | protein_coding | ENSBTAG00000004193.5 | vascular endothelial zinc finger 1 [Source:VGNC Symbol;Acc:VGNC:36788] |
| 19 | 8,806,081 | 8,809,808 | SRSF1 | protein_coding | ENSBTAG00000014766.4 | serine and arginine rich splicing factor 1 [Source:VGNC Symbol;Acc:VGNC:35297] |
| 19 | 8,885,982 | 8,893,225 | DYNLL2 | protein_coding | ENSBTAG00000025313.3 | dynein light chain LC8-type 2 [Source:VGNC Symbol;Acc:VGNC:28275] |
| 19 | 11,607,447 | 11,633,858 | TBX4 | protein_coding | ENSBTAG00000009968.6 | T-box transcription factor 4 [Source:VGNC Symbol;Acc:VGNC:35670] |
| 19 | 13,301,089 | 13,307,764 | LHX1 | protein_coding | ENSBTAG00000033562.3 | LIM homeobox 1 [Source:VGNC Symbol;Acc:VGNC:30871] |
| 19 | 13,312,212 | 13,420,345 | AATF | protein_coding | ENSBTAG00000021927.5 | apoptosis antagonizing transcription factor [Source:VGNC Symbol;Acc:VGNC:25452] |
| 19 | 14,543,295 | 14,573,019 | TAF15 | protein_coding | ENSBTAG00000006916.6 | TATA-box binding protein associated factor 15 [Source:VGNC Symbol;Acc:VGNC:54497] |
| 19 | 14,587,761 | 14,613,432 | MMP28 | protein_coding | ENSBTAG00000006086.5 | matrix metallopeptidase 28 [Source:VGNC Symbol;Acc:VGNC:31528] |
| 19 | 14,614,634 | 14,615,948 | C19H17orf50 | protein_coding | ENSBTAG00000045621.2 | chromosome 19 C17orf50 homolog [Source:VGNC Symbol;Acc:VGNC:52653] |
| 19 | 14,621,338 | 14,628,732 | GAS2L2 | protein_coding | ENSBTAG00000018519.3 | growth arrest specific 2 like 2 [Source:VGNC Symbol;Acc:VGNC:29259] |
| 19 | 14,632,048 | 14,641,729 | RASL10B | protein_coding | ENSBTAG00000018518.6 | RAS like family 10 member B [Source:VGNC Symbol;Acc:VGNC:33753] |
| 19 | 14,648,905 | 14,757,836 | AP2B1 | protein_coding | ENSBTAG00000020316.6 | adaptor related protein complex 2 subunit beta 1 [Source:VGNC Symbol;Acc:VGNC:55033] |
| 19 | 14,874,860 | 14,875,020 | U1 | snRNA | ENSBTAG00000028421.2 | U1 spliceosomal RNA [Source:RFAM;Acc:RF00003] |
| 19 | 14,913,862 | 14,947,268 | UNC45B | protein_coding | ENSBTAG00000002898.5 | unc-45 myosin chaperone B [Source:VGNC Symbol;Acc:VGNC:36668] |
| 19 | 14,954,563 | 14,963,358 | NLE1 | protein_coding | ENSBTAG00000019094.5 | notchless homolog 1 [Source:VGNC Symbol;Acc:VGNC:32109] |
| 19 | 14,964,939 | 14,976,767 | FNDC8 | protein_coding | ENSBTAG00000019092.4 | fibronectin type III domain containing 8 [Source:VGNC Symbol;Acc:VGNC:29066] |
| 19 | 14,978,195 | 14,991,205 | RAD51D | protein_coding | ENSBTAG00000019082.4 | RAD51 paralog D [Source:VGNC Symbol;Acc:VGNC:33687] |
| 19 | 14,988,931 | 14,989,001 | bta-mir-2331 | miRNA | ENSBTAG00000045174.2 | bta-mir-2331 [Source:miRBase;Acc:MI0011357] |
| 19 | 14,997,717 | 15,072,713 | RFFL | protein_coding | ENSBTAG00000013645.6 | ring finger and FYVE like domain containing E3 ubiquitin protein ligase [Source:VGNC Symbol;Acc:VGNC:57149] |
| 19 | 15,077,781 | 15,098,380 | LIG3 | protein_coding | ENSBTAG00000018689.6 | DNA ligase 3 [Source:VGNC Symbol;Acc:VGNC:30883] |
| 19 | 15,118,728 | 15,120,467 | ZNF830 | protein_coding | ENSBTAG00000020340.3 | zinc finger protein 830 [Source:VGNC Symbol;Acc:VGNC:37354] |
| 19 | 15,120,579 | 15,156,414 | CCT6B | protein_coding | ENSBTAG00000020338.5 | chaperonin containing TCP1 subunit 6B [Source:VGNC Symbol;Acc:VGNC:26999] |
| 19 | 15,463,728 | 15,480,095 | TMEM132E | protein_coding | ENSBTAG00000002680.5 | transmembrane protein 132E [Source:VGNC Symbol;Acc:VGNC:35968] |
| 19 | 15,867,084 | 15,869,981 | CCL11 | protein_coding | ENSBTAG00000004129.4 | chemokine (C-C motif) ligand 11 [Source:NCBI gene (formerly Entrezgene);Acc:404072] |
| 19 | 15,902,777 | 15,905,368 | CCL2 | protein_coding | ENSBTAG00000037811.3 | chemokine (C-C motif) ligand 2 [Source:NCBI gene (formerly Entrezgene);Acc:281043] |
| 19 | 16,022,746 | 17,228,096 | ASIC2 | protein_coding | ENSBTAG00000025200.5 | acid sensing ion channel subunit 2 [Source:VGNC Symbol;Acc:VGNC:26211] |
| 19 | 16,073,672 | 16,073,753 | U4 | snRNA | ENSBTAG00000044815.2 | U4 spliceosomal RNA [Source:RFAM;Acc:RF00015] |
| 19 | 16,162,780 | 16,162,886 | 5S_rRNA | rRNA | ENSBTAG00000043750.2 | 5S ribosomal RNA [Source:RFAM;Acc:RF00001] |
| 19 | 17,330,801 | 17,691,005 | MYO1D | protein_coding | ENSBTAG00000015527.5 | myosin ID [Source:VGNC Symbol;Acc:VGNC:31819] |
| 19 | 18,061,035 | 18,096,596 | ATAD5 | protein_coding | ENSBTAG00000018383.6 | ATPase family AAA domain containing 5 [Source:VGNC Symbol;Acc:VGNC:26235] |
| 19 | 18,107,484 | 18,149,013 | CRLF3 | protein_coding | ENSBTAG00000018381.6 | cytokine receptor like factor 3 [Source:VGNC Symbol;Acc:VGNC:27718] |
| 19 | 18,156,522 | 18,196,012 | SUZ12 | protein_coding | ENSBTAG00000018752.4 | SUZ12 polycomb repressive complex 2 subunit [Source:VGNC Symbol;Acc:VGNC:35493] |
| 19 | 18,221,902 | 18,249,177 | UTP6 | protein_coding | ENSBTAG00000015612.5 | UTP6 small subunit processome component [Source:VGNC Symbol;Acc:VGNC:36748] |
| 19 | 18232999 | 18,233,121 | SNORA54 | snoRNA | ENSBTAG00000042680.2 | small nucleolar RNA, H/ACA box 54 [Source:HGNC Symbol;Acc:HGNC:32647] |
| 19 | 18,233,055 | 18,233,123 | bta-mir-2332 | miRNA | ENSBTAG00000051634.1 | bta-mir-2332 [Source:miRBase;Acc:MI0011358] |
| 19 | 18,255,692 | 18,262,657 | COPRS | protein_coding | ENSBTAG00000049483.1 | coordinator of PRMT5 and differentiation stimulator [Source:VGNC Symbol;Acc:VGNC:27598] |
| 19 | 18,461,502 | 18,461,612 | bta-mir-365-2 | miRNA | ENSBTAG00000029914.2 | bta-mir-365-2 [Source:miRBase;Acc:MI0009812] |
| 19 | 18,463,796 | 18,463,858 | bta-mir-2333 | miRNA | ENSBTAG00000045149.2 | bta-mir-2333 [Source:miRBase;Acc:MI0011359] |
| 19 | 18,474,461 | 18,474,541 | bta-mir-193a | miRNA | ENSBTAG00000029955.2 | bta-mir-193a [Source:miRBase;Acc:MI0005014] |
| 19 | 18,501,132 | 18,574,408 | RAB11FIP4 | protein_coding | ENSBTAG00000016972.5 | RAB11 family interacting protein 4 [Source:VGNC Symbol;Acc:VGNC:33619] |
| 19 | 18,667,321 | 18,671,399 | EVI2A | protein_coding | ENSBTAG00000009354.3 | ecotropic viral integration site 2A [Source:NCBI gene (formerly Entrezgene);Acc:528920] |
| 19 | 18,674,661 | 18,683,264 | EVI2B | protein_coding | ENSBTAG00000009353.4 | ecotropic viral integration site 2B [Source:VGNC Symbol;Acc:VGNC:55822] |
| 19 | 18,690,773 | 18,693,479 | OMG | protein_coding | ENSBTAG00000025213.3 | oligodendrocyte myelin glycoprotein [Source:VGNC Symbol;Acc:VGNC:50238] |
| 19 | 18,763,113 | 18,873,847 | NF1 | protein_coding | ENSBTAG00000011829.6 | neurofibromin 1 [Source:VGNC Symbol;Acc:VGNC:54463] |
| 19 | 19,651,228 | 19,780,827 | NLK | protein_coding | ENSBTAG00000014825.5 | nemo like kinase [Source:VGNC Symbol;Acc:VGNC:32111] |
| 19 | 19,943,529 | 19,967,651 | SLC13A2 | protein_coding | ENSBTAG00000008788.6 | solute carrier family 13 member 2 [Source:VGNC Symbol;Acc:VGNC:34674] |
| 19 | 19,977,270 | 20,005,151 | FOXN1 | protein_coding | ENSBTAG00000013095.4 | forkhead box N1 [Source:VGNC Symbol;Acc:VGNC:29095] |
| 19 | 20,009,504 | 20,015,204 | UNC119 | protein_coding | ENSBTAG00000013096.5 | unc-119 lipid binding chaperone [Source:VGNC Symbol;Acc:VGNC:36662] |
| 19 | 20,016,185 | 20,027,945 | PIGS | protein_coding | ENSBTAG00000013098.6 | phosphatidylinositol glycan anchor biosynthesis class S [Source:VGNC Symbol;Acc:VGNC:32877] |
| 19 | 20,030,282 | 20,034,197 | ALDOC | protein_coding | ENSBTAG00000013099.5 | aldolase, fructose-bisphosphate C [Source:VGNC Symbol;Acc:VGNC:25823] |
| 19 | 20,034,503 | 20,053,455 | SPAG5 | protein_coding | ENSBTAG00000013100.6 | sperm associated antigen 5 [Source:VGNC Symbol;Acc:VGNC:55873] |
| 19 | 20,056,746 | 20,060,305 | RSKR | protein_coding | ENSBTAG00000011984.5 | ribosomal protein S6 kinase related [Source:VGNC Symbol;Acc:VGNC:57704] |
| 19 | 20,060,814 | 20,088,152 | KIAA0100 | protein_coding | ENSBTAG00000011988.6 | KIAA0100 [Source:VGNC Symbol;Acc:VGNC:30546] |
| 19 | 20,090,371 | 20,098,195 | SDF2 | protein_coding | ENSBTAG00000012845.4 | stromal cell derived factor 2 [Source:VGNC Symbol;Acc:VGNC:34385] |
| 19 | 20,098,012 | 20,129,719 | SUPT6H | protein_coding | ENSBTAG00000009036.6 | SPT6 homolog, histone chaperone and transcription elongation factor [Source:VGNC Symbol;Acc:VGNC:50619] |
| 19 | 20,436,390 | 20,467,849 | MYO18A | protein_coding | ENSBTAG00000017380.6 | myosin XVIIIA [Source:VGNC Symbol;Acc:VGNC:31813] |
| 19 | 20,543,611 | 20,543,742 | SNORA72 | snoRNA | ENSBTAG00000042201.2 | Small nucleolar RNA SNORA72 [Source:RFAM;Acc:RF00139] |
| 19 | 20,576,451 | 20,591,495 | CRYBA1 | protein_coding | ENSBTAG00000005352.2 | crystallin beta A1 [Source:VGNC Symbol;Acc:VGNC:27733] |
| 19 | 20,612,946 | 20,620,127 | NUFIP2 | protein_coding | ENSBTAG00000033077.4 | nuclear FMR1 interacting protein 2 [Source:VGNC Symbol;Acc:VGNC:32346] |
| 19 | 20,882,415 | 21,124,185 | SSH2 | protein_coding | ENSBTAG00000011011.6 | slingshot protein phosphatase 2 [Source:VGNC Symbol;Acc:VGNC:35313] |
| 19 | 21,175,356 | 21,193,833 | EFCAB5 | protein_coding | ENSBTAG00000019122.6 | EF-hand calcium binding domain 5 [Source:VGNC Symbol;Acc:VGNC:28345] |
| 19 | 21,237,162 | 21,283,030 | NSRP1 | protein_coding | ENSBTAG00000019341.6 | nuclear speckle splicing regulatory protein 1 [Source:VGNC Symbol;Acc:VGNC:32284] |
| 19 | 21,290,410 | 21,323,440 | SLC6A4 | protein_coding | ENSBTAG00000019349.6 | solute carrier family 6 member 4 [Source:VGNC Symbol;Acc:VGNC:49971] |
| 19 | 21,889,110 | 22,053,102 | NXN | protein_coding | ENSBTAG00000000855.6 | nucleoredoxin [Source:VGNC Symbol;Acc:VGNC:32376] |
| 19 | 22,060,689 | 22,065,001 | MRM3 | protein_coding | ENSBTAG00000019162.6 | mitochondrial rRNA methyltransferase 3 [Source:VGNC Symbol;Acc:VGNC:31607] |
| 19 | 22,065,122 | 22,084,876 | GLOD4 | protein_coding | ENSBTAG00000019160.6 | glyoxalase domain containing 4 [Source:VGNC Symbol;Acc:VGNC:50005] |
| 19 | 22,069,218 | 22,069,286 | bta-mir-2335 | miRNA | ENSBTAG00000050210.1 | bta-mir-2335 [Source:miRBase;Acc:MI0011361] |
| 19 | 22,088,342 | 22,096,721 | GEMIN4 | protein_coding | ENSBTAG00000013393.6 | gem nuclear organelle associated protein 4 [Source:VGNC Symbol;Acc:VGNC:29317] |
| 19 | 22,088,603 | 22,089,801 | DBIL5 | protein_coding | ENSBTAG00000013395.4 | endozepine-like peptide 2 [Source:NCBI gene (formerly Entrezgene);Acc:353112] |
| 19 | 22,097,877 | 22,106,826 | TLCD3A | protein_coding | ENSBTAG00000046273.2 | TLC domain containing 3A [Source:VGNC Symbol;Acc:VGNC:28822] |
| 19 | 53,005,648 | 53,334,641 | RBFOX3 | protein_coding | ENSBTAG00000006280.6 | RNA binding fox-1 homolog 3 [Source:VGNC Symbol;Acc:VGNC:33779] |
| 19 | 53,335,620 | 53,347,068 | ENGASE | protein_coding | ENSBTAG00000006277.6 | endo-beta-N-acetylglucosaminidase [Source:VGNC Symbol;Acc:VGNC:28493] |
| 19 | 53,358,290 | 53,366,382 | C1QTNF1 | protein_coding | ENSBTAG00000006276.6 | C1q and TNF related 1 [Source:VGNC Symbol;Acc:VGNC:26622] |
| 19 | 53,391,266 | 53,406,328 | CANT1 | protein_coding | ENSBTAG00000009991.4 | calcium activated nucleotidase 1 [Source:VGNC Symbol;Acc:VGNC:26735] |
| 19 | 53,416,674 | 53,427,208 | LGALS3BP | protein_coding | ENSBTAG00000001368.5 | galectin 3 binding protein [Source:VGNC Symbol;Acc:VGNC:30853] |
| 19 | 53,460,671 | 53,514,937 | TIMP2 | protein_coding | ENSBTAG00000010899.4 | TIMP metallopeptidase inhibitor 2 [Source:VGNC Symbol;Acc:VGNC:35871] |
| 19 | 53,522,615 | 53,553,993 | USP36 | protein_coding | ENSBTAG00000021505.6 | ubiquitin specific peptidase 36 [Source:VGNC Symbol;Acc:VGNC:52844] |
| 19 | 53,565,765 | 53,643,768 | CYTH1 | protein_coding | ENSBTAG00000006663.6 | cytohesin 1 [Source:VGNC Symbol;Acc:VGNC:55809] |
| 19 | 53,691,404 | 53,787,907 | DNAH17 | protein_coding | ENSBTAG00000000920.6 | dynein axonemal heavy chain 17 [Source:VGNC Symbol;Acc:VGNC:28124] |
| 19 | 53,787,203 | 53,824,616 | PGS1 | protein_coding | ENSBTAG00000000675.6 | phosphatidylglycerophosphate synthase 1 [Source:VGNC Symbol;Acc:VGNC:32803] |
| 19 | 53,840,159 | 53,840,858 | SOCS3 | protein_coding | ENSBTAG00000008441.3 | suppressor of cytokine signaling 3 [Source:VGNC Symbol;Acc:VGNC:35121] |
| 19 | 53,928,092 | 53,933,612 | TMEM235 | protein_coding | ENSBTAG00000024926.5 | transmembrane protein 235 [Source:VGNC Symbol;Acc:VGNC:36048] |
| 19 | 53,940,667 | 53,947,960 | BIRC5 | protein_coding | ENSBTAG00000013573.3 | baculoviral IAP repeat containing 5 [Source:VGNC Symbol;Acc:VGNC:26501] |
| 19 | 53,954,985 | 53,977,477 | AFMID | protein_coding | ENSBTAG00000007125.6 | arylformamidase [Source:VGNC Symbol;Acc:VGNC:25716] |
| 19 | 53,977,498 | 53,991,174 | TK1 | protein_coding | ENSBTAG00000007121.6 | thymidine kinase 1 [Source:VGNC Symbol;Acc:VGNC:35884] |
| 19 | 53,992,203 | 53,995,976 | SYNGR2 | protein_coding | ENSBTAG00000019069.5 | synaptogyrin 2 [Source:VGNC Symbol;Acc:VGNC:107278] |
| 19 | 54,025,900 | 54,048,017 | TMC6 | protein_coding | ENSBTAG00000018661.6 | transmembrane channel like 6 [Source:VGNC Symbol;Acc:VGNC:35922] |
| 19 | 54,038,763 | 54,038,841 | bta-mir-2348 | miRNA | ENSBTAG00000044955.2 | bta-mir-2348 [Source:miRBase;Acc:MI0011376] |
| 19 | 54,051,772 | 54,124,386 | TNRC6C | protein_coding | ENSBTAG00000018658.7 | trinucleotide repeat containing adaptor 6C [Source:VGNC Symbol;Acc:VGNC:36206] |
| 19 | 54,497,005 | 54,676,832 | SEPTIN9 | protein_coding | ENSBTAG00000002633.6 | septin 9 [Source:VGNC Symbol;Acc:VGNC:34459] |
| 19 | 54,906,180 | 54,972,843 | MGAT5B | protein_coding | ENSBTAG00000005104.7 | alpha-1,6-mannosylglycoprotein 6-beta-N-acetylglucosaminyltransferase B [Source:VGNC Symbol;Acc:VGNC:55847] |
| 19 | 55,014,709 | 55,043,048 | MFSD11 | protein_coding | ENSBTAG00000018260.4 | major facilitator superfamily domain containing 11 [Source:VGNC Symbol;Acc:VGNC:31433] |
| 19 | 55,121,131 | 55,140,468 | ST6GALNAC1 | protein_coding | ENSBTAG00000001998.6 | ST6 N-acetylgalactosaminide alpha-2,6-sialyltransferase 1 [Source:VGNC Symbol;Acc:VGNC:35339] |
| 19 | 55,174,502 | 55,193,764 | ST6GALNAC2 | protein_coding | ENSBTAG00000015255.5 | ST6 N-acetylgalactosaminide alpha-2,6-sialyltransferase 2 [Source:VGNC Symbol;Acc:VGNC:35340] |
| 19 | 55,195,660 | 55,195,732 | SNORD1A | snoRNA | ENSBTAG00000043414.2 | small nucleolar RNA, C/D box 1A [Source:HGNC Symbol;Acc:HGNC:32556] |
| 19 | 55,196,206 | 55,196,290 | SNORD1B | snoRNA | ENSBTAG00000042461.2 | small nucleolar RNA, C/D box 1B [Source:HGNC Symbol;Acc:HGNC:32676] |
| 19 | 55,197,780 | 55,197,857 | SNORD1C | snoRNA | ENSBTAG00000042706.2 | small nucleolar RNA, C/D box 1C [Source:HGNC Symbol;Acc:HGNC:32677] |
| 19 | 55,217,714 | 55,227,282 | CYGB | protein_coding | ENSBTAG00000005556.5 | cytoglobin [Source:VGNC Symbol;Acc:VGNC:50268] |
| 19 | 55,249,957 | 55,274,837 | RHBDF2 | protein_coding | ENSBTAG00000003436.6 | rhomboid 5 homolog 2 [Source:VGNC Symbol;Acc:VGNC:33934] |
| 19 | 55,288,432 | 55,290,366 | AANAT | protein_coding | ENSBTAG00000050098.1 | aralkylamine N-acetyltransferase [Source:NCBI gene (formerly Entrezgene);Acc:281583] |
| 19 | 55,299,835 | 55,349,164 | UBE2O | protein_coding | ENSBTAG00000020115.6 | ubiquitin conjugating enzyme E2 O [Source:VGNC Symbol;Acc:VGNC:36591] |
| 19 | 55,350,377 | 55,354,150 | SPHK1 | protein_coding | ENSBTAG00000008507.5 | sphingosine kinase 1 [Source:VGNC Symbol;Acc:VGNC:35210] |
| 19 | 55,387,190 | 55,413,082 | PRPSAP1 | protein_coding | ENSBTAG00000030172.3 | phosphoribosyl pyrophosphate synthetase associated protein 1 [Source:VGNC Symbol;Acc:VGNC:33386] |
| 19 | 55,416,033 | 55,440,784 | QRICH2 | protein_coding | ENSBTAG00000030173.4 | glutamine rich 2 [Source:VGNC Symbol;Acc:VGNC:33603] |
| 19 | 55,443,082 | 55,449,253 | UBALD2 | protein_coding | ENSBTAG00000013792.5 | UBA like domain containing 2 [Source:VGNC Symbol;Acc:VGNC:36570] |
| 19 | 55,464,958 | 55,539,900 | RNF157 | protein_coding | ENSBTAG00000016240.6 | ring finger protein 157 [Source:VGNC Symbol;Acc:VGNC:34031] |
| 19 | 55,541,080 | 55,545,858 | FOXJ1 | protein_coding | ENSBTAG00000007916.6 | forkhead box J1 [Source:VGNC Symbol;Acc:VGNC:29089] |
| 19 | 55,560,758 | 55,581,189 | EXOC7 | protein_coding | ENSBTAG00000007910.5 | exocyst complex component 7 [Source:VGNC Symbol;Acc:VGNC:28652] |

^1^Positions based on ARS-UCD1.2 Bos taurus genome assembly

^2^Ensembl database Gene stable ID version accession

^3^Ensembl database Gene description

Table S8 – Chromosome (BTA) and identification of genes (start and end position, name, type, ID and description) for stayability (STAY) of Nellore females

| BTA | ^1^Gene start | ^1^Gene end | Gene name | Gene type | ^2^Gene stable ID version | ^3^Gene description |
| --- | --- | --- | --- | --- | --- | --- |
| 2 | 120,805,616 | 120,811,995 | FNDC5 | protein_coding | ENSBTAG00000051540.1 | fibronectin type III domain containing 5 [Source:VGNC Symbol;Acc:VGNC:56109] |
| 2 | 120,815,271 | 120,849,384 | S100PBP | protein_coding | ENSBTAG00000018067.5 | S100P binding protein [Source:VGNC Symbol;Acc:VGNC:34250] |
| 2 | 120,849,154 | 120,881,261 | YARS1 | protein_coding | ENSBTAG00000018065.4 | tyrosyl-tRNA synthetase 1 [Source:VGNC Symbol;Acc:VGNC:37015] |
| 2 | 120,883,700 | 120,910,058 | KIAA1522 | protein_coding | ENSBTAG00000018062.6 | KIAA1522 [Source:VGNC Symbol;Acc:VGNC:30570] |
| 2 | 120,951,772 | 120,969,740 | SYNC | protein_coding | ENSBTAG00000026684.4 | syncoilin, intermediate filament protein [Source:VGNC Symbol;Acc:VGNC:35517] |
| 2 | 120,969,745 | 120,986,234 | RBBP4 | protein_coding | ENSBTAG00000005904.6 | RB binding protein 4, chromatin remodeling factor [Source:VGNC Symbol;Acc:VGNC:33770] |
| 2 | 120,987,011 | 120,998,937 | ZBTB8OS | protein_coding | ENSBTAG00000027159.5 | zinc finger and BTB domain containing 8 opposite strand [Source:VGNC Symbol;Acc:VGNC:53571] |
| 2 | 121,015,835 | 121,076,671 | ZBTB8A | protein_coding | ENSBTAG00000018199.5 | zinc finger and BTB domain containing 8A [Source:VGNC Symbol;Acc:VGNC:37092] |
| 2 | 125,249,517 | 125,390,286 | EYA3 | protein_coding | ENSBTAG00000043989.3 | EYA transcriptional coactivator and phosphatase 3 [Source:VGNC Symbol;Acc:VGNC:28672] |
| 2 | 125,392,642 | 125,400,258 | XKR8 | protein_coding | ENSBTAG00000008800.5 | XK related 8 [Source:VGNC Symbol;Acc:VGNC:36990] |
| 2 | 125,401,099 | 125,432,986 | SMPDL3B | protein_coding | ENSBTAG00000012997.4 | sphingomyelin phosphodiesterase acid like 3B [Source:VGNC Symbol;Acc:VGNC:35034] |
| 2 | 125,448,288 | 125,470,852 | RPA2 | protein_coding | ENSBTAG00000006225.5 | replication protein A2 [Source:VGNC Symbol;Acc:VGNC:34095] |
| 2 | 125,473,679 | 125,486,124 | THEMIS2 | protein_coding | ENSBTAG00000006223.5 | thymocyte selection associated family member 2 [Source:VGNC Symbol;Acc:VGNC:35836] |
| 2 | 125,526,927 | 125,545,896 | PPP1R8 | protein_coding | ENSBTAG00000003866.4 | protein phosphatase 1 regulatory subunit 8 [Source:VGNC Symbol;Acc:VGNC:33249] |
| 2 | 125,541,373 | 125,541,538 | SCARNA1 | scaRNA | ENSBTAG00000044441.2 | small Cajal body-specific RNA 1 [Source:HGNC Symbol;Acc:HGNC:32555] |
| 2 | 125,550,304 | 125,585,869 | STX12 | protein_coding | ENSBTAG00000001192.6 | syntaxin 12 [Source:VGNC Symbol;Acc:VGNC:35432] |
| 2 | 125,604,197 | 125,635,148 | FAM76A | protein_coding | ENSBTAG00000019614.6 | family with sequence similarity 76 member A [Source:VGNC Symbol;Acc:VGNC:28827] |
| 2 | 125,662,055 | 125,665,793 | IFI6 | protein_coding | ENSBTAG00000007554.4 | interferon alpha inducible protein 6 [Source:VGNC Symbol;Acc:VGNC:30047] |
| 2 | 125,688,547 | 125,709,381 | FGR | protein_coding | ENSBTAG00000011784.6 | FGR proto-onco, Src family tyrosine kinase [Source:VGNC Symbol;Acc:VGNC:28994] |
| 2 | 125,759,503 | 125,780,816 | AHDC1 | protein_coding | ENSBTAG00000037456.4 | AT-hook DNA binding motif containing 1 [Source:VGNC Symbol;Acc:VGNC:25753] |
| 2 | 125,814,828 | 125,890,887 | WASF2 | protein_coding | ENSBTAG00000018374.5 | WASP family member 2 [Source:VGNC Symbol;Acc:VGNC:36866] |
| 2 | 125,855,668 | 125,855,774 | U6 | snRNA | ENSBTAG00000052426.1 | U6 spliceosomal RNA [Source:RFAM;Acc:RF00026] |
| 2 | 125,893,878 | 125,894,870 | GPR3 | protein_coding | ENSBTAG00000002505.6 | G protein-coupled receptor 3 [Source:VGNC Symbol;Acc:VGNC:29581] |
| 2 | 125,905,989 | 125,909,985 | CD164L2 | protein_coding | ENSBTAG00000019728.6 | CD164 molecule like 2 [Source:VGNC Symbol;Acc:VGNC:27005] |
| 2 | 125,921,162 | 125,934,238 | MAP3K6 | protein_coding | ENSBTAG00000010254.5 | mitogen-activated protein kinase kinase kinase 6 [Source:VGNC Symbol;Acc:VGNC:31199] |
| 2 | 125,934,205 | 125,940,846 | SYTL1 | protein_coding | ENSBTAG00000010253.6 | synaptotagmin like 1 [Source:VGNC Symbol;Acc:VGNC:35546] |
| 2 | 125946146 | 125,956,940 | TMEM222 | protein_coding | ENSBTAG00000010249.6 | transmembrane protein 222 [Source:VGNC Symbol;Acc:VGNC:36043] |
| 2 | 125,970,802 | 126,036,698 | WDTC1 | protein_coding | ENSBTAG00000053588.1 | WD and tetratricopeptide repeats 1 [Source:VGNC Symbol;Acc:VGNC:107027] |
| 2 | 126,090,279 | 126,142,300 | SLC9A1 | protein_coding | ENSBTAG00000008766.6 | solute carrier family 9 member A1 [Source:NCBI gene (formerly Entrezgene);Acc:317654] |
| 2 | 126,212,749 | 126,220,286 | TENT5B | protein_coding | ENSBTAG00000018413.5 | terminal nucleotidyltransferase 5B [Source:VGNC Symbol;Acc:VGNC:28816] |
| 2 | 126,229,849 | 126,230,547 | TRNP1 | protein_coding | ENSBTAG00000054719.1 | TMF1 regulated nuclear protein 1 [Source:VGNC Symbol;Acc:VGNC:107002] |
| 2 | 126,257,409 | 126,270,759 | KDF1 | protein_coding | ENSBTAG00000019252.5 | keratinocyte differentiation factor 1 [Source:VGNC Symbol;Acc:VGNC:30521] |
| 2 | 126,273,255 | 126,286,771 | NUDC | protein_coding | ENSBTAG00000004416.4 | nuclear distribution C, dynein complex regulator [Source:VGNC Symbol;Acc:VGNC:32321] |
| 2 | 126,303,160 | 126,305,471 | NR0B2 | protein_coding | ENSBTAG00000014848.4 | nuclear receptor subfamily 0 group B member 2 [Source:VGNC Symbol;Acc:VGNC:32227] |
| 2 | 126,314,664 | 126,321,490 | GPATCH3 | protein_coding | ENSBTAG00000014849.4 | G-patch domain containing 3 [Source:VGNC Symbol;Acc:VGNC:29518] |
| 2 | 126,320,997 | 126,333,342 | GPN2 | protein_coding | ENSBTAG00000014850.4 | GPN-loop GTPase 2 [Source:VGNC Symbol;Acc:VGNC:29539] |
| 2 | 126,340,193 | 126,341,536 | SFN | protein_coding | ENSBTAG00000009223.5 | stratifin [Source:VGNC Symbol;Acc:VGNC:34517] |
| 2 | 126,349,905 | 126,374,294 | ZDHHC18 | protein_coding | ENSBTAG00000046672.2 | zinc finger DHHC-type palmitoyltransferase 18 [Source:VGNC Symbol;Acc:VGNC:37133] |
| 2 | 126,392,028 | 126,402,241 | PIGV | protein_coding | ENSBTAG00000009796.6 | phosphatidylinositol glycan anchor biosynthesis class V [Source:VGNC Symbol;Acc:VGNC:32880] |
| 2 | 126,408,497 | 126,477,461 | ARID1A | protein_coding | ENSBTAG00000001024.6 | AT-rich interaction domain 1A [Source:VGNC Symbol;Acc:VGNC:26123] |
| 2 | 126,574,904 | 126,612,862 | RPS6KA1 | protein_coding | ENSBTAG00000014447.5 | ribosomal protein S6 kinase A1 [Source:VGNC Symbol;Acc:VGNC:34140] |
| 2 | 126,666,827 | 126,704,429 | DHDDS | protein_coding | ENSBTAG00000003235.6 | dehydrodolichyl diphosphate synthase subunit [Source:VGNC Symbol;Acc:VGNC:28035] |
| 2 | 126,708,949 | 126,720,207 | LIN28A | protein_coding | ENSBTAG00000040497.2 | lin-28 homolog A [Source:NCBI gene (formerly Entrezgene);Acc:614997] |
| 2 | 126,841,741 | 126,876,537 | CEP85 | protein_coding | ENSBTAG00000009579.5 | centrosomal protein 85 [Source:VGNC Symbol;Acc:VGNC:27216] |
| 2 | 126,896,197 | 126,910,286 | CATSPER4 | protein_coding | ENSBTAG00000026638.5 | cation channel sperm associated 4 [Source:VGNC Symbol;Acc:VGNC:55330] |
| 2 | 126,911,248 | 126,923,367 | CNKSR1 | protein_coding | ENSBTAG00000019399.6 | connector enhancer of kinase suppressor of Ras 1 [Source:VGNC Symbol;Acc:VGNC:97254] |
| 2 | 126,928,734 | 126,929,203 | ZNF593OS | protein_coding | ENSBTAG00000050680.1 | ZNF593 opposite strand [Source:HGNC Symbol;Acc:HGNC:41278] |
| 2 | 126,929,533 | 126,930,613 | ZNF593 | protein_coding | ENSBTAG00000009562.6 | zinc finger protein 593 [Source:VGNC Symbol;Acc:VGNC:56158] |
| 2 | 126,936,277 | 126,939,832 | FAM110D | protein_coding | ENSBTAG00000013131.6 | family with sequence similarity 110 member D [Source:VGNC Symbol;Acc:VGNC:54426] |
| 2 | 126,966,876 | 126,975,053 | PDIK1L | protein_coding | ENSBTAG00000001513.4 | PDLIM1 interacting kinase 1 like [Source:VGNC Symbol;Acc:VGNC:32701] |
| 2 | 127,020,869 | 127,035,929 | TRIM63 | protein_coding | ENSBTAG00000005085.5 | tripartite motif containing 63 [Source:VGNC Symbol;Acc:VGNC:36346] |
| 2 | 127,042,911 | 127,050,544 | SLC30A2 | protein_coding | ENSBTAG00000006355.6 | solute carrier family 30 member 2 [Source:VGNC Symbol;Acc:VGNC:34807] |
| 2 | 127,052,889 | 127,070,881 | EXTL1 | protein_coding | ENSBTAG00000006349.5 | exostosin like glycosyltransferase 1 [Source:VGNC Symbol;Acc:VGNC:28667] |
| 2 | 127,089,778 | 127,125,126 | PAFAH2 | protein_coding | ENSBTAG00000005105.6 | platelet activating factor acetylhydrolase 2 [Source:VGNC Symbol;Acc:VGNC:32552] |
| 2 | 127,152,813 | 127,183,806 | STMN1 | protein_coding | ENSBTAG00000013761.4 | stathmin 1 [Source:NCBI gene (formerly Entrezgene);Acc:616317] |
| 2 | 127,217,390 | 127,222,938 | PAQR7 | protein_coding | ENSBTAG00000021787.3 | progestin and adipoQ receptor family member 7 [Source:VGNC Symbol;Acc:VGNC:32579] |
| 2 | 127,225,459 | 127,237,892 | AUNIP | protein_coding | ENSBTAG00000021786.4 | aurora kinase A and ninein interacting protein [Source:VGNC Symbol;Acc:VGNC:26347] |
| 2 | 127,238,786 | 127,253,787 | MTFR1L | protein_coding | ENSBTAG00000021781.6 | mitochondrial fission regulator 1 like [Source:VGNC Symbol;Acc:VGNC:31727] |
| 2 | 127,256,063 | 127,272,993 | SELENON | protein_coding | ENSBTAG00000021778.6 | selenoprotein N [Source:VGNC Symbol;Acc:VGNC:56142] |
| 2 | 127,292,652 | 127,444,339 | MAN1C1 | protein_coding | ENSBTAG00000003069.7 | mannosidase alpha class 1C member 1 [Source:VGNC Symbol;Acc:VGNC:31163] |
| 2 | 127,482,749 | 127,508,799 | LDLRAP1 | protein_coding | ENSBTAG00000001050.6 | low density lipoprotein receptor adaptor protein 1 [Source:VGNC Symbol;Acc:VGNC:30830] |
| 2 | 127,542,968 | 127,609,384 | MACO1 | protein_coding | ENSBTAG00000002988.6 | macoilin 1 [Source:VGNC Symbol;Acc:VGNC:36098] |
| 2 | 128,924,106 | 128,936,367 | SRSF10 | protein_coding | ENSBTAG00000008072.6 | serine and arginine rich splicing factor 10 [Source:VGNC Symbol;Acc:VGNC:35298] |
| 2 | 128,938,566 | 128,942,609 | PNRC2 | protein_coding | ENSBTAG00000030435.2 | proline rich nuclear receptor coactivator 2 [Source:VGNC Symbol;Acc:VGNC:33099] |
| 2 | 129,001,537 | 129,038,262 | CNR2 | protein_coding | ENSBTAG00000019371.5 | cannabinoid receptor 2 [Source:VGNC Symbol;Acc:VGNC:27530] |
| 2 | 129,056,428 | 129,079,502 | FUCA1 | protein_coding | ENSBTAG00000030434.2 | alpha-L-fucosidase 1 [Source:VGNC Symbol;Acc:VGNC:29140] |
| 2 | 129,091,481 | 129,110,145 | HMGCL | protein_coding | ENSBTAG00000021832.4 | 3-hydroxy-3-methylglutaryl-CoA lyase [Source:VGNC Symbol;Acc:VGNC:29877] |
| 2 | 129,111,017 | 129,115,576 | GALE | protein_coding | ENSBTAG00000005002.3 | UDP-galactose-4-epimerase [Source:VGNC Symbol;Acc:VGNC:29218] |
| 2 | 129,115,781 | 129,121,108 | LYPLA2 | protein_coding | ENSBTAG00000011625.6 | lysophospholipase 2 [Source:VGNC Symbol;Acc:VGNC:31106] |
| 2 | 129,122,974 | 129,130,667 | PITHD1 | protein_coding | ENSBTAG00000021620.5 | PITH domain containing 1 [Source:VGNC Symbol;Acc:VGNC:32918] |
| 2 | 129,146,487 | 129,146,577 | bta-mir-10181 | miRNA | ENSBTAG00000049982.1 | bta-mir-10181 [Source:miRBase;Acc:MI0032940] |
| 2 | 129,147,034 | 129,156,032 | ELOA | protein_coding | ENSBTAG00000026585.5 | elongin A [Source:VGNC Symbol;Acc:VGNC:56105] |
| 2 | 129,168,191 | 129,198,058 | RPL11 | protein_coding | ENSBTAG00000020905.4 | ribosomal protein L11 [Source:VGNC Symbol;Acc:VGNC:34108] |
| 2 | 129,296,772 | 129,298,371 | ID3 | protein_coding | ENSBTAG00000030425.3 | inhibitor of DNA binding 3, HLH protein [Source:VGNC Symbol;Acc:VGNC:30032] |
| 2 | 129,326,862 | 129,343,702 | E2F2 | protein_coding | ENSBTAG00000014400.6 | E2F transcription factor 2 [Source:VGNC Symbol;Acc:VGNC:54420] |
| 2 | 129,367,152 | 129,417,868 | ASAP3 | protein_coding | ENSBTAG00000012263.6 | ArfGAP with SH3 domain, ankyrin repeat and PH domain 3 [Source:VGNC Symbol;Acc:VGNC:54406] |
| 2 | 129,421,761 | 129,471,183 | TCEA3 | protein_coding | ENSBTAG00000038865.3 | transcription elongation factor A3 [Source:VGNC Symbol;Acc:VGNC:35681] |
| 2 | 134,985,221 | 135,154,958 | ARHGEF10L | protein_coding | ENSBTAG00000000684.6 | Rho guanine nucleotide exchange factor 10 like [Source:VGNC Symbol;Acc:VGNC:26105] |
| 3 | 83,258,392 | 83,334,028 | KANK4 | protein_coding | ENSBTAG00000014382.6 | KN motif and ankyrin repeat domains 4 [Source:VGNC Symbol;Acc:VGNC:55243] |
| 3 | 84,203,809 | 84,620,790 | NFIA | protein_coding | ENSBTAG00000000074.5 | nuclear factor I A [Source:VGNC Symbol;Acc:VGNC:32038] |
| 3 | 85,428,412 | 85,428,575 | U1 | snRNA | ENSBTAG00000051869.1 | U1 spliceosomal RNA [Source:RFAM;Acc:RF00003] |
| 3 | 85,704,934 | 85,786,712 | C3H1orf87 | protein_coding | ENSBTAG00000009481.6 | chromosome 3 C1orf87 homolog [Source:VGNC Symbol;Acc:VGNC:52717] |
| 3 | 88,677,121 | 89,136,086 | DAB1 | protein_coding | ENSBTAG00000013802.5 | DAB adaptor protein 1 [Source:VGNC Symbol;Acc:VGNC:27864] |
| 3 | 94,334,017 | 94,401,951 | RAB3B | protein_coding | ENSBTAG00000005337.5 | RAB3B, member RAS onco family [Source:VGNC Symbol;Acc:VGNC:33650] |
| 3 | 94,437,449 | 94,540,296 | NRDC | protein_coding | ENSBTAG00000021174.5 | nardilysin convertase [Source:VGNC Symbol;Acc:VGNC:32258] |
| 3 | 94,462,220 | 94,462,295 | bta-mir-761 | miRNA | ENSBTAG00000036394.2 | bta-mir-761 [Source:miRBase;Acc:MI0009895] |
| 3 | 94,541,126 | 94,702,613 | OSBPL9 | protein_coding | ENSBTAG00000021170.6 | oxysterol binding protein like 9 [Source:VGNC Symbol;Acc:VGNC:32467] |
| 3 | 94,548,208 | 94,548,265 | bta-mir-11991 | miRNA | ENSBTAG00000049085.1 | bta-mir-11991 [Source:miRBase;Acc:MI0038355] |
| 3 | 95,140,835 | 95,186,049 | C3H1orf185 | protein_coding | ENSBTAG00000009674.5 | chromosome 3 C1orf185 homolog [Source:VGNC Symbol;Acc:VGNC:54905] |
| 3 | 95,337,691 | 95,342,769 | CDKN2C | protein_coding | ENSBTAG00000011059.4 | cyclin dependent kinase inhibitor 2C [Source:VGNC Symbol;Acc:VGNC:27146] |
| 3 | 95,351,469 | 95,849,104 | FAF1 | protein_coding | ENSBTAG00000014482.4 | Fas associated factor 1 [Source:VGNC Symbol;Acc:VGNC:28705] |
| 3 | 96,071,533 | 96,166,159 | ELAVL4 | protein_coding | ENSBTAG00000021046.6 | ELAV like RNA binding protein 4 [Source:VGNC Symbol;Acc:VGNC:52769] |
| 3 | 97,468,115 | 97,518,125 | BEND5 | protein_coding | ENSBTAG00000009209.5 | BEN domain containing 5 [Source:VGNC Symbol;Acc:VGNC:26468] |
| 3 | 97,776,435 | 97,942,867 | SPATA6 | protein_coding | ENSBTAG00000003128.6 | spermatosis associated 6 [Source:VGNC Symbol;Acc:VGNC:35189] |
| 3 | 97,963,204 | 98,001,735 | SLC5A9 | protein_coding | ENSBTAG00000014225.6 | solute carrier family 5 member 9 [Source:VGNC Symbol;Acc:VGNC:34912] |
| 3 | 98,066,623 | 98,090,010 | SKINT1 | protein_coding | ENSBTAG00000009192.6 | selection and upkeep of intraepithelial T cells 1 [Source:NCBI gene (formerly Entrezgene);Acc:528184] |
| 3 | 98,278,655 | 98,494,949 | TRABD2B | protein_coding | ENSBTAG00000054324.1 | TraB domain containing 2B [Source:VGNC Symbol;Acc:VGNC:106996] |
| 3 | 98,790,856 | 98,790,963 | 5S_rRNA | rRNA | ENSBTAG00000028255.2 | 5S ribosomal RNA [Source:RFAM;Acc:RF00001] |
| 3 | 98,832,725 | 98,834,185 | FOXD2 | protein_coding | ENSBTAG00000045643.2 | forkhead box D2 [Source:VGNC Symbol;Acc:VGNC:55192] |
| 3 | 98,850,503 | 98,852,477 | FOXE3 | protein_coding | ENSBTAG00000051575.1 | forkhead box E3 [Source:VGNC Symbol;Acc:VGNC:106745] |
| 3 | 98,887,940 | 98,924,294 | CMPK1 | protein_coding | ENSBTAG00000019956.4 | cytidine/uridine monophosphate kinase 1 [Source:VGNC Symbol;Acc:VGNC:27480] |
| 3 | 98,940,131 | 98,990,375 | STIL | protein_coding | ENSBTAG00000017844.6 | STIL centriolar assembly protein [Source:VGNC Symbol;Acc:VGNC:35382] |
| 3 | 99,005,834 | 99,022,661 | TAL1 | protein_coding | ENSBTAG00000005029.6 | TAL bHLH transcription factor 1, erythroid differentiation factor [Source:VGNC Symbol;Acc:VGNC:35588] |
| 3 | 99,025,649 | 99,050,629 | PDZK1IP1 | protein_coding | ENSBTAG00000002555.4 | PDZK1 interacting protein 1 [Source:VGNC Symbol;Acc:VGNC:32730] |
| 3 | 99,069,008 | 99,097,597 | CYP4X1 | protein_coding | ENSBTAG00000015647.6 | cytochrome P450 family 4 subfamily X member 1 [Source:HGNC Symbol;Acc:HGNC:20244] |
| 3 | 99,208,577 | 99,222,625 | CYP4A11 | protein_coding | ENSBTAG00000037890.3 | cytochrome P450, family 4, subfamily A, polypeptide 11 [Source:NCBI gene (formerly Entrezgene);Acc:511890] |
| 3 | 99,319,612 | 99,334,690 | CYP4A22 | protein_coding | ENSBTAG00000013481.6 | cytochrome P450, family 4, subfamily A, polypeptide 22 [Source:NCBI gene (formerly Entrezgene);Acc:516085] |
| 3 | 99,337,603 | 99,358,612 | CYP4B1 | protein_coding | ENSBTAG00000011976.5 | cytochrome P450, family 4, subfamily B, polypeptide 1 [Source:NCBI gene (formerly Entrezgene);Acc:540149] |
| 3 | 111,603,940 | 112,289,188 | CSMD2 | protein_coding | ENSBTAG00000005784.6 | CUB and Sushi multiple domains 2 [Source:VGNC Symbol;Acc:VGNC:27762] |
| 3 | 111,927,003 | 111,927,727 | HMGB4 | protein_coding | ENSBTAG00000000335.5 | high mobility group box 4 [Source:VGNC Symbol;Acc:VGNC:29876] |
| 5 | 98,558,817 | 98,559,755 | TAS2R42 | protein_coding | ENSBTAG00000030472.2 | taste receptor, type 2, member 42 [Source:NCBI gene (formerly Entrezgene);Acc:664646] |
| 5 | 98,571,197 | 98,571,403 | SMIM10L1 | protein_coding | ENSBTAG00000052681.1 | small integral membrane protein 10 like 1 [Source:VGNC Symbol;Acc:VGNC:106936] |
| 5 | 98,588,763 | 98,589,680 | TAS2R46 | protein_coding | ENSBTAG00000030471.2 | taste receptor, type 2, member 46 [Source:NCBI gene (formerly Entrezgene);Acc:664639] |
| 5 | 98,625,559 | 98,626,446 | T2R65A | protein_coding | ENSBTAG00000023258.3 | bitter taste receptor Bota-T2R65A [Source:NCBI gene (formerly Entrezgene);Acc:664647] |
| 5 | 98,749,622 | 98,750,521 | TAS2R10 | protein_coding | ENSBTAG00000030463.2 | taste receptor, type 2, member 10 [Source:NCBI gene (formerly Entrezgene);Acc:664636] |
| 5 | 98,761,040 | 98,761,969 | T2R10C | protein_coding | ENSBTAG00000030461.2 | bitter taste receptor Bota-T2R10C [Source:NCBI gene (formerly Entrezgene);Acc:664644] |
| 5 | 98,773,684 | 98,779,676 | TAS2R8 | protein_coding | ENSBTAG00000048398.1 | taste 2 receptor member 8 [Source:VGNC Symbol;Acc:VGNC:106967] |
| 5 | 98,781,269 | 98,785,838 | TAS2R7 | protein_coding | ENSBTAG00000051374.1 | taste 2 receptor member 7 [Source:VGNC Symbol;Acc:VGNC:109397] |
| 5 | 98,853,392 | 98,878,560 | YBX3 | protein_coding | ENSBTAG00000009663.5 | Y-box binding protein 3 [Source:VGNC Symbol;Acc:VGNC:37018] |
| 5 | 98,891,931 | 98,945,941 | STYK1 | protein_coding | ENSBTAG00000015351.5 | serine/threonine/tyrosine kinase 1 [Source:VGNC Symbol;Acc:VGNC:35451] |
| 5 | 99,603,137 | 99,613,790 | KLRK1 | protein_coding | ENSBTAG00000004917.6 | killer cell lectin-like receptor subfamily K, member 1 [Source:NCBI gene (formerly Entrezgene);Acc:404058] |
| 5 | 99,655,608 | 99,660,385 | KLRD1 | protein_coding | ENSBTAG00000046389.2 | killer cell lectin-like receptor subfamily D, member 1 [Source:NCBI gene (formerly Entrezgene);Acc:444877] |
| 5 | 99,764,511 | 99,773,990 | GABARAPL1 | protein_coding | ENSBTAG00000011765.6 | GABA type A receptor associated protein like 1 [Source:VGNC Symbol;Acc:VGNC:56201] |
| 5 | 99,782,216 | 99,792,430 | TMEM52B | protein_coding | ENSBTAG00000011023.5 | transmembrane protein 52B [Source:VGNC Symbol;Acc:VGNC:36095] |
| 5 | 99,803,497 | 99,815,138 | OLR1 | protein_coding | ENSBTAG00000004547.4 | oxidized low density lipoprotein receptor 1 [Source:VGNC Symbol;Acc:VGNC:32427] |
| 5 | 99,833,307 | 99,860,241 | CLEC7A | protein_coding | ENSBTAG00000014546.6 | C-type lectin domain containing 7A [Source:VGNC Symbol;Acc:VGNC:58597] |
| 5 | 99860312 | 99,880,739 | CLEC1A | protein_coding | ENSBTAG00000030424.4 | C-type lectin domain family 1 member A [Source:VGNC Symbol;Acc:VGNC:27427] |
| 5 | 99,887,314 | 99,899,431 | CLEC9A | protein_coding | ENSBTAG00000009537.6 | C-type lectin domain containing 9A [Source:VGNC Symbol;Acc:VGNC:58367] |
| 5 | 99,905,264 | 99,936,312 | CLEC1B | protein_coding | ENSBTAG00000030423.4 | C-type lectin domain family 1 member B [Source:VGNC Symbol;Acc:VGNC:58366] |
| 5 | 99,926,493 | 99,932,833 | CLEC12B | protein_coding | ENSBTAG00000004293.6 | C-type lectin domain family 12 member B [Source:VGNC Symbol;Acc:VGNC:27424] |
| 5 | 99,947,296 | 99,960,045 | CLEC12A | protein_coding | ENSBTAG00000012815.6 | C-type lectin domain family 12 member A [Source:VGNC Symbol;Acc:VGNC:27423] |
| 5 | 100,504,892 | 100,630,214 | KLRB1 | protein_coding | ENSBTAG00000012029.6 | killer cell lectin like receptor B1 [Source:VGNC Symbol;Acc:VGNC:30684] |
| 5 | 100,679,985 | 100,732,005 | OVOS2 | protein_coding | ENSBTAG00000005243.6 | ovostatin 2 [Source:NCBI gene (formerly Entrezgene);Acc:508026] |
| 5 | 100,873,612 | 100,891,039 | A2M | protein_coding | ENSBTAG00000018137.6 | alpha-2-macroglobulin [Source:VGNC Symbol;Acc:VGNC:55837] |
| 5 | 100,946,808 | 100,964,339 | KLRG1 | protein_coding | ENSBTAG00000013640.5 | killer cell lectin like receptor G1 [Source:VGNC Symbol;Acc:VGNC:30687] |
| 5 | 100,984,954 | 100,998,031 | M6PR | protein_coding | ENSBTAG00000018207.4 | mannose-6-phosphate receptor, cation dependent [Source:VGNC Symbol;Acc:VGNC:31125] |
| 5 | 100,997,211 | 101,017,135 | PHC1 | protein_coding | ENSBTAG00000018205.6 | polyhomeotic homolog 1 [Source:VGNC Symbol;Acc:VGNC:32810] |
| 5 | 101,041,433 | 101,090,609 | A2ML1 | protein_coding | ENSBTAG00000004097.6 | alpha-2-macroglobulin like 1 [Source:VGNC Symbol;Acc:VGNC:25435] |
| 5 | 101,087,152 | 101,087,214 | bta-mir-2284r | miRNA | ENSBTAG00000044623.2 | bta-mir-2284r [Source:miRBase;Acc:MI0011488] |
| 5 | 101,125,927 | 101,163,364 | RIMKLB | protein_coding | ENSBTAG00000003291.6 | ribosomal modification protein rimK like family member B [Source:VGNC Symbol;Acc:VGNC:33971] |
| 5 | 101,190,160 | 101,203,715 | MFAP5 | protein_coding | ENSBTAG00000000310.6 | microfibril associated protein 5 [Source:VGNC Symbol;Acc:VGNC:31425] |
| 5 | 101,224,468 | 101,234,839 | AICDA | protein_coding | ENSBTAG00000018849.6 | activation induced cytidine deaminase [Source:VGNC Symbol;Acc:VGNC:25757] |
| 5 | 101,268,561 | 101,274,410 | APOBEC1 | protein_coding | ENSBTAG00000014683.5 | apolipoprotein B mRNA editing enzyme catalytic subunit 1 [Source:VGNC Symbol;Acc:VGNC:26027] |
| 7 | 16,245,817 | 16,345,949 | ARHGEF18 | protein_coding | ENSBTAG00000040507.3 | Rho/Rac guanine nucleotide exchange factor 18 [Source:VGNC Symbol;Acc:VGNC:106426] |
| 7 | 16,347,652 | 16,361,803 | PEX11G | protein_coding | ENSBTAG00000018894.6 | peroxisomal biosis factor 11 gamma [Source:VGNC Symbol;Acc:VGNC:32753] |
| 7 | 16,368,804 | 16,373,988 | TEX45 | protein_coding | ENSBTAG00000002629.4 | testis expressed 45 [Source:VGNC Symbol;Acc:VGNC:52885] |
| 7 | 16,386,268 | 16,388,881 | ZNF358 | protein_coding | ENSBTAG00000046837.2 | zinc finger protein 358 [Source:VGNC Symbol;Acc:VGNC:37257] |
| 7 | 16,390,698 | 16,399,664 | MCOLN1 | protein_coding | ENSBTAG00000005592.6 | mucolipin TRP cation channel 1 [Source:VGNC Symbol;Acc:VGNC:31318] |
| 7 | 16,399,795 | 16,424,614 | PNPLA6 | protein_coding | ENSBTAG00000032137.4 | patatin like phospholipase domain containing 6 [Source:VGNC Symbol;Acc:VGNC:33093] |
| 7 | 16,447,830 | 16,464,459 | CAMSAP3 | protein_coding | ENSBTAG00000011356.4 | calmodulin regulated spectrin associated protein family member 3 [Source:VGNC Symbol;Acc:VGNC:26731] |
| 7 | 16,450,200 | 16,474,545 | XAB2 | protein_coding | ENSBTAG00000011360.6 | XPA binding protein 2 [Source:VGNC Symbol;Acc:VGNC:36981] |
| 7 | 16,474,631 | 16,476,389 | PET100 | protein_coding | ENSBTAG00000048824.1 | PET100 cytochrome c oxidase chaperone [Source:HGNC Symbol;Acc:HGNC:40038] |
| 7 | 16,476,406 | 16,477,872 | PCP2 | protein_coding | ENSBTAG00000008906.5 | Purkinje cell protein 2 [Source:VGNC Symbol;Acc:VGNC:32644] |
| 7 | 16,482,854 | 16,491,641 | STXBP2 | protein_coding | ENSBTAG00000009178.4 | syntaxin binding protein 2 [Source:VGNC Symbol;Acc:VGNC:35446] |
| 7 | 17,557,794 | 17,633,496 | ADGRE1 | protein_coding | ENSBTAG00000007901.6 | adhesion G protein-coupled receptor E1 [Source:HGNC Symbol;Acc:HGNC:3336] |
| 7 | 18,141,671 | 18,210,109 | MLLT1 | protein_coding | ENSBTAG00000002277.6 | MLLT1 super elongation complex subunit [Source:VGNC Symbol;Acc:VGNC:31501] |
| 7 | 18,256,064 | 18,269,611 | ACSBG2 | protein_coding | ENSBTAG00000009105.6 | acyl-CoA synthetase bubblegum family member 2 [Source:VGNC Symbol;Acc:VGNC:25561] |
| 7 | 18,305,239 | 18,398,128 | RFX2 | protein_coding | ENSBTAG00000017661.6 | regulatory factor X2 [Source:VGNC Symbol;Acc:VGNC:33894] |
| 7 | 18,414,908 | 18,464,857 | RANBP3 | protein_coding | ENSBTAG00000006070.5 | RAN binding protein 3 [Source:VGNC Symbol;Acc:VGNC:33710] |
| 7 | 18,466,099 | 18,467,633 | CAPS | protein_coding | ENSBTAG00000006069.4 | calcyphosine [Source:VGNC Symbol;Acc:VGNC:26753] |
| 7 | 18,469,286 | 18,472,439 | VMAC | protein_coding | ENSBTAG00000006067.4 | vimentin type intermediate filament associated coiled-coil protein [Source:VGNC Symbol;Acc:VGNC:97326] |
| 7 | 18,473,539 | 18,479,129 | NDUFA11 | protein_coding | ENSBTAG00000019025.4 | NADH:ubiquinone oxidoreductase subunit A11 [Source:NCBI gene (formerly Entrezgene);Acc:326346] |
| 7 | 18,515,026 | 18,516,457 | FUT6 | protein_coding | ENSBTAG00000000414.6 | fucosyltransferase 6 [Source:NCBI gene (formerly Entrezgene);Acc:338077] |
| 7 | 18,518,580 | 18,532,314 | NRTN | protein_coding | ENSBTAG00000000413.5 | neurturin [Source:VGNC Symbol;Acc:VGNC:32270] |
| 7 | 18,541,354 | 18,546,361 | DUS3L | protein_coding | ENSBTAG00000011842.6 | dihydrouridine synthase 3 like [Source:VGNC Symbol;Acc:VGNC:103057] |
| 7 | 18,545,676 | 18,548,664 | PRR22 | protein_coding | ENSBTAG00000025550.3 | proline rich 22 [Source:VGNC Symbol;Acc:VGNC:52867] |
| 7 | 18,552,769 | 18,589,512 | CATSPERD | protein_coding | ENSBTAG00000001796.6 | cation channel sperm associated auxiliary subunit delta [Source:VGNC Symbol;Acc:VGNC:26797] |
| 7 | 18,590,319 | 18,608,816 | LONP1 | protein_coding | ENSBTAG00000001795.6 | lon peptidase 1, mitochondrial [Source:VGNC Symbol;Acc:VGNC:30947] |
| 7 | 18,608,950 | 18,610,219 | RPL36 | protein_coding | ENSBTAG00000001794.3 | ribosomal protein L36 [Source:NCBI gene (formerly Entrezgene);Acc:768327] |
| 7 | 18,611,628 | 18,616,460 | HSD11B1L | protein_coding | ENSBTAG00000001793.4 | hydroxysteroid 11-beta dehydrogenase 1 like [Source:VGNC Symbol;Acc:VGNC:29966] |
| 7 | 18,617,143 | 18,618,572 | MICOS13 | protein_coding | ENSBTAG00000001792.5 | mitochondrial contact site and cristae organizing system subunit 13 [Source:VGNC Symbol;Acc:VGNC:52730] |
| 7 | 18,621,612 | 18,651,390 | SAFB | protein_coding | ENSBTAG00000007875.6 | scaffold attachment factor B [Source:NCBI gene (formerly Entrezgene);Acc:539785] |
| 7 | 18,682,927 | 18,711,822 | SAFB2 | protein_coding | ENSBTAG00000001790.6 | scaffold attachment factor B2 [Source:VGNC Symbol;Acc:VGNC:34262] |
| 7 | 18,835,671 | 18,838,466 | ZNRF4 | protein_coding | ENSBTAG00000051350.1 | zinc and ring finger 4 [Source:VGNC Symbol;Acc:VGNC:37365] |
| 7 | 18,936,414 | 19,046,578 | PTPRS | protein_coding | ENSBTAG00000018052.5 | protein tyrosine phosphatase receptor type S [Source:VGNC Symbol;Acc:VGNC:33558] |
| 7 | 19,096,391 | 19,175,953 | KDM4B | protein_coding | ENSBTAG00000015487.5 | lysine demethylase 4B [Source:VGNC Symbol;Acc:VGNC:30528] |
| 7 | 19,223,877 | 19,257,053 | UHRF1 | protein_coding | ENSBTAG00000002224.6 | ubiquitin like with PHD and ring finger domains 1 [Source:VGNC Symbol;Acc:VGNC:36654] |
| 7 | 19,261,039 | 19,274,362 | ARRDC5 | protein_coding | ENSBTAG00000031673.2 | arrestin domain containing 5 [Source:VGNC Symbol;Acc:VGNC:26175] |
| 7 | 19,291,174 | 19,311,897 | PLIN3 | protein_coding | ENSBTAG00000011805.6 | perilipin 3 [Source:VGNC Symbol;Acc:VGNC:33031] |
| 7 | 19,335,555 | 19,337,855 | TICAM1 | pseudogene | ENSBTAG00000019966.6 | toll like receptor adaptor molecule 1 [Source:VGNC Symbol;Acc:VGNC:52887] |
| 7 | 19,353,553 | 19,357,132 | FEM1A | protein_coding | ENSBTAG00000003476.6 | fem-1 homolog A [Source:VGNC Symbol;Acc:VGNC:28944] |
| 7 | 19,372,417 | 19,372,498 | bta-mir-7-3 | miRNA | ENSBTAG00000029968.2 | bta-mir-7-3 [Source:miRBase;Acc:MI0005056] |
| 7 | 19,428,848 | 19,469,011 | DPP9 | protein_coding | ENSBTAG00000021134.6 | dipeptidyl peptidase 9 [Source:VGNC Symbol;Acc:VGNC:28190] |
| 7 | 19,470,114 | 19,482,055 | MYDGF | protein_coding | ENSBTAG00000018655.4 | myeloid derived growth factor [Source:VGNC Symbol;Acc:VGNC:31790] |
| 7 | 19,483,938 | 19,503,011 | TNFAIP8L1 | protein_coding | ENSBTAG00000037765.3 | TNF alpha induced protein 8 like 1 [Source:VGNC Symbol;Acc:VGNC:36158] |
| 7 | 19,542,123 | 19,542,178 | bta-mir-2285by | miRNA | ENSBTAG00000053768.1 | bta-mir-2285by [Source:miRBase;Acc:MI0038369] |
| 7 | 19,574,478 | 19,584,168 | SEMA6B | protein_coding | ENSBTAG00000031658.4 | semaphorin 6B [Source:VGNC Symbol;Acc:VGNC:34442] |
| 7 | 19,596,244 | 19,599,321 | LRG1 | protein_coding | ENSBTAG00000031647.2 | leucine rich alpha-2-glycoprotein 1 [Source:VGNC Symbol;Acc:VGNC:30980] |
| 7 | 19,600,448 | 19,611,770 | PLIN5 | protein_coding | ENSBTAG00000038464.3 | perilipin 5 [Source:VGNC Symbol;Acc:VGNC:33032] |
| 7 | 19,614,545 | 19,626,137 | PLIN4 | protein_coding | ENSBTAG00000015690.6 | perilipin 4 [Source:VGNC Symbol;Acc:VGNC:55299] |
| 7 | 19,627,416 | 19,645,495 | HDGFL2 | protein_coding | ENSBTAG00000013251.6 | HDGF like 2 [Source:VGNC Symbol;Acc:VGNC:109383] |
| 7 | 19,663,055 | 19,674,553 | UBXN6 | protein_coding | ENSBTAG00000008186.5 | UBX domain protein 6 [Source:VGNC Symbol;Acc:VGNC:36628] |
| 7 | 19,676,722 | 19,701,793 | CHAF1A | protein_coding | ENSBTAG00000008181.6 | chromatin assembly factor 1 subunit A [Source:VGNC Symbol;Acc:VGNC:27266] |
| 7 | 19,706,831 | 19,734,112 | SH3GL1 | protein_coding | ENSBTAG00000006007.4 | SH3 domain containing GRB2 like 1, endophilin A2 [Source:VGNC Symbol;Acc:VGNC:34572] |
| 7 | 19,734,266 | 19,744,478 | MPND | protein_coding | ENSBTAG00000006005.6 | MPN domain containing [Source:VGNC Symbol;Acc:VGNC:31580] |
| 7 | 19,749,298 | 19,761,854 | STAP2 | protein_coding | ENSBTAG00000006001.6 | signal transducing adaptor family member 2 [Source:VGNC Symbol;Acc:VGNC:35359] |
| 7 | 19,761,502 | 19,774,023 | FSD1 | protein_coding | ENSBTAG00000005999.5 | fibronectin type III and SPRY domain containing 1 [Source:VGNC Symbol;Acc:VGNC:29129] |
| 7 | 19,775,155 | 19,782,514 | TMIGD2 | protein_coding | ENSBTAG00000004936.6 | transmembrane and immunoglobulin domain containing 2 [Source:VGNC Symbol;Acc:VGNC:36133] |
| 7 | 19,783,231 | 19,791,715 | SHD | protein_coding | ENSBTAG00000039696.4 | Src homology 2 domain containing transforming protein D [Source:VGNC Symbol;Acc:VGNC:34594] |
| 7 | 19,797,987 | 19,812,101 | YJU2 | protein_coding | ENSBTAG00000012830.6 | YJU2 splicing factor homolog [Source:VGNC Symbol;Acc:VGNC:26935] |
| 7 | 19,814,211 | 19,819,636 | EBI3 | protein_coding | ENSBTAG00000012829.5 | Epstein-Barr virus induced 3 [Source:NCBI gene (formerly Entrezgene);Acc:514933] |
| 7 | 19,856,911 | 19,865,253 | SIRT6 | protein_coding | ENSBTAG00000019909.6 | sirtuin 6 [Source:VGNC Symbol;Acc:VGNC:34635] |
| 7 | 19,866,004 | 19,877,349 | CREB3L3 | protein_coding | ENSBTAG00000010215.6 | cAMP responsive element binding protein 3 like 3 [Source:VGNC Symbol;Acc:VGNC:27696] |
| 7 | 19,909,945 | 19,932,216 | MAP2K2 | protein_coding | ENSBTAG00000024450.5 | mitogen-activated protein kinase kinase 2 [Source:VGNC Symbol;Acc:VGNC:55215] |
| 7 | 19,956,235 | 19,976,863 | ZBTB7A | protein_coding | ENSBTAG00000020117.6 | zinc finger and BTB domain containing 7A [Source:VGNC Symbol;Acc:VGNC:37089] |
| 7 | 19,978,943 | 20,004,157 | PIAS4 | protein_coding | ENSBTAG00000000753.6 | protein inhibitor of activated STAT 4 [Source:VGNC Symbol;Acc:VGNC:32858] |
| 7 | 20,027,762 | 20,036,651 | EEF2 | protein_coding | ENSBTAG00000004258.4 | eukaryotic translation elongation factor 2 [Source:VGNC Symbol;Acc:VGNC:28335] |
| 7 | 20,030,713 | 20,030,785 | bta-mir-1434 | miRNA | ENSBTAG00000051833.1 | bta-mir-1434 [Source:miRBase;Acc:MI0011514] |
| 7 | 20,030,719 | 20,030,783 | SNORD37 | snoRNA | ENSBTAG00000042383.2 | small nucleolar RNA, C/D box 37 [Source:HGNC Symbol;Acc:HGNC:10166] |
| 7 | 20,040,198 | 20,053,609 | DAPK3 | protein_coding | ENSBTAG00000020417.4 | death associated protein kinase 3 [Source:VGNC Symbol;Acc:VGNC:27878] |
| 7 | 20,047,255 | 20,047,326 | bta-mir-2456 | miRNA | ENSBTAG00000045270.2 | bta-mir-2456 [Source:miRBase;Acc:MI0011515] |
| 7 | 20,064,778 | 20,069,191 | NMRK2 | protein_coding | ENSBTAG00000031573.3 | nicotinamide riboside kinase 2 [Source:VGNC Symbol;Acc:VGNC:106842] |
| 7 | 20,081,660 | 20,119,357 | ATCAY | protein_coding | ENSBTAG00000018375.5 | ATCAY kinesin light chain interacting caytaxin [Source:VGNC Symbol;Acc:VGNC:26237] |
| 7 | 20,132,959 | 20,178,566 | ZFR2 | protein_coding | ENSBTAG00000000755.5 | zinc finger RNA binding protein 2 [Source:VGNC Symbol;Acc:VGNC:37172] |
| 7 | 20,195,053 | 20,203,174 | MATK | protein_coding | ENSBTAG00000006619.6 | megakaryocyte-associated tyrosine kinase [Source:VGNC Symbol;Acc:VGNC:31263] |
| 7 | 20,206,334 | 20,209,157 | RAX2 | protein_coding | ENSBTAG00000009750.3 | retina and anterior neural fold homeobox 2 [Source:VGNC Symbol;Acc:VGNC:33767] |
| 7 | 20,209,218 | 20,212,341 | MRPL54 | protein_coding | ENSBTAG00000008396.6 | mitochondrial ribosomal protein L54 [Source:VGNC Symbol;Acc:VGNC:31649] |
| 7 | 20,214,159 | 20,223,118 | APBA3 | protein_coding | ENSBTAG00000008395.3 | amyloid beta protein binding family A member 3 [Source:VGNC Symbol;Acc:VGNC:26002] |
| 7 | 20,221,723 | 20,252,247 | TJP3 | protein_coding | ENSBTAG00000018342.4 | tight junction protein 3 [Source:VGNC Symbol;Acc:VGNC:35883] |
| 7 | 20,261,476 | 20,311,632 | PIP5K1C | protein_coding | ENSBTAG00000000710.7 | phosphatidylinositol-4-phosphate 5-kinase type 1 gamma [Source:VGNC Symbol;Acc:VGNC:32912] |
| 7 | 20,313,628 | 20,324,899 | CACTIN | protein_coding | ENSBTAG00000010373.5 | cactin, spliceosome C complex subunit [Source:VGNC Symbol;Acc:VGNC:58364] |
| 7 | 20,334,826 | 20,343,537 | TBXA2R | protein_coding | ENSBTAG00000014944.6 | thromboxane A2 receptor [Source:VGNC Symbol;Acc:VGNC:35673] |
| 7 | 20,346,684 | 20,350,042 | GIPC3 | protein_coding | ENSBTAG00000050809.1 | GIPC PDZ domain containing family member 3 [Source:NCBI gene (formerly Entrezgene);Acc:618265] |
| 7 | 20,353,044 | 20,358,586 | HMG20B | protein_coding | ENSBTAG00000008789.6 | high mobility group 20B [Source:NCBI gene (formerly Entrezgene);Acc:507723] |
| 7 | 20,369,007 | 20,378,072 | MFSD12 | protein_coding | ENSBTAG00000014979.6 | major facilitator superfamily domain containing 12 [Source:VGNC Symbol;Acc:VGNC:58388] |
| 7 | 20,379,062 | 20,382,521 | TEKTIP1 | protein_coding | ENSBTAG00000014982.6 | tektin bundle interacting protein 1 [Source:NCBI gene (formerly Entrezgene);Acc:768023] |
| 7 | 20,384,849 | 20,403,319 | FZR1 | protein_coding | ENSBTAG00000031387.2 | fizzy and cell division cycle 20 related 1 [Source:VGNC Symbol;Acc:VGNC:29173] |
| 7 | 20,418,396 | 20,426,869 | DOHH | protein_coding | ENSBTAG00000005272.6 | deoxyhypusine hydroxylase [Source:VGNC Symbol;Acc:VGNC:28164] |
| 7 | 20,433,050 | 20,436,476 | SMIM24 | protein_coding | ENSBTAG00000031383.4 | small integral membrane protein 24 [Source:VGNC Symbol;Acc:VGNC:35020] |
| 7 | 20,448,283 | 20,516,117 | NFIC | protein_coding | ENSBTAG00000008520.6 | nuclear factor I C [Source:VGNC Symbol;Acc:VGNC:32040] |
| 7 | 20,589,314 | 20,621,829 | CELF5 | protein_coding | ENSBTAG00000021774.6 | CUGBP Elav-like family member 5 [Source:VGNC Symbol;Acc:VGNC:55204] |
| 7 | 20,631,429 | 20,645,731 | NCLN | protein_coding | ENSBTAG00000020759.5 | nicalin [Source:VGNC Symbol;Acc:VGNC:31919] |
| 7 | 20,652,027 | 20,653,184 | S1PR4 | protein_coding | ENSBTAG00000000381.6 | sphingosine-1-phosphate receptor 4 [Source:VGNC Symbol;Acc:VGNC:34255] |
| 7 | 20,665,428 | 20,685,281 | GNA15 | protein_coding | ENSBTAG00000019988.6 | G protein subunit alpha 15 [Source:VGNC Symbol;Acc:VGNC:29448] |
| 7 | 20,694,784 | 20,713,444 | GNA11 | protein_coding | ENSBTAG00000012181.6 | G protein subunit alpha 11 [Source:VGNC Symbol;Acc:VGNC:29444] |
| 7 | 20,734,843 | 20,743,350 | TLE5 | protein_coding | ENSBTAG00000031363.4 | TLE family member 5, transcriptional modulator [Source:NCBI gene (formerly Entrezgene);Acc:505375] |
| 7 | 20,747,877 | 20,777,774 | TLE2 | protein_coding | ENSBTAG00000001153.6 | TLE family member 2, transcriptional corepressor [Source:VGNC Symbol;Acc:VGNC:49981] |
| 7 | 20,778,581 | 20,790,166 | TLE6 | protein_coding | ENSBTAG00000001152.5 | TLE family member 6, subcortical maternal complex member [Source:VGNC Symbol;Acc:VGNC:52895] |
| 7 | 20,847,764 | 20,853,001 | ZNF555 | protein_coding | ENSBTAG00000008397.5 | zinc finger protein 555 [Source:VGNC Symbol;Acc:VGNC:55316] |
| 7 | 20,868,563 | 20,873,708 | ZNF554 | protein_coding | ENSBTAG00000031352.4 | zinc finger protein 554 [Source:VGNC Symbol;Acc:VGNC:37301] |
| 7 | 20,886,519 | 20,901,121 | THOP1 | protein_coding | ENSBTAG00000020446.6 | thimet oligopeptidase 1 [Source:VGNC Symbol;Acc:VGNC:35843] |
| 7 | 20,903,069 | 20,917,593 | SGTA | protein_coding | ENSBTAG00000015090.6 | small glutamine rich tetratricopeptide repeat co-chaperone alpha [Source:VGNC Symbol;Acc:VGNC:34553] |
| 7 | 20,936,284 | 20,945,759 | SLC39A3 | protein_coding | ENSBTAG00000017706.3 | solute carrier family 39 member 3 [Source:VGNC Symbol;Acc:VGNC:52882] |
| 7 | 20,954,134 | 20,954,730 | DIRAS1 | protein_coding | ENSBTAG00000045981.2 | DIRAS family GTPase 1 [Source:VGNC Symbol;Acc:VGNC:28069] |
| 7 | 20,959,393 | 21,097,631 | GNG7 | protein_coding | ENSBTAG00000007644.4 | G protein subunit gamma 7 [Source:VGNC Symbol;Acc:VGNC:29468] |
| 7 | 21,118,031 | 21,120,298 | GADD45B | protein_coding | ENSBTAG00000025462.4 | growth arrest and DNA damage inducible beta [Source:VGNC Symbol;Acc:VGNC:29207] |
| 7 | 21,133,347 | 21,154,526 | LMNB2 | protein_coding | ENSBTAG00000013624.6 | lamin B2 [Source:VGNC Symbol;Acc:VGNC:30931] |
| 7 | 21,154,979 | 21,156,156 | TIMM13 | protein_coding | ENSBTAG00000013623.5 | translocase of inner mitochondrial membrane 13 [Source:VGNC Symbol;Acc:VGNC:52893] |
| 7 | 21,164,683 | 21,194,842 | TMPRSS9 | protein_coding | ENSBTAG00000000718.6 | transmembrane serine protease 9 [Source:VGNC Symbol;Acc:VGNC:36145] |
| 7 | 21,225,361 | 21,239,159 | SPPL2B | protein_coding | ENSBTAG00000004524.6 | signal peptide peptidase like 2B [Source:VGNC Symbol;Acc:VGNC:35235] |
| 7 | 21,239,214 | 21,243,937 | LSM7 | protein_coding | ENSBTAG00000004521.6 | LSM7 homolog, U6 small nuclear RNA and mRNA degradation associated [Source:VGNC Symbol;Acc:VGNC:31059] |
| 7 | 21,265,619 | 21,267,397 | LINGO3 | protein_coding | ENSBTAG00000016478.6 | leucine rich repeat and Ig domain containing 3 [Source:VGNC Symbol;Acc:VGNC:30901] |
| 7 | 21,274,513 | 21,280,458 | PEAK3 | protein_coding | ENSBTAG00000016477.4 | PEAK family member 3 [Source:VGNC Symbol;Acc:VGNC:49546] |
| 7 | 21,283,208 | 21,286,244 | OAZ1 | protein_coding | ENSBTAG00000018522.6 | ornithine decarboxylase antizyme 1 [Source:VGNC Symbol;Acc:VGNC:55296] |
| 7 | 21,334,365 | 21,386,441 | DOT1L | protein_coding | ENSBTAG00000009996.6 | DOT1 like histone lysine methyltransferase [Source:VGNC Symbol;Acc:VGNC:50607] |
| 7 | 21,389,365 | 21,391,998 | PLEKHJ1 | protein_coding | ENSBTAG00000001647.4 | pleckstrin homology domain containing J1 [Source:VGNC Symbol;Acc:VGNC:33023] |
| 7 | 21,392,211 | 21,401,682 | SF3A2 | protein_coding | ENSBTAG00000025452.5 | splicing factor 3a subunit 2 [Source:VGNC Symbol;Acc:VGNC:34507] |
| 7 | 21,401,483 | 21,404,348 | AMH | protein_coding | ENSBTAG00000014955.6 | anti-Mullerian hormone [Source:VGNC Symbol;Acc:VGNC:25864] |
| 7 | 21,404,529 | 21,408,418 | JSRP1 | protein_coding | ENSBTAG00000014956.6 | junctional sarcoplasmic reticulum protein 1 [Source:VGNC Symbol;Acc:VGNC:50101] |
| 7 | 21,413,767 | 21,447,278 | AP3D1 | protein_coding | ENSBTAG00000009034.6 | adaptor related protein complex 3 subunit delta 1 [Source:VGNC Symbol;Acc:VGNC:25988] |
| 7 | 21,448,720 | 21,451,824 | IZUMO4 | protein_coding | ENSBTAG00000025448.5 | IZUMO family member 4 [Source:VGNC Symbol;Acc:VGNC:30357] |
| 7 | 21,451,865 | 21,470,865 | MOB3A | protein_coding | ENSBTAG00000014526.5 | MOB kinase activator 3A [Source:VGNC Symbol;Acc:VGNC:31544] |
| 7 | 21,489,873 | 21,501,464 | MKNK2 | protein_coding | ENSBTAG00000018049.6 | MAPK interacting serine/threonine kinase 2 [Source:VGNC Symbol;Acc:VGNC:31492] |
| 7 | 94,029,771 | 94,398,651 | MCTP1 | protein_coding | ENSBTAG00000054976.1 | multiple C2 and transmembrane domain containing 1 [Source:VGNC Symbol;Acc:VGNC:106816] |
| 7 | 94,517,927 | 94,518,003 | bta-mir-2284z-7 | miRNA | ENSBTAG00000051715.1 | bta-mir-2284z-7 [Source:miRBase;Acc:MI0022341] |
| 7 | 94,687,293 | 94,744,875 | FAM81B | protein_coding | ENSBTAG00000019287.5 | family with sequence similarity 81 member B [Source:VGNC Symbol;Acc:VGNC:28831] |
| 7 | 95,035,646 | 95,120,697 | RHOBTB3 | protein_coding | ENSBTAG00000004413.6 | Rho related BTB domain containing 3 [Source:VGNC Symbol;Acc:VGNC:33946] |
| 7 | 95,122,128 | 95,132,776 | GLRX | protein_coding | ENSBTAG00000038186.2 | glutaredoxin [Source:NCBI gene (formerly Entrezgene);Acc:515416] |
| 7 | 95,212,712 | 95,286,507 | ELL2 | protein_coding | ENSBTAG00000007214.6 | elongation factor for RNA polymerase II 2 [Source:VGNC Symbol;Acc:VGNC:28436] |
| 7 | 96,033,978 | 96,167,151 | CAST | protein_coding | ENSBTAG00000000874.6 | calpastatin [Source:VGNC Symbol;Acc:VGNC:26790] |
| 7 | 96,168,529 | 96,204,132 | ERAP1 | protein_coding | ENSBTAG00000013557.5 | endoplasmic reticulum aminopeptidase 1 [Source:VGNC Symbol;Acc:VGNC:28560] |
| 7 | 96,303,404 | 96,356,159 | ERAP2 | protein_coding | ENSBTAG00000039275.3 | endoplasmic reticulum aminopeptidase 2 [Source:VGNC Symbol;Acc:VGNC:28561] |
| 7 | 96,371,532 | 96,466,676 | LNPEP | protein_coding | ENSBTAG00000019900.6 | leucyl and cystinyl aminopeptidase [Source:VGNC Symbol;Acc:VGNC:30944] |
| 7 | 96,536,559 | 96,592,167 | LIX1 | protein_coding | ENSBTAG00000009772.6 | limb and CNS expressed 1 [Source:VGNC Symbol;Acc:VGNC:30912] |
| 7 | 106,497,219 | 106,784,981 | EFNA5 | protein_coding | ENSBTAG00000016515.5 | ephrin A5 [Source:VGNC Symbol;Acc:VGNC:28358] |
| 7 | 106,989,100 | 107,509,243 | FBXL17 | protein_coding | ENSBTAG00000054662.1 | F-box and leucine rich repeat protein 17 [Source:VGNC Symbol;Acc:VGNC:106738] |
| 7 | 107,827,212 | 108,280,766 | FER | protein_coding | ENSBTAG00000003051.7 | FER tyrosine kinase [Source:VGNC Symbol;Acc:VGNC:28948] |
| 9 | 44,880,467 | 44,930,046 | BVES | protein_coding | ENSBTAG00000018790.5 | blood vessel epicardial substance [Source:VGNC Symbol;Acc:VGNC:26610] |
| 9 | 44,948,437 | 45,069,126 | LIN28B | protein_coding | ENSBTAG00000043973.3 | lin-28 homolog B [Source:VGNC Symbol;Acc:VGNC:108131] |
| 9 | 47,889,245 | 48,618,507 | GRIK2 | protein_coding | ENSBTAG00000033153.4 | glutamate ionotropic receptor kainate type subunit 2 [Source:VGNC Symbol;Acc:VGNC:29643] |
| 9 | 49,096,929 | 49,429,819 | ASCC3 | protein_coding | ENSBTAG00000020482.6 | activating signal cointegrator 1 complex subunit 3 [Source:VGNC Symbol;Acc:VGNC:26205] |
| 9 | 55,089,680 | 55,089,993 | 7SK | misc_RNA | ENSBTAG00000043941.2 | 7SK RNA [Source:RFAM;Acc:RF00100] |
| 10 | 85,254,086 | 85,275,390 | PTGR2 | protein_coding | ENSBTAG00000003747.6 | prostaglandin reductase 2 [Source:NCBI gene (formerly Entrezgene);Acc:506263] |
| 10 | 85,267,238 | 85,267,295 | bta-mir-7859 | miRNA | ENSBTAG00000049659.1 | bta-mir-7859 [Source:miRBase;Acc:MI0025529] |
| 10 | 85,277,621 | 85,320,048 | ZNF410 | protein_coding | ENSBTAG00000020327.5 | zinc finger protein 410 [Source:VGNC Symbol;Acc:VGNC:37271] |
| 10 | 85,321,516 | 85,333,750 | FAM161B | protein_coding | ENSBTAG00000020329.6 | FAM161 centrosomal protein B [Source:VGNC Symbol;Acc:VGNC:28743] |
| 10 | 85,333,909 | 85,348,765 | COQ6 | protein_coding | ENSBTAG00000020331.4 | coenzyme Q6, monooxygenase [Source:VGNC Symbol;Acc:VGNC:27614] |
| 10 | 85,351,607 | 85,377,557 | ENTPD5 | protein_coding | ENSBTAG00000020334.6 | ectonucleoside triphosphate diphosphohydrolase 5 (inactive) [Source:VGNC Symbol;Acc:VGNC:28511] |
| 10 | 85,384,726 | 85,426,894 | BBOF1 | protein_coding | ENSBTAG00000018467.5 | basal body orientation factor 1 [Source:VGNC Symbol;Acc:VGNC:26431] |
| 10 | 85,428,884 | 85,447,592 | ALDH6A1 | protein_coding | ENSBTAG00000018469.4 | aldehyde dehydrogenase 6 family member A1 [Source:VGNC Symbol;Acc:VGNC:25817] |
| 10 | 85,448,145 | 85,560,860 | LIN52 | protein_coding | ENSBTAG00000030557.3 | lin-52 DREAM MuvB core complex component [Source:VGNC Symbol;Acc:VGNC:30893] |
| 10 | 85,599,605 | 85,618,776 | VSX2 | protein_coding | ENSBTAG00000014632.5 | visual system homeobox 2 [Source:VGNC Symbol;Acc:VGNC:36846] |
| 10 | 85,631,547 | 85,647,111 | ABCD4 | protein_coding | ENSBTAG00000014633.6 | ATP binding cassette subfamily D member 4 [Source:VGNC Symbol;Acc:VGNC:55099] |
| 10 | 85,679,086 | 85,681,188 | VRTN | protein_coding | ENSBTAG00000005774.5 | vertebrae development associated [Source:VGNC Symbol;Acc:VGNC:36837] |
| 10 | 85,710,786 | 85,768,852 | SYNDIG1L | protein_coding | ENSBTAG00000052950.1 | synapse differentiation inducing 1 like [Source:VGNC Symbol;Acc:VGNC:35520] |
| 10 | 85,774,962 | 85,783,794 | NPC2 | protein_coding | ENSBTAG00000021955.3 | NPC intracellular cholesterol transporter 2 [Source:VGNC Symbol;Acc:VGNC:32196] |
| 10 | 85,776,496 | 85,776,681 | U2 | snRNA | ENSBTAG00000043732.3 | U2 spliceosomal RNA [Source:RFAM;Acc:RF00004] |
| 10 | 85,783,972 | 85,785,721 | ISCA2 | protein_coding | ENSBTAG00000021956.5 | iron-sulfur cluster assembly 2 [Source:VGNC Symbol;Acc:VGNC:30291] |
| 10 | 85,791,017 | 85,898,441 | LTBP2 | protein_coding | ENSBTAG00000021957.4 | latent transforming growth factor beta binding protein 2 [Source:VGNC Symbol;Acc:VGNC:31072] |
| 10 | 85,945,768 | 85,988,421 | AREL1 | protein_coding | ENSBTAG00000020379.5 | apoptosis resistant E3 ubiquitin protein ligase 1 [Source:VGNC Symbol;Acc:VGNC:26058] |
| 10 | 85,987,324 | 85,998,914 | FCF1 | protein_coding | ENSBTAG00000020381.4 | FCF1 rRNA-processing protein [Source:VGNC Symbol;Acc:VGNC:28933] |
| 10 | 86,019,850 | 86,083,721 | YLPM1 | protein_coding | ENSBTAG00000008287.6 | YLP motif containing 1 [Source:VGNC Symbol;Acc:VGNC:37031] |
| 10 | 86,108,354 | 86,116,785 | PROX2 | protein_coding | ENSBTAG00000015052.6 | prospero homeobox 2 [Source:VGNC Symbol;Acc:VGNC:33370] |
| 10 | 86,713,963 | 86,736,092 | BATF | protein_coding | ENSBTAG00000025405.4 | basic leucine zipper ATF-like transcription factor [Source:VGNC Symbol;Acc:VGNC:26425] |
| 10 | 86,764,090 | 86,898,356 | FLVCR2 | protein_coding | ENSBTAG00000040078.3 | feline leukemia virus subgroup C cellular receptor family, member 2 [Source:NCBI gene (formerly Entrezgene);Acc:507318] |
| 10 | 86,908,115 | 86,916,703 | ERG28 | protein_coding | ENSBTAG00000011987.6 | ergosterol biosynthesis 28 homolog [Source:VGNC Symbol;Acc:VGNC:55113] |
| 10 | 86,918,978 | 87,207,088 | TTLL5 | protein_coding | ENSBTAG00000025403.5 | tubulin tyrosine ligase like 5 [Source:VGNC Symbol;Acc:VGNC:36493] |
| 10 | 87,237,844 | 87,271,480 | TGFB3 | protein_coding | ENSBTAG00000012004.5 | transforming growth factor beta 3 [Source:VGNC Symbol;Acc:VGNC:35803] |
| 10 | 87,263,348 | 87,371,765 | IFT43 | protein_coding | ENSBTAG00000012005.4 | intraflagellar transport 43 [Source:VGNC Symbol;Acc:VGNC:30068] |
| 12 | 30,327,227 | 30,333,011 | HMGB1 | protein_coding | ENSBTAG00000018103.4 | high mobility group box 1 [Source:VGNC Symbol;Acc:VGNC:53816] |
| 12 | 30,478,919 | 30,533,274 | KATNAL1 | protein_coding | ENSBTAG00000009340.6 | katanin catalytic subunit A1 like 1 [Source:VGNC Symbol;Acc:VGNC:30406] |
| 12 | 31,001,535 | 31,388,275 | MTUS2 | protein_coding | ENSBTAG00000001094.6 | microtubule associated scaffold protein 2 [Source:VGNC Symbol;Acc:VGNC:31757] |
| 12 | 31,471,716 | 31,482,858 | POMP | protein_coding | ENSBTAG00000014024.4 | proteasome maturation protein [Source:VGNC Symbol;Acc:VGNC:33157] |
| 12 | 31,624,125 | 31,829,369 | FLT1 | protein_coding | ENSBTAG00000016915.7 | fms related receptor tyrosine kinase 1 [Source:VGNC Symbol;Acc:VGNC:29041] |
| 12 | 31,797,031 | 31,797,108 | bta-mir-2300a | miRNA | ENSBTAG00000044883.2 | bta-mir-2300a [Source:miRBase;Acc:MI0011310] |
| 12 | 31,834,570 | 31,957,265 | PAN3 | protein_coding | ENSBTAG00000009912.6 | poly(A) specific ribonuclease subunit PAN3 [Source:VGNC Symbol;Acc:VGNC:53830] |
| 12 | 32,872,542 | 32,875,852 | GPR12 | protein_coding | ENSBTAG00000010769.6 | G protein-coupled receptor 12 [Source:VGNC Symbol;Acc:VGNC:29547] |
| 12 | 32,913,740 | 32,961,755 | WASF3 | protein_coding | ENSBTAG00000014619.5 | WASP family member 3 [Source:VGNC Symbol;Acc:VGNC:36867] |
| 12 | 33,082,022 | 33,153,677 | CDK8 | protein_coding | ENSBTAG00000016737.5 | cyclin dependent kinase 8 [Source:VGNC Symbol;Acc:VGNC:27135] |
| 12 | 33,169,752 | 33,179,038 | RNF6 | protein_coding | ENSBTAG00000005318.6 | ring finger protein 6 [Source:VGNC Symbol;Acc:VGNC:34071] |
| 17 | 34,353,125 | 34,357,245 | SPRY1 | protein_coding | ENSBTAG00000021245.5 | sprouty RTK signaling antagonist 1 [Source:VGNC Symbol;Acc:VGNC:35241] |
| 17 | 34,358,675 | 34,358,736 | bta-mir-12036 | miRNA | ENSBTAG00000052988.1 | bta-mir-12036 [Source:miRBase;Acc:MI0038457] |
| 17 | 34,422,664 | 34,747,076 | SPATA5 | protein_coding | ENSBTAG00000000125.6 | spermatosis associated 5 [Source:VGNC Symbol;Acc:VGNC:35187] |
| 17 | 64,971,207 | 64,996,531 | PIWIL3 | protein_coding | ENSBTAG00000015526.6 | piwi like RNA-mediated silencing 3 [Source:VGNC Symbol;Acc:VGNC:32929] |
| 17 | 65,010,788 | 65,083,923 | SGSM1 | protein_coding | ENSBTAG00000006592.6 | small G protein signaling modulator 1 [Source:VGNC Symbol;Acc:VGNC:34550] |
| 17 | 65,089,357 | 65,098,969 | LHFPL7 | protein_coding | ENSBTAG00000006594.4 | LHFPL tetraspan subfamily member 7 [Source:VGNC Symbol;Acc:VGNC:36033] |
| 17 | 65,153,148 | 65,283,727 | KIAA1671 | protein_coding | ENSBTAG00000044022.3 | KIAA1671 [Source:VGNC Symbol;Acc:VGNC:30575] |
| 17 | 65,290,954 | 65,297,599 | CRYBB3 | protein_coding | ENSBTAG00000001255.4 | crystallin beta B3 [Source:VGNC Symbol;Acc:VGNC:27738] |
| 17 | 65,295,023 | 65,318,605 | CRYBB2 | protein_coding | ENSBTAG00000004088.3 | crystallin beta B2 [Source:VGNC Symbol;Acc:VGNC:27737] |
| 17 | 65,389,743 | 65,505,336 | GRK3 | protein_coding | ENSBTAG00000000005.6 | G protein-coupled receptor kinase 3 [Source:VGNC Symbol;Acc:VGNC:53904] |
| 17 | 65,530,211 | 65,744,827 | MYO18B | protein_coding | ENSBTAG00000000698.6 | myosin XVIIIB [Source:VGNC Symbol;Acc:VGNC:31814] |
| 17 | 65,558,887 | 65,558,965 | bta-mir-10020 | miRNA | ENSBTAG00000053799.1 | bta-mir-10020 [Source:miRBase;Acc:MI0032666] |
| 17 | 65,872,051 | 66,063,271 | SEZ6L | protein_coding | ENSBTAG00000006569.6 | seizure related 6 homolog like [Source:VGNC Symbol;Acc:VGNC:34503] |
| 17 | 66,098,793 | 66,107,865 | ASPHD2 | protein_coding | ENSBTAG00000037832.3 | aspartate beta-hydroxylase domain containing 2 [Source:VGNC Symbol;Acc:VGNC:26221] |
| 17 | 66,113,220 | 66,140,520 | HPS4 | protein_coding | ENSBTAG00000017038.6 | HPS4 biosis of lysosomal organelles complex 3 subunit 2 [Source:VGNC Symbol;Acc:VGNC:29945] |
| 17 | 67,173,546 | 67,221,633 | MN1 | protein_coding | ENSBTAG00000010464.6 | MN1 proto-onco, transcriptional regulator [Source:VGNC Symbol;Acc:VGNC:31536] |
| 17 | 67,268,876 | 67,330,126 | PITPNB | protein_coding | ENSBTAG00000017799.6 | phosphatidylinositol transfer protein beta [Source:VGNC Symbol;Acc:VGNC:32920] |
| 17 | 67,395,806 | 67,801,228 | TTC28 | protein_coding | ENSBTAG00000012193.6 | tetratricopeptide repeat domain 28 [Source:VGNC Symbol;Acc:VGNC:36467] |
| 19 | 5,115,956 | 5,315,869 | STXBP4 | protein_coding | ENSBTAG00000016078.6 | syntaxin binding protein 4 [Source:VGNC Symbol;Acc:VGNC:106959] |
| 19 | 15,782,652 | 15,786,668 | CCL1 | protein_coding | ENSBTAG00000008832.6 | C-C motif chemokine ligand 1 [Source:VGNC Symbol;Acc:VGNC:26943] |
| 19 | 15,807,710 | 15,809,743 | CCL8 | protein_coding | ENSBTAG00000014113.4 | chemokine (C-C motif) ligand 8 [Source:NCBI gene (formerly Entrezgene);Acc:281044] |
| 19 | 18,989,423 | 19,004,078 | WSB1 | protein_coding | ENSBTAG00000005008.5 | WD repeat and SOCS box containing 1 [Source:VGNC Symbol;Acc:VGNC:36971] |
| 19 | 19,088,871 | 19,247,444 | KSR1 | protein_coding | ENSBTAG00000006601.6 | kinase suppressor of ras 1 [Source:VGNC Symbol;Acc:VGNC:30754] |
| 19 | 19,368,210 | 19,387,215 | LGALS9 | protein_coding | ENSBTAG00000006846.5 | galectin 9 [Source:NCBI gene (formerly Entrezgene);Acc:510813] |
| 19 | 19,388,579 | 19,429,569 | NOS2 | protein_coding | ENSBTAG00000006894.6 | nitric oxide synthase 2 [Source:VGNC Symbol;Acc:VGNC:32173] |
| 19 | 19,487,860 | 19,503,235 | LYRM9 | protein_coding | ENSBTAG00000033298.4 | LYR motif containing 9 [Source:NCBI gene (formerly Entrezgene);Acc:615250] |
| 19 | 19,806,850 | 19,814,864 | TMEM97 | protein_coding | ENSBTAG00000008109.3 | transmembrane protein 97 [Source:VGNC Symbol;Acc:VGNC:36129] |
| 19 | 19,814,952 | 19,821,909 | IFT20 | protein_coding | ENSBTAG00000008110.4 | intraflagellar transport 20 [Source:VGNC Symbol;Acc:VGNC:50160] |
| 19 | 19,822,153 | 19,830,834 | TNFAIP1 | protein_coding | ENSBTAG00000008115.6 | TNF alpha induced protein 1 [Source:VGNC Symbol;Acc:VGNC:36154] |
| 19 | 19,826,425 | 19,841,200 | POLDIP2 | protein_coding | ENSBTAG00000008116.6 | DNA polymerase delta interacting protein 2 [Source:VGNC Symbol;Acc:VGNC:33116] |
| 19 | 19,841,240 | 19,846,107 | TMEM199 | protein_coding | ENSBTAG00000008120.5 | transmembrane protein 199 [Source:VGNC Symbol;Acc:VGNC:36020] |
| 19 | 19,852,996 | 19,854,571 | SEBOX | protein_coding | ENSBTAG00000033270.4 | SEBOX homeobox [Source:VGNC Symbol;Acc:VGNC:34401] |
| 19 | 19,855,894 | 19,858,909 | VTN | protein_coding | ENSBTAG00000016151.4 | vitronectin [Source:VGNC Symbol;Acc:VGNC:55883] |
| 19 | 19,861,214 | 19,881,877 | SARM1 | protein_coding | ENSBTAG00000002816.6 | sterile alpha and TIR motif containing 1 [Source:VGNC Symbol;Acc:VGNC:34290] |
| 19 | 19,884,289 | 19,891,906 | SLC46A1 | protein_coding | ENSBTAG00000002817.6 | solute carrier family 46 member 1 [Source:VGNC Symbol;Acc:VGNC:34884] |
| 19 | 19,905,572 | 19,905,671 | SNORA70 | snoRNA | ENSBTAG00000044525.2 | Small nucleolar RNA SNORA70 [Source:RFAM;Acc:RF00156] |
| 19 | 19,943,529 | 19,967,651 | SLC13A2 | protein_coding | ENSBTAG00000008788.6 | solute carrier family 13 member 2 [Source:VGNC Symbol;Acc:VGNC:34674] |
| 19 | 19,977,270 | 20,005,151 | FOXN1 | protein_coding | ENSBTAG00000013095.4 | forkhead box N1 [Source:VGNC Symbol;Acc:VGNC:29095] |
| 19 | 20,882,415 | 21,124,185 | SSH2 | protein_coding | ENSBTAG00000011011.6 | slingshot protein phosphatase 2 [Source:VGNC Symbol;Acc:VGNC:35313] |
| 19 | 21,674,362 | 21,862,869 | ABR | protein_coding | ENSBTAG00000008424.6 | ABR activator of RhoGEF and GTPase [Source:VGNC Symbol;Acc:VGNC:25511] |
| 19 | 21,863,372 | 21,870,716 | TIMM22 | protein_coding | ENSBTAG00000008423.3 | translocase of inner mitochondrial membrane 22 [Source:VGNC Symbol;Acc:VGNC:49575] |
| 19 | 21,889,110 | 22,053,102 | NXN | protein_coding | ENSBTAG00000000855.6 | nucleoredoxin [Source:VGNC Symbol;Acc:VGNC:32376] |
| 19 | 53,954,985 | 53,977,477 | AFMID | protein_coding | ENSBTAG00000007125.6 | arylformamidase [Source:VGNC Symbol;Acc:VGNC:25716] |
| 19 | 53,977,498 | 53,991,174 | TK1 | protein_coding | ENSBTAG00000007121.6 | thymidine kinase 1 [Source:VGNC Symbol;Acc:VGNC:35884] |
| 19 | 53,992,203 | 53,995,976 | SYNGR2 | protein_coding | ENSBTAG00000019069.5 | synaptogyrin 2 [Source:VGNC Symbol;Acc:VGNC:107278] |
| 19 | 54,013,443 | 54,024,053 | TMC8 | protein_coding | ENSBTAG00000019067.5 | transmembrane channel like 8 [Source:VGNC Symbol;Acc:VGNC:35924] |
| 19 | 54,025,900 | 54,048,017 | TMC6 | protein_coding | ENSBTAG00000018661.6 | transmembrane channel like 6 [Source:VGNC Symbol;Acc:VGNC:35922] |
| 19 | 54,038,763 | 54,038,841 | bta-mir-2348 | miRNA | ENSBTAG00000044955.2 | bta-mir-2348 [Source:miRBase;Acc:MI0011376] |
| 19 | 54,051,772 | 54,124,386 | TNRC6C | protein_coding | ENSBTAG00000018658.7 | trinucleotide repeat containing adaptor 6C [Source:VGNC Symbol;Acc:VGNC:36206] |
| 19 | 55,984,816 | 56,020,314 | LLGL2 | protein_coding | ENSBTAG00000020067.6 | LLGL scribble cell polarity complex component 2 [Source:VGNC Symbol;Acc:VGNC:30916] |
| 19 | 56,020,690 | 56,027,838 | TSEN54 | protein_coding | ENSBTAG00000000824.5 | tRNA splicing endonuclease subunit 54 [Source:VGNC Symbol;Acc:VGNC:36412] |
| 19 | 56,028,993 | 56,043,211 | CASKIN2 | protein_coding | ENSBTAG00000007220.6 | CASK interacting protein 2 [Source:VGNC Symbol;Acc:VGNC:26778] |
| 19 | 56,044,033 | 56,058,416 | TMEM94 | protein_coding | ENSBTAG00000007217.6 | transmembrane protein 94 [Source:VGNC Symbol;Acc:VGNC:36128] |
| 19 | 56,117,146 | 56,181,857 | GRB2 | protein_coding | ENSBTAG00000004736.5 | growth factor receptor bound protein 2 [Source:VGNC Symbol;Acc:VGNC:29631] |
| 22 | 45,924,535 | 46,818,511 | CACNA2D3 | protein_coding | ENSBTAG00000013117.7 | calcium voltage-gated channel auxiliary subunit alpha2delta 3 [Source:VGNC Symbol;Acc:VGNC:26681] |
| 22 | 46,051,998 | 46,062,657 | LRTM1 | protein_coding | ENSBTAG00000013124.6 | leucine rich repeats and transmembrane domains 1 [Source:VGNC Symbol;Acc:VGNC:31046] |
| 22 | 47,024,359 | 47,031,572 | SELENOK | protein_coding | ENSBTAG00000032374.5 | selenoprotein K [Source:NCBI gene (formerly Entrezgene);Acc:615114] |
| 22 | 47,032,971 | 47,056,658 | ACTR8 | protein_coding | ENSBTAG00000011180.4 | actin related protein 8 [Source:VGNC Symbol;Acc:VGNC:25589] |
| 22 | 47,051,292 | 47,066,805 | IL17RB | protein_coding | ENSBTAG00000011178.6 | interleukin 17 receptor B [Source:HGNC Symbol;Acc:HGNC:18015] |
| 22 | 47,133,914 | 47,158,681 | CHDH | protein_coding | ENSBTAG00000010027.5 | choline dehydrogenase [Source:VGNC Symbol;Acc:VGNC:27285] |
| 22 | 47,162,857 | 47,508,559 | CACNA1D | protein_coding | ENSBTAG00000010026.6 | calcium voltage-gated channel subunit alpha1 D [Source:VGNC Symbol;Acc:VGNC:26674] |
| 22 | 47,646,509 | 47,690,569 | DCP1A | protein_coding | ENSBTAG00000010863.5 | decapping mRNA 1A [Source:VGNC Symbol;Acc:VGNC:27919] |
| 22 | 47,707,405 | 47,731,710 | TKT | protein_coding | ENSBTAG00000003758.5 | transketolase [Source:VGNC Symbol;Acc:VGNC:58616] |
| 22 | 47,749,990 | 47,780,548 | PRKCD | protein_coding | ENSBTAG00000008719.6 | protein kinase C delta [Source:VGNC Symbol;Acc:VGNC:33329] |
| 22 | 47,812,822 | 47,852,306 | RFT1 | protein_coding | ENSBTAG00000020973.7 | RFT1 homolog [Source:NCBI gene (formerly Entrezgene);Acc:504597] |
| 22 | 47,877,481 | 47,992,605 | SFMBT1 | protein_coding | ENSBTAG00000011345.7 | Scm like with four mbt domains 1 [Source:VGNC Symbol;Acc:VGNC:34515] |
| 22 | 47,999,883 | 48,051,428 | TMEM110 | protein_coding | ENSBTAG00000011344.5 | transmembrane protein 110 [Source:NCBI gene (formerly Entrezgene);Acc:538654] |
| 22 | 48,056,089 | 48,057,235 | MUSTN1 | protein_coding | ENSBTAG00000032531.2 | musculoskeletal, embryonic nuclear protein 1 [Source:NCBI gene (formerly Entrezgene);Acc:616088] |
| 22 | 48,059,508 | 48,079,899 | ITIH4 | protein_coding | ENSBTAG00000007850.5 | inter-alpha-trypsin inhibitor heavy chain 4 [Source:VGNC Symbol;Acc:VGNC:30336] |
| 22 | 48,078,023 | 48,091,969 | ITIH3 | protein_coding | ENSBTAG00000007846.6 | inter-alpha-trypsin inhibitor heavy chain 3 [Source:VGNC Symbol;Acc:VGNC:30335] |
| 22 | 48,099,219 | 48,113,488 | ITIH1 | protein_coding | ENSBTAG00000007843.5 | inter-alpha-trypsin inhibitor heavy chain 1 [Source:VGNC Symbol;Acc:VGNC:30334] |
| 22 | 48,124,642 | 48,152,515 | NEK4 | protein_coding | ENSBTAG00000004749.5 | NIMA related kinase 4 [Source:VGNC Symbol;Acc:VGNC:31995] |
| 22 | 48,176,132 | 48,283,003 | PBRM1 | protein_coding | ENSBTAG00000014786.6 | polybromo 1 [Source:VGNC Symbol;Acc:VGNC:32606] |
| 22 | 48,286,485 | 48,289,693 | SMIM4 | protein_coding | ENSBTAG00000044079.3 | small integral membrane protein 4 [Source:VGNC Symbol;Acc:VGNC:35022] |
| 22 | 48,291,599 | 48,343,007 | NT5DC2 | protein_coding | ENSBTAG00000014784.6 | 5'-nucleotidase domain containing 2 [Source:VGNC Symbol;Acc:VGNC:32295] |
| 22 | 48,343,002 | 48,368,328 | STAB1 | protein_coding | ENSBTAG00000014782.6 | stabilin 1 [Source:VGNC Symbol;Acc:VGNC:35348] |
| 22 | 48,370,379 | 48,432,781 | NISCH | protein_coding | ENSBTAG00000014779.5 | nischarin [Source:VGNC Symbol;Acc:VGNC:32088] |
| 22 | 48,434,632 | 48,437,765 | TNNC1 | protein_coding | ENSBTAG00000045757.2 | troponin C1, slow skeletal and cardiac type [Source:VGNC Symbol;Acc:VGNC:36189] |

^1^Positions based on ARS-UCD1.2 Bos taurus genome assembly

^2^Ensembl database Gene stable ID version accession

^3^Ensembl database Gene description

Table S9 - Enrichment functional analysis for genes found: Biological Process

| ^1^GO ID | ^2^GO Term | Term PValue | Bonferroni Correction | Associated Genes Found |
| --- | --- | --- | --- | --- |
| GO:0072359 | circulatory system development | 0.00 | 0.02 | [ATG5, BVES, CDH13, FLT1, FOXJ1, FOXN1, JMJD6, LGALS12, MYDGF, POPDC3, SOCS3, TAL1] |
| GO:0007507 | heart development | 0.10 | 0.10 | [ATG5, BVES, FOXJ1, JMJD6, POPDC3] |
| GO:0046649 | lymphocyte activation | 0.00 | 0.00 | [ATG5, BATF, CLEC7A, FGR, FOXJ1, FOXN1, JMJD6, MYB, SAMSN1, SELENOK, SLC7A1, SUPT6H, TICAM1, UNC13D, ZNF683] |
| GO:0002520 | immune system development | 0.00 | 0.02 | [ATG5, BATF, BVES, FLT1, FOXJ1, FOXN1, JMJD6, MYB, OSTM1, SUPT6H, TAL1, ZNF683] |
| GO:0042110 | T cell activation | 0.00 | 0.01 | [ATG5, BATF, FOXJ1, FOXN1, JMJD6, MYB, SELENOK, SLC7A1, ZNF683] |
| GO:0030097 | hemopoiesis | 0.00 | 0.02 | [ATG5, BATF, BVES, FLT1, FOXJ1, FOXN1, JMJD6, MYB, OSTM1, TAL1, ZNF683] |
| GO:0002521 | leukocyte differentiation | 0.00 | 0.02 | [ATG5, BATF, FOXJ1, FOXN1, JMJD6, MYB, OSTM1, TAL1, ZNF683] |
| GO:0050863 | regulation of T cell activation | 0.00 | 0.02 | [FOXJ1, FOXN1, MYB, SELENOK, SLC7A1, ZNF683] |
| GO:0030217 | T cell differentiation | 0.00 | 0.00 | [ATG5, BATF, FOXJ1, FOXN1, JMJD6, MYB, ZNF683] |
| GO:0002682 | regulation of immune system process | 0.00 | 0.00 | [ATG5, CLEC12A, CLEC12B, CLEC7A, FGR, FOXJ1, FOXN1, MYB, SAMSN1, SELENOK, SLC7A1, SUPT6H, TAL1, TICAM1, UNC13D, ZNF683] |
| GO:0002250 | adaptive immune response | 0.01 | 0.02 | [BATF, CLEC7A, FOXJ1, SAMSN1, SUPT6H, UNC13D] |
| GO:0002443 | leukocyte mediated immunity | 0.00 | 0.00 | [BATF, CLEC12B, CLEC7A, FGR, FOXJ1, SUPT6H, TICAM1, UNC13D] |
| GO:0002366 | leukocyte activation involved in immune response | 0.00 | 0.01 | [BATF, CLEC7A, FGR, MYB, SUPT6H, UNC13D, ZNF683] |
| GO:0002697 | regulation of immune effector process | 0.00 | 0.00 | [ATG5, CLEC12B, CLEC7A, FGR, FOXJ1, MYB, SUPT6H, TICAM1, UNC13D, ZNF683] |
| GO:0046649 | lymphocyte activation | 0.00 | 0.00 | [ATG5, BATF, CLEC7A, FGR, FOXJ1, FOXN1, JMJD6, MYB, SAMSN1, SELENOK, SLC7A1, SUPT6H, TICAM1, UNC13D, ZNF683] |
| GO:0050776 | regulation of immune response | 0.00 | 0.02 | [CLEC12A, CLEC12B, CLEC7A, FGR, FOXJ1, MYB, SAMSN1, SELENOK, SUPT6H, UNC13D] |
| GO:0002449 | lymphocyte mediated immunity | 0.01 | 0.02 | [BATF, CLEC12B, FOXJ1, SUPT6H, UNC13D] |
| GO:0002694 | regulation of leukocyte activation | 0.00 | 0.00 | [CLEC7A, FGR, FOXJ1, FOXN1, MYB, SAMSN1, SELENOK, SLC7A1, SUPT6H, TICAM1, UNC13D, ZNF683] |
| GO:0002285 | lymphocyte activation involved in immune response | 0.00 | 0.02 | [BATF, MYB, SUPT6H, UNC13D, ZNF683] |
| GO:0002703 | regulation of leukocyte mediated immunity | 0.00 | 0.00 | [CLEC12B, CLEC7A, FGR, FOXJ1, SUPT6H, TICAM1, UNC13D] |
| GO:0042110 | T cell activation | 0.00 | 0.01 | [ATG5, BATF, FOXJ1, FOXN1, JMJD6, MYB, SELENOK, SLC7A1, ZNF683] |
| GO:0050863 | regulation of T cell activation | 0.00 | 0.02 | [FOXJ1, FOXN1, MYB, SELENOK, SLC7A1, ZNF683] |
| GO:0030217 | T cell differentiation | 0.00 | 0.00 | [ATG5, BATF, FOXJ1, FOXN1, JMJD6, MYB, ZNF683] |

^1^Gene ontology term ID

^2^Gene ontology Term

Table S10 - Enrichment functional analysis for genes found: Immune System

| ^1^GO ID | ^2^GO Term | Term PValue | Bonferroni Correction | Associated Genes Found |
| --- | --- | --- | --- | --- |
| GO:0002366 | leukocyte activation involved in immune response | 0.01 | 0.08 | [BATF, CLEC7A, FGR, MYB, SUPT6H, UNC13D, ZNF683] |
| GO:0002285 | lymphocyte activation involved in immune response | 0.04 | 0.17 | [BATF, MYB, SUPT6H, UNC13D, ZNF683] |
| GO:0030097 | hemopoiesis | 0.28 | 0.28 | [ATG5, BATF, BVES, FLT1, FOXJ1, FOXN1, JMJD6, MYB, OSTM1, TAL1, ZNF683] |
| GO:0042110 | T cell activation | 0.04 | 0.21 | [ATG5, BATF, FOXJ1, FOXN1, JMJD6, MYB, SELENOK, SLC7A1, ZNF683] |
| GO:0051249 | regulation of lymphocyte activation | 0.00 | 0.01 | [CLEC7A, FGR, FOXJ1, FOXN1, MYB, SAMSN1, SELENOK, SLC7A1, SUPT6H, TICAM1, ZNF683] |
| GO:0002521 | leukocyte differentiation | 0.09 | 0.26 | [ATG5, BATF, FOXJ1, FOXN1, JMJD6, MYB, OSTM1, TAL1, ZNF683] |
| GO:0050863 | regulation of T cell activation | 0.11 | 0.22 | [FOXJ1, FOXN1, MYB, SELENOK, SLC7A1, ZNF683] |
| GO:0030217 | T cell differentiation | 0.01 | 0.08 | [ATG5, BATF, FOXJ1, FOXN1, JMJD6, MYB, ZNF683] |

^1^Gene ontology term ID

^2^Gene ontology Term

Table S11 - Enrichment functional analysis for genes found: Molecular Function

| ^1^GO ID | ^2^GO Term | Term PValue | Bonferroni Correction | Associated Genes Found |
| --- | --- | --- | --- | --- |
| GO:0046649 | lymphocyte activation | 0.00 | 0.00 | [ATG5, BATF, CLEC7A, FGR, FOXJ1, FOXN1, JMJD6, MYB, SAMSN1, SELENOK, SLC7A1, SUPT6H, TICAM1, UNC13D, ZNF683] |
| GO:0005488 | binding | 0.24 | 1.00 | [A2M, ABR, ACOX1, ACTR8, ARRDC5, ASCC3, ATP2C2, BATF, BEND5, BVES, CDH1, CDH13, CDH3, CHDH, CLEC12A, CLEC12B, CLEC1A, CLEC7A, COBLL1, COQ6, CRYBG2, CYGB, DPP9, EVPL, FGD3, FGR, FLT1, FLVCR2, FOXJ1, FOXN1, GALK1, GPR12, GRB14, HSPA13, JDP2, JMJD6, LGALS12, LNX2, LRTM1, M6PR, MTIF3, MYB, NLK, NSRP1, OLR1, POPDC3, PRPSAP1, RNF157, SAMSN1, SCML4, SEC63, SELENOK, SLC30A2, SOCS3, SPATA6, SRP68, SRSF2, STIL, STYK1, SUPT6H, TAL1, TICAM1, TK1, TM2D1, TRABD2B, TRIM47, TRIM63, TRIM65, UBXN11, UNC13D, VSX2, WBP2, YBX3, ZNF410, ZNF683] |
| GO:0005515 | protein binding | 0.60 | 1.00 | [A2M, ACOX1, ARRDC5, CDH1, CDH13, CDH3, CLEC12A, CLEC12B, COBLL1, DPP9, FGR, FLT1, GRB14, HSPA13, JMJD6, LGALS12, LNX2, LRTM1, M6PR, NLK, OLR1, PRPSAP1, SAMSN1, SCML4, SELENOK, SLC30A2, SOCS3, SPATA6, SRP68, STIL, STYK1, SUPT6H, TAL1, TICAM1, TK1, TRABD2B, TRIM63, UBXN11, UNC13D] |
| GO:0003824 | catalytic activity | 0.18 | 1.00 | [A2M, ABR, ACOX1, ASCC3, ASPHD2, ATG10, BVES, CDH3, CHDH, CLEC7A, COQ6, DPP9, FGD3, FGR, FLT1, FOXJ1, GALK1, IFI6, JMJD6, NLK, PAFAH2, PREP, PRPSAP1, RHBDF2, RNF157, SOCS3, ST6GALNAC1, ST6GALNAC2, STYK1, TK1, TRABD2B, TRIM47, TRIM63, UBE2O, WFDC1] |
| GO:0016740 | transferase activity | 0.89 | 1.00 | [ABR, ATG10, FGR, FLT1, GALK1, NLK, PAFAH2, PRPSAP1, RNF157, SOCS3, ST6GALNAC1, ST6GALNAC2, STYK1, TK1, TRIM47, TRIM63, UBE2O] |
| GO:0140096 | catalytic activity, acting on a protein | 0.90 | 1.00 | [A2M, ABR, ATG10, CLEC7A, DPP9, FGR, FLT1, IFI6, JMJD6, NLK, PREP, RHBDF2, RNF157, STYK1, TRABD2B, TRIM47, TRIM63, UBE2O, WFDC1] |
| GO:0036094 | small molecule binding | 0.48 | 1.00 | [ABR, ACOX1, ACTR8, ASCC3, ATP2C2, BVES, CHDH, COQ6, CYGB, FGD3, FGR, FLT1, GALK1, HSPA13, NLK, POPDC3, STYK1, TK1] |
| GO:0043167 | ion binding | 0.91 | 0.91 | [ABR, ACOX1, ACTR8, ASCC3, ATP2C2, BVES, CDH1, CDH13, CDH3, CHDH, CLEC7A, COQ6, CYGB, FGD3, FGR, FLT1, GALK1, GPR12, HSPA13, JMJD6, LNX2, NLK, POPDC3, PRPSAP1, RNF157, STYK1, TK1, TRABD2B, TRIM47, TRIM63, TRIM65, ZNF410] |
| GO:0097159 | organic cyclic compound binding | 0.23 | 1.00 | [ABR, ACOX1, ACTR8, ASCC3, ATP2C2, BATF, BEND5, BVES, CHDH, COQ6, CYGB, FGD3, FGR, FLT1, FLVCR2, FOXJ1, FOXN1, GALK1, HSPA13, JDP2, JMJD6, MTIF3, MYB, NLK, NSRP1, POPDC3, SEC63, SRP68, SRSF2, STYK1, SUPT6H, TAL1, TK1, VSX2, WBP2, YBX3, ZNF410, ZNF683] |
| GO:1901363 | heterocyclic compound binding | 0.19 | 1.00 | [ABR, ACOX1, ACTR8, ASCC3, ATP2C2, BATF, BEND5, BVES, CHDH, COQ6, CYGB, FGD3, FGR, FLT1, FLVCR2, FOXJ1, FOXN1, GALK1, HSPA13, JDP2, JMJD6, MTIF3, MYB, NLK, NSRP1, POPDC3, SEC63, SRP68, SRSF2, STYK1, SUPT6H, TAL1, TK1, VSX2, WBP2, YBX3, ZNF410, ZNF683] |
| GO:0003676 | nucleic acid binding | 0.70 | 1.00 | [ASCC3, BATF, BEND5, FOXJ1, FOXN1, JDP2, JMJD6, MTIF3, MYB, NSRP1, SEC63, SRP68, SRSF2, SUPT6H, TAL1, VSX2, WBP2, YBX3, ZNF410, ZNF683] |
| GO:0043168 | anion binding | 0.48 | 1.00 | [ABR, ACOX1, ACTR8, ASCC3, ATP2C2, BVES, CHDH, COQ6, FGD3, FGR, FLT1, GALK1, HSPA13, NLK, POPDC3, STYK1, TK1] |
| GO:0043169 | cation binding | 0.90 | 1.00 | [ATP2C2, CDH1, CDH13, CDH3, CLEC7A, CYGB, FGD3, GPR12, JMJD6, LNX2, NLK, PRPSAP1, RNF157, TK1, TRABD2B, TRIM47, TRIM63, TRIM65, ZNF410] |
| GO:0003677 | DNA binding | 0.88 | 1.00 | [BATF, BEND5, FOXJ1, FOXN1, JDP2, MYB, SUPT6H, TAL1, VSX2, WBP2, YBX3, ZNF410, ZNF683] |

^1^Gene ontology term ID

^2^Gene ontology Term

Table S12 - Enrichment functional analysis for genes found: KEGG pathways

| ^1^GO ID | ^2^GO Term | P-value | Bonferroni Correction | Associated Genes Found |
| --- | --- | --- | --- | --- |
| KEGG:04614 | Renin-angiotensin system | 0.10 | 1.00 | [PREP] |
| KEGG:04742 | Taste transduction | 0.04 | 1.00 | [TAS2R7, TAS2R8] |
| KEGG:04810 | Regulation of actin cytoskeleton | 0.59 | 1.00 | [FGD3] |
| KEGG:00512 | Mucin type O-glycan biosynthesis | 0.12 | 1.00 | [ST6GALNAC1] |
| KEGG:05144 | Malaria | 0.22 | 1.00 | [KLRB1] |
| KEGG:05206 | MicroRNAs in cancer | 1.00 | 1.00 | [SLC7A1] |
| KEGG:00565 | Ether lipid metabolism | 0.19 | 1.00 | [PAFAH2] |
| KEGG:00130 | Ubiquinone and other terpenoid-quinone biosynthesis | 0.04 | 1.00 | [COQ6] |
| KEGG:04062 | Chemokine signaling pathway | 0.54 | 1.00 | [FGR] |
| KEGG:04218 | Cellular senescence | 0.50 | 1.00 | [LIN52] |
| KEGG:00260 | Glycine, serine and threonine metabolism | 0.16 | 1.00 | [CHDH] |
| KEGG:04530 | Tight junction | 0.17 | 1.00 | [BVES, YBX3] |
| KEGG:04610 | Complement and coagulation cascades | 0.32 | 1.00 | [A2M] |
| KEGG:00240 | Pyrimidine metabolism | 0.21 | 1.00 | [TK1] |
| KEGG:00983 | Drug metabolism | 0.27 | 1.00 | [TK1] |
| KEGG:00052 | Galactose metabolism | 0.12 | 1.00 | [GALK1] |
| KEGG:00520 | Amino sugar and nucleotide sugar metabolism | 0.19 | 1.00 | [GALK1] |
| KEGG:03060 | Protein export | 0.00 | 0.30 | [SEC63, SRP68] |
| KEGG:04141 | Protein processing in endoplasmic reticulum | 0.50 | 1.00 | [SEC63] |
| KEGG:03320 | PPAR signaling pathway | 0.04 | 1.00 | [ACOX1, OLR1] |
| KEGG:04142 | Lysosome | 0.40 | 1.00 | [M6PR] |
| KEGG:04145 | Phagosome | 0.03 | 1.00 | [CLEC7A, M6PR, OLR1] |
| KEGG:04625 | C-type lectin receptor signaling pathway | 0.36 | 1.00 | [CLEC7A] |
| KEGG:04910 | Insulin signaling pathway | 0.11 | 1.00 | [EXOC7, SOCS3] |
| KEGG:05132 | Salmonella infection | 0.24 | 1.00 | [EXOC7, M6PR] |
| KEGG:05152 | Tuberculosis | 0.56 | 1.00 | [CLEC7A] |
| KEGG:00071 | Fatty acid degradation | 0.16 | 1.00 | [ACOX1] |
| KEGG:00410 | beta-Alanine metabolism | 0.13 | 1.00 | [ACOX1] |
| KEGG:00592 | alpha-Linolenic acid metabolism | 0.11 | 1.00 | [ACOX1] |
| KEGG:00640 | Propanoate metabolism | 0.13 | 1.00 | [ACOX1] |
| KEGG:01040 | Biosynthesis of unsaturated fatty acids | 0.12 | 1.00 | [ACOX1] |
| KEGG:03320 | PPAR signaling pathway | 0.04 | 1.00 | [ACOX1, OLR1] |
| KEGG:04024 | cAMP signaling pathway | 0.62 | 1.00 | [ACOX1] |
| KEGG:04145 | Phagosome | 0.03 | 1.00 | [CLEC7A, M6PR, OLR1] |
| KEGG:04146 | Peroxisome | 0.29 | 1.00 | [ACOX1] |
| KEGG:04010 | MAPK signaling pathway | 0.34 | 1.00 | [FLT1, NLK] |
| KEGG:04014 | Ras signaling pathway | 1.00 | 1.00 | [FLT1] |
| KEGG:04015 | Rap1 signaling pathway | 0.22 | 1.00 | [CDH1, FLT1] |
| KEGG:04066 | HIF-1 signaling pathway | 0.37 | 1.00 | [FLT1] |
| KEGG:04068 | FoxO signaling pathway | 0.42 | 1.00 | [NLK] |
| KEGG:04151 | PI3K-Akt signaling pathway | 0.67 | 1.00 | [FLT1, MYB] |
| KEGG:04310 | Wnt signaling pathway | 0.49 | 1.00 | [NLK] |
| KEGG:04371 | Apelin signaling pathway | 0.44 | 1.00 | [CDH1] |
| KEGG:04390 | Hippo signaling pathway | 0.47 | 1.00 | [CDH1] |
| KEGG:04510 | Focal adhesion | 0.56 | 1.00 | [FLT1] |
| KEGG:04514 | Cell adhesion molecules (CAMs) | 0.14 | 1.00 | [CDH1, CDH3] |
| KEGG:04520 | Adherens junction | 0.03 | 1.00 | [CDH1, NLK] |
| KEGG:05100 | Bacterial invasion of epithelial cells | 0.26 | 1.00 | [CDH1] |
| KEGG:05200 | Pathways in cancer | 0.72 | 1.00 | [CDH1] |
| KEGG:05202 | Transcriptional misregulation in cancer | 0.55 | 1.00 | [FLT1] |
| KEGG:05213 | Endometrial cancer | 0.22 | 1.00 | [CDH1] |
| KEGG:05216 | Thyroid cancer | 0.15 | 1.00 | [CDH1] |
| KEGG:05218 | Melanoma | 0.26 | 1.00 | [CDH1] |
| KEGG:05219 | Bladder cancer | 0.16 | 1.00 | [CDH1] |
| KEGG:05226 | Gastric cancer | 0.47 | 1.00 | [CDH1] |
| KEGG:05323 | Rheumatoid arthritis | 0.35 | 1.00 | [FLT1] |
| KEGG:03040 | Spliceosome | 0.46 | 1.00 | [SRSF2] |
| KEGG:04064 | NF-kappa B signaling pathway | 0.35 | 1.00 | [TICAM1] |
| KEGG:04120 | Ubiquitin mediated proteolysis | 0.11 | 1.00 | [SOCS3, UBE2O] |
| KEGG:04136 | Autophagy | 0.01 | 0.60 | [ATG10, ATG5] |
| KEGG:04137 | Mitophagy | 0.24 | 1.00 | [ATG5] |
| KEGG:04140 | Autophagy | 0.12 | 1.00 | [ATG10, ATG5] |
| KEGG:04211 | Longevity regulating pathway | 0.31 | 1.00 | [ATG5] |
| KEGG:04213 | Longevity regulating pathway | 0.23 | 1.00 | [ATG5] |
| KEGG:04216 | Ferroptosis | 0.17 | 1.00 | [ATG5] |
| KEGG:04217 | Necroptosis | 0.52 | 1.00 | [TICAM1] |
| KEGG:04380 | Osteoclast differentiation | 0.43 | 1.00 | [SOCS3] |
| KEGG:04620 | Toll-like receptor signaling pathway | 0.37 | 1.00 | [TICAM1] |
| KEGG:04621 | NOD-like receptor signaling pathway | 0.18 | 1.00 | [ATG5, TICAM1] |
| KEGG:04622 | RIG-I-like receptor signaling pathway | 0.35 | 1.00 | [ATG5] |
| KEGG:04630 | JAK-STAT signaling pathway | 0.57 | 1.00 | [SOCS3] |
| KEGG:04668 | TNF signaling pathway | 0.39 | 1.00 | [SOCS3] |
| KEGG:04910 | Insulin signaling pathway | 0.11 | 1.00 | [EXOC7, SOCS3] |
| KEGG:04917 | Prolactin signaling pathway | 0.29 | 1.00 | [SOCS3] |
| KEGG:04920 | Adipocytokine signaling pathway | 0.26 | 1.00 | [SOCS3] |
| KEGG:04930 | Type II diabetes mellitus | 0.17 | 1.00 | [SOCS3] |
| KEGG:04931 | Insulin resistance | 0.37 | 1.00 | [SOCS3] |
| KEGG:04932 | Non-alcoholic fatty liver disease (NAFLD) | 0.48 | 1.00 | [SOCS3] |
| KEGG:04935 | Growth hormone synthesis, secretion and action | 0.39 | 1.00 | [SOCS3] |
| KEGG:05132 | Salmonella infection | 0.24 | 1.00 | [EXOC7, M6PR] |
| KEGG:05133 | Pertussis | 0.27 | 1.00 | [TICAM1] |
| KEGG:05135 | Yersinia infection | 0.42 | 1.00 | [TICAM1] |
| KEGG:05142 | Chagas disease (American trypanosomiasis) | 0.38 | 1.00 | [TICAM1] |
| KEGG:05160 | Hepatitis C | 0.15 | 1.00 | [SOCS3, TICAM1] |
| KEGG:05161 | Hepatitis B | 0.51 | 1.00 | [TICAM1] |
| KEGG:05164 | Influenza A | 0.17 | 1.00 | [SOCS3, TICAM1] |
| KEGG:05165 | Human papillomavirus infection | 1.00 | 1.00 | [TICAM1] |
| KEGG:05167 | Kaposi sarcoma-associated herpesvirus infection | 0.58 | 1.00 | [TICAM1] |
| KEGG:05168 | Herpes simplex virus 1 infection | 0.23 | 1.00 | [SOCS3, SRSF2, TICAM1] |
| KEGG:05235 | PD-L1 expression and PD-1 checkpoint pathway in cancer | 0.06 | 1.00 | [BATF, TICAM1] |

^1^Gene ontology term ID

^2^Gene ontology Term

Table S13 - GO terms related to biological processes

| ^1^GO ID | ^2^GO Term | P-value | Bonferroni Correction | Associated Genes Found |
| --- | --- | --- | --- | --- |
| GO:0050789 | regulation of biological process | 0.64 | 1.00 | [BATF, BVES, CDH13, GPR12, JDP2, PLIN3, SLC7A1, TICAM1, TRABD2B, VRTN, VSX2] |
| GO:0048518 | positive regulation of biological process | 0.32 | 1.00 | [BATF, BVES, CDH13, JDP2, PLIN3, SLC7A1, TICAM1, TRABD2B] |
| GO:0019222 | regulation of metabolic process | 0.24 | 1.00 | [BATF, BVES, CDH13, GPR12, JDP2, TICAM1, TRABD2B, VRTN, VSX2] |
| GO:0006807 | nitrogen compound metabolic process | 1.00 | 1.00 | [ARRDC5, BATF, CDH13, JDP2, LIN52, TICAM1, TK1, TRABD2B, VRTN, VSX2] |
| GO:0044238 | primary metabolic process | 0.82 | 1.00 | [ARRDC5, BATF, CDH13, JDP2, LIN52, TICAM1, TK1, TRABD2B, VRTN, VSX2] |
| GO:0044260 | cellular macromolecule metabolic process | 0.25 | 1.00 | [ARRDC5, BATF, BVES, CDH13, JDP2, LIN52, TICAM1, TK1, TRABD2B, VRTN, VSX2] |
| GO:0034645 | cellular macromolecule biosynthetic process | 0.10 | 0.84 | [BATF, CDH13, JDP2, LIN52, TICAM1, TK1, VRTN, VSX2] |
| GO:0034654 | nucleobase-containing compound biosynthetic process | 0.04 | 0.34 | [BATF, CDH13, JDP2, LIN52, TICAM1, TK1, VRTN, VSX2] |
| GO:0090304 | nucleic acid metabolic process | 0.12 | 0.82 | [BATF, CDH13, JDP2, LIN52, TICAM1, TK1, VRTN, VSX2] |

^1^Gene ontology term ID

^2^Gene ontology Term

Table S14 - GO terms related to immune system

| ^1^GO ID | ^2^GO Term | Term PValue | Bonferroni Correction | Associated Genes Found |
| --- | --- | --- | --- | --- |
| GO:0002376 | immune system process | 1.00 | 1.00 | [BATF, BVES, SLC7A1, TICAM1] |
| GO:0002440 | production of molecular mediator of immune response | 0.03 | 0.31 | [BATF, TICAM1] |
| GO:0072540 | T-helper 17 cell lineage commitment | 0.02 | 0.24 | [BATF] |
| GO:0002443 | leukocyte mediated immunity | 0.09 | 0.52 | [BATF, TICAM1] |
| GO:0046649 | lymphocyte activation | 0.07 | 0.58 | [BATF, SLC7A1, TICAM1] |
| GO:0098542 | defense response to other organism | 0.63 | 1.00 | [BATF, TICAM1] |
| GO:0030101 | natural killer cell activation | 0.14 | 0.72 | [TICAM1] |
| GO:0051251 | positive regulation of lymphocyte activation | 0.06 | 0.52 | [SLC7A1, TICAM1] |
| GO:0002244 | hematopoietic progenitor cell differentiation | 0.02 | 0.21 | [BATF, BVES] |
| GO:0002372 | myeloid dendritic cell cytokine production | 0.01 | 0.16 | [TICAM1] |
| GO:0051607 | defense response to virus | 0.35 | 1.00 | [TICAM1] |
| GO:0042102 | positive regulation of T cell proliferation | 0.15 | 0.59 | [SLC7A1] |
| GO:0043011 | myeloid dendritic cell differentiation | 0.04 | 0.37 | [BATF] |
| GO:0002735 | positive regulation of myeloid dendritic cell cytokine production | 0.01 | 0.16 | [TICAM1] |
| GO:0045190 | isotype switching | 0.08 | 0.56 | [BATF] |
| GO:0045064 | T-helper 2 cell differentiation | 0.03 | 0.32 | [BATF] |

^1^Gene ontology term ID

^2^Gene ontology Term

Table S15 - GO terms related to molecular function

| ^1^GO ID | ^2^GO Term | Term PValue | Bonferroni Correction | Associated Genes Found |
| --- | --- | --- | --- | --- |
| GO:0005488 | binding | 0.27 | 1.00 | [A2M, ARRDC5, ASCC3, BATF, BVES, CDH13, GPR12, JDP2, LRTM1, M6PR, POPDC3, SPATA6, TICAM1, TK1, TRABD2B, VSX2] |
| GO:0005515 | protein binding | 0.47 | 1.00 | [A2M, ARRDC5, CDH13, LRTM1, M6PR, SPATA6, TICAM1, TK1, TRABD2B] |
| GO:0032027 | myosin light chain binding | 0.01 | 0.37 | [SPATA6] |
| GO:0003674 | molecular_function | 1.00 | 1.00 | [A2M, ARRDC5, ASCC3, BATF, BVES, CDH13, GPR12, JDP2, LRTM1, M6PR, POPDC3, SLC7A1, SPATA6, TICAM1, TK1, TMC6, TRABD2B, VSX2] |
| GO:0008201 | heparin binding | 0.14 | 1.00 | [LRTM1] |
| GO:0048495 | Roundabout binding | 0.01 | 0.47 | [LRTM1] |
| GO:0031210 | phosphatidylcholine binding | 0.04 | 1.00 | [GPR12] |
| GO:0004930 | G protein-coupled receptor activity | 1.00 | 1.00 | [GPR12] |
| GO:0043167 | ion binding | 0.63 | 1.00 | [ASCC3, BVES, CDH13, GPR12, POPDC3, TK1, TRABD2B] |
| GO:0042802 | identical protein binding | 0.71 | 1.00 | [CDH13, TK1] |
| GO:0043169 | cation binding | 1.00 | 1.00 | [CDH13, GPR12, TK1, TRABD2B] |
| GO:0042802 | identical protein binding | 0.71 | 1.00 | [CDH13, TK1] |
| GO:0008270 | zinc ion binding | 0.57 | 1.00 | [TK1] |
| GO:0005524 | ATP binding | 0.68 | 1.00 | [ASCC3, TK1] |
| GO:0004797 | thymidine kinase activity | 0.00 | 0.06 | [TK1] |
| GO:0015075 | ion transmembrane transporter activity | 0.32 | 1.00 | [SLC7A1, TMC6] |
| GO:0005290 | L-histidine transmembrane transporter activity | 0.01 | 0.27 | [SLC7A1] |
| GO:0008381 | mechanosensitive ion channel activity | 0.02 | 0.80 | [TMC6] |
| GO:0000064 | L-ornithine transmembrane transporter activity | 0.02 | 0.74 | [SLC7A1] |
| GO:0015181 | arginine transmembrane transporter activity | 0.02 | 0.77 | [SLC7A1] |
| GO:0015189 | L-lysine transmembrane transporter activity | 0.02 | 0.71 | [SLC7A1] |
| GO:0044877 | protein-containing complex binding | 0.29 | 1.00 | [CDH13, M6PR] |
| GO:0019904 | protein domain specific binding | 0.45 | 1.00 | [M6PR] |
| GO:0042802 | identical protein binding | 0.71 | 1.00 | [CDH13, TK1] |
| GO:0043169 | cation binding | 1.00 | 1.00 | [CDH13, GPR12, TK1, TRABD2B] |
| GO:0055100 | adiponectin binding | 0.00 | 0.17 | [CDH13] |
| GO:0045296 | cadherin binding | 0.07 | 1.00 | [CDH13] |
| GO:0005509 | calcium ion binding | 0.53 | 1.00 | [CDH13] |
| GO:0030169 | low-density lipoprotein particle binding | 0.02 | 0.80 | [CDH13] |
| GO:0008270 | zinc ion binding | 0.57 | 1.00 | [TK1] |
| GO:0004797 | thymidine kinase activity | 0.00 | 0.06 | [TK1] |
| GO:0003824 | catalytic activity | 0.16 | 1.00 | [A2M, ASCC3, BVES, TK1, TRABD2B] |
| GO:0005198 | structural molecule activity | 0.45 | 1.00 | [BVES] |
| GO:0065009 | regulation of molecular function | 1.00 | 1.00 | [A2M, BVES, TICAM1] |
| GO:0003682 | chromatin binding | 0.45 | 1.00 | [JDP2] |
| GO:0003700 | DNA-binding transcription factor activity | 0.09 | 1.00 | [BATF, JDP2, TICAM1, VSX2] |
| GO:0043167 | ion binding | 0.63 | 1.00 | [ASCC3, BVES, CDH13, GPR12, POPDC3, TK1, TRABD2B] |
| GO:0097159 | organic cyclic compound binding | 0.80 | 1.00 | [ASCC3, BATF, BVES, JDP2, POPDC3, TK1, VSX2] |
| GO:0097367 | carbohydrate derivative binding | 0.17 | 1.00 | [ASCC3, BVES, LRTM1, POPDC3, TK1] |
| GO:1901363 | heterocyclic compound binding | 0.80 | 1.00 | [ASCC3, BATF, BVES, JDP2, POPDC3, TK1, VSX2] |
| GO:0003676 | nucleic acid binding | 0.77 | 1.00 | [ASCC3, BATF, JDP2, VSX2] |
| GO:0017147 | Wnt-protein binding | 0.03 | 1.00 | [TRABD2B] |
| GO:0019899 | enzyme binding | 0.43 | 1.00 | [A2M, ARRDC5, TICAM1] |
| GO:0043168 | anion binding | 0.51 | 1.00 | [ASCC3, BVES, POPDC3, TK1] |
| GO:0043169 | cation binding | 1.00 | 1.00 | [CDH13, GPR12, TK1, TRABD2B] |
| GO:0051336 | regulation of hydrolase activity | 0.28 | 1.00 | [A2M, BVES] |
| GO:1990756 | ubiquitin ligase-substrate adaptor activity | 0.03 | 0.96 | [ARRDC5] |
| GO:0004386 | helicase activity | 0.16 | 1.00 | [ASCC3] |
| GO:0001228 | DNA-binding transcription activator activity, RNA polymerase II-specific | 0.38 | 1.00 | [BATF] |
| GO:0002020 | protease binding | 0.11 | 1.00 | [A2M] |
| GO:0004175 | endopeptidase activity | 0.23 | 1.00 | [A2M, TRABD2B] |
| GO:0051092 | positive regulation of NF-kappaB transcription factor activity | 0.14 | 1.00 | [TICAM1] |
| GO:0019901 | protein kinase binding | 0.47 | 1.00 | [TICAM1] |
| GO:0031625 | ubiquitin protein ligase binding | 0.25 | 1.00 | [ARRDC5] |
| GO:0004867 | serine-type endopeptidase inhibitor activity | 0.12 | 1.00 | [A2M] |
| GO:0008270 | zinc ion binding | 0.57 | 1.00 | [TK1] |
| GO:0030552 | cAMP binding | 0.00 | 0.01 | [BVES, POPDC3] |
| GO:0032559 | adenyl ribonucleotide binding | 0.08 | 1.00 | [ASCC3, BVES, POPDC3, TK1] |
| GO:0005524 | ATP binding | 0.68 | 1.00 | [ASCC3, TK1] |
| GO:0000977 | RNA polymerase II transcription regulatory region sequence-specific DNA binding | 0.16 | 1.00 | [BATF, JDP2, VSX2] |
| GO:0003924 | GTPase activity | 1.00 | 1.00 | [BVES] |
| GO:0004797 | thymidine kinase activity | 0.00 | 0.06 | [TK1] |

^1^Gene ontology term ID

^2^Gene ontology Term
